# The Optical Properties, UV-Vis. Absorption and Fluorescence Spectra of 4-Pentylphenyl 4-*n*-benzoate Derivatives in Different Solvents

**DOI:** 10.1007/s10895-025-04154-9

**Published:** 2025-02-08

**Authors:** Yadigar Gülseven Sıdır, İsa Sıdır

**Affiliations:** Faculty of Sciences and Letters, Department of Physics, Bitlis Eren University, Bitlis, 13000 Türkiye

**Keywords:** 4-Pentylphenyl 4-*n*-benzoate, Forbidden Energy, Refractive Index, Liquid Crystal, Solvatochromism, Electronic Transitions

## Abstract

In this paper, electronic absorbance and fluorescence spectra of 4-Pentylphenyl 4-*n*-benzoate derivatives have been measured in 29 solvents, which are non-polar, polar protic and polar aprotic solvents, and electronic transitions that vary depending on the solvent are identified. As the solvent polarity increases, the forbidden energy difference between the frontier orbitals decreases. The statistical models in order to describe the solvent effect were derived using different solvent parameters. Quite complex and multiple absorbance transitions were observed in different solvent environments. Local fluorescence transition and intramolecular charge transfer occurred in the fluorescence spectra. Absorbance transitions are global transitions, and π*←π is the absorbance electronic transition. The frontier molecular orbitals and electrostatic potential surface were founded using quantum chemical calculations. Refractive indices were found with five different methods and forbidden energy gaps were found with the Tauc method. The forbidden energy ranges were found around 4.1 and 4.5, and the forbidden energy gap decreased as the alkyl chain became longer. All compounds can be defined as insulation materials according to the forbidden energy range. Refractive index values close to the E7 liquid crystal mixture used in liquid crystal display panels were found in the investigated liquid crystals.

**Supplementary Information:**

The online version contains supplementary material available at 10.1007/s10895-025-04154-9.

## Introduction

Liquid crystal is the state of matter that has the properties of the medium between a solid crystal and an amorphous liquid or isotropic liquid [1]. Liquid crystal molecules generally have a few common properties. If these are the most common, they should be similar in molecular structure. The most common structures of liquid crystals are a rod-like molecular structure, rigidity of the long axis, and strong dipole and/or easily polarizable substituents. Many studies have reported that rod-like liquid crystal in the nematic phase exhibits excellent performance in modern imaging technology [2]. Many factors affect the presence of the liquid crystal phase. In general, the observed liquid crystal phase is basically defined by the geometric shape of the molecule. However, interactions took place between liquid crystal molecules such as intramolecular scattering, repulsive-attractive intermolecular interactions and hydrogen bonds may play an important role. The interaction between molecular electric dipoles produces significant attractive forces between molecules [3–13]. 

Investigations of optical properties of liquid crystal compounds, which have applications such as flat panel displays, digital thermometers, field-effect transistors, which can be classified as organic electronic devices [14, 15], are a very common research topic. The most basic parameters of optical properties are forbidden energy gap and refractive index. These two parameters refractive index and forbidden energy gap can determine with optical calculations using by Uv-vis. absorption spectra [16–22]. 

One of the most general methods of examining electronic effects on the structure of liquid crystals is fluorescence spectroscopy, which is one of the types of emission spectroscopy. Fluorescence spectroscopy is the light or spectrum that can be used for all kinds of materials or molecules, whether organic or inorganic (whether complex structures), that is released as a result of the material spontaneously falling back to the minimum energy state as a result of excitation [23]. It is the process of spontaneous light emission by electronically excited species of a material. In general, fluorescence spectroscopy is widely used in studies on LCs, for example, to replace the backlight component of LCs used in imaging [24–25], for fluorescent LCD materials [26], anisotropic organic light-emitting diodes [27–28], inkjet printing dyes [29], for 1D semiconductors [30], fluorescent LCD gels for photonic applications [31–32] and LCD displays [33]. There are applications such as finding the electric dipole moments of the compounds that can be used using solvatochromic methods [34–36].

In this study, the absorbance and fluorescence spectra of liquid crystals with 3 different structures: 4-Pentlyphenyl-4-methylbenzoate (4PP4metB), 4-Pently-4-pentlybenzoate (4PP4pentB), and 4-Pentylphenyl 4-(octyloxy) benzoate (4PP4octoxB) by studying the solvent effect on liquid crystals, both the electronic structures of liquid crystals and possible intra- and intermolecular interactions have been attempted to be interpreted. In addition, electronic absorbance spectrum, frontier molecular orbitals and electrostatic potential surfaces were calculated using quantum chemical calculations. LSERs models were created and interpreted for each electronic transition. The energy band gap and refractive index were interpreted using semi-empirical methods in different solvent environments. The investigated liquid crystals are characterized by its structural arrangement, which is a nematic liquid crystal and has a nematic phase by aligning directionally without positional order of rod-shaped molecules in optical electronics such as displays (LCDs), and its electronic structure was examined due to its properties and applications in liquid crystal technology. Since the liquid crystals selected in this study have carboxylic acid group, it is aimed to explain how electronic interactions change depending on the solvent and how the length of the alkyl chain affects the electronic transitions. So, both intramolecular and intermolecular interactions to the solvent-solute relationships of liquid crystals have been examined as qualitative and quantitative. The absorbance and fluorescence spectra of the investigated LC compounds were measured in solvent environments with different properties, electronic structure calculations were performed with quantum chemical calculations, and the ground and excited states of these compounds with LC properties were investigated. Here, theoretical electronic structure determination was done by performing quantum chemical calculations.

## Materials and methods

### Materials

**Fig. 1:**
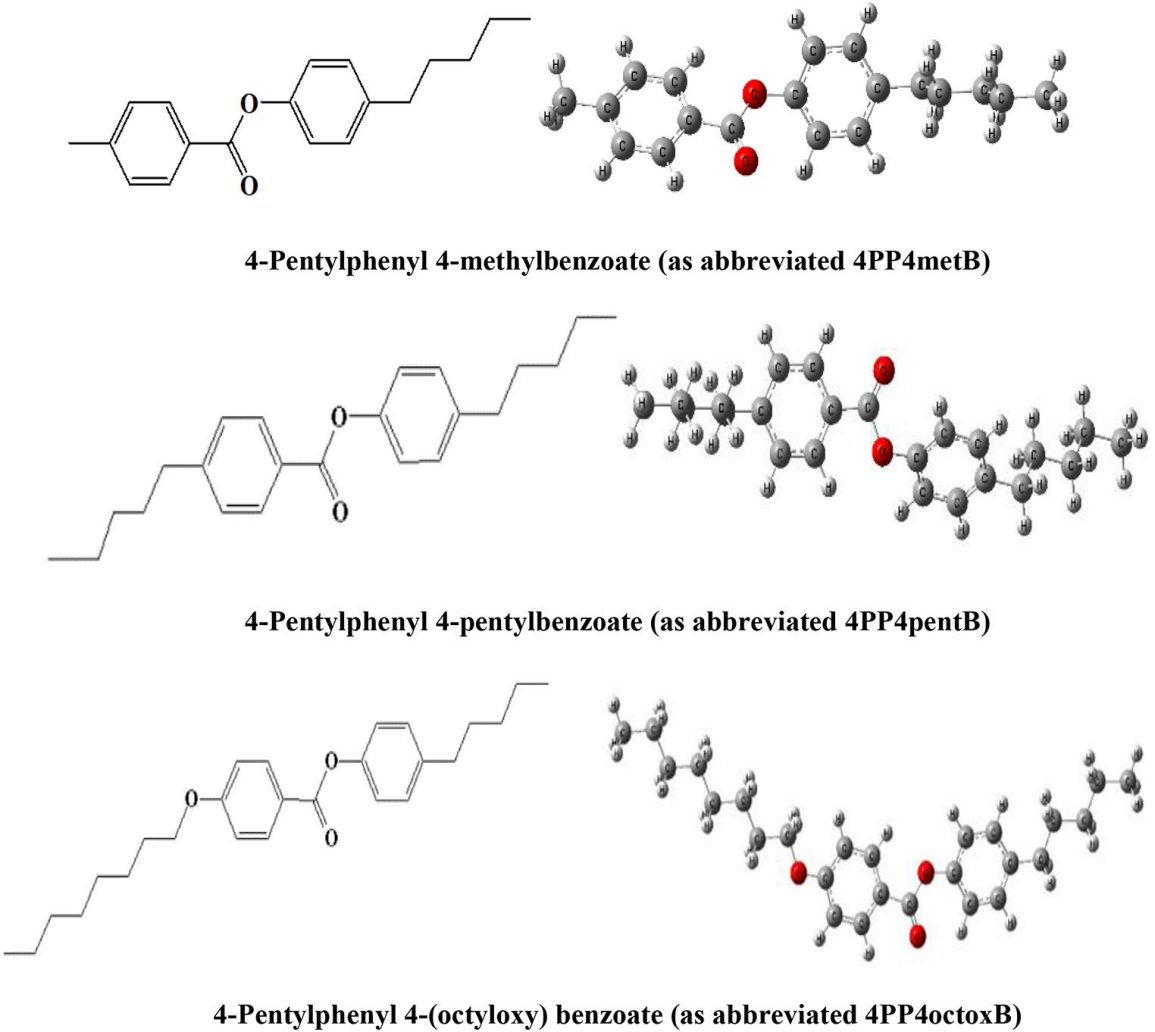
The molecular structure of 4PP4metB, 4PP4propB and 4PP4octoxB LCs

The 4PP4metB, 4PP4pentB and 4PP4octoxB liquid crystals and n-pentane, n-hexane, cyclohexane, 1,4-dioxane, benzene, toluene, o-xylene, diethyl ether, chloroform, ethyl acetate, n-butyl acetate, THF, DCM, 1-octanol, 1-heptanol, 1-hexanol, 1-butanol, iso-butanol, 2-propanol, acetone, 1-propanol, ethanol, benzonitrile, methanol, DMF, acetonitrile, ethylene glycol, DMSO and water solvents were purchased from Sigma-Aldrich and used in our study without any purification. Solvents used in the study were ACS spectrophotometric grade. The molecular structures of 4PP4metB, 4PP4pentB and 4PP4octoxB LCs can be seen from Fig. 1.

### Experimental Method

The solutions consisting of liquid crystal and solvents were prepared as approximately 4 × 10^− 5^ M. In order for the liquid crystals to be completely dissolved in the solutions, the solvents were mixed by using a heater and stirrer, and the liquid crystals were completely dissolved in the solvent. Ultraviolet visible region (UV-vis) absorption spectra were measured room in the wavelength range of 200–800 nm using Perkin Elmer Lambda-35 UV-vis Spectrophotometer. Fluorescence spectra were measured using Perkin Elmer LS-55 Fluorescence Spectrometer. A xenon flash lamp was used as the excitation source and the excitation wavelength was taken differently for each molecule since the excitation wavelength varies according to the molecule. It is seen that the fluorescence spectra of the studied molecules do not show dependence on the excitation wavelength. All measurements were made at room temperature using a 1 cm x 1 cm quartz cell. The obtained spectra were processed using Spectragryph v.1.2.17d and Originpro8 package program. [37, 38]. In fluorescence spectra measurements, excitation wavelengths were selected according to the absorbance bands of the investigated LCs liquid crystals and measured with using 260 nm for 4PP4metB, 250 nm for 4PP4pentB and 280 nm for 4PP4octoxB, respectively.

### Quantum Chemical Calculations

The stable structures of the molecules were determined by geometric optimizations using quantum chemical calculations. These ground state calculations were performed using the B3LYP/6-311G(d, p) method/basis sets [37–41]. Excited state calculations were performed using the TD-DFT (TD-B3LYP)/6-311G(d, p) level of theory [42, 43]. The 6-311G(d, p) basis set includes diffuse and polarization functions. “(d, p)” in “6-311G(d, p)” indicates the existence of diffuse functions (d-type) and polarization functions (p-type). Diffuse functions are added to account for electron density far from the nucleus, while polarization functions improve the description of electron correlation effects. The 6-311G(d, p) basis set containing the polarized function gives results consistent with the experimental results in TD-DFT calculations [44–47]. DFT calculations were done in the gas phase. Quantum chemical calculations were made using the IEFPCM-B3LYP/6-311G(d, p) method and basis set for energy optimization and frequency calculation in benzene environment [48–50].Quantum chemical calculations were performed using Gaussian 09 and GaussView 5.0 software [51–52].

## Result and Discussion

### Electronic Structure and Transitions

**Fig. 2:**
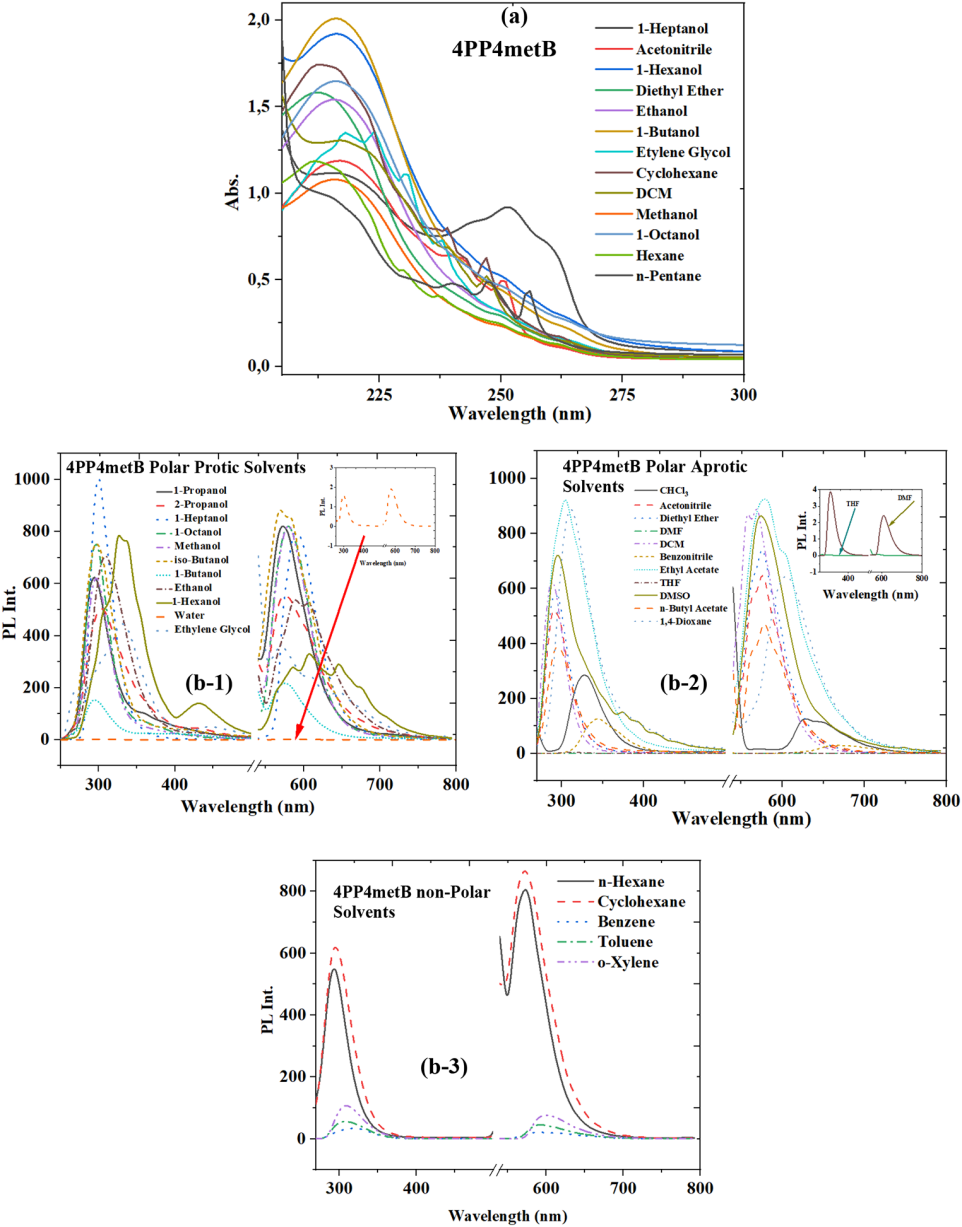
The absorbance (**a**) and fluorescence (**b**-1, **b**-2 and **b**-3) spectra of 4PP4metB LC

**Fig. 3:**
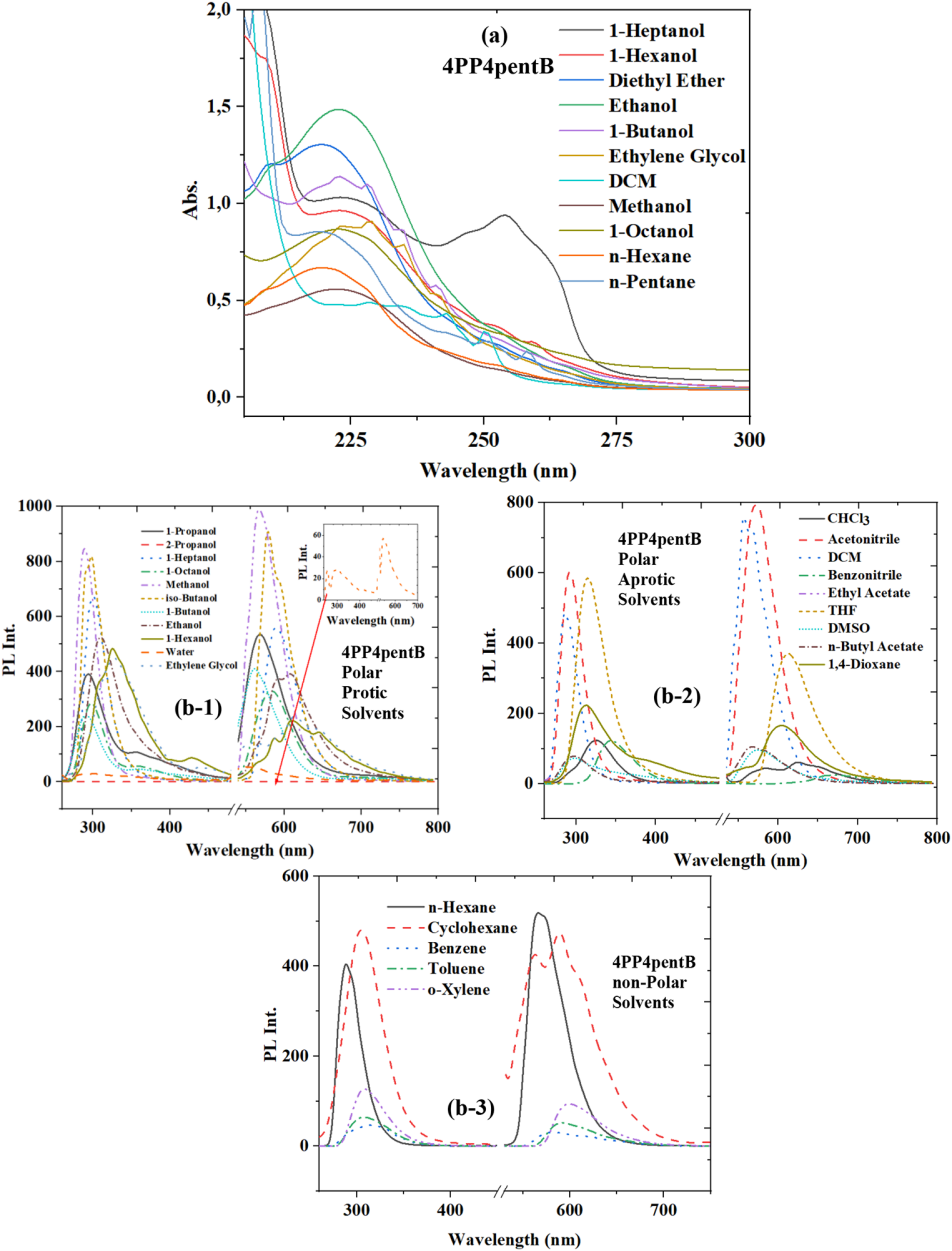
The absorbance (**a**) and fluorescence (**b**-1, **b**-2 and **b**-3) spectra of 4PP4pentB LC

**Fig. 4:**
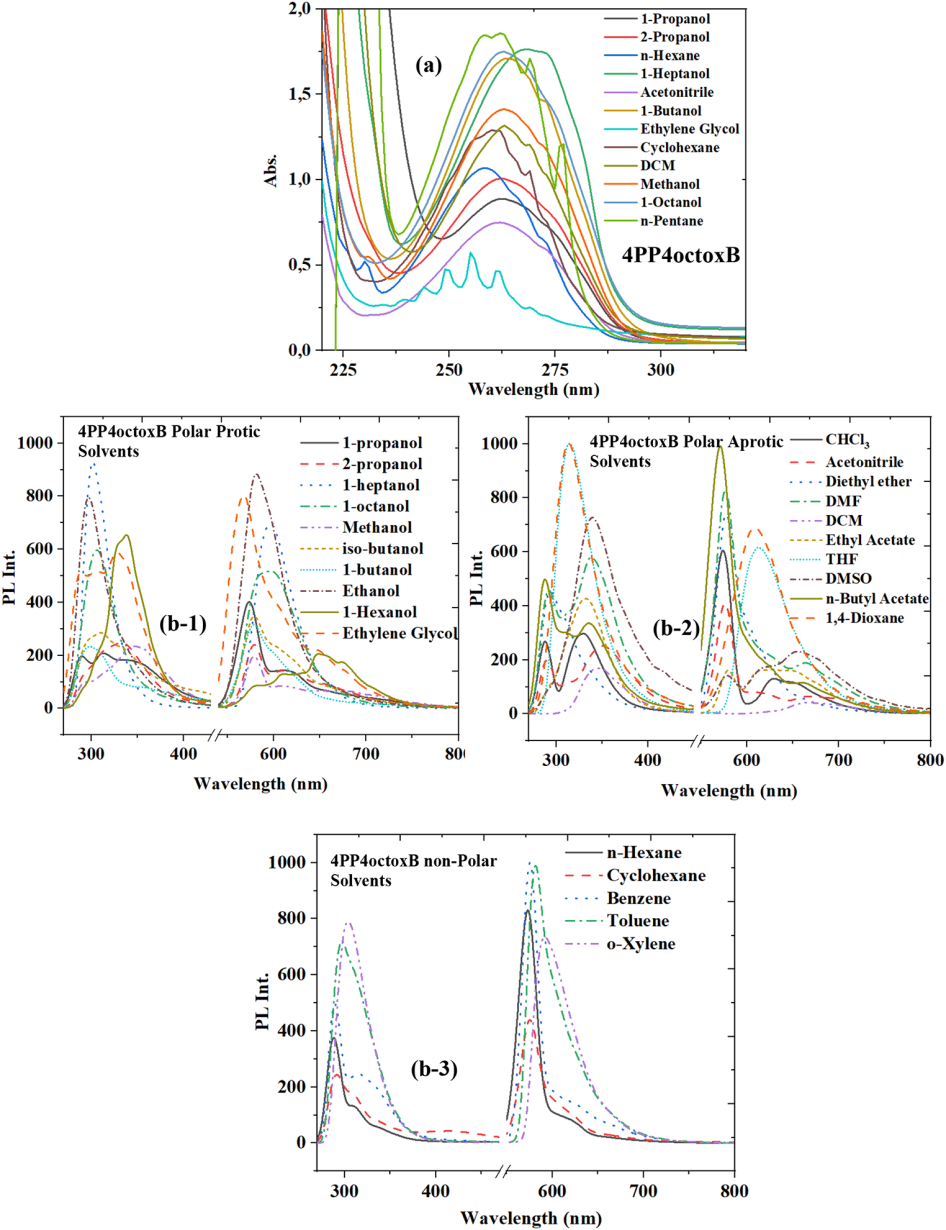
The absorbance (**a**) and fluorescence (**b**-1, **b**-2 and **b**-3) spectra of 4PP4octoxB LC

The electronic fluorescence and absorption spectra of 4PP4metB, 4PP4pentB and 4PP4octoxB LCs in non-polar, polar protic and polar aprotic solvents are shown in Figs. 2, 3 and 4, respectively. Electronic absorption and fluorescence spectra data are listed in Tables 1, 2 and 3. Both absorbance and fluorescence spectra of the investigated LCs in Tables 1, 2 and 3 showed different wavelengths and different numbers of maximum peaks depending on the solvent. If the observed wavelengths are separated according to their transition energies, for 4PP4metB liquid crystal, 211–218 nm (5.8–5.7 eV) for λ_abs1_ in all of solvents and 224–231 nm (5.5–5.3 eV) for λ_abs2_ observed in n-Pentane, n-Hexane (non-polar solvents) and Ethylene glycol polar protic solvent. Third absorbance electronic transition wavelength have observed to range of 248 nm (5.2 eV) from 237 nm (5 eV) (λ_abs3_). The last band was observed between 251 nm (4.9 eV) and 265 nm (4.6 eV), and an extra peak (in 304 nm) was observed in the polar protic solvents 1,4-Dioxane (see Fig. 1s). It can be seen in Fig. 2 that the absorbance spectra of 4PP4metB have observed to three or four electronic transitions bands in n-Pentane, n-Hexane, Cyclohexane, Acetonitrile, 1-Heptanol and Ethylene glycol solvents.

**Table 1:** The wavelength values of absorbance and fluorescence spectra of 4PP4metB LC

Solvents	4pp4metB					
	λ_abs1_	λ_abs2_	λ_abs3_	λ_abs4_	λ_PL1_	λ_PL2_
*n*-Pentane	211	231	240,248	256	295	562, 575, 592
*n*-Hexane	212	230	237	-	293	573
Cyclohexane	214	-	239,247	-	295	528, 572
1, 4-Dioxane	-	-	-	251, 304	312	605
Benzene	-	-	-	-	318	593, 612
Toluene	-	-	-	-	308	593
o-Xylene	-	-	-	-	310	601
Diethyl Ether	212	-	-	-	292	576
Chloroform	-	-	-	-	328	627, 649
Ethyl acetate	-	-	-	-	304	579, 599
*n*-Butyl acetate	-	-	-	-	296	566, 578
THF	-	-	-	257, 265	312	611
DCM	217	-	239, 247	-	288	558, 569
1-Octanol	216	-	-	-	296	580
1-Heptanol	216	-	244	251, 260	299	590
1-Hexanol	216	-	-	-	327, 337, 431	585, 608, 646, 674
1-Butanol	216	-	-	-	295	576
iso-Butanol	-	-	-	-	297	526, 569, 581
2-Propanol	-	-	-	-	303	575
Acetone	-	-	-	-	-	-
1-Propanol	-	-	-	-	293	572
Ethanol	215	-	-	-	310	588, 605
Benzonitrile	-	-	-	-	344	669
Methanol	216	-	-	-	297	530, 580
DMF	-	-	-	-	294	584
Acetonitrile	217	-	241	251	290	565, 576
Ethylene glycol	218	224, 230	238	-	330	570, 605, 645
DMSO	-	-	-	-	296, 374	574
Water	-	-	-	-	297	580

There are 211, 231, 240 and 256 nm peaks in n-Pentane, but in Ethylene glycol (Polar protic solvent) the higher polarity than n-Pentane (non-polar solvent) have observed to absorbance bands in 218, 224, 230 (shoulder peak) and 238 nm. Van der Waals interactions, which is global solvent effect, are electrostatic interactions between solvent and solute molecules that vary depending on the distance. These interactions can be defined as the interaction between solvent polarity and frontier orbitals of the solute. In the bathochromic shift, the solvent polarity increased the energy difference between the frontier orbitals of the dissolved liquid crystal. There are two main fluorescence bands in the 4PP4metB liquid crystal according to Table 1; Fig. 2 (b-1,b-2 and b-3). In the first fluorescence band, it was observed between 288 and 327 nm, except for 1-Hexanol. While three different fluorescence peaks were observed at 327, 337 and 431 nm in 1-Hexanol, it was between 296 and 374 nm in DMSO. The second fluorescence band is a fluorescence transition that is divided into fine structures, or has four wavelengths. Solvent dependent changes were observed in both fluorescence bands. As can be seen from Table 1 and Fig. 2, the 1st absorbance band in both the absorbance and fluorescence spectrum depending on the used solvent vary in the range of 219–223 nm, while the 2nd absorbance band has been observed only Cyclohexane, DCM, 1-Butanol and Ethylene Glycol. The third absorbance band has been observed in n-Pentane, Cyclohexane, DCM, 1-Heptanol and 1-Butanol. We can say that there is a bathochromic shift in the first band. The 2nd fluorescence bands have been observed in 4PP4metB compound, and it can be said that this compound forms a dimer structure [51, 52]. The first fluorescence band has the maximum of the spectrum ranging from 286 nm to 344 nm, while the second fluorescence band has spectrum bands ranging from 510 nm to 670 nm. As seen in Fig. 3, it can be interpreted as the π*← π electronic transition (first electronic transition) of the methoxy group, which is the global transition for the electronic absorption transition of the 4PP4metB molecule.

**Table 2:** The wavelength values of absorbance and fluorescence spectra of 4PP4pentB LC

Solvent	4PP4pentB					
	λ_abs1_	λ_abs2_	λ_abs3_	λ_abs4_	λ_PL1_	λ_PL2_
*n*-Pentane	219	-	244	250, 258	296	576, 589
*n*-Hexane	220	-	-	-	289	566
Cyclohexane	223, 228	235	243, 246	250	305	563, 589
1,4-Dioxane	-	-	-	259, 266, 303	313, 385	564, 605
Benzene	-	-	-	-	313, 325	583, 617
Toluene	-	-	-	-	307	592
o-Xylene	-	-	-	-	309	598
Diethylether	220	-	-	-	-	-
Chloroform	-	-	-	-	325	584, 624, 645
Ethyl acetate	-	-	-	-	310	573, 586, 606
*n*-Butyl acetate	-	-	-	-	298	568, 583
THF	-		-	260; 267	315	612
DCM	228	234	243	250	286	557, 568
1-Octanol	222	-	-	-	298, 356	583, 697
1-Heptanol	224	-	248	254	301	589
1-Hexanol	224	-		-	310, 325, 429	587, 610, 644
1-Butanol	223, 228	234	241	-	291, 358	562, 702
iso-Butanol	-	-	-	-	291	510, 572
2-Propanol	-	-	-	-	304	579
Acetone	-	-	-	-	-	-
1-Propanol	-	-	-	-	294, 357	568
Ethanol	223	-	-	-	310	592, 608
Benzonitrile	-	-	-		344	670
Methanol	222	-	-		289	567
DMF	-	-	-	-	-	-
Acetonitrile	-	-	-	-	291	510, 572
Ethylene glycol	223, 228	235	-	-	328, 441	561, 613, 643
DMSO	-	-	-	-	298, 371	574
Water	-	-	-	-	303	545, 558

**Table 3:** The wavelength values of absorbance and fluorescence spectra of 4PP4octoxB LC

Solvent		4PP4octoxB		
	λ_abs1_	λ_abs2_	λ_PL1_	λ_PL2_
*n*-Pentane	-	258, 262, 269, 276	288, 337	572, 618, 656
*n*-Hexane	209	258	312, 329	612
Cyclohexane	217	261	291, 310	417, 575
1,4-Dioxane	-	-	313	609
Benzene	-	-	289, 314	576, 620
Toluene	-	-	297	581
o-Xylene	-	-	304	592
Diethyl Ether	-	-	294	578
Chloroform	-	264	330	629, 653
Ethyl acetate	-	-	333	580, 629
*n*-Butyl Acetate	-	-	288, 312, 335	571, 659
THF	-	-	314	612
DCM	-	263	291, 338	576, 664
1-Octanol	218	263	306	595
1-Heptanol	-	269	301	596
1-Hexanol	-	-	338	579, 611, 648, 676
1-Butanol	-	264	298, 358	578
iso-Butanol	-	262	311	579
2-Propanol	213	263	334	611, 651
Acetone	-	-	-	-
1-Propanol	-	262	314, 337	612
Ethanol	-	-	297	581
Benzonitrile	-	-	347	668, 692
Methanol	208	263	295, 347	579, 610, 650, 680,713
DMF	-	-	288, 357	573, 671, 707
Acetonitrile	210	262	351	611, 670, 696
Ethylene Glycol	-	240, 244, 249, 255, 261	291, 310	417, 575
DMSO	-	-	339	579, 616, 655
Water	-	-	-	-

As seen in Fig. 3; Table 2, there are four absorbance bands and two fluorescence bands for 4PP4pentB. In 4PP4pentB, the first absorbance band was observed as 219–228 nm (5.6–5.4 eV), while the second absorbance band was observed as 234 nm and 235 nm (5.2 eV) in cyclohexane, DCM, 1-Butanol and Ethylene Glycol. The 3rd absorbance band of 4PP4PentB LC was observed between 214 nm and 248 nm. The last absorbance band was observed between 250 nm (4.9 eV) and 266 nm (4.6 eV). In 1,4-Dioxane, a different band was observed at 303 nm (Fig. 1S). There are two main bands in the 4PP4pentB fluorescence spectrum. Fluorescence peaks in the first band range from 291 to 429 nm. The second fluorescence band exists peaks ranging from 510 nm to 644 nm depending on the solvent. For 4PP4pentB, shoulder peaks with wavelengths of 429 and 441 nm were observed in the 1st fluorescence band in 1-hexanol and ethylene glycol, respectively. However, in the second fluorescence band, a shoulder peak was observed at 702 nm in 1-Butanol and 670 nm in benzonitrile medium. These shoulder peaks are fluorescence electronic transitions that occur as a result of specific interactions between solvent and solute.

Unlike 4PP4metB and 4PP4pentB, there are two main electronic absorbance transition bands in the 4PP4octoxB liquid crystal. The first absorbance peak is between 209 and 218 nm (5.9–5.6 eV). In the absorbance band, there are peaks extending up to 258–276 nm (4.8–4.4 eV). In 4PP4octoxB, n-Pentane and Ethylene Glycol are observed four and five peaks, and the electronic absorbance transitions have fine structured wavelengths. According to the fluorescence spectra, two main bands are observed. First band contains one or two peak except for n-Butyl acetate. The maximum of five fluorescence peaks have observed in the second fluorescence spectrum of 4PP4octoxB LC. Besides, the fluorescence shoulder peak in methanol is 721 nm, which is the highest wavelength.

Figure 4 shows the electronic fluorescence and absorption spectra of the 4PP4octoxB molecule in some selected solvents. As can be seen Fig. 4, it can be interpreted as the π-π* electronic transition, which is due to the methoxy group, which is the global transition for the electronic absorption transition. There are two bands of electronic fluorescence. The short wavelength fluorescence band at C = O is the same as local fluorescence, but the long wavelength fluorescence is the intramolecular charge transfer that occurs with O-C = O. As can be seen from the spectra of the investigated LC compounds, shifts occurred in the electronic transition wavelengths depending on the solvent.

Resultantly, as can be seen from Figs. 2, 3 and 4; Tables 1, 2 and 3, the π*←π electronic transition of the methoxy group for all the molecules studied can be interpreted as the global transition for the electronic absorption transition for the LCs. The lowest energy electronic absorbance transitions can be described as n←π* occurring at C = O. As seen from Figs. 2, 3 and 4, there are two electronic fluorescence bands. The short wavelength fluorescence band at C = O is local fluorescence, but the long wavelength fluorescence is intramolecular charge transfer that occurs in the O-C = O band.

Solvents have a global effect on the boundary molecular orbitals of solute molecules through polarity (polarity induction) and polarizability (dispersion orientation effect), while the solvent acts as a hydrogen bond donor ability and hydrogen bond acceptor ability. They specifically affect solute molecules. These effects affect not only the energy of the boundary orbitals of solvated molecules but also the wavelength and number of peaks of electronic transitions. To better understand solvent-solute interactions, absorbance and fluorescence spectra of non-polar, polar protic and polar aprotic solvents were examined in different solvent groups (Figs. 2, 3 and 4(b)).

### Solute Effect on Absorbance and Fluorescence Spectra

Solvent effect has been determined as the changes in the electronic transitions of the investigated compound depending on the solvent. It is explained into mechanism in wavelength shifts in the compound by looking at the electronic spectra. The linear solvation energy relationships can determine the contribution of solvent dielectric constant, refractive index, hydrogen bond acceptor, hydrogen bond donor parameters on spectra. The specific/nonspecific interactions and intra/inter molecular interactions in electronic transition are determines via LSERs. The LSERs approach is preferable because it has been successfully applied to the positions or intensities of absorption maxima in UV–visible absorption and fluorescence spectra [53–63].

We tried to establish a correlation between experimental spectral values with four solvatochromic variables of the polarity function, electronic polarizability function, H-bonding donor ability and H-bonding acceptor ability in order to evaluate respective contributions to the solute–solvent interactions of the LC compounds. In this case, the position of the absorption or fluorescence spectrum band (ν_max_) in a given solvent was used in solvatochromic models shown below. The statistical models were derived using SPSS15.0 for Windows Evaluation Version [62].

KAT solvatochromism can be the LSERs model, which is found by expanding the polarity and polarizability parameters give as detail to π* parameter [58–61].

1 $$\upsilon_{max}\hspace{0.17em}=\hspace{0.17em}C_{0}+C_{1}f\left(n\right)\hspace{0.17em}+\hspace{0.17em}C_{2}f\left(\epsilon\right)\hspace{0.17em}+\hspace{0.17em}C_{3}\hspace{0.17em}\beta+\hspace{0.17em}C_{4}\alpha$$

The coefficient C_1_ (polarizability or dispersion-polarization) and C_2_ (polarity or orientation-induction) show specific interactions, while C_3_ (Hydrogen bonding acceptor) and C_4_ (Hydrogen bonding donor) show non-specific interactions [60].

Catalán Solvatochromism;

2 $$\upsilon_{max}\hspace{0.17em}=\hspace{0.17em}C_{5}+C_{6}SP+C_{7}SdP+C_{8}SA+C_{9}SB$$

The C_6_ (polarizability of solvent) and C_7_ (dipolarity of solvent) solvatochromic coefficients show the effect of global interactions, while C_8_ (solvent of acidity) and C_9_ (solvent of basicity) show the effect of non-global interactions. The C_0_ and C_5_ coefficients are the gas phase property of the compound, which is the same as the dependent variable. Statistic calculations have been done in SPSS15 packet program. The used parameters values in LSERs calculations have been listed in Table 1S.

**Fig. 5:**
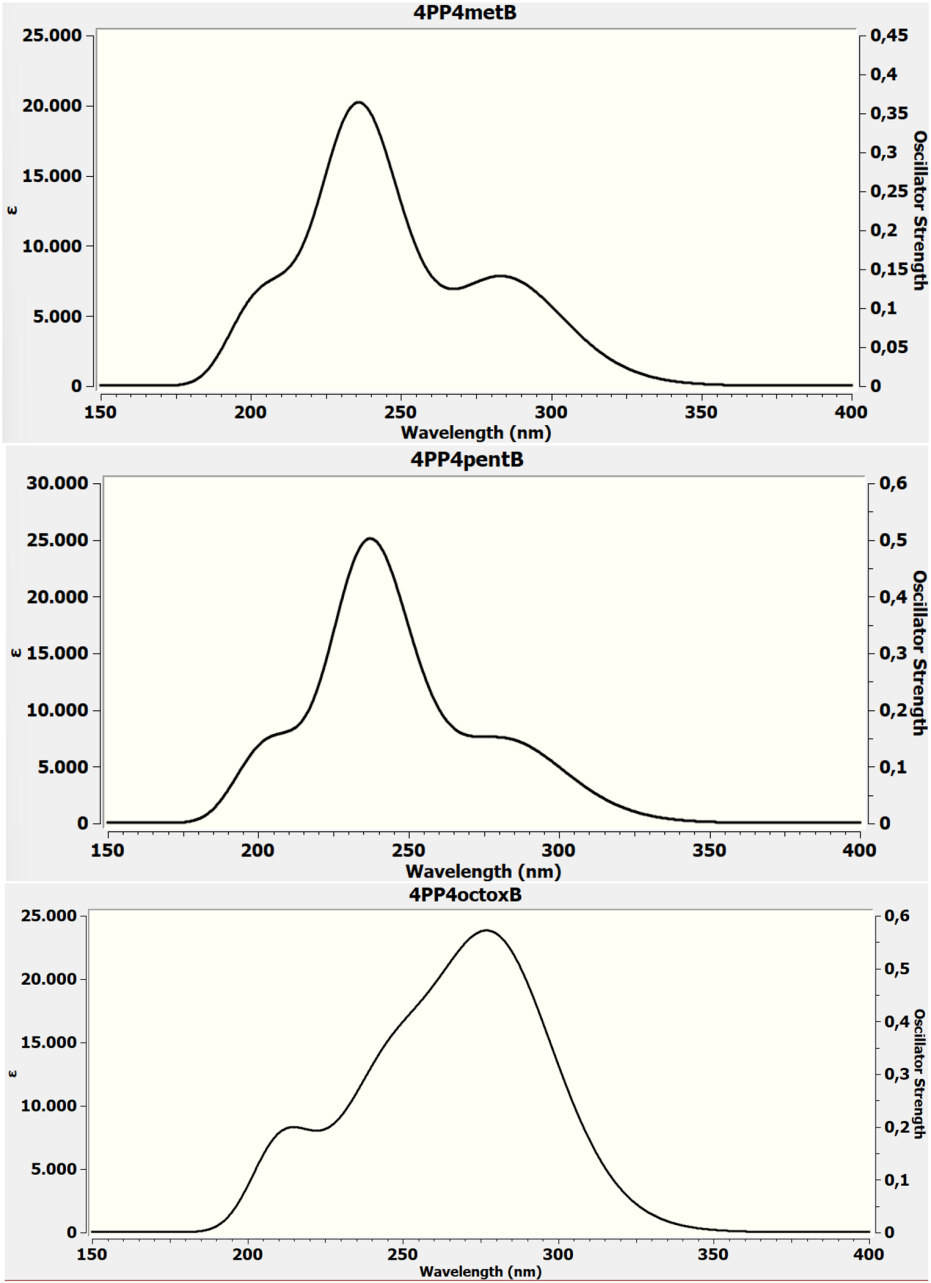
The electronic absorption spectra of LCs calculated by TD-DFT (B3LYP)/6-311G(d, p) in the gas phase

**Fig. 6:**
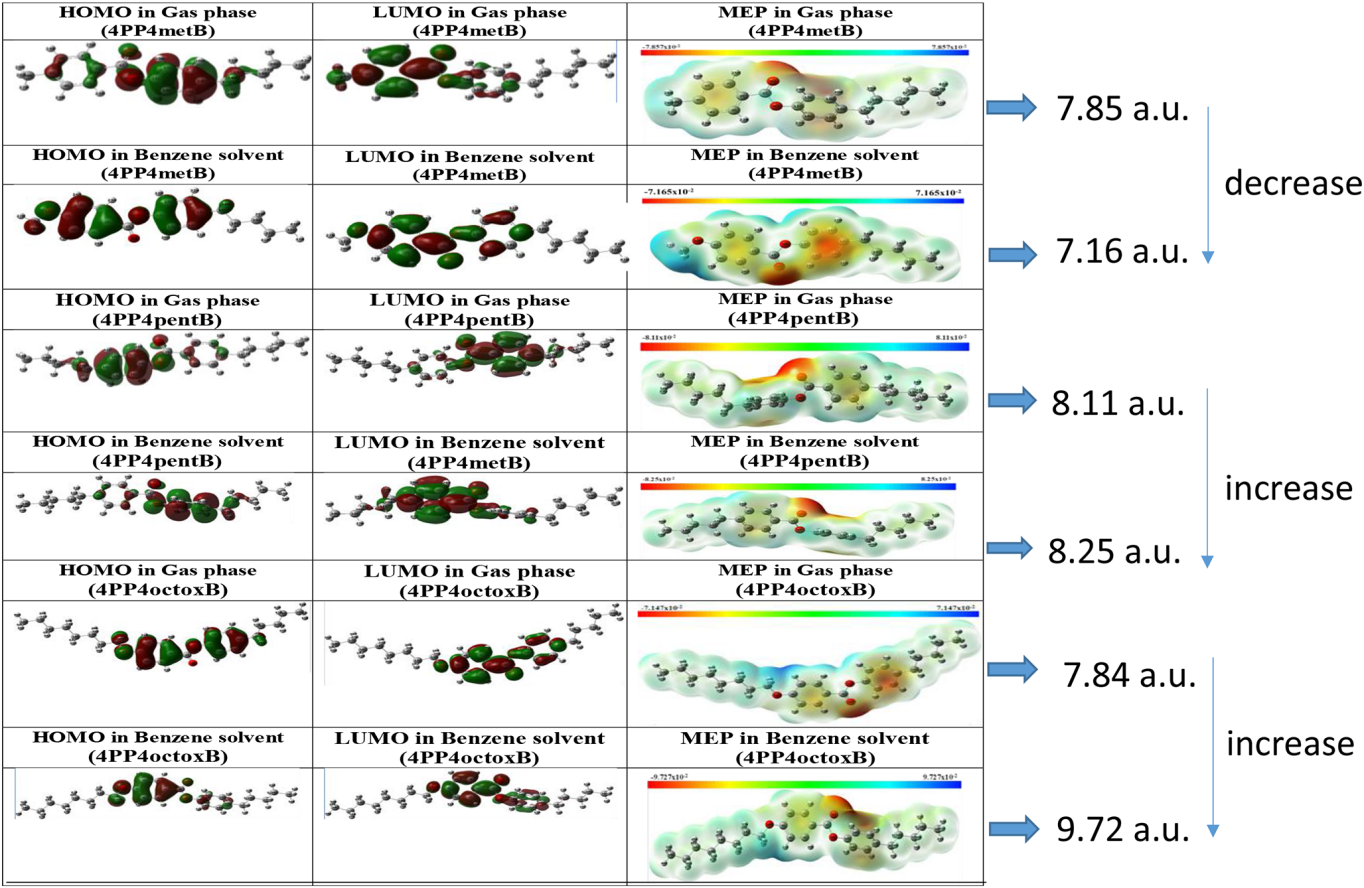
HOMO, LUMO and MEP shapes of 4PP4metB, 4PP4pentB and 4PP4octoxB LCs calculated using the B3LYP/6-311G(d, p) method/basis set

**Fig. 7:**
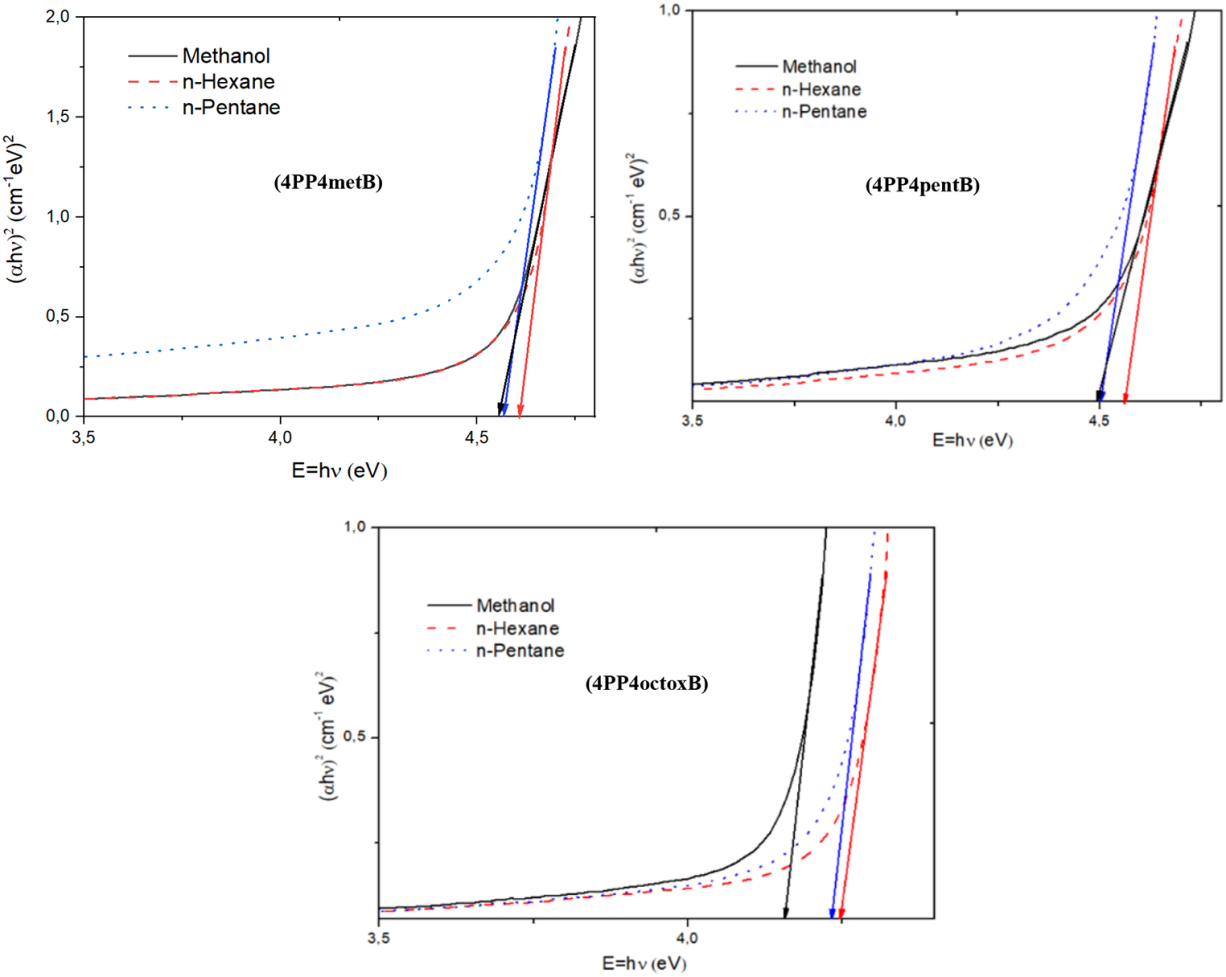
The graph (αhν)^2^ versus E = hν of 4PP4metB, 4PP4pentB and 4PP4octoxB LCs

In this study, before LSER calculations were done, the linear correlations between both absorbance and fluorescence transitions of 8 independent parameters ( *f(n)*, *f(ε)*, *β*, *α*, *SP*, *SdP*, *SA* and *SB*) were investigated. These correlation graphs are shown in Fig. 2-27 S in Supplementary Material. We can say from Fig. 2S that the correlation between *f(ε)* and ν_exp_ is better for the Abs1 wavenumber of 4PP4metB compound. As seen from Fig. 3S, among the correlations of Abs1 with the Catalán parameters for 4PP4metB LC, the correlation between *SP* and *ν*_*exp*_ was found to be better than the correlation between *SdP* and *ν*_*exp*_. For Abs1 of 4PP4metB compound, the *SA* and *SB* parameter, which describes specific interactions, has a better *SA* correlation than *SB*. The correlation value between *f(ε)* and ν_exp_ for 4PP4metB PL1 is R^2^ = 0.7159, which is a good correlation value while PL1 of 4PP4metB compound does not have a good correlation between ν_exp_ for *α*, *f(n)* and *β* (see Fig. 4S). Considering the correlation value with the Catalan parameters, a good correlation is found between SB and ν_exp_, which only describe specific interactions, with the correlation value of these two parameters R^2^ = 06805 (see Fig. 5S). When the correlations between PL2 molecular fluorescence spectroscopy wavenumber of 4PP4metB compound and KAT parameters were evaluated, no suitable correlation was hinged. As seen from Fig. 6S, however, there is a correlation of R^2^ = 0.4684 between *f(n)* and ν_exp_. No good correlation was found with Catalán parameters for 4PP4metB PL2 wavenumbers. However, there is a correlation of R^2^ = 0.475 between *SP* and ν_exp_ (see Fig. 7S). As in Fig. 8S, when the correlation between the photoluminescence 3rd wavenumber and KAT parameters for the 4PP4metB compound is examined separately, no correlation close to R^2^ > 0.7 was observed. In Fig. 9S, it can be said that the 4PP4metB PL3 correlation for the Catalán parameters is better than the correlation of the KAT parameters.

Figure 10S and 15S show separate correlations for both KAT and Catalán parameters for the electronic absorbance and fluorescence wavelengths of the investigated 4PP4pentB compound. While no satisfactory correlation was found between Abs1 band and KAT parameters, when we looked at Catalán parameters, a correlation of 0.559 was found between solvent polarizability and experimental wavenumber. That is, in the 1st band of absorbance, solvent polarization is quite dominant among the Catalán parameters. There is no correlation with the KAT parameters for the 4PP4pentB PL1 fluorescence band, while the solvent polarity is quite dominant in Catalán. While there was no correlation with the KAT parameters for the 4PP4pentB PL1 fluorescence band, there was a correlation between solvent polarizability and R^2^ = 0.6672 for Catalán. As seen in Fig. 13S S, correlation graphs between 4PP4pentB and λ_PL2_, no correlation was observed when looking at the correlation of both KAT parameter and Catalán parameter.

The graphs of the wavenumbers showing the electronic transition energies of λ_Abs1_, λ_PL1_, λ_PL2_, λ_PL3_, λ_PL4_ and λ_PL5_ of the 4PP4octoxB molecule, the correlations between the KAT parameters (see Fig. 16S, 18 S, 20 S, 22 S, 24 S and 26 S) and the correlations between the Catalán parameters (see Fig. 17S, 19 S, 21 S, 23 S, 25 S and 27 S) can be seen in Figs. 16, 17, 18, 19, 20, 21, 22, 23, 24, 25 and 26, 27 S. In general, there is no correlation between individual parameters and wavenumbers. However, while the correlation between PL2 wavelength and *SP* was found to be R^2^ = 0.5913, the relationship between *f(ε)* and ν_exp_ for PL3 was found to be R^2^ = 0.6361.

Since the absorbance and fluorescence frequencies of the investigated molecules and solvent parameters could not be individually correlated, multiple linear regression analysis was performed, and it was aimed to find the correlation value on the derived LSER models R^2^ > 0.7. Thus, the coefficients and statistical parameters of the models seen in Tables 4 and 5 were found. The magnitudes and signs of the coefficients of the KAT and Catalán parameters of the equations found for the LSER models were found by multiple linear regression analysis. As can be seen from Tables 4 and 5, C_0_ and C_5_ values represent the wavelengths of the investigated compounds in gas or inert medium [16]. Solvents used in deriving LSER models are given below the Tables 4 and 5. The magnitude and sign of the coefficients C_i_ can indicate the degree of the different solute–solvent interactions. Their values for each absorbance and fluorescence band are summarized in Tables 4 and 5.

**Table 4:** LSERs models using KAT parameters, LSERs coefficients and statistical’ results

Liquid Crystals	Wavelength	C_0_	C_1_	C_2_	C_3_	C_4_	*R*	*R* ^2^	F	*P*	Number of Solvents
4pp4metB	λ_abs1_	50,148	-11,301	-1703	810	-439	0.98	0.97	65.18	0.000	13^a^
	λ_PL1_	33,183	-4363	1946	207	-197	0.86	0.74	5.79	0.017	13^b^
	λ_PL2_	21,182	-13,470	-1884	1016	11.82	0.84	0.73	9.89	0.000	20^c^
	λ_PL2−s_	19,617	-9369	-1695	336	258	0.85	0.73	2.02	0.295	8^d^
4pp4pentB	λ_abs1_	49,158	-14,218	-2288	1274	-0.39	0.91	0.83	8.98	0.007	12^e^
	λ_PL1_	40,016	-21,934	-4341	165	1469	0.84	0.71	6.95	0.005	17^f^
	λ_PL2_	18,501	-5130	404	-1089	766	0.87	0.76	9.94	0.001	18^g^
4pp4octoxB	λ_abs1_	38,979	-242	-3493	566	-403	0.84	0.71	1.88	0.315	8^h^
	λ_PL1_	34,407	-3229	-6484	656	5054	0.88	0.78	7.21	0.009	13^I^
	λ_PL2_	30,104	3543	-3346	1145	652	0.83	0.69	2.82	0.142	10^i^
	λ_PL3_	17,405	121	-110	-133	36	0.84	0.71	4.44	0.042	13^k^
	λ_PL4_	16,683	-1057	-1003	1038	109	0.86	0.74	5.72	0.018	14^l^
	λ_PL5_	15,048	-748	1842	293	410	0.84	0.80	5.99	0.027	11^m^

^a^ n-Pentane, n-Hexane, Cyclohexane, Diethylether, DCM, 1-Octanol, 1-Heptanol, 1-Hexanol,1-Butanol, Methanol, Acetonitrile, DMSO and Ethylene glycol

^b^ 1,4-Dioxane, Toluene, Ethylacetate, n-Butylacetate, 1-Octanol, 1-Heptanol, 1-Butanol, iso-Butanol, 1-Propanol,

Methanol, DMF, Acetonitrile and DMSO

^c^ n-Pentane, n-Hexane, Benzene, Toluene, o-Xylene, Diethyl Ether, Ethyl acetate, n-Butyl acetate, 1-Octanol, 1-

Heptanol, 1-Hexanol, 1-Butanol, iso-Butanol, 2-Propanol, 1-Propanol, Ethanol, Benzonitrile, Methanol, DMF,

Acetonitrile and Water

^d^ n-Pentane, 1,4-Dioxane, Benzene, Chloroform, Ethyl acetate, THF, 1-Hexanol and Ethanol

^e^n-Pentane, n-Hexane, Cyclohexane, Diethylether, DCM, 1-Octanol, 1-Heptanol, 1-Hexanol, 1-Butanol, Ethanol, Methanol and Ethylene glycol

^f^n-Pentane, n-Hexane, Cyclohexane, 1,4-Dioxane, Benzene, Toluene, o-Xylene, Ethyl acetate, n-Butyl acetate, THF

DCM, 1-Octanol, 1-Hexanol, 2-Propanol, Ethanol, Benzonitrile and Ethylene glycol

^g^n-Pentane, n-Hexane, Benzene, Toluene, o-Xylene, Chloroform, Ethyl acetate, n-Butyl acetate, DCM, 1-Octanol, 1-Hexanol, iso-Butanol, 2-Propanol, 1-Propanol, Ethanol, Methanol, Acetonitrile and Water

^h^n-Pentane, Chloroform, DCM, iso-Butanol, 2-Propanol, 1-Propanol, Methanol and Acetonitrile

^ı^n-Hexane, Cyclohexane, 1,4-Dioxane, Benzene, Chloroform, Ethyl acetate, 1-Octanol, 1-Heptanol, 1-Butanol, iso-Butanol, 1-Propanol, Ethanol and Ethylene Glycol

^i^n-Pentane, n-Hexane, n-Butyl Acetate, DCM, 2-Propanol, 1-Propanol, Benzonitrile, Methanol, DMF and Acetonitrile

^k^n-Hexane, Cyclohexane, Benzene, Diethylether, Ethyl acetate, DCM, 1-Hexanol, 1-Butanol, iso-Butanol, Ethanol, Methanol, DMSO and Water

^l^n-Pentane, n-Hexane, 1,4-Dioxane, Benzene, Chloroform, Ethyl acetate, THF, 1-Octanol, 1-Heptanol, 1-Hexanol, 2-Propanol, 1-Propanol, Methanol and DMSO

^m^n-Pentane, Chloroform, n-Butyl Acetate, DCM, 1-Hexanol, 2-Propanol, Benzonitrile, Methanol, DMF, Acetonitrile and DMSO

**Table 5:** Derived LSERs models using CAT parameters, LSERs coefficients and statistical’ results

Liquid Crystals	Wavelength	C_5_	C_6_	C_7_	C_8_	C_9_	*R*	*R* ^2^	F	*P*	Number of Solvents
4pp4metB	λ_abs1_	49,331	-3521	-293	-189	-307	0.91	0.82	8.49	0.008	12^a^
	λ_PL1_	34,671	-13	24	-478	1036	0.85	0.73	4.13	0.060	11^b^
	λ_PL2_	20,623	-4487	-1608	1456	504	0.85	0.72	6.51	0.008	21^c^
	λ_PL−s_	19,683	-3615	-1219	940	-178	0.91	0.84	6.76	0.030	12^d^
4pp4pentB	_λabs1_	48,361	-4759	-1108	1169	51	0.88	0.77	6.17	0.019	12^e^
	λ_PL1_	43,187	-14,593	-1984	1106	1217	0.85	0.73	10.30	0.000	21^f^
	λ_PL2_	17,920	-404	-1283	1609	336	0.87	0.76	7.43	0.006	14^g^
4pp4octoxB	λ_abs1_	40,694	-4105	615	1267	1116	0.85	0.73	3.41	0.105	10^h^
	λ_PL1_	34,273	-1935	-4819	4911	887	0.83	0.70	4.03	0.052	12^ı^
	λ_PL2_	30,577	-3407	3831	-156	902	0.85	0.72	2.67	0.181	9^i^
	λ_PL3_	17,555	-145	-227	346	-229	0.84	0.71	5.69	0.014	14^k^
	λ_PL4_	15,586	955	-702	737	604	0.86	0.74	6.50	0.010	14^l^
	λ_PL5_	14,826	728	474	796	174	0.85	0.73	4.10	0.061	11^m^

^a^n-Pentane, n-Hexane, Cyclohexane, Diethylether, DCM, 1-Octanol, 1-Heptanol, 1-Hexanol, 1-Butanol, Methanol, Ethylene glycol and DMSO

^b^n-Butylacetate, DCM,1-Octanol,1-Heptanol,1-Butanol, iso-Butanol, Methanol, DMF, Acetonitrile, DMSO and Water

^c^n-Pentane, n-Hexane, Benzene, Toluene, o-Xylene, Diethyl Ether, Ethyl acetate, n-Butyl acetate, 1-Octanol, 1-Heptanol, 1-Hexanol, 1-Butanol, iso-Butanol, 2-Propanol, 1-Propanol, Ethanol, Benzonitrile, Methanol, DMF, Acetonitrile and Water

^d^n-Pentane, Cyclohexane,1,4-Dioxane, Benzene, Chloroform, Ethyl acetate, n-Butyl acetate, THF, DCM, 1-Hexanol, Ethanol and

Ethylene glycol

^e^n-Pentane, n-Hexane, Cyclohexane, Diethyl ether, DCM, 1-Octanol,1-Heptanol, 1-Hexanol, 1-Butanol, Ethanol, Methanol and

Ethylene glycol

^f^n-Pentane, n-Hexane, Cyclohexane, 1,4-Dioxane, Benzene, Toluene, o-Xylene, Ethyl acetate, n-Butyl acetate, THF, 1-Octanol, 1-Hexanol, 1-Butanol, iso-Butanol, 2-Propanol, 1-Propanol, Ethanol, Benzonitrile, Methanol, Ethylene glycol and

DMSO

^g^n-Hexane, Cyclohexane, 1,4-Dioxane, Benzene, Toluene, o-Xylene, Chloroform, Ethyl acetate, n-Butyl acetate, iso-Butanol, 2-propanol,

1-propanol, Methanol and Ethylene glycol

^h^n-Pentane, Chloroform, DCM, 1-Heptanol, 1-Butanol, iso-Butanol, 2-propanol, 1-propanol, Acetonitrile and Ethylene glycol

^ı^n-Pentane, n-Hexane, Cyclohexane, 1,4-Dioxane, Benzene, Chloroform, Ethyl acetate, 1-Octanol, 1-Heptanol, iso-Butanol, 1-Propanol and Ethylene Glycol

^i^n-Hexane, n-Butyl Acetate, DCM, 1-Hexanol, 1-Propanol, Benzonitrile, Methanol, DMF and Acetonitrile

^k^n-Pentane, Cyclohexane, Benzene, Toluene, o-Xylene, Diethylether, Ethylacetate, DCM, 1-Hexanol, 1-Butanol, iso-Butanol, Ethanol,

Methanol and DMF

^l^n-Pentane, n-Hexane, 1,4-Dioxane, Benzene, Chloroform, Ethylacetate, THF, 1-Octanol, 1-Heptanol, 1-Hexanol, 2-propanol, 1-propanol, Methanol and DMSO

^m^n-Pentane, Chloroform, n-Butylacetate, DCM, 1-Hexanol, 2-propanol, Benzonitrile, Methanol, DMF, Acetonitrile and DMSO

When we look at Table 4, solvent polarity is effective only in λ_Abs1_ and λ_PL5_ electronic transitions of 4PP4octoxB compound, while solvent polarization is effective in other electronic transitions. Except for the λ_PL2_ and λ_PL3_ electronic transitions of the 4PP4octoxB compound, the C_1_ coefficient in the LSER models applied for the electronic transitions of other compounds has a negative sign, so the experienced compounds a bathochromic shift due to the effect of the orientation induction interaction. If we look at the hydrogen bond acceptor and hydrogen bond donor coefficients that define specific interactions from solvent-solute interactions, we can see that 4PP4metB has all electronic transitions, 4PP4pentB has λ_Abs1_ and λ_PL1_ electronic transition wavelengths, 4PP4octoxB has λ_PL3_ electronic transition wavelength and all electronic transition wavelengths except λ_PL4_ electronic transition wavelength. In these transitions, the hydrogen bonding acceptor capacity of the solvents has much more effect on the mentioned electronic transitions than the hydrogen bonding donor capacity. We can say that in electronic transitions, the negative coefficient of C_3_ shifts to a higher wavelength depending on the hydrogen bonding capacity.

As seen in Table 5, for the λ_Abs1_, λ_PL2_ and λ_PL−S_ electronic transitions of 4PP4metB, the λ_Abs1_, λ_PL3_, λ_PL4_ and λ_PL5_ electronic transitions of 4PP4pentB, and the λ_Abs1_ and λ_PL1_ electronic transitions of 4PP4octoxB have coefficients │C6│>│C7│, so, the solvent polarization of these electronic transitions is more effective than the solvent dipolarity. As the solvent polarity increases, the energy difference between the molecular orbitals where the electronic transition occurs decreases, this effect is the hypsochromic effect. We can say that the C_6_ coefficient is also negative. However, it is seen from the CAT model that solvent polarity dominates the solvent polarization ability in the λ_PL2_ of 4PP4pentB and, λ_PL2_, λ_PL3_ and λ_PL1_ electronic transitions of 4PP4octoxB.

Considering the C_8_ and C_9_ coefficients, we can define as effect of C_8_ solvent acidity and C_9_ solvent basicity on electronic transitions. Thus, λ_PL2_ and λ_PL−S_ transition transitions of 4PP4metB compound, and λ_ABS1_, λ_PL1_, λ_PL3_, λ_PL4_ and λ_PL5_ electronic transitions of 4PP4octoxB compound, solvent acidity in these electronic transitions has more influence than solvent basicity. For these mentioned transitions, it is seen from Table 5 that the coefficient from C_8_ is positive. Solvent acidity produced hypsochromic effect, that is, as the solvent acidity increases, the energy difference between the molecular orbitals in which these transitions occur decreases. Since the other electronic transitions and λ_PL2_ electronic transitions of 4PP4metB are C_9_│>│C_8_│, thus solvent basicity for these electronic transitions is more effective than solvent acidity. As can be seen from Table 5, since the C_9_ coefficient is positive for the fluorescence transitions of λ_PL1_ of 4PP4metB and λ_PL2_ of 4PP4octoxB, in this situation there is a bathochromic effect.

### Theoretical Absorbance Spectra

The transitions observed is absorption spectra can be defined by dipole-dipole transitions and there are some selection rules. The most important of these is that the spin quantum number must be zero after the transition. In other words, the electron that is up (S = 1/2) and down (S=-1/2) must go to the excited state as up and down. In this case, the spin quantum number is equal to 0. These transitions are singlet transitions. If the sum of the spin quantum numbers in the excited state is ± 1, this transition is a triplet state that makes an unauthorized electronic transition. Table 6 lists the electronic transitions of LCs, their spin and spatial symmetry, transition wavelengths and energies, and oscillator strengths calculated with B3LYP/6-311G (d, p). Since liquid crystals are large, experimental results are different, and a large number of absorption wavelengths are observed, nstate = 10 was selected. However, those with oscillator strengths greater than 0.1 were added to Table 6. Quantum chemical calculations were performed in the gas phase with the TD-B3LYP/6-311G(d, p) method and basis set without any singlet or triplet selection. For 4PP4metB, there is 1 triplet transition at 285.12 nm, four different electronic transitions contributing to 236.94 nm and two different electronic transitions contributing to 211.01 nm. For 4PP4pentB liquid crystal, there is only 1 transition with energy of 283.64 nm and 4.3711 eV from Singlet-A type transitions with oscillator strength greater than 0.1, two different transitions at 237.52 nm and 5.2199 eV and finally two different transitions at 207.75 nm and 5.9679 eV. For 4PP4octoxB, differently from other liquid crystals, there is a triplet transition at 280.61 nm and 4.4184 eV. The transition of 4PP4octoxB at 250.80 nm and 4.9435 eV is provided by five different electronic transitions. Singlet-A type transitions were observed at two different wavelengths of 248.15 nm and 211.70 nm Table 7.

**Table 6:** The electronic transitions, spin and spatial symmetry, transition wavelengths&energies and oscillator strengths of LCs calculated with B3LYP/6-311G (d, p)

4PP4metB				
Excited States	Triplet-A	Energy (eV)	Wavelength (nm)	*f*
HOMO -> LUMO	0.70292	4.3485	285.12	0.1862
	**Singlet-A**			
HOMO-3 -> LUMO	-0.10343	5.2328	236.94	0.4238
HOMO-3-> LUMO + 1	0.15613			
HOMO-2 -> LUMO	0.65079			
HOMO -> LUMO + 2	-0.11886			
HOMO-1 -> LUMO + 2	-0.33380	5.8758	211.01	0.1049
HOMO -> LUMO + 3	0.60536			
4PP4pentB				
Excited States	**Singlet-A**	**Energy (eV)**	**Wavelength (nm)**	***f***
HOMO -> LUMO	0.70173	4.3711	283.64	0.1722
HOMO-2 -> LUMO + 1	0.14140	5.2199	237.52	0.5702
HOMO-2 -> LUMO	0.66097			
HOMO-1 -> LUMO + 2	-0.32870	5.9679	207.75	0.1244
HOMO -> LUMO + 3	0.60550			
4PP4octoxB				
Excited States	**Triplet-A**	**Energy (eV)**	**Wavelength (nm)**	***f***
HOMO -> LUMO	0.69902	4.4184	280.61	0.5371
HOMO-4 -> LUMO	-0.21985	4.9435	250.80	0.1085
HOMO-2 -> LUMO	0.37760			
HOMO-2 -> LUMO + 3	-0.10645			
HOMO-1 -> LUMO	0.42677			
HOMO-> LUMO + 2	-0.29777			
Excited States	**Singlet-A**	**Energy (eV)**	**Wavelength (nm)**	***f***
HOMO-4 -> LUMO	0.53260	4.9964	248.15	0.1068
HOMO-2 -> LUMO	-0.15024			
HOMO-1 -> LUMO	0.40948			
HOMO-3-> LUMO	0.23839	5.8566	211.70	0.1360
HOMO-2 -> LUMO + 2	-0.21845			
HOMO-1-> LUMO + 1	-0.23977			
HOMO-1 -> LUMO + 2	-0.19989			
HOMO -> LUMO + 3	0.51374			

**Table 7:** HOMO and LUMO values in gas phase and in benzene

	HOMO	LUMO	ΔE (E_LUMO_-E_HOMO_)(eV)
4PP4metB (Gas phase )	-9.532	-5.724	3.808
4PP4metB (in Benzene medium)	-9.537	-5.717	3.820
4PP4pentB (Gas phase )	-9.549	-5.780	3.767
4PP4pentB (in Benzene medium)	-9.588	-5.766	3.822
4PP4octoxB(Gas phase )	-9.199	-5.416	3.783
4PP4octoxB (in Benzene medium)	-9.201	-5.378	3.823

Uv-vis spectra have been shown in Fig. 5. The calculated electronic absorbance transitions as theoretical for 4PP4metB in gas phase, we can say that for singlet type transition having 236.94 nm value has the highest oscillator intensity of the electrons. The other singlet type transition is the HOMO-1→LUMO + 2 and HOMO→LUMO + 3 electronic transitions that occur at 211.01 nm. The electronic transition between HOMO → LUMO as a triplet transition with a wavelength of 285.12 nm and energy of 4.3485 eV. Experimentally, it is quite consistent with the long wavelength transition observed in cyclohexane, n-hexane, and n-heptane, which are compatible with the allowed transitions.

Oscillator strength (*f*) is a non-dimensional quantity that expresses the absorbance of electromagnetic radiation or the probability of emission electronic transition as a result of transitions between orbital energy levels of an atom or molecule [63–64]. Oscillator strength is the ratio between the quantum mechanical transition rate and the classical absorption/emission rate of a single electron oscillator having the same frequency as the transition [65–66].

The calculated singlet types electronic absorbance transitions as theoretical for 4PP4pentB in gas phase are 207.75 nm (*f* = 0.1244), 237.52 nm (*f* = 0.5702) and 283.64 nm (*f* = 0.1722). The calculated electronic transitions as theoretical with the electronic transitions in cyclohexane, DCM and 1-Butanol solvents are compatible.

From the calculated electronic absorbance transitions for 4PP4octoxB in gas phase have been theoretically founded as 211.70 nm (*f* = 0.1360, Singlet transition), 248.15 nm (*f* = 0.1068, Singlet transition) and 280.61 nm (*f* = 0.5371, Triplet transition). Experimentally, it is quite consistent with the short wavelength transition observed in 2-propanol and acetonitrile, which are compatible with the allowed transitions.

### MEP, HOMO and LUMO Shapes

We have calculated HOMO, LUMO and MEP in gas phase and benzene solvent medium. The energy levels and shape of the molecular orbital arrange the HOMO and the LUMO for molecules provide useful a lot of information on electronic transitions and structure. The HOMO and LUMO also indicate to areas having possible electrophilic and nucleophilic interaction in molecule, respectively. As seen in Fig. 6, green areas are positive values, when red areas are negative values for HOMO-LUMO shape. The MEP shape describes the electrostatic potential of the surface of constant electron density. MEP identifies negative and positive regions in the molecule. Red (negative) regions in the MEP are associated with electrophilic activity, while blue (positive) identify areas of nucleophilic activity [12–13].

So, in the Fig. 6, HOMO, LUMO and MEP in both gas phase and benzene medium have distributed all of 4PP4metB molecule. In the distributions of HOMO and LUMO, the red regions are the electron donor and the green regions are the electron acceptor regions. HOMO is dispersed over the entire molecule except the alkyl chain for the gas phase and benzene solvent. The LUMO in the benzene phase and the gas phase is almost the same. For 4PP4metB molecule, molecular electrostatic potential in the gas phase is 7.85 while benzene phase is 7.16. Molecular electrostatic potential value in benzene phase compare to the gas phase decreased. We can say that the negative part in the molecule is localized on benzene and alkyl group, while the positive part is concentrated on the methoxy.

Regarding 4PP4pentB in the gas phase and benzene solvent; HOMO is dispersed over the entire molecule except the alkyl chain. The LUMO benzene and the gas phase is almost the same. Electron acceptor and donor molecules are distributed in gas phase HOMO. Molecular electrostatic potential value in benzene phase compare to the gas phase increased. So, because the molecular electrostatic potential increased due to the polarity effect of benzene medium. The negative part in the molecule is localized only on the carbonyl group, while the positive part is concentrated on the benzenes and alkyl chain.

For 4PP4octoxB, HOMO is dispersed over the entire molecule for the gas and benzene phase except for the alkyl chain. The LUMO in gas and benzene phase is almost the same. HOMO in gas phase is changed electron acceptor and donor areas. Electrostatic potential in gas phase is found 7.84 a.u. while in the benzene phase is found 9.72 a.u. The molecular electrostatic potential increased due to the polarity effect of benzene. The negative part in the molecule is localized only on the carbonyl group, while the positive part is concentrated on the benzenes and alkyl chain. We can see from Fig. 6 that, HOMO and LUMO localized on molecule have changed whereas MEP has change both localization and numerically in terms of electrostatic potential depending on the substituents.

Table 7 shows the HOMO, LUMO and ΔE values of liquid crystals. When the ΔE values of 4PP4metB (methyl substituent) and 4PP4pentB (pentyl substituent) liquid crystals are compared, the value of 4PP4pentB was found to be lower than that of 4PP4metB. As the alkyl group increased, the ΔE value decreased. While there is no significant change in ΔE values when comparing 4PP4metB with 4PP4octoxB (octyloxy substituent), the ΔE value of 4PP4octoxB in the gas phase decreased by very small amounts compared to the ΔE value of 4PP4metB, and the opposite occurred in the benzene environment. When 4PP4pentB and 4PP4octoxB were compared, for the gas phase. as well as the ΔE value in the benzene phase a small decrease were occurred.

**Table 8:** The energy gap E_g_ and refractive index (n) values of 4PP4metB, 4PP4pentB and 4PP4octoxB LCs calculated by using various methods

Solvents	E_g_ (eV)	Moss	Ravindra	Herve-Vandamme	Kumar-Singh	Reddy	*n*
4PP4metB							
Methanol	4.559	2.136	1.257	1.967	2.064	2.461	1.547^a^
n-Hexane	4.578	2.134	1.245	1.963	2.061	2.458	
n-Pentane	4.595	2.132	1.235	1.960	2.059	2.456	
4PP4pentB							
Methanol	4.496	2.143	1.296	1.978	2.073	2.470	1.533^a^
n-Hexane	4.563	2.136	1.254	1.966	2.064	2.461	
n-Pentane	4.504	2.143	1.291	1.977	2.072	2.469	
4PP4octoxB							
Methanol	4.158	2.186	1.506	2.044	2.126	2.524	1.522^c^
n-Hexane	4.248	2.174	1.450	2.026	2.112	2.509	
n-Pentane	4.232	2.176	1.460	2.029	2.114	2.512	

^a^https://www.molbase.com/supplier/808518-product-16559640.html Accessed 19.12.2024

^b^https://www.echemi.com/produce/pr23120747345-4-pentylphenyl-4-octyloxybenzoate.html, Accessed 19.12.2024

### Optical Properties

We have calculated the energy gap of LC molecules by Tauc method experimentally. Thus, E_g_ values are calculated using graphs plotted experimentally (αhν)^2^ versus

$$\:E\hspace{0.17em}=\hspace{0.17em}h\nu$$.

The mass excitation coefficient (α_mass_( Lg^− 1^cm^− 1^)) describes the light absorbed by a molecule at a specific wavelength per mass density for optical applications [16].

3 $$\alpha_{mass}=\:\varepsilon\:\:/\:MA$$

where ɛ (Lmol^− 1^cm^− 1^) is the molar excitation coefficient and MA is the molar weight. The band width or band gap of the optical transition (*E*_*g*_) depends on the absorbance coefficient α_mass_ (cm^− 1^) and the energy of the photon (hν (eV)).

For optical transitions, direct or indirect inter band transition, fundamental transitions absorbance edge data can be analyzed within the framework of single electron theory. Optical gap (E_g_), optical transitions can be evaluated in the absorption spectrum using Tauc’s law [16],

4 $$ \left(ah\upsilon\right)^{n}=A^{\star}(h\upsilon-E_{g})$$

Here A* is constant. This equation constant is n; when we examine it by taking 1/2 and 2 in the equation; it gives us information about the direct and indirect transitions of the cell from the optical band gap. In the electronic absorption transition mechanism, the momentum and total energy of the electron-photon system are conserved. The refractive indices and excitation coefficients of the molecules are determined by UV-Vis. spectroscopy method. These levels are found by measuring the fundamental absorption spectrum of the refractive index levels depending on the density of the molecules. Like the type of band gap given in Eq. 4, a direct pass band gap is considered to be allowed [16].

The refractive index (n) values of the investigated compounds were determined in three different solvents, namely methanol, n-pentane and n-hexane, by using Moss, Ravindra, Hervé-Vandamme, Kumar-Singh and Reddy relations and given in Table 8. The relationship between the refractive index n and E_g_ (forbidden energy gap) is determined using the relationships Moss [17–18], Ravindra [19], Herve-Vandamme [20], Kumar Singh [21] and Reddy [22].

For semiconductors, the Moss relationship is as in Eq. 5;

5 $$n^{4} =\hspace{0.17em}95\:eV/Eg$$

The Ravindra relation is represented by a linear relation determined by the variation in E_g_ n (Eq. (6)).

6 $$\:n\hspace{0.17em}=\hspace{0.17em}4.084-0.62E_{g}$$

The Herve-Vandamme relationship is given in Eq. 7;

7 $$n^{2}=\hspace{0.17em}1+(A/(E_{g}+B\left)\right)^{2}$$

Here, A is a constant that is assumed to be the ionization energy in the ground state of the hydrogen atom and B is the difference between E_g_ and UV resonance energy.

The Kumar Singh relationship is given in Eqs. 8,

8 $$\:n\hspace{0.17em}=\hspace{0.17em}3.3668/\left(E_{g}\right)^{0.32234}$$

The Reddy relationship is given in Eq. 9.

9 $$\:n=(154/(E_{g}-0.365))^{0,5}$$

Since the investigated compounds have liquid crystal properties, forbidden band gaps were found by using Tauc’s method and refractive indices were found by five different methods. The forbidden band gaps were found from the graphs drawn against the αhν against E = hν of the studied liquid crystals in Fig. 7. Forbidden band energy and refractive index values have been listed in Table 8. Forbidden band energy intervals were found as E_g4PP4metB_> E_g4PP4pentB_> E_g4PP4octoxB_. As the alkyl chain in the compounds gets longer, the band gap energy tends to decrease. They can say that LC compounds are more reactive due to the decrease in the forbidden energy gap with the lengthening of the alkyl chain. The lower gap energy gap has found in methanol, a polar protic solvent, compared to other solvents. It can be said that the polar protic solvent reduces the band gap energy by polarizing the molecular orbitals of the studied LCs. As seen in Table 8, the band gap energy of 4pp4metB liquid crystal is around 4.4 to 4.5 eV, while 4PP4octoxB is found between 4.1 and 4.2 eV. As seen in Table 8, band gap energy decreases as the alkyl chain increases. At the same time, band gap energy is lower in 4PP4octoxB compound compared to other investigated liquid crystal compounds. Thus, since 4PP4octoxB compound has push-pull property, conjugation increases because the extension of alkyl chain and having oxygen atom of this liquid crystal compound increases the conjugation of the molecule and decreases the band gap energy [67–68]. The studied liquid crystal compounds have insulating properties according to the forbidden energy gap [69].

As seen in Table 8, according to literature research, the refractive index of the 4PP4metB compound was found to be 1.547, the refractive index of the 4PP4pentB liquid crystal was found to be 1.533 and the refractive index of the 4PP4octoxB liquid crystal was found to be 1.522, and we can say that they are compatible with the refractive index values found by the Ravindra method. The refractive indices found in the Moss, Herve-Vandamme, Kumar-Singh and Reddy relations are greater than the refractive index values in the literature, and the reason for this difference is due to the effects of the solvents on the electronic transition [70–73].

The refractive index affects the speed, color or wavelength of light in the material, temperature and optical density. In this study, the quasi-experimental refractive indices were found according to the relations of Moss, Ravindra, Herve-Vandamme, Kumar-Singh and Reddy. The refractive indices of the investigated liquid crystals were found between 1.2 and 2.5. The effective refractive index of the E7 compound used in LCD panels at room temperature has been reported as 1.5224 [71]. Thus, the refractive index of the compounds in our study was found to be higher than the refractive index of the E7 compound, except for the Ravindra method.

## Conclusions

The absorbance and fluorescence transitions of 4-Pentylphenyl 4-*n*-benzoate derivatives were investigated in the solvent environment with different polarity, and its electronic transitions were interpreted. The bathochromic effect is more dominant in the absorption and fluorescence electronic transitions. These bathochromic shifts correspond to more stabilize excited state as compared to the ground state. During the fluorescence transition of the compounds, they interact strongly through intramolecular charge transfer. As seen from derived LSERs models for determined as quantitative to solute-solvent interactions if C coefficients are negative, as depending on solvent in the absorption and fluorescence electronic transition produce bathochromic effects. The bathochromic effect means that the wavelength shifts to larger wavelengths with the solvent effect, that is, the electronic transition energy decreases, while the hypsochromic effect means that the solvent effect shifts to smaller wavelengths, that is, the electronic transition energy is high. These bathochromic shifts correspond to more stabilize excited state as compared to the ground state. It has been found that the refractive indices of LCs tend to increase as the length of the alkyl chain increases. The forbidden energy gap of the liquid crystal compounds was found between 4 and 4.5 eV, and it may think that the compounds can as insulators. Theoretically calculated electronic transitions were found to be compatible with experimental results. Since the electronic structure and transitions change depending on the solvent, the investigated liquid crystals are solvatochromic materials. Due they have the ability to easily manipulate light and multiple different wavelength transitions in the absorbance and fluorescence spectrum, they have the potential to be usable in optoelectronic devices.

## Electronic Supplementary Material

Below is the link to the electronic supplementary material.

Supplementary Material 1

## Data Availability

No datasets were generated or analysed during the current study.

## References

[1] Villanueva-García M, Gutiérrez-Parra RN, Martínez-Richa A, Juvencio R (2005) Quantitative structure-property relationships to estimate nematic transition temperatures in thermotropic liquid crystals. J Mol Struct (THEOCHEM) 727:63–69. 10.1016/j.theochem.2005.02.033

[2] Andrienko D (2018) Introduction to liquid crystals. J Mol Liq 267:520–541. 10.1016/j.molliq.2018.01.175

[3] Singh S (2005) Phase transitions in liquid crystals. Phys Rep 324:107–269. 10.1016/S0370-1573(99)00049-6

[4] Ojha PD, Pisipati VGKM (2002) Molecular ordering of a cyano compound at a displacive transition temperature: a statistical analysis based on quantum mechanics and computer simulations. Liq Cryst 29(7):979–984. 10.1080/02678290210145184

[5] Belyaev BA, Drokin NA, Shabanov VF, Baranova VA (2004) Dielectric properties of liquid crystals of the cyano derivative compounds with different fragments in the molecular core. Phys Sol Stat 46(3):574–578. 10.1134/1.1687881

[6] Eikelschulte F, Yakovenko SY, Paschek D, Geiger A (2000) Electrostatic properties of cyano-containing mesogens. Liq Cryst 27(9):1137–1146. 10.1080/02678290050121971

[7] Richardson PR, Bates SP, Crain J, Jones AC (2000) Structure and properties of isolated liquid crystal molecules: jet spectroscopy and ab initio calculations of 4-cyanobiphenyl liq cryst. 27(6):845–850. 10.1080/026782900202345

[8] Dominguez H, Velasco E, Alejandre J (2022) Stress anisotropy in liquid crystalline phases. Mol Phys 100(16):2739–2744. 10.1080/00268970210132513

[9] Matsushima J et al (2002) Transition moment orientation and rotational bias of three carbonyl groups in large polarization FLCs observed by polarized FTIR. Liq Cryst 29(1):27–37. 10.1080/02678290110039525

[10] Gennes de PG, Prost J (1993) The physics of Liquid crystals. Clarendon, Oxford

[11] Das P, Praveen PL (2019) Solvent polarity and chain length effects in thermotropic mesophase formation process: comparative quantum and thermodynamic analysis. J Mol Liq 288:111029. 10.1016/j.molliq.2019.111029

[12] Pirbudak Altıntaş G, Gülseven Sıdır Y (2024) Solvatochromism, electric dipole moments, optical properties and electronic structure of biphenylcarbonitrile liquid crystals. Opt Mat 157:116342. 10.1016/j.optmat.2024.116342

[13] Pirbudak Altıntaş G, Gülseven Sıdır Y, Sıdır İ (2025) Determination on optical properties, dipole moments, solvent effect on electronic transitions and global activity parameters of liquid crystals of 4’-methyl-2-biphenylcarbonitrile and 4′-hydroxy-4-biphenylcarbonitrile. J Mol Liq 420:126795. 10.1016/j.molliq.2024.126795

[14] Lin YC, Li GS, Yu PJ, Ercan E (2022) Wen-Chang Chen Organic liquid crystals in optoelectronic device applications: field-effect transistors, nonvolatile memory, and photovoltaics. J Chin Chem Soc 69(8):1289–1304. 10.1002/jccs.20220006

[15] Shi L, Yan W (2023) Investigation of optoelectronic functional crystals: Crystal Growth, Properties and Applications. Materials 16:6871. 10.3390/ma16216871 37959468 10.3390/ma16216871 PMC10649129

[16] Tauc J (1974) Amorphous and Liquid Semiconductors. Plenum, New York

[17] Moss TS (1985) Relations between the Refractive Index and Energy Gap of Semiconductors. Phy Sta Sol B 131:415–427. 10.1002/pssb.2221310202

[18] Moss TS (1950) A relationship between the refractive index and the infra-red threshold of sensitivity for photoconductors. Proc Phys Soc Sec B 63(3):167–176. 10.1088/0370-1301/63/3/302

[19] Ravindra NM, Auluck S, Srivastava VK (1979) On the Penn gap in Semiconductors. Phys St Sol B 93(2):155–160. 10.1002/pssb.2220930257

[20] Hervé P, Vandamme LKJ (1994) General relation between refractive index and energy gap in semiconductors. Infr Phys Tech 35(4):609–615. 10.1016/1350-4495(94)90026-4

[21] Kumar V, Singh JK (2010) Model for calculating the refractive index of different materials. Ind J Pure and App Phy 48(8): 571–574. doi:https://doi.org/handle/123456789/9962

[22] Reddy RR, Anjaneyulu S (1992) Analysis of the Moss and Ravindra relations. Phy Sta Sol B 174(2):91–93. 10.1002/pssb.2221740238

[23] Valeur B, Berberan-Santos M (2012) Characteristics of fluorescence emission. In: Molecular fluorescence, 2nd edn. Wiley, Hoboken. 10.1002/9783527650002

[24] Bertolotti M, Sansoni G, Scudieri F (1979) Dye laser emission in liquid crystal hosts. Appl Opt 18:528–531. https://opg.optica.org/ao/abstract.cfm?URI=ao-18-4-528 20208756 10.1364/AO.18.000528

[25] Mysliwiec J, Szukalska A, Szukalski A, Sznitko L (2021) Liquid crystal lasers: the last decade and the future. Nanophotonics 2021(10):2309–2346. 10.1515/nanoph-2021-0096

[26] Grell M, Bradley DDC, Inbasekaran M, Woo EP (1997) A glass-forming conjugated main-chain liquid crystal polymer for polarized electroluminescence applications. Adv Mater 1997, 9: 798–802. 10.1002/adma.19970091006

[27] O’Neill M, Kelly S (2003) Liquid crystals for charge transport. Luminescence Photonics Adv Mater 15:1135–1146. 10.1002/adma.200300009

[28] De J, Abdul Haseeb MM, Yadav Rak, Gupta SP, Bala I, Chawla P, Kesavan KK, Jou JH, Pal SK (2020) AIE-active mechanoluminescent discotic liquid crystals for applications in OLEDs and bio-imaging. Chem Commun 56:14279–14282. 10.1039/D0CC05813K 10.1039/d0cc05813k 33125010

[29] Bobrovsky A, Shibaev V, Hamplová V, Novotna V, Kašpar M (2015) Photochromic and fluorescent LC gels based on a bent-shaped azobenzene-containing gelator. RSC Adv 5:56891–56895. 10.1039/C5RA07234D

[30] Zhang L, Cui Y, Wang Q, Zhou H, Wang H, Li Y, Yang Z, Cao H, Wang D, He W (2022) Spatial patterning of fluorescent liquid crystal ink based on Inkjet Printing. Molecules 27:5536. 10.3390/molecules27175536 36080303 10.3390/molecules27175536 PMC9458137

[31] Tong X, Zhao Y, An BK, Park SY (2006) Fluorescent liquid-crystal gels with electrically switchable photoluminescence. Adv Funct Mater 16:1799–1804. 10.1002/adfm.200500868

[32] Bobrovsky A, Shibaev V, Hamplová V, Novotna V, Kašpar M (2015) Photochromic and fluorescent LC gels based on a bent-shaped azobenzene-containing gelator. RSC Adv 5:56891–56895. 10.1039/c5ra07234d

[33] Sergeyev S, Pisula W, Geerts YH (2007) Discotic liquid crystals: a new generation of organic semiconductors. Chem Soc Rev 36:1902–1929. 10.1039/B417320C 17982517 10.1039/b417320c

[34] Gülseven Sıdır Y, Sıdır İ (2015) Solvatochromic fluorescence of 4-alkoxybenzoic acid liquid crystals: Ground and excited state dipole moments of monomer and dimer structures determined by solvatochromic shift methods. J Mol Liq 211:591–603. 10.1016/j.molliq.2015.07.053

[35] Gülseven Sıdır Y, Sıdır İ, Demiray F (2017) Dipole moment and solvatochromism of benzoic acid liquid crystals: tuning the dipole moment and molecular orbital energies by substituted au under external electric field. J Mol Struct 1137:440–452. 10.1016/j.molstruc.2017.02.055

[36] Gülseven Sıdır Y, Sıdır İ, Berber H (2022) Optoelectronic properties by solution technique and comprehensive solvatochromism of novel fluorescent Schiff base derivatives. J Mol Liq 357:119110. 10.1016/j.molliq.2022.119110

[37] Origin(Pro) Version Version 2008 OriginLab Corporation, Northampton,MA, USA

[38] Menges F Spectragryph - optical spectroscopy software, Version 1.x.x, 202x., http://www.effemm2.de/spectragryph/

[39] Koppel IA et al (1972) The infuence of the solvent on organic reactivity. In: Chapman NB, Shorter JS (eds) Advances in Linear Free Energy relationships. Plenum, London

[40] Becke AD (1993) Density-functional thermochemistry. III. The role of exact exchange. J Chem Phys 98:5648–5652. 10.1063/1.464913

[41] Chengteh L, Yang W, Parr RG (1988) Development of the Colle-Salvetti correlation-energy formula into a functional of the electron density. Phy Rev B 37:785. 10.1103/PhysRevB.37.785 10.1103/physrevb.37.785 9944570

[42] Srivastava R et al (2017) A combined experimental and theoretical DFT (B3LYP, CAM-B3LYP and M06-2X) study on electronic structure, hydrogen bonding, solvent effects and spectral features of methyl 1H-indol-5-carboxylate. J Mol Struct 1137:725–741. 10.1016/j.molstruc.2017.02.084

[43] Plumley JA, Dannenberg JJ (2011) A comparison of the behavior of Functional/Basis set combinations for hydrogen-bonding in the water dimer with emphasis on basis set Superposition Error. J Comput Chem 32(8):1519–1527. 10.1002/jcc.21729 21328398 10.1002/jcc.21729 PMC3073166

[44] Krishnan R, Binkley JS, Seeger R, Pople JA (1980) Self-consistent molecular orbital methods. XX. A basis set for correlated wave functions. J Chem Phy 72:650–654. 10.1063/1.438955

[45] Trani F, Barone V (2011) Silicon Nanocrystal Functionalization: Analytic Fitting of DFTB parameters. J Chem Com 7(3):713–719. 10.1021/ct1006086 10.1021/ct1006086 26596303

[46] Hamada NMM (2018) Synthesis, Spectroscopic characterization, and TimeDependent DFT calculations of 1-Methyl-5-phenyl-5Hpyrido[1,2-a]quinazoline-3,6-dione and its starting precursor in different solvents. ChemistryOpen 7:814–823. 10.1002/open.201800146 30338205 10.1002/open.201800146 PMC6182252

[47] Hussain A et al (2020) Structural parameters, electronic, linear and nonlinear optical exploration of thiopyrimidine derivatives: a comparison between DFT/TDDFT and experimental study. J Mol Struct 1201:127183. 10.1016/j.molstruc.2019.127183

[48] Scalmani G, Frisch MJ (2010) Continuous surface charge polarizable continuum models of solvation. I. General formalism. J Chem Phys 132:114110. 10.1063/1.3359469 20331284 10.1063/1.3359469

[49] Tomasi J, Mennucci B, Cammi R (2005) Quantum mechanical continuum solvation models. Chem Rev 105:2999–3093. 10.1021/cr9904009 16092826 10.1021/cr9904009

[50] Frisch MJ (2009) Gaussian 09 User’s Gaussian, Incorporated

[51] Frisch MJ et al (2009) Gaussian 09, Revision A.02; Gaussian, Inc.: Wallingford, CT, USA

[52] Nielsen AB et al (2009) Gauss View 5.0, user’s reference. GAUSSIAN Inc., Pittsburgh

[53] Pirbudak Altıntaş G, Gülseven Sıdır Y (2024) Solvatochromism, electric dipole moments, optical properties and electronic structure of biphenylcarbonitrile liquid crystals. Opt Mat 15:116342. 10.1016/j.optmat.2024.116342

[54] Gülseven Sıdır Y (2020) The solvatochromism, electronic structure, electric dipole moments and DFT calculations of benzoic acid liquid crystals. Liq Cryst 47(10):1435–1451. 10.1080/02678292.2020.1733685

[55] Reichardt C, Welton T (2011) Solvents and Solvent efects in Organic Chemistry. Wiley, New York

[56] Kamlet MJ, Taft RW (1982) Linear solvation energy relationships. 20. Intra-vs. Intermolecular hydrogen bonding by some 2-nitroaniline and 2-nitrophenol derivatives. J Org Chem 47(9):1734–1738. 10.1021/jo00348a027

[57] Kamlet MJ, Abboud JLM, Abraham MH, Taft RW (1983) Linear solvation energy relationships. 23. A comprehensive collection of the solvatochromic parameters,.Pi.*,. alpha., and. beta., and some methods for simplifying the generalized solvatochromic equation. J Org Chem 48:2877–2887. 10.1021/jo00165a018

[58] Kamlet MJ, Abboud JLM, Taft R (1997) The solvatochromic comparison method. 6. The.pi.* scale of solvent polarities. J Amer Chem Soc 99(18):6027–6038. 10.1021/ja00460a031

[59] Kamlet MJ, Abboud JLM, Taft RW (1981) An examination of Linear Solvation Energy Relationships., Editor(s): R. W Taft. Prog Phy Org Chem 13:485. 10.1002/9780470171929.ch6

[60] Catalán J Toward a generalized treatment of the Solvent Effect based on four empirical scales: Dipolarity (SdP, a New Scale), polarizability (SP), acidity (SA), and basicity (SB) of the medium. J Phys Chem B 113 (17):5951–5960. 10.1021/jp8095727 10.1021/jp8095727 19344103

[61] Gülseven Sıdır Y, Aslan C, Berber H, Sıdır İ (2019) The electronic structure, solvatochromism, and electric dipole moments of new Schiff base derivatives using absorbance and fluorescence spectra. Struct Chem 30:835–851. 10.1007/s11224-018-1228-8

[62] Gülseven Sıdır Y, Berber H, Sıdır İ (2019) The dipole moments and solvatochromism of ((4-(Benzyloxy)benzylidene)amino)phenol compounds as Solvatochromic materials. J Sol Chem 48:775–806. 10.1007/s10953-019-00885-z

[63] Kara YE, Gülseven Sıdır Y, Sıdır İ, Kandemirli F (2023) Solvatochromism and optoelectronic properties of thiosemicarbazone derivatives having π-conjugated systemsJ. Sol Chem 52:570–587. 10.1007/s10953-023-01248-5

[64] SPSS Statistics for Windows, version 15.0, SPSS Inc., Chicago, III, USA

[65] Demtröder W (2003) Laser Spectroscopy: Basic Concepts and Instrumentation. Springer. p. 31. ISBN 978-3-540-65225-0

[66] Robinson JW (1996) Atomic Spectroscopy Marcel Dekker Incorporated. ISBN 978-0-8247-9742-3

[67] Westermayr J, Marquetand P (2021) Machine learning for electronically Excited States of. Molecules Chem Rev 121(16):9873–9926. 10.1021/acs.chemrev.0c00749 33211478 10.1021/acs.chemrev.0c00749 PMC8391943

[68] Hilborn RC (1982) Einstein coefficients, cross sections, f values, dipole moments, and all that Amer. J Phys 50(11):982–986. 10.1119/1.12937

[69] Gnida P (2024) Unexpected impact of N-Alkyl chain length in Bis-2-cyanoacrylic acid substituted phenothiazines on the Photovoltaic response of DSSCs. Ind Eng Chem 63(16):7133–7153. 10.1021/acs.iecr.4c00045

[70] Li M, Leenaers PJ, Wienkb MM, Janssen RAJ (2020) The effect of alkyl side chain length on the formation of two semi-crystalline phases in low band gap conjugated polymers. J Mater Chem C 8:5856–5867. 10.1039/D0TC00172D

[71] Book (2024) Introduction to Inorganic Chemistry, LibreText™ Chemistry

[72] Renge I (2018) Refractive index dependence of solvatochromism. J Photochem Photobio A: Chem 353:433–444. 10.1016/j.jphotochem.2017.11.048

[73] Li J, Wen CH, Gauza S, Lu R, Wu ST (2005) Refractive indices of liquid crystals for Display Applications. Ieee/Osa J Dısplay Tech 1:51–61. 10.1109/JDT.2005.853357

