# Supplementary material for: The Optical Properties, UV-Vis. Absorption and Fluorescence Spectra of 4-Pentylphenyl 4-n-benzoate Derivatives in Different Solvents
Source: J Fluoresc. 2025 Feb 8;35(9):8269–87. doi: 10.1007/s10895-025-04154-9 (PMC12583416; doi:10.1007/s10895-025-04154-9)
Supplement: Supplementary file 1 — Supplementary Material 1 [file 10895_2025_4154_MOESM1_ESM.docx]

**SUPPLEMENTARY MATERIALS**

**The optical properties, UV-Vis. absorption and fluorescence spectra of 4-Pentylphenyl 4-*n*-benzoate derivatives in different solvents**

**Yadigar GÜLSEVEN SIDIR^[[1]](#footnote-1)^, İsa SIDIR**

Bitlis Eren University, Faculty of Arts and Science, Department of Physics, 13000, Bitlis, Türkiye.

**Figure 1S.** The absorption spectra of LCs in1,4-Dioxane solvent…………………………….2

**Figure 2S.** The correlations of ν_cal_, *β, α, f(n)* and *f(ε)* versus ν_exp_  of λ_ABS1_ wavelength of 4PP4metB molecule……………………………………………………………………………3

**Figure 3S.** The correlations of νcal, SP, SdP, SA and SB versus νexp of λ_ABS1_ wavelength of 4PP4metB molecule……………………………………………………………………………4

**Figure 4S.** The correlations of νcal, β, α, f(n) and f(ε) versus νexp of λ_PL1_ wavelength of 4PP4metB molecule……………………………………………………………………………5

**Figure 5S.** The correlations of νcal, SP, SdP, SA and SB versus νexp of λ_PL1_ wavelength of 4PP4metB molecule……………………………………………………………………………6

**Figure 6S.** The correlations of νcal, β, α, f(n) and f(ε) versus νexp of λ_PL2_ wavelength of 4PP4metB molecule…………………………………………………………………………....7

**Figure 7S.** The correlations of *ν_cal_, SP, SdP, SA* and *SB* versus ν_exp_  of λ_PL2_ wavelength of 4PP4metB molecule……………………………………………………………………………8

**Figure 8S.** The correlations of *ν_cal,_ β, α, f(n)* and *f(ε)* versus ν_exp_ of λ_PL3_ wavelength of 4PP4metB molecule…………………………………………………………………………....9

**Figure 9S.** The correlations of *ν_cal_, SP, SdP, SA* and *SB* versus ν_exp_ of λ_PL3_ wavelength of 4PP4metB molecule…………………………………………………………………………..10

**Figure 10S.** The correlations of *ν_cal_, β,α, f(n)* and *f(ε)* versus ν_exp_  of λ_ABS1_ wavelength of 4PP4pentB molecule……………………………………………………………………….…11

**Figure 11S.** The correlations of *ν_cal_, SP, SdP, SA* and *SB* versus ν_exp_ of λ_ABS1_ wavelength of 4PP4pentB molecule……………………………………………………………………….…12

**Figure 12S.** The correlations of *ν_cal_, β, α, f(n)* and *f(ε)* versus ν_exp_ of λ_PL1_ wavelength of 4PP4pentB molecule……………………………………………………………………….…13

**Figure 13S.** The correlations of νcal, SP, SdP, SA and SB versus νexp of λ_PL1_ wavelength of 4PP4pentB molecule………………………………………………………………………….14

**Figure 14S.** The correlations of *ν_cal,_ β, α, f(n) and f(ε)* versus ν_exp_ of λ_PL2_ wavelength of 4PP4pentB molecule…………………………………………………………………….……15

**Figure 15S.** The correlations of νcal, SP, SdP, SA and SB versus νexp of λ_PL2_ wavelength of 4PP4pentB molecule………………………………………………………………………16

**Figure 16S.** The correlations of *ν_cal,_ β, α, f(n)* and *f(ε)* versus ν_exp_  of λ_ABS1_ wavelength of 4PPoctoxBB molecule………………………………………………………………………..17

**Figure 17S.** The correlations of νcal, SP, SdP, SA and SB versus νexp of λ_ABS1_ wavelength of 4PP4pentB molecule………………………………………………………………….……18

**Figure 18S.** The correlations of *ν_cal_, β, α, f(n)* and *f(ε)* versus ν_exp_ of λ_PL1_ wavelength of 4PPoctoxBB molecule………………………………………………………………………..19

**Figure 19S.** The correlations of *ν_cal_, SP, SdP, SA* and *SB* versus ν_exp_ of λ_PL1_ wavelength of 4PPoctoxBB molecule…………………………………………………………………..……20

**Figure 20S.** The correlations of *ν_cal_, β, α, f(n)* and *f(ε)* versus ν_exp_ of λ_PL2_ wavelength of 4PPoctoxBB molecule………………………………………………………………………..21

**Figure 21S.** The correlations of *ν_cal_, SP, SdP, SA* and *SB* versus ν_exp_ of λ_PL2_ wavelength of 4PPoctoxBB molecule……………………………………………………………………..…22

**Figure 22S.** The correlations of *ν_cal_, β, α, f(n)* and *f(ε)* versus ν_exp_ of λ_PL3_ wavelength of 4PPoctoxBB molecule………………………………………………………………………..23

**Figure 23S.** The correlations of *ν_cal_, SP, SdP, SA* and *SB* versus ν_exp_  of λ_PL3_ wavelength of 4PPoctoxB molecule………………………………………………………………………….24

**Figure 24S.** The correlations of *ν_cal_, β, α, f(n)* and *f(ε)* versus ν_exp_  of λ_PL4_ wavelength of 4PPoctoxB molecule………………………………………………………………………….25

**Figure 25S.** The correlations of *ν_cal_, SP, SdP, SA* and *SB* versus νexp of λ_PL4_ wavelength of 4PPoctoxB molecule……………………………………………………………………….....26

**Figure 26S.** The correlations of *ν_cal_, β, α, f(n)* and *f(ε)* versus ν_exp_ of λ_PL5_ wavelength of 4PPoctoxB molecule………………………………………………………………………….27

**Figure 27S.** The correlations of *ν_cal,_ SP, SdP, SA* and *SB* versus ν_exp_ of λ_PL5_ wavelength of 4PPoctoxB molecule………………………………………………………………………….28

**Table 1S.** The used parameters values in LSERs calculation……………………………..…29

**
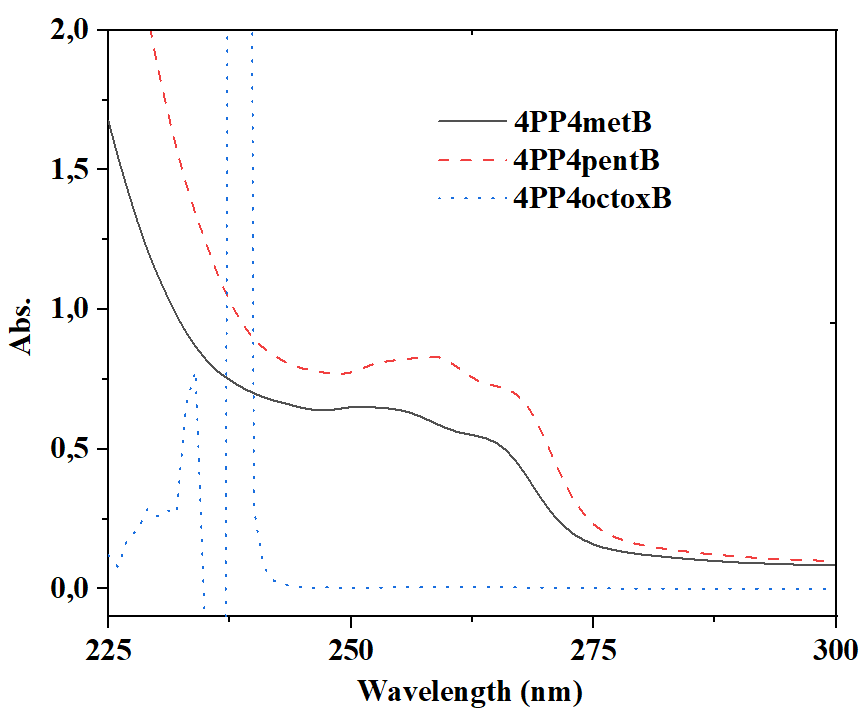
**

**Figure 1S.** The absorption spectra of LCs in1,4-Dioxane solvent.


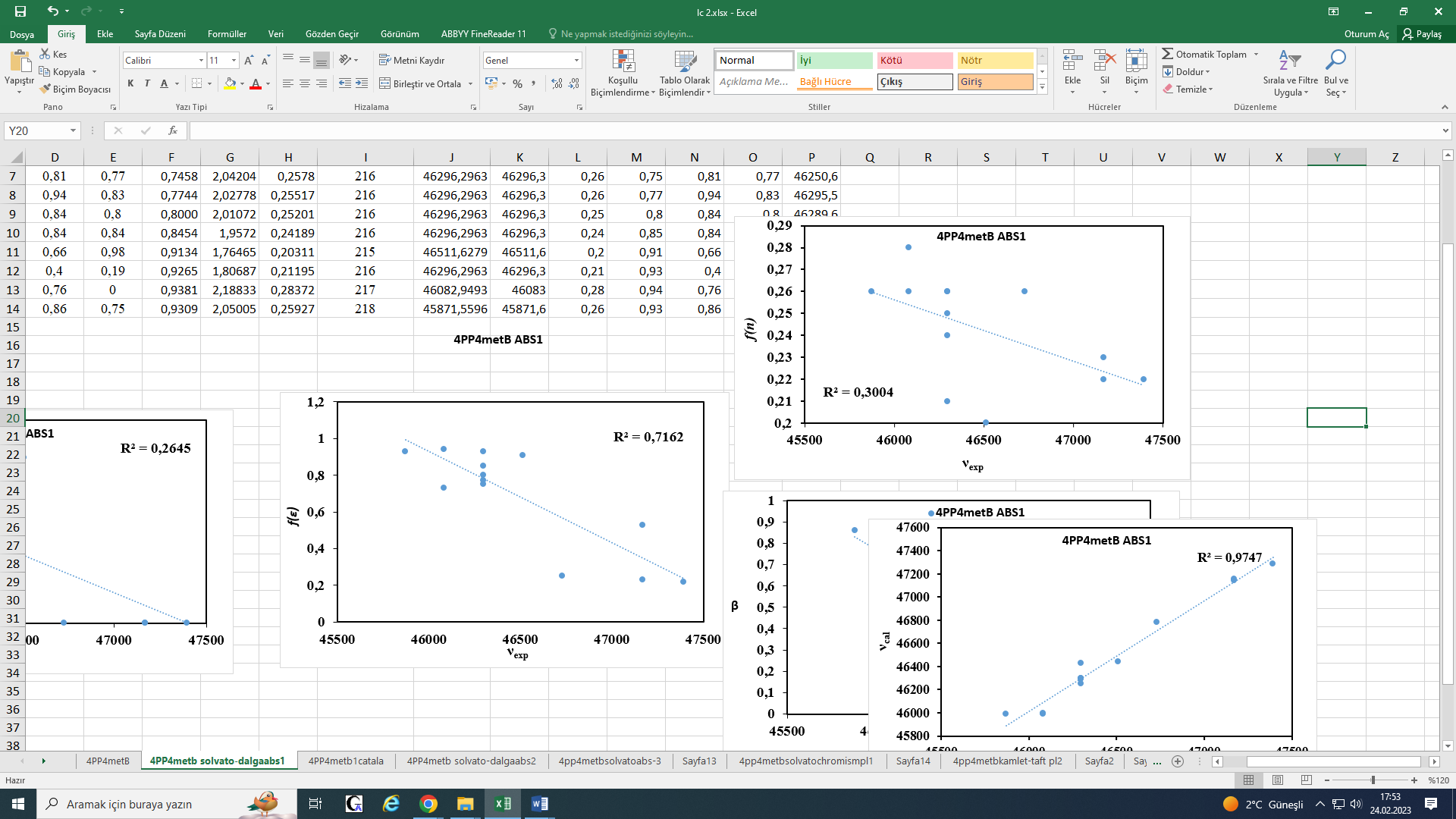

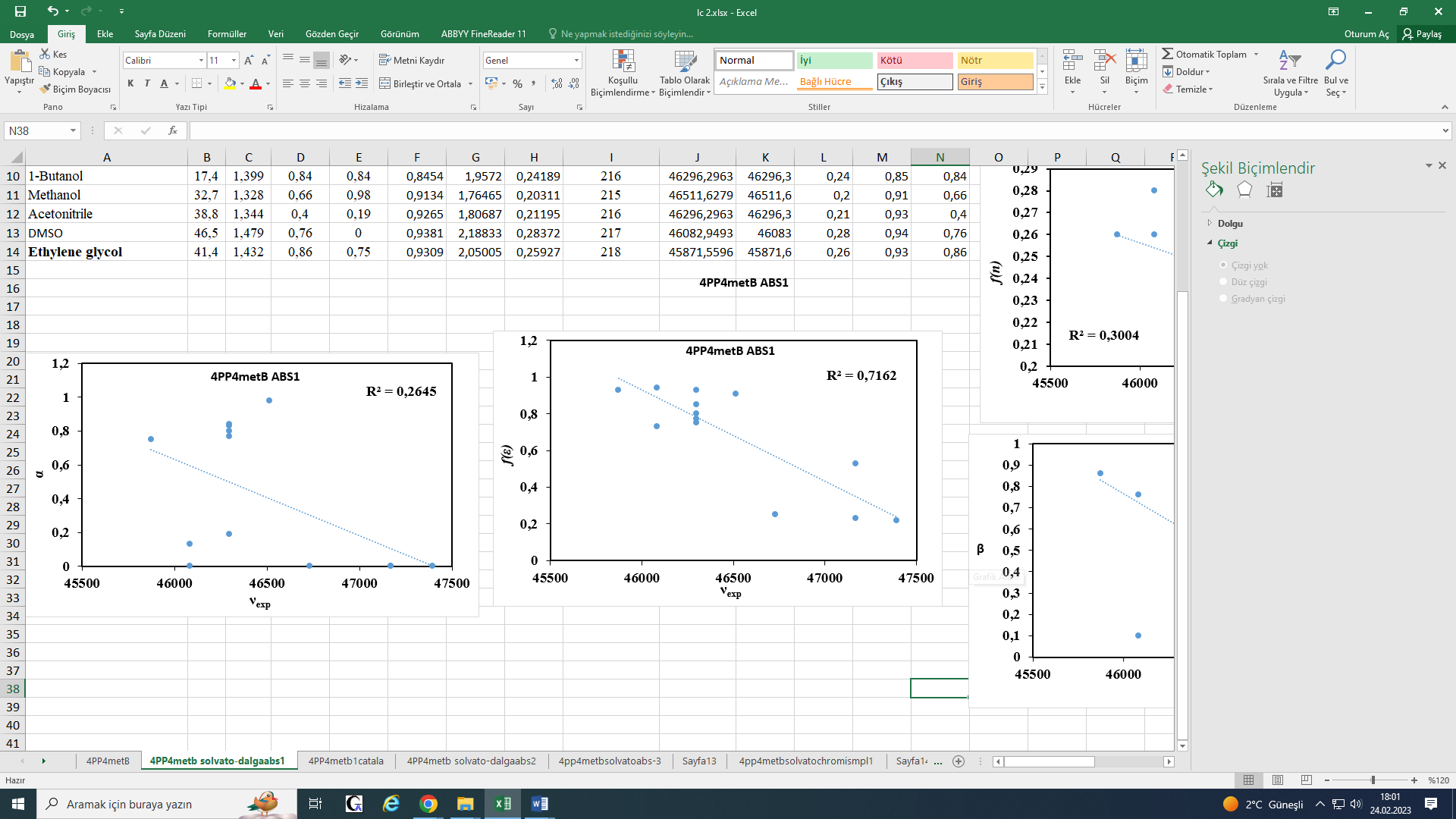

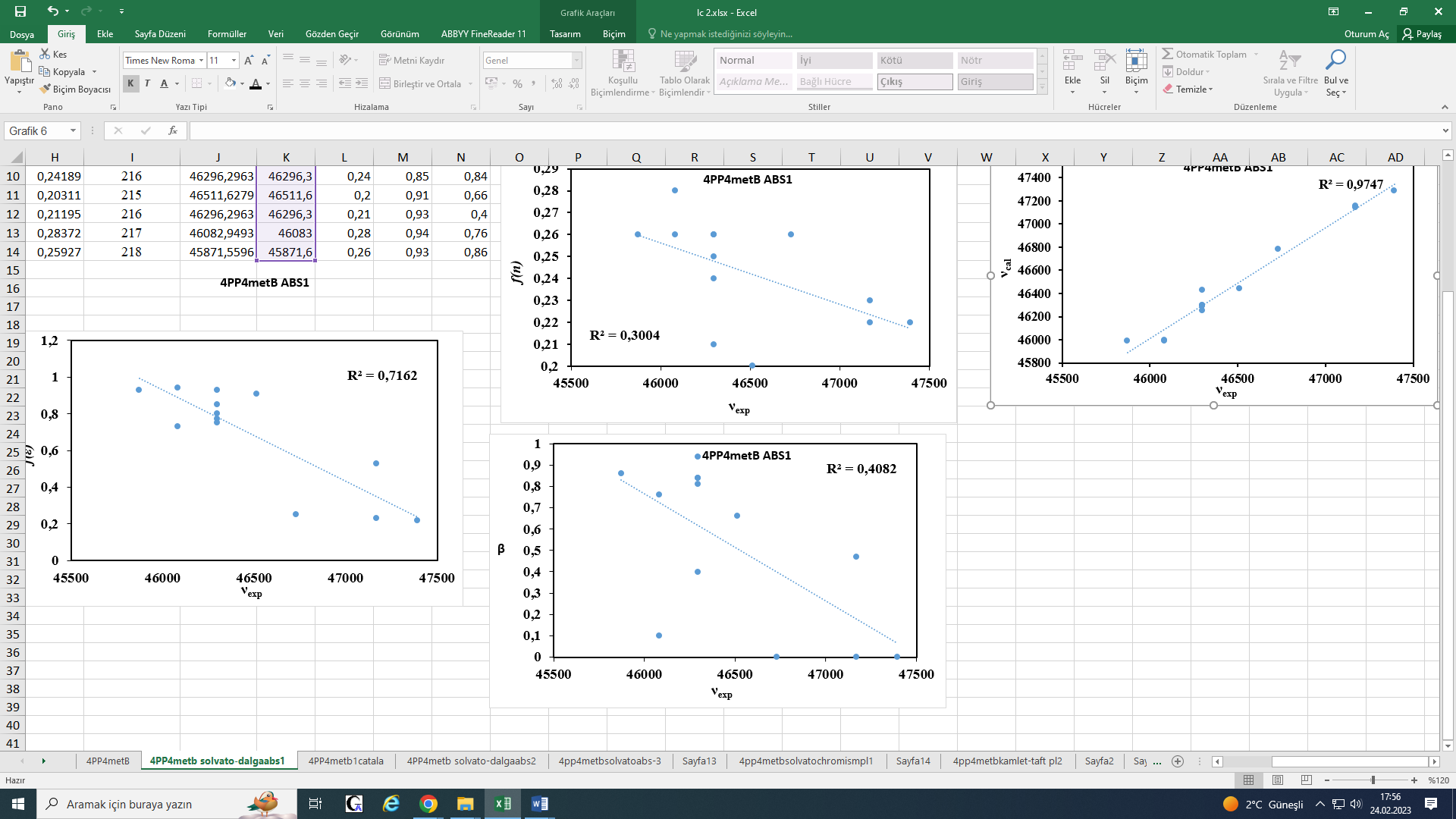

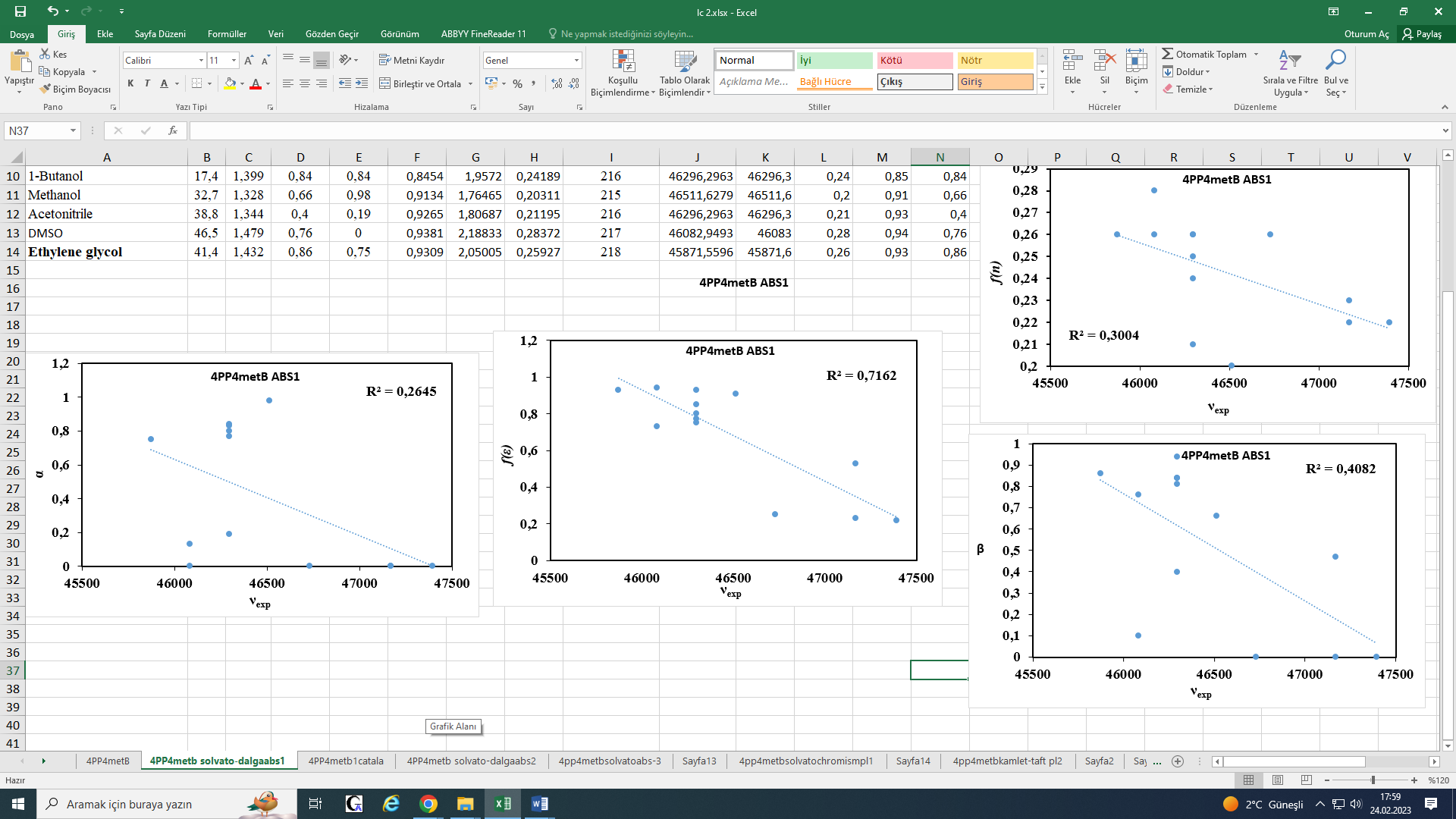

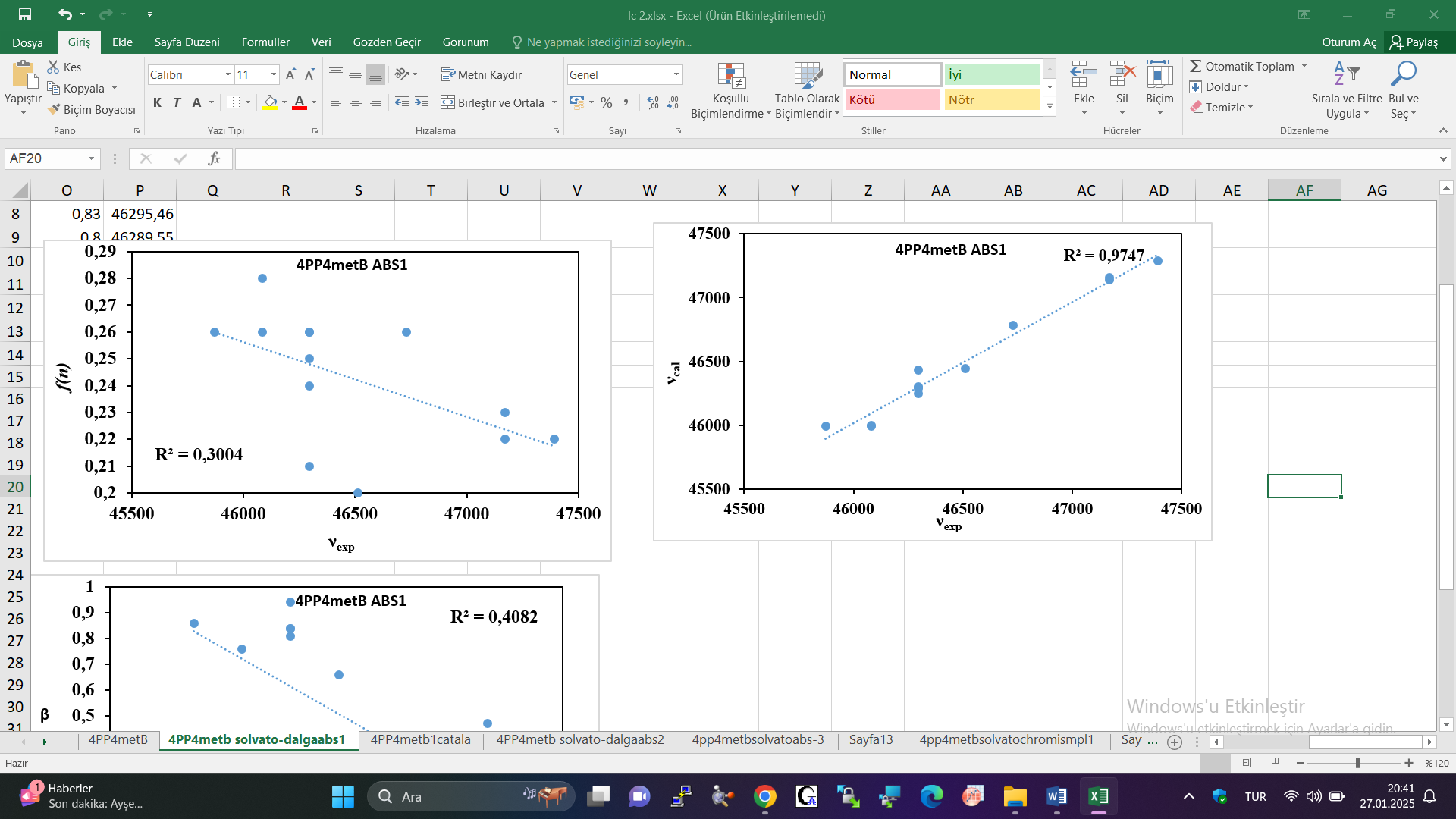


**Figure 2S.** The correlations of ν_cal_, *β, α, f(n)* and *f(ε)* versus ν_exp_  of λ_ABS1_ wavelength of 4PP4metB molecule.


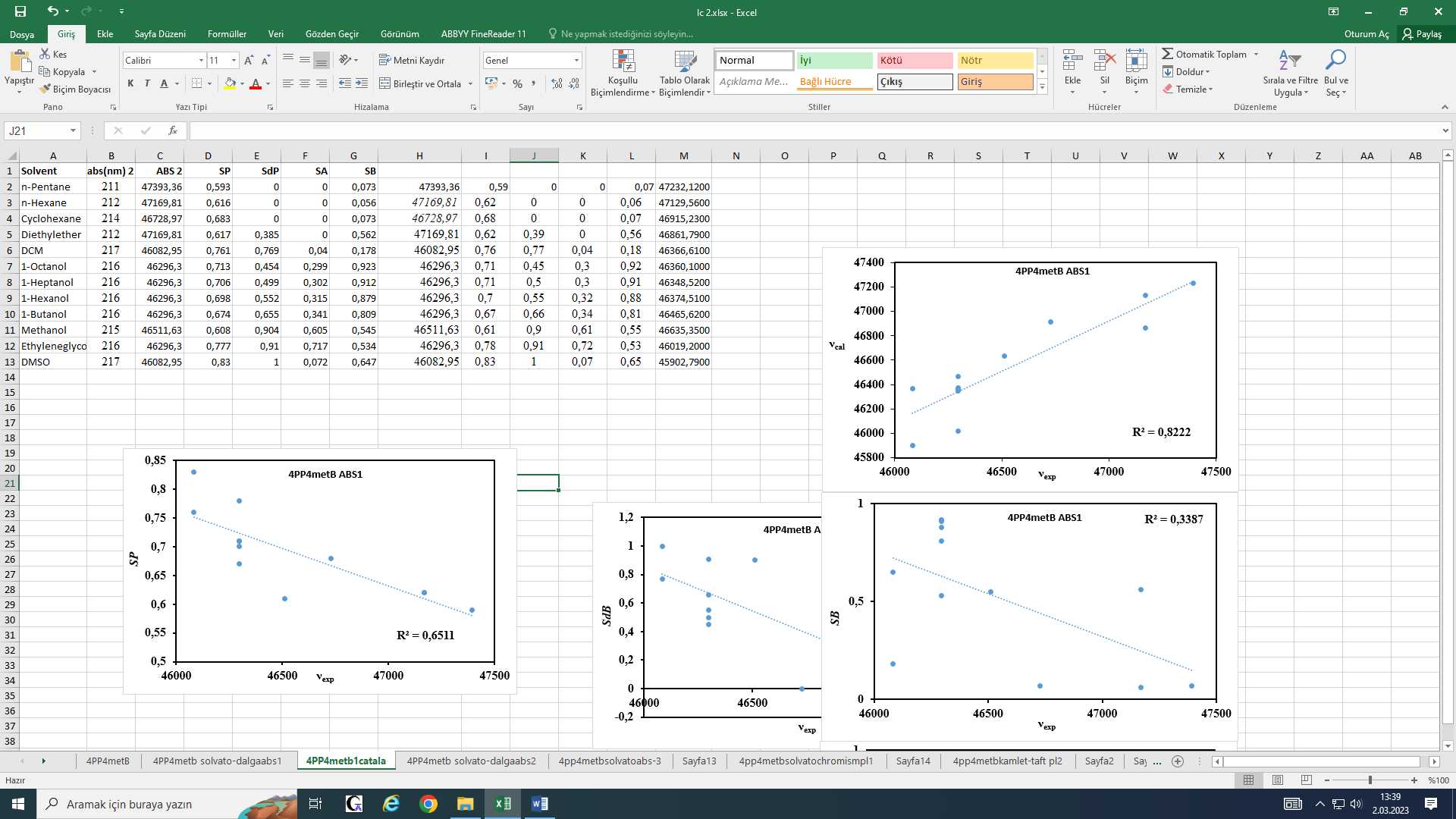

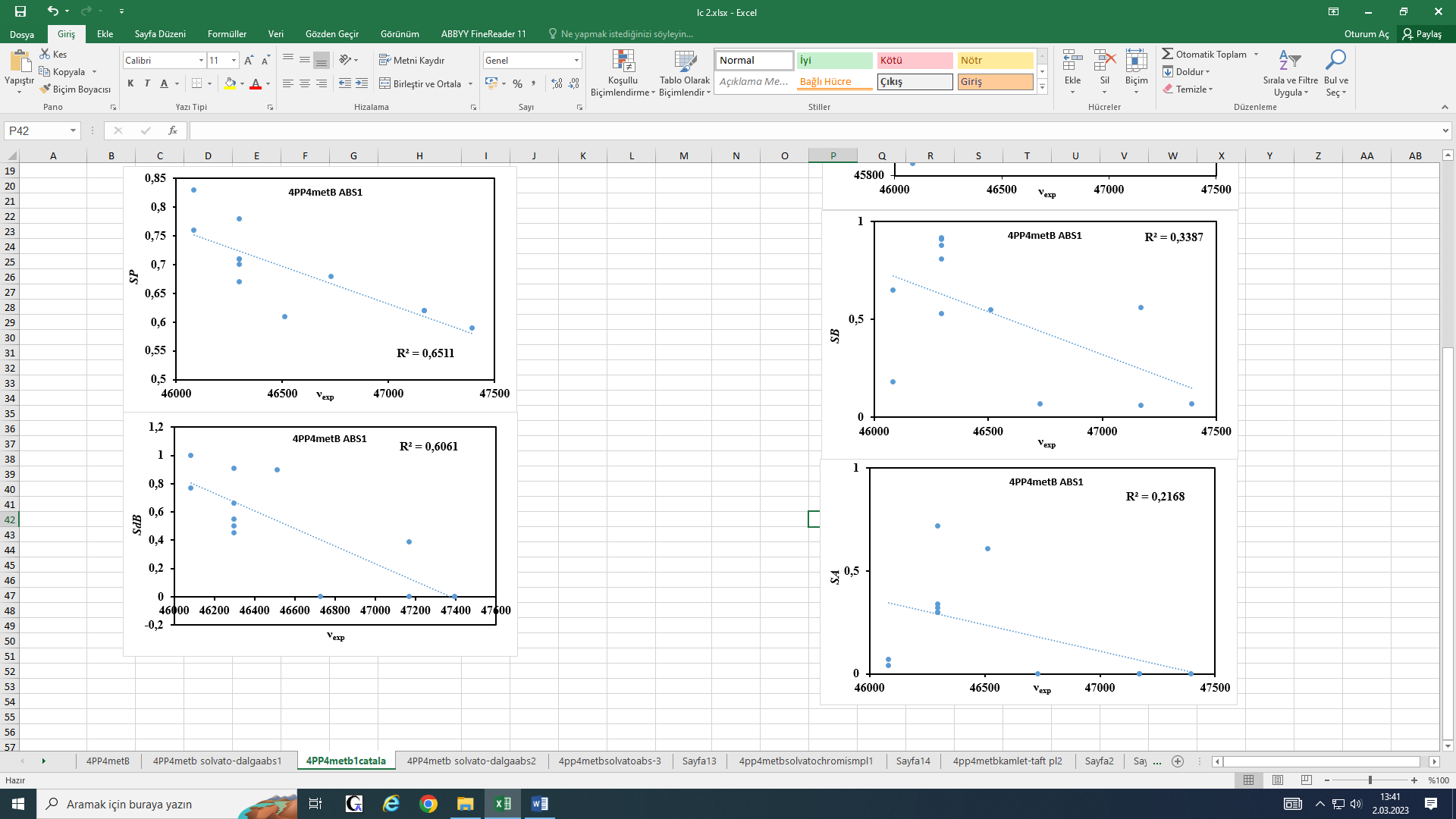

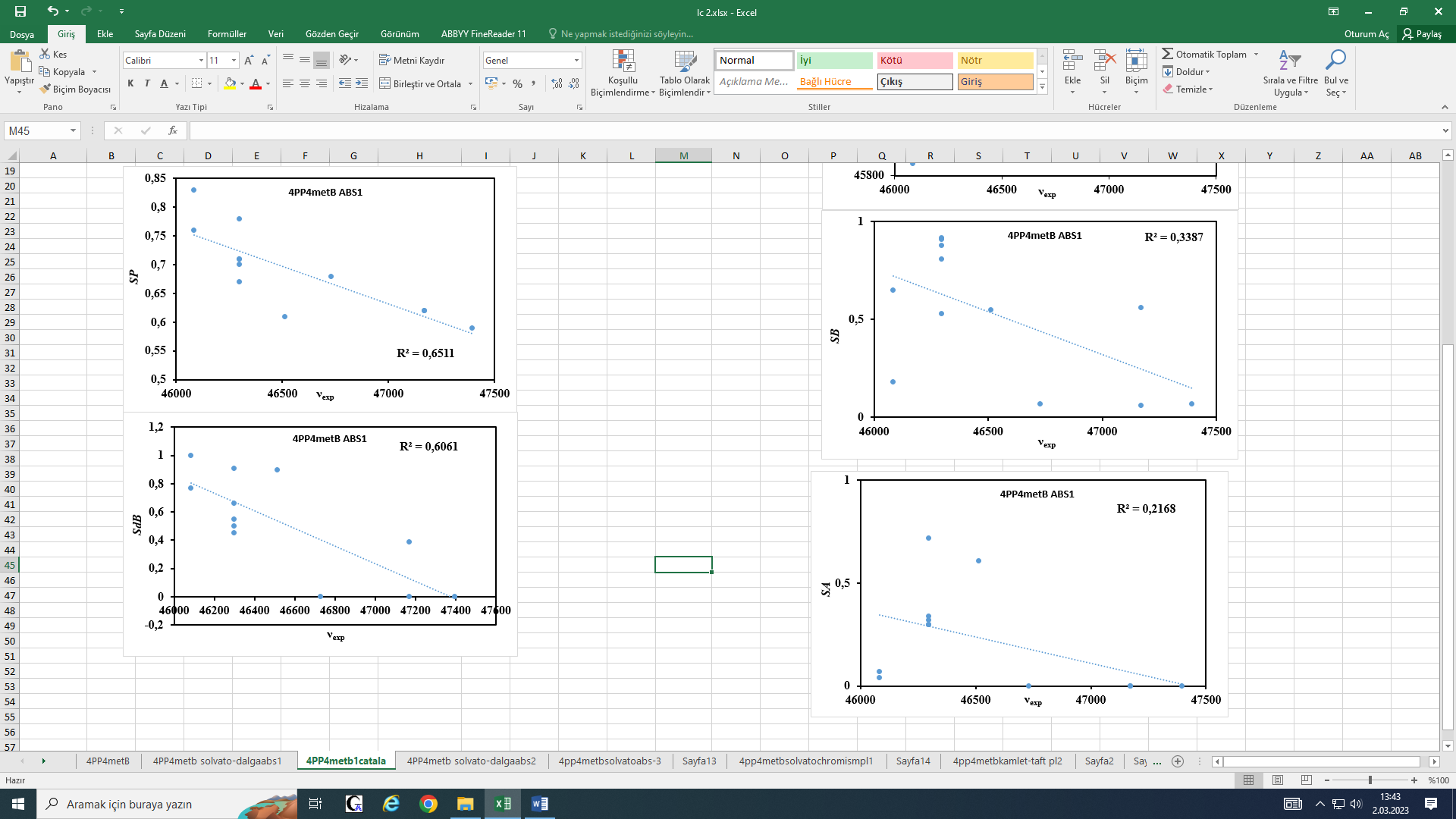

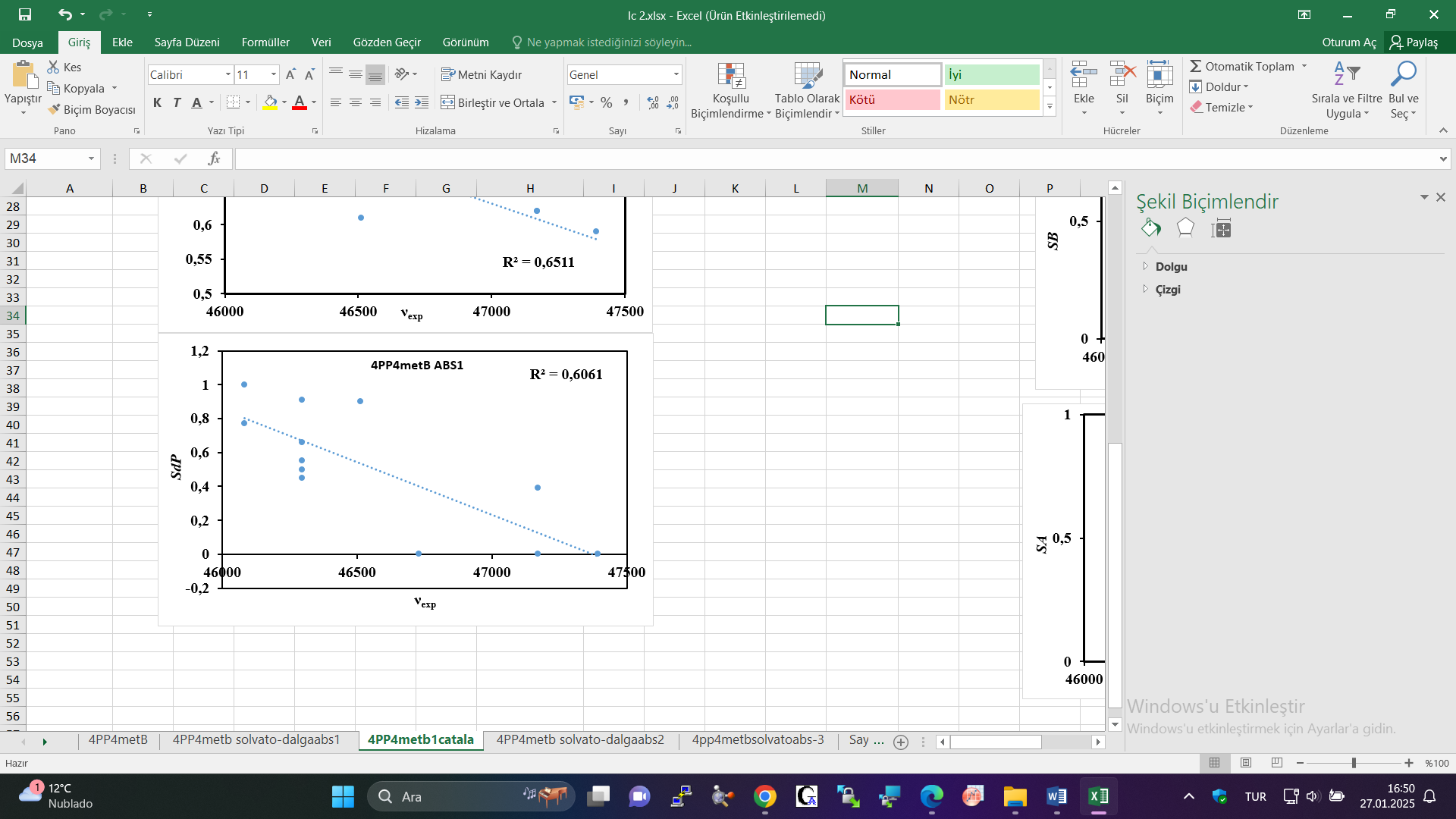

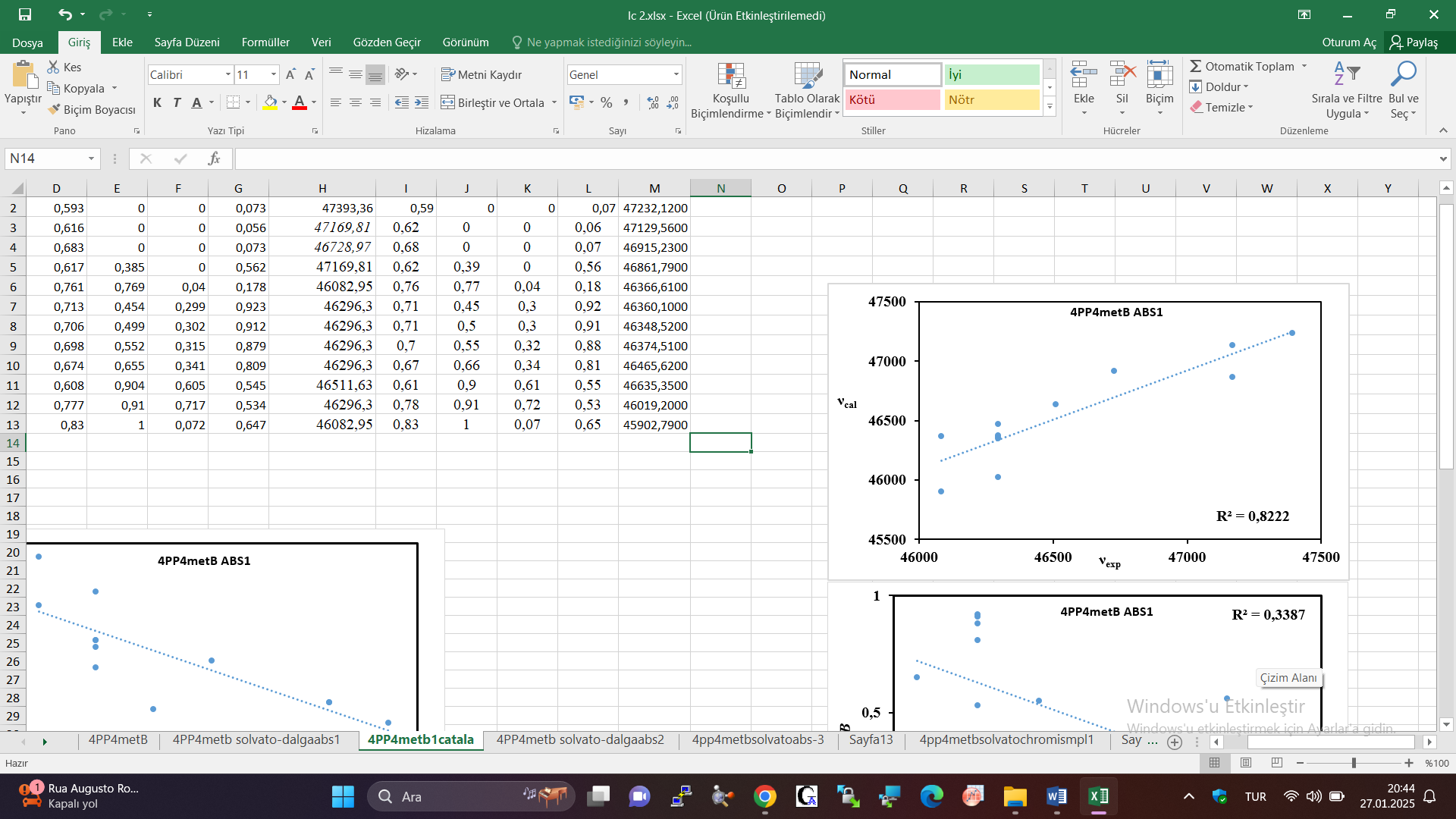


**Figure 3S.** The correlations of ν_cal_, *SP, SdP, SA* and *SB* versus ν_exp_ of λ_ABS1_ wavelength of 4PP4metB molecule.


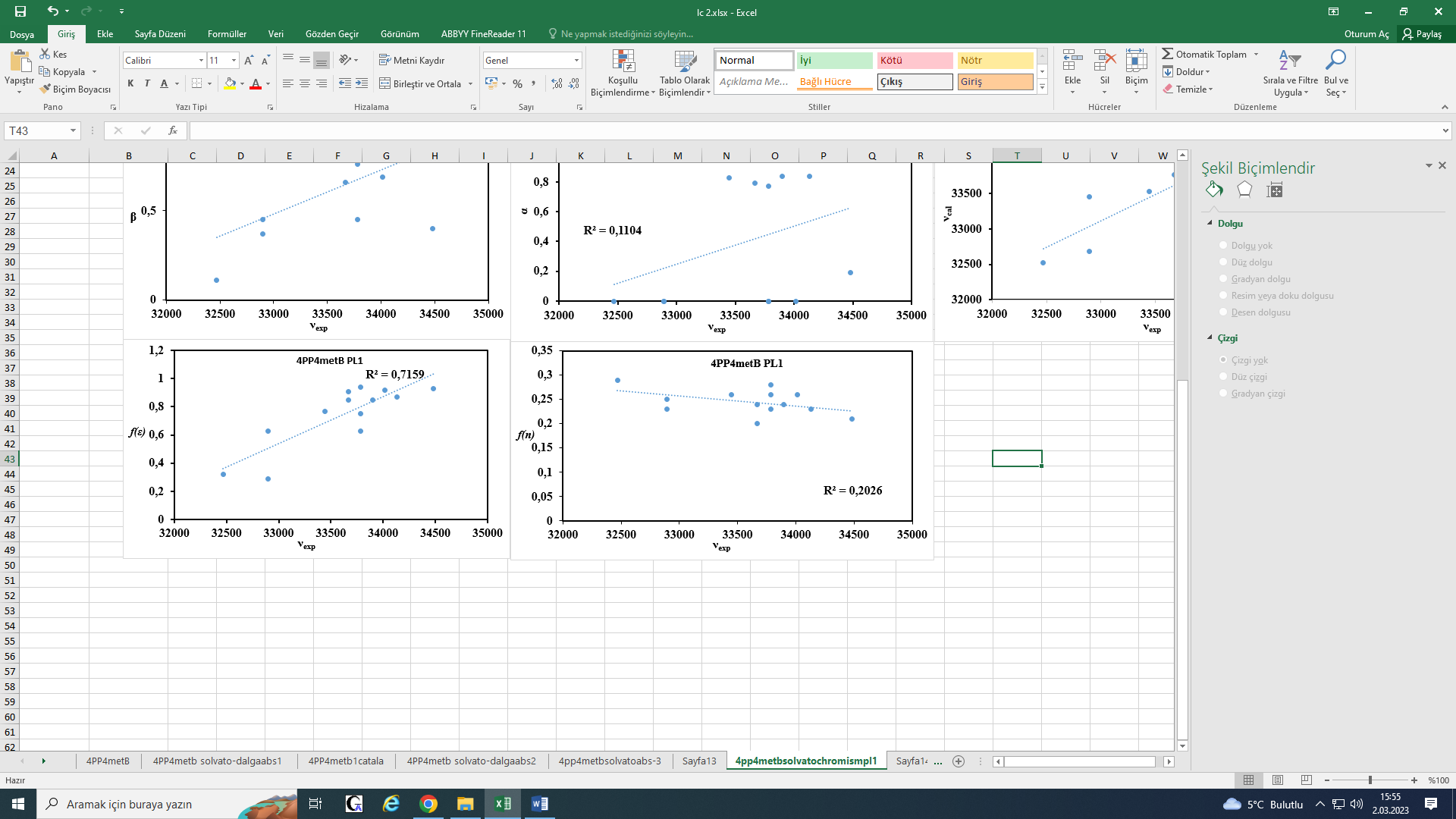

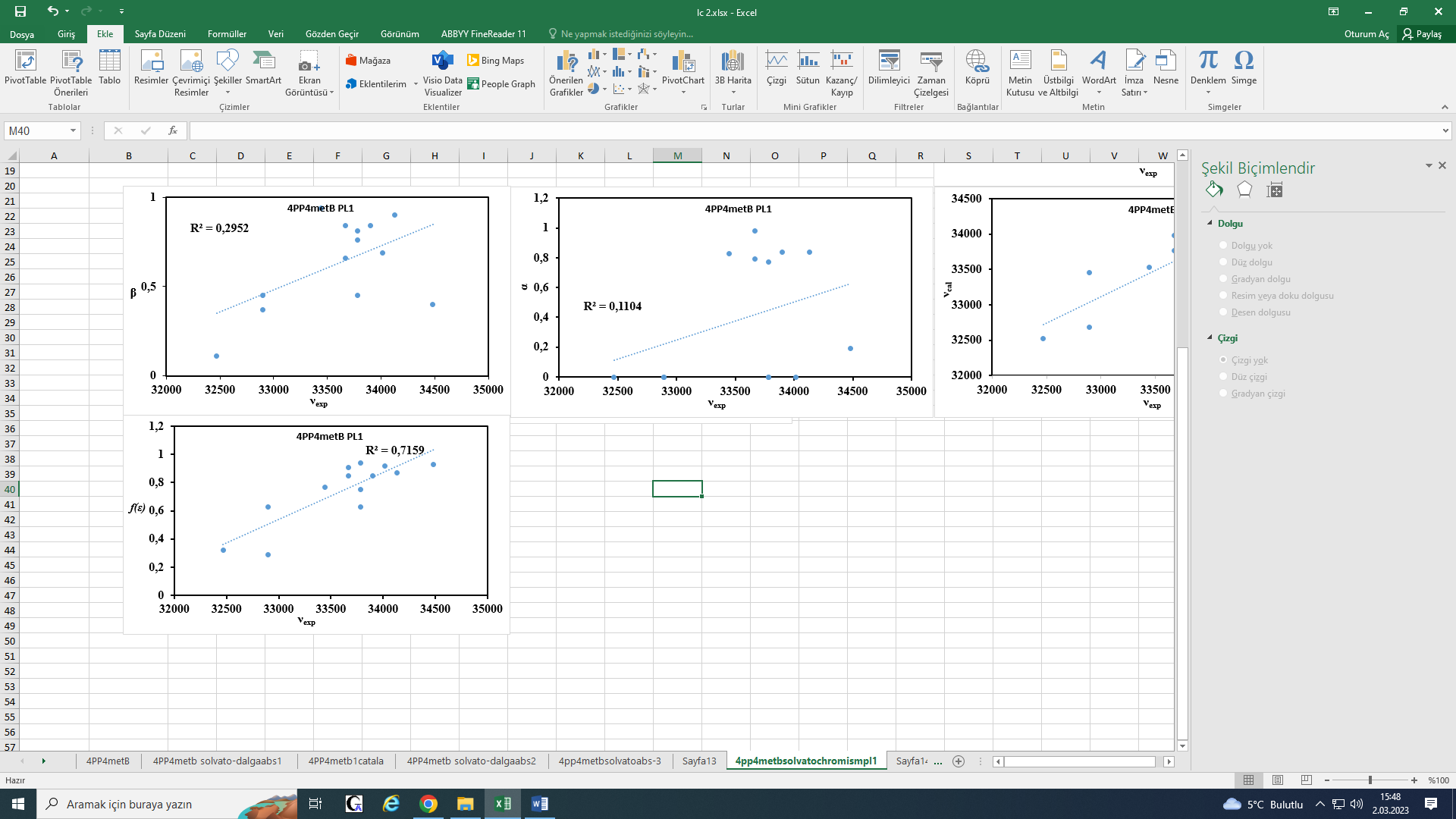

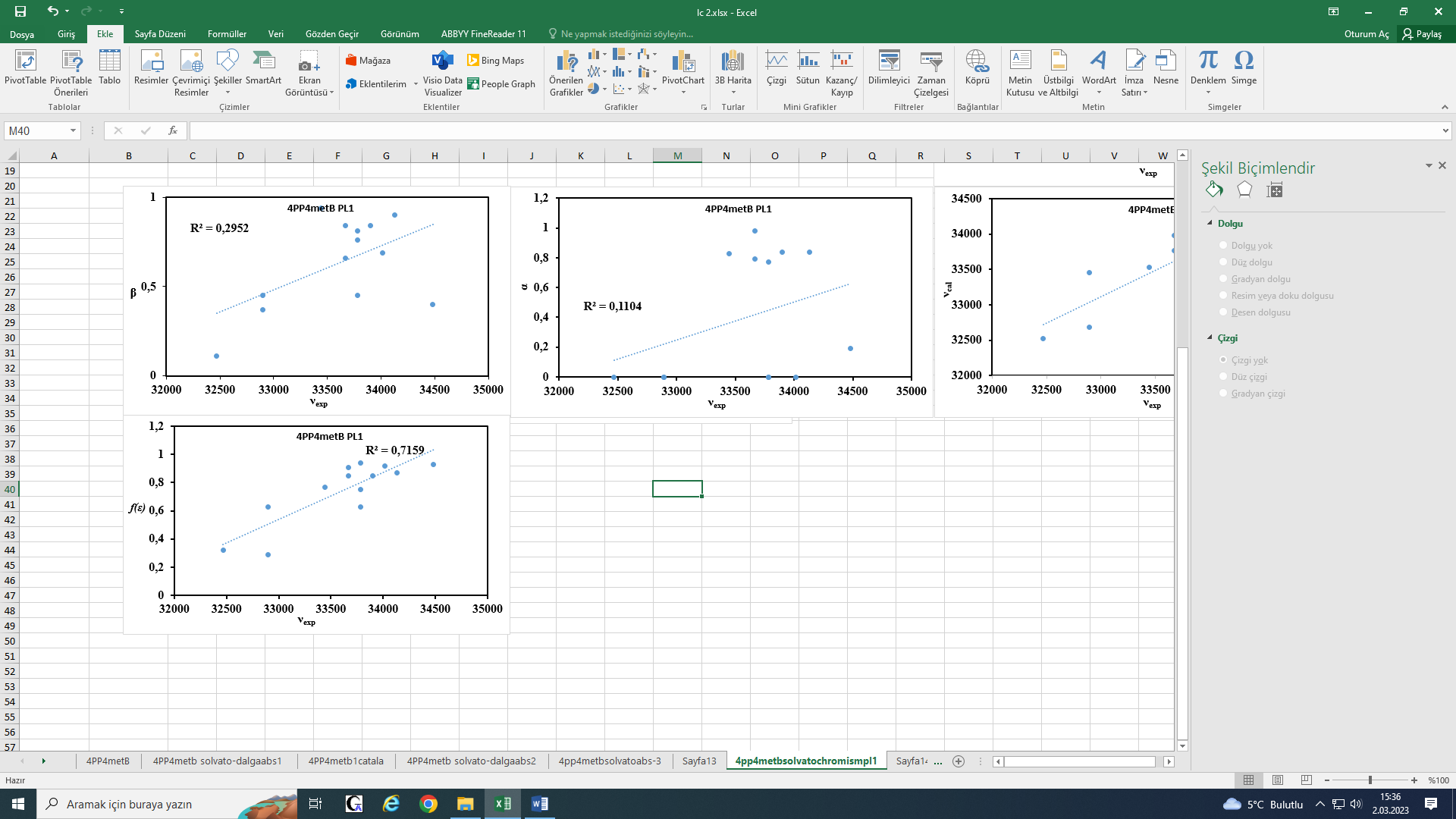

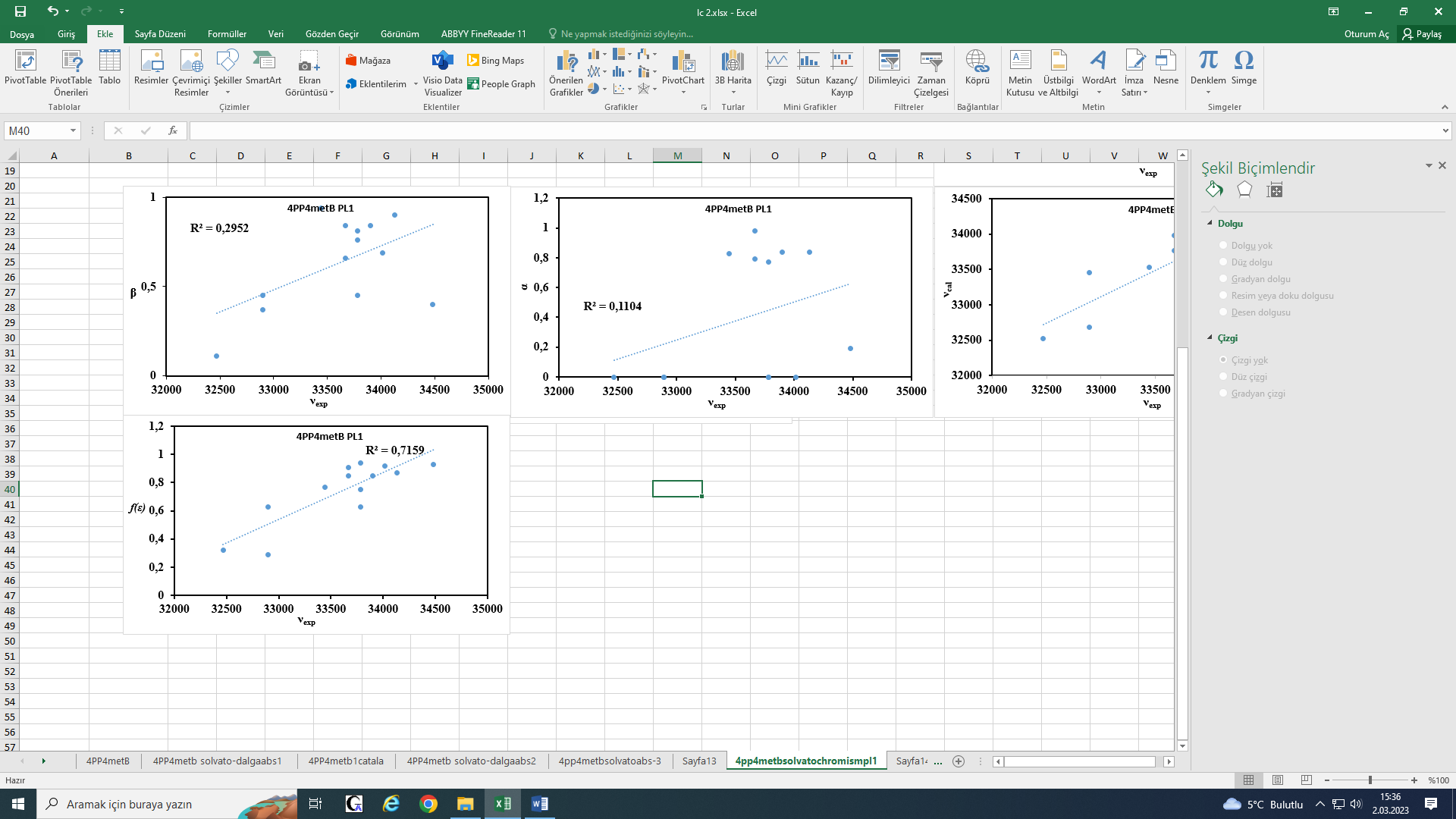

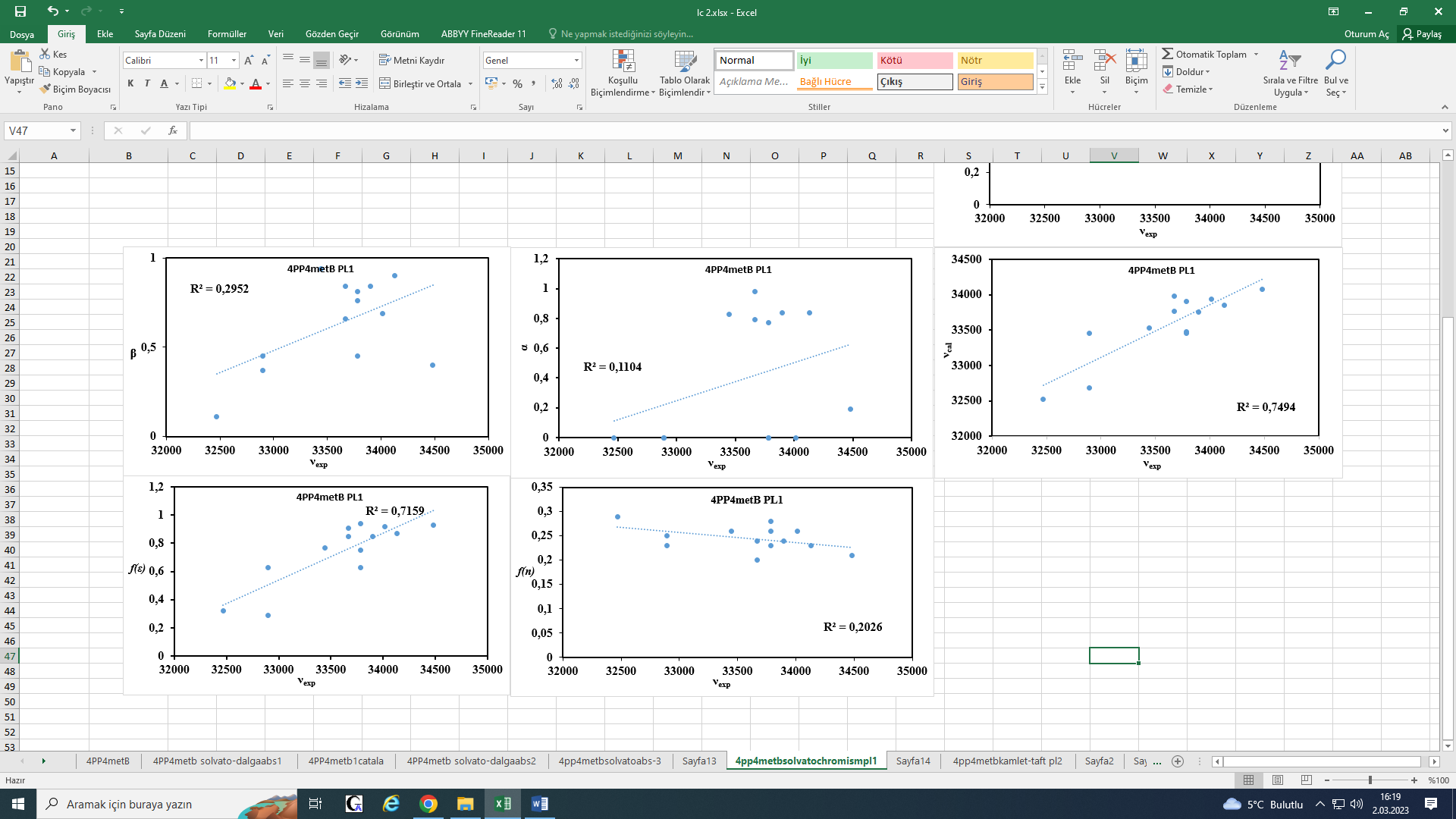


**Figure 4S.** The correlations of *ν_cal_, β, α, f(n)* and *f(ε)* versus ν_exp_ of λ_PL1_ wavelength of 4PP4metB molecule.


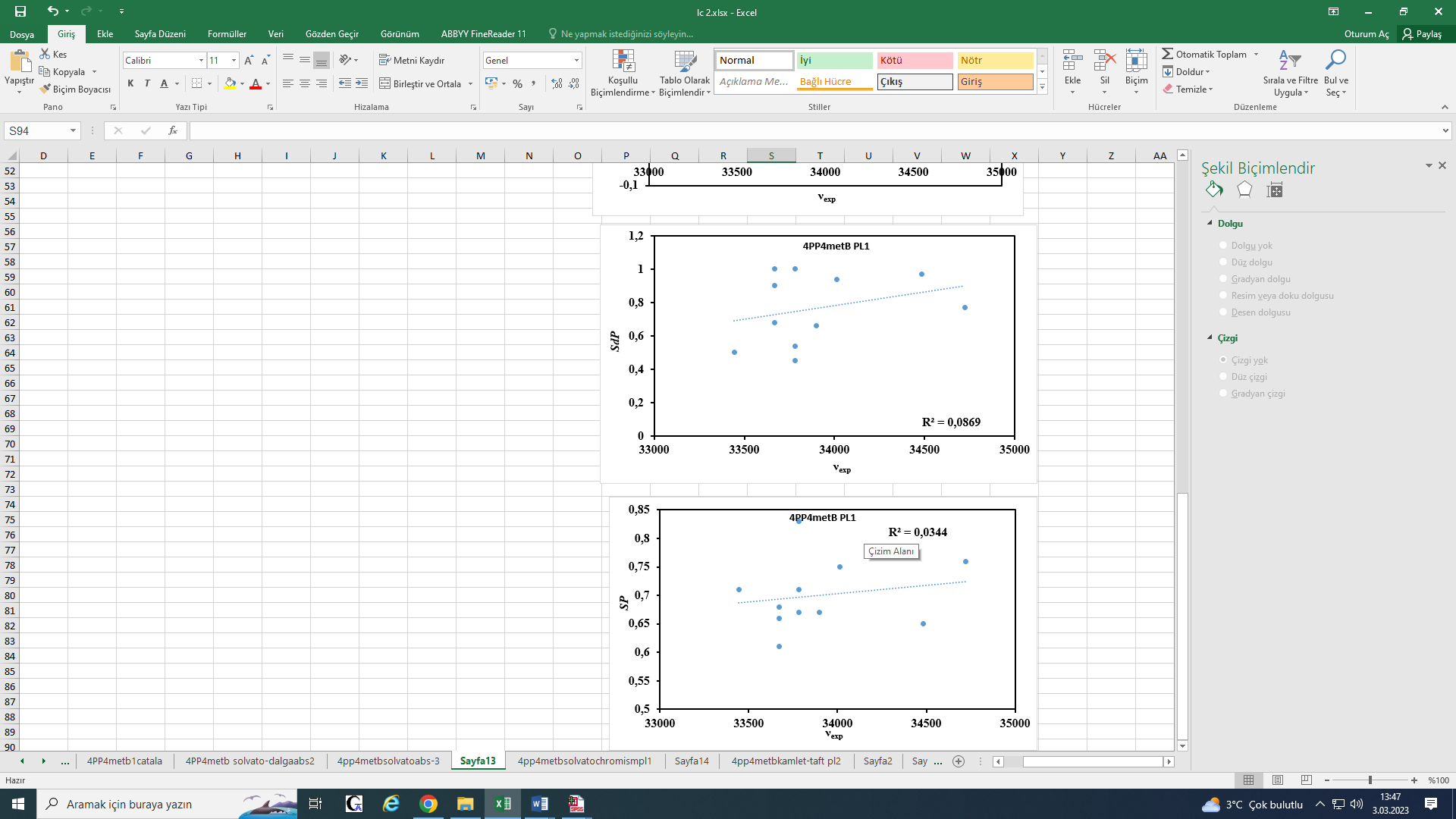

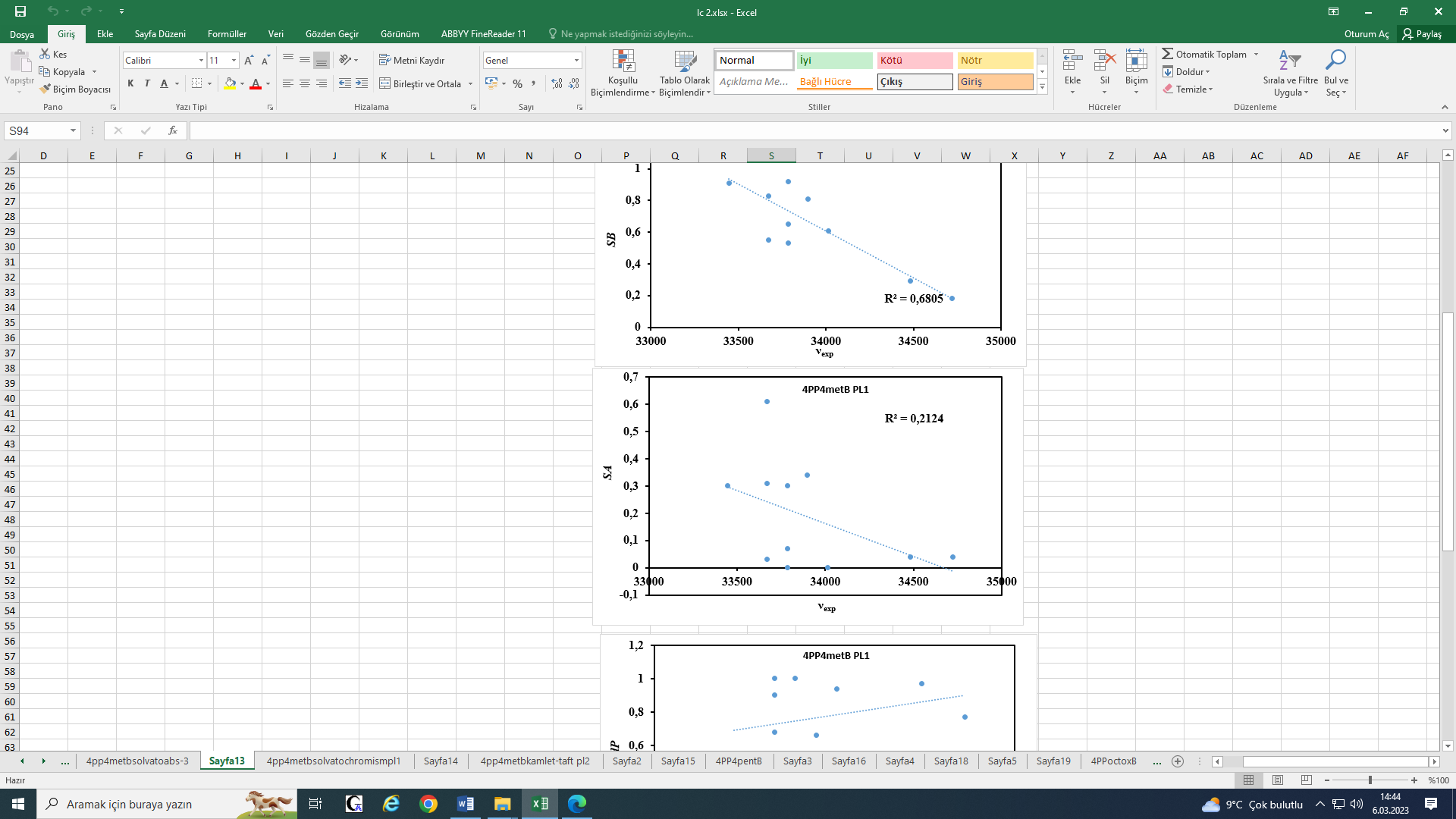

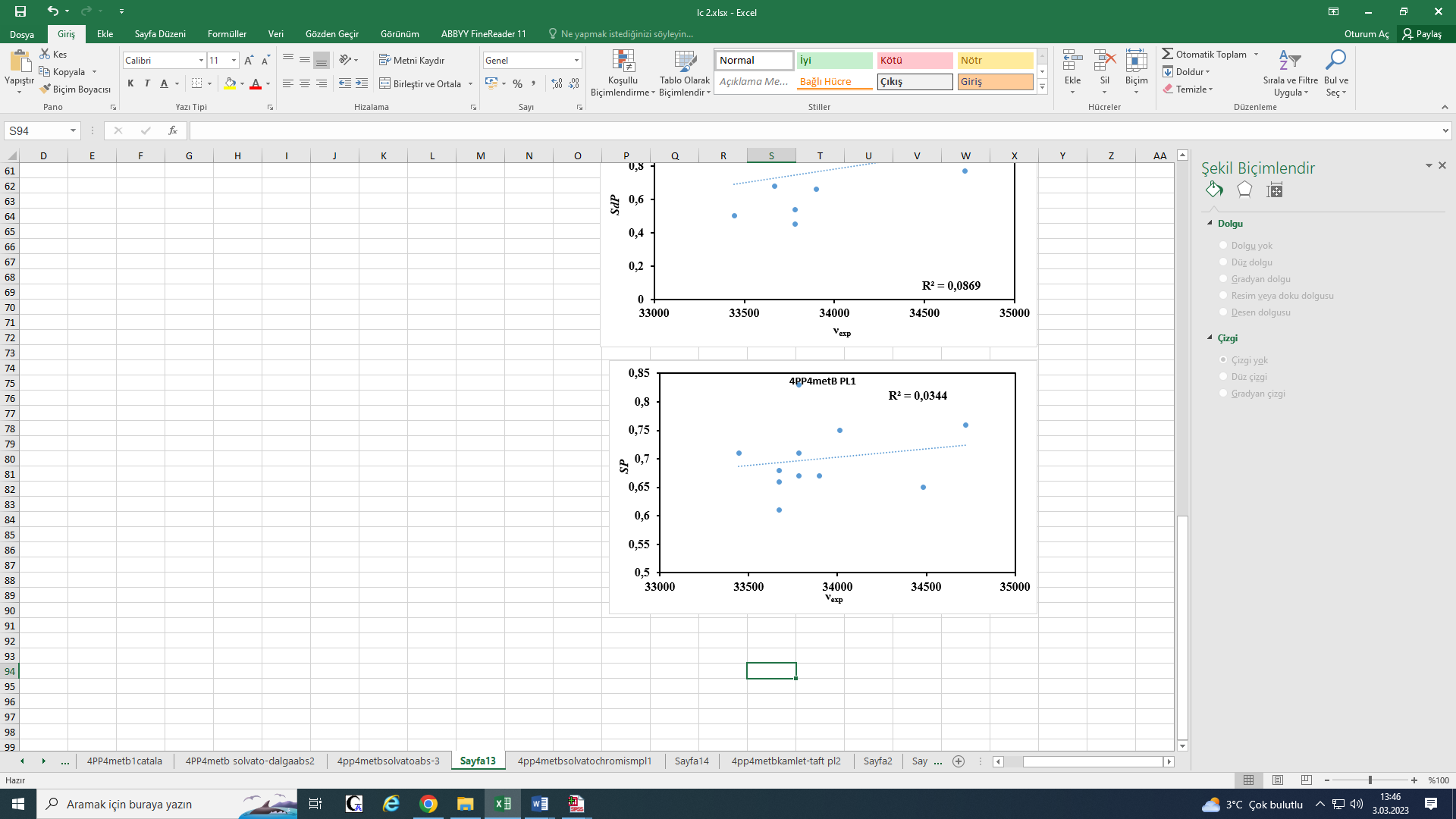

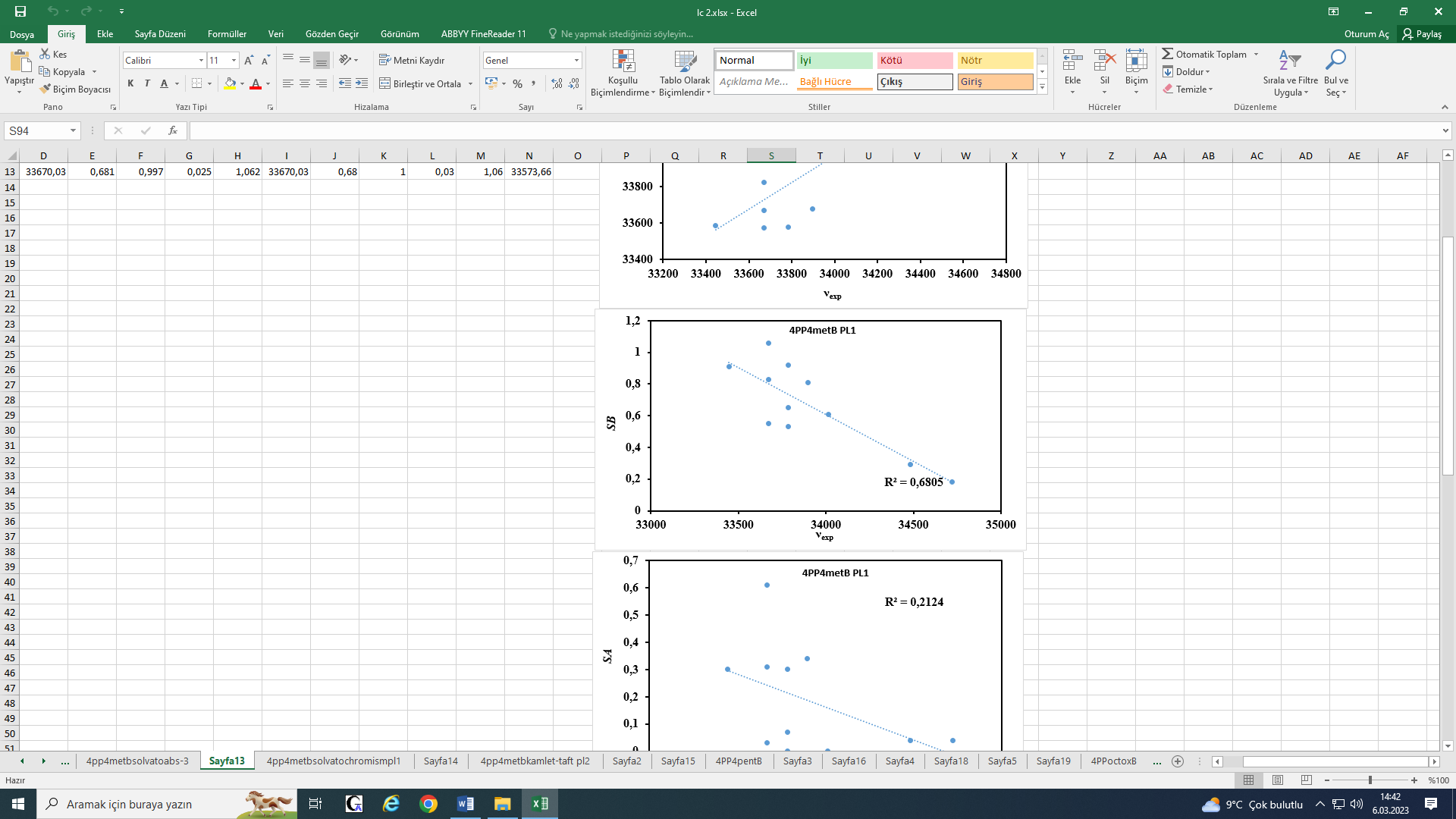

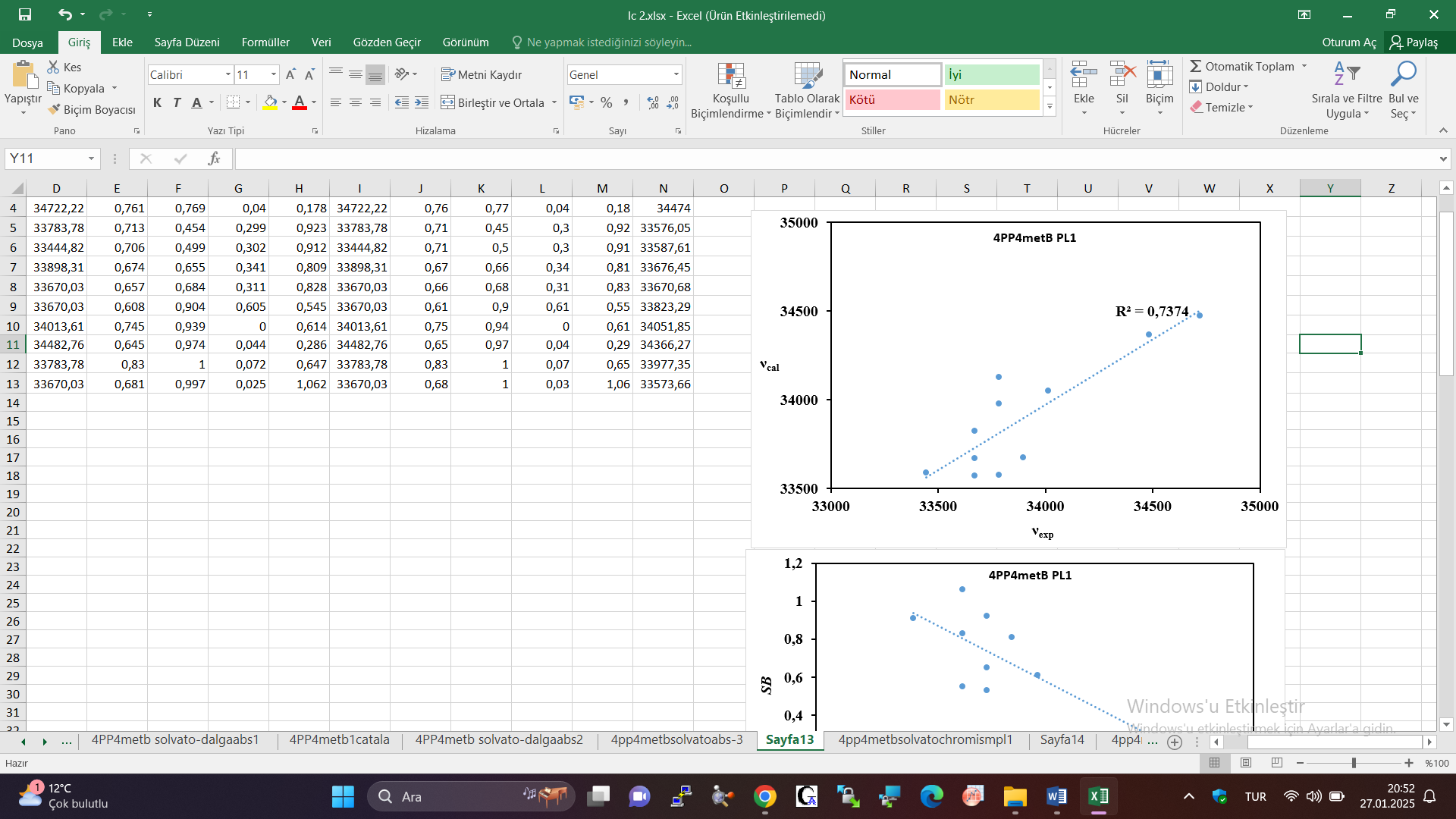


**Figure 5S.** The correlations of *ν_cal_, SP, SdP, SA* and *SB* versus ν_exp_ of λ_PL1_ wavelength of 4PP4metB molecule.


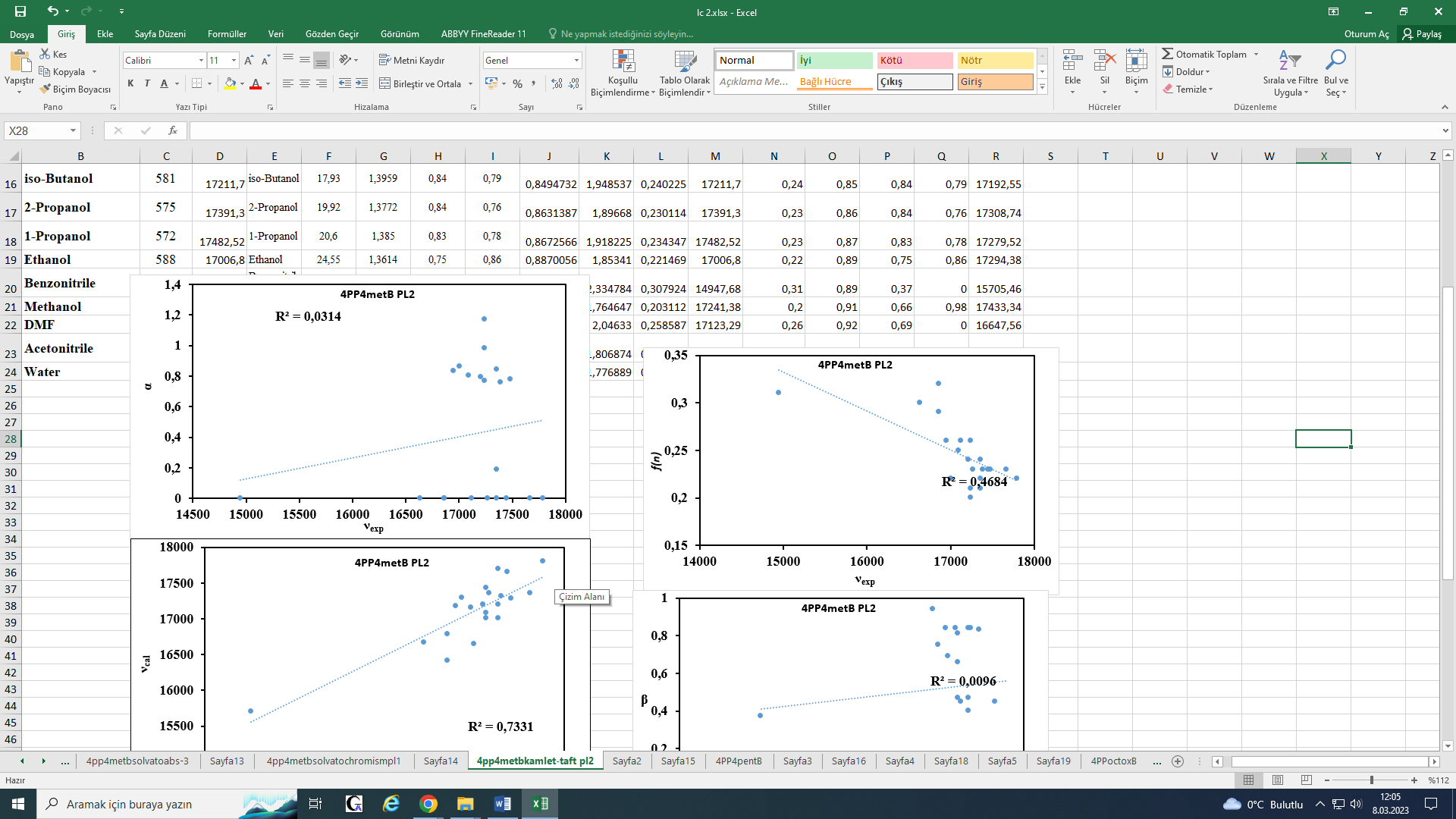

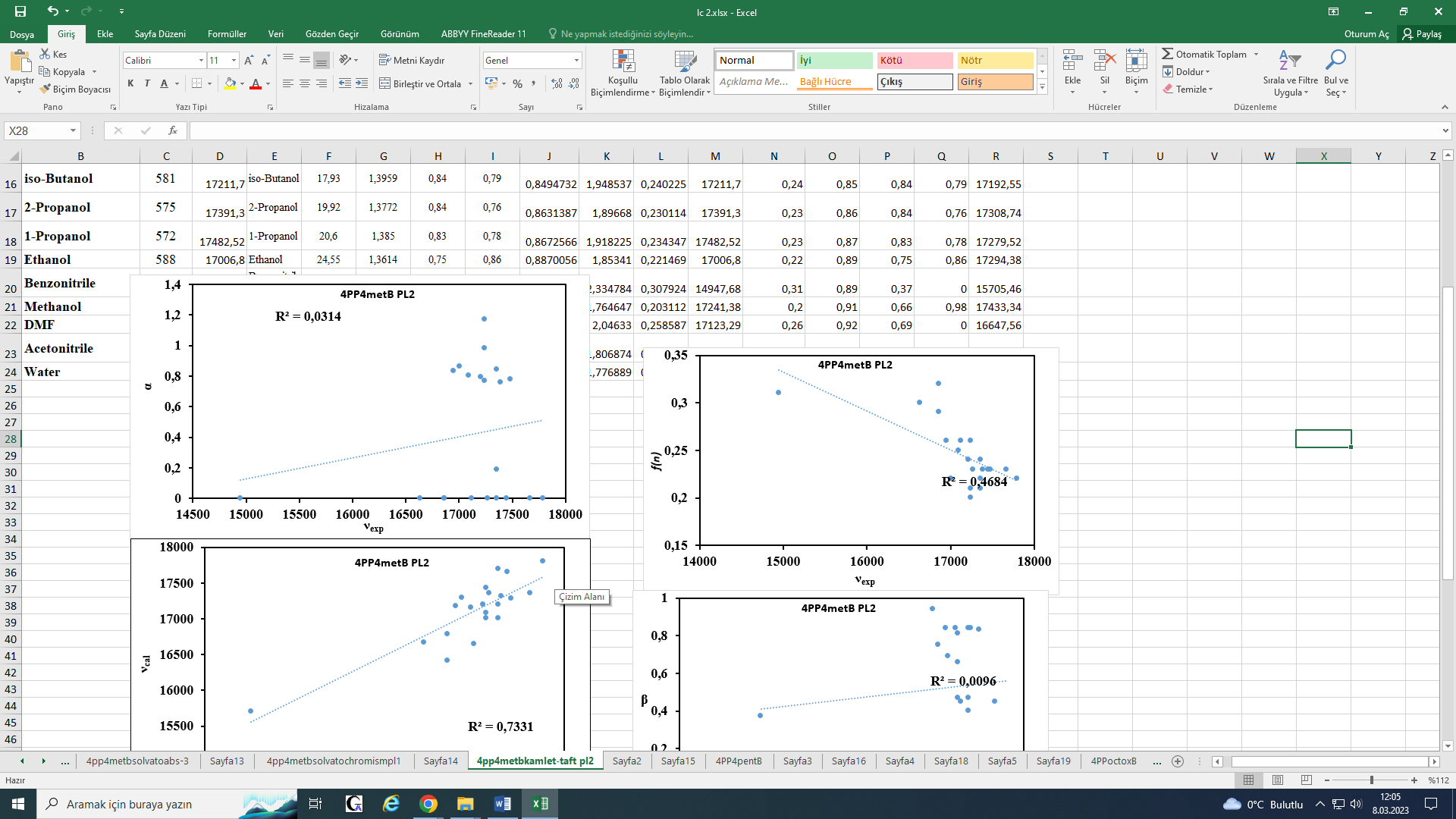

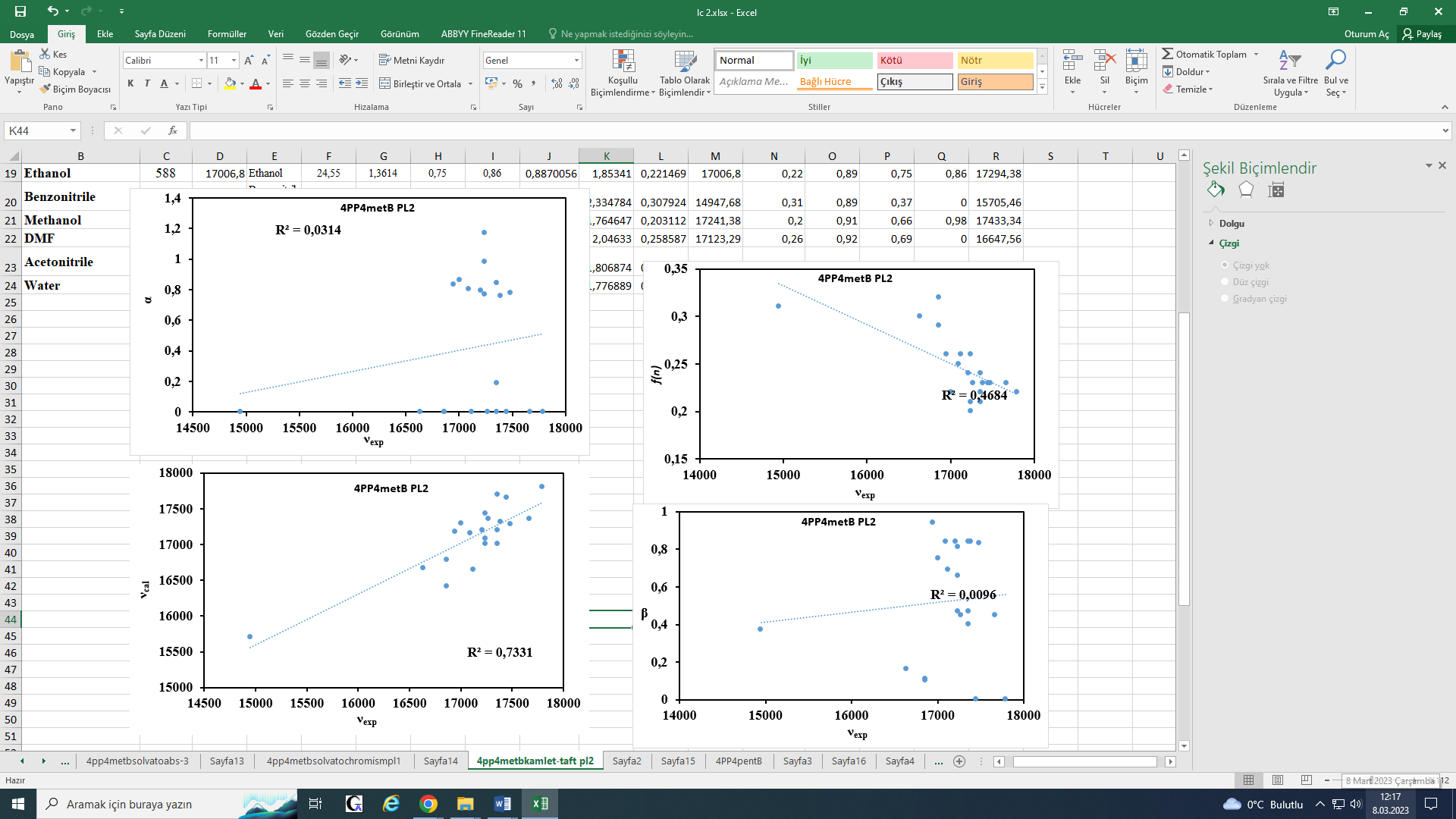

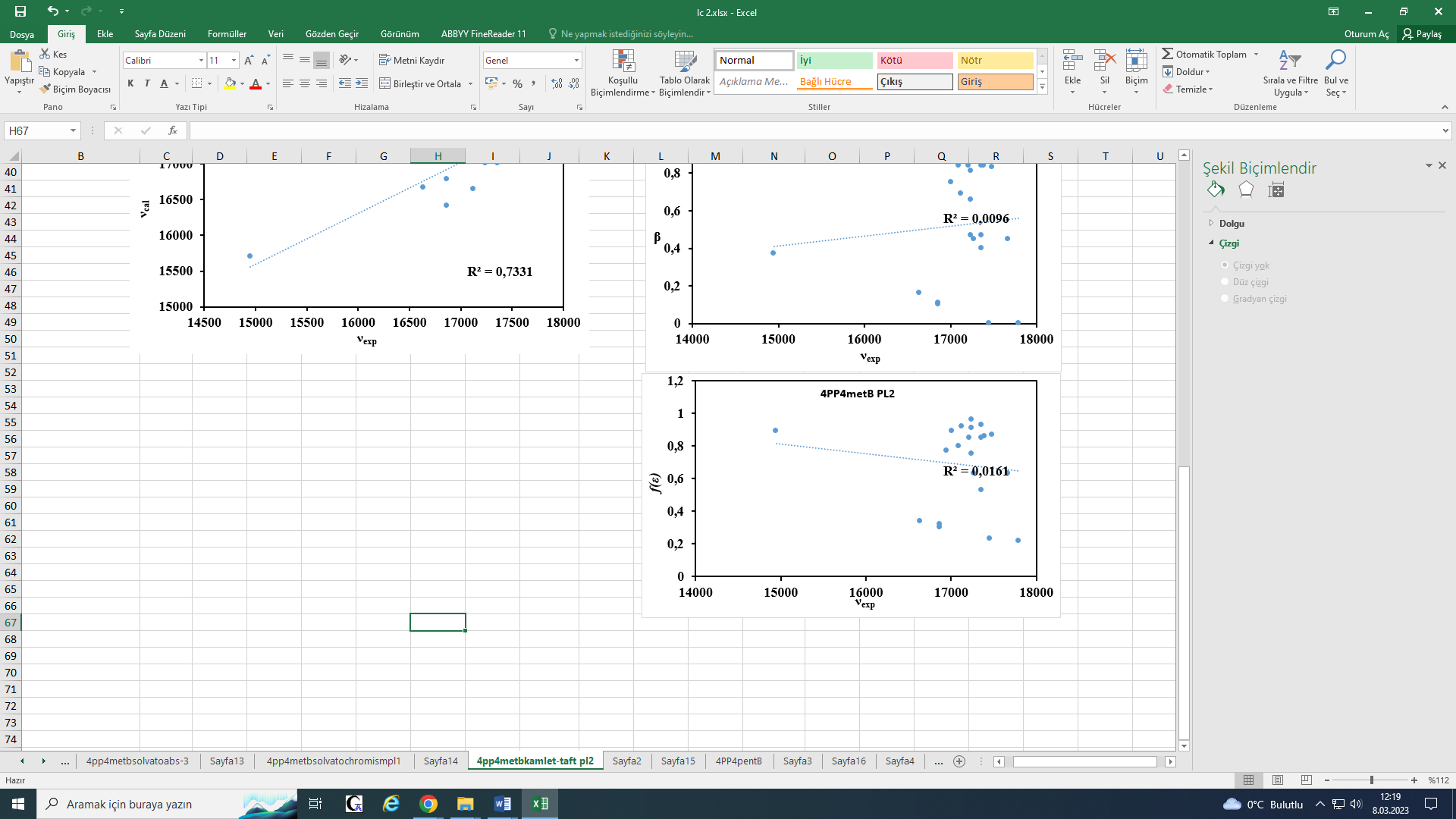

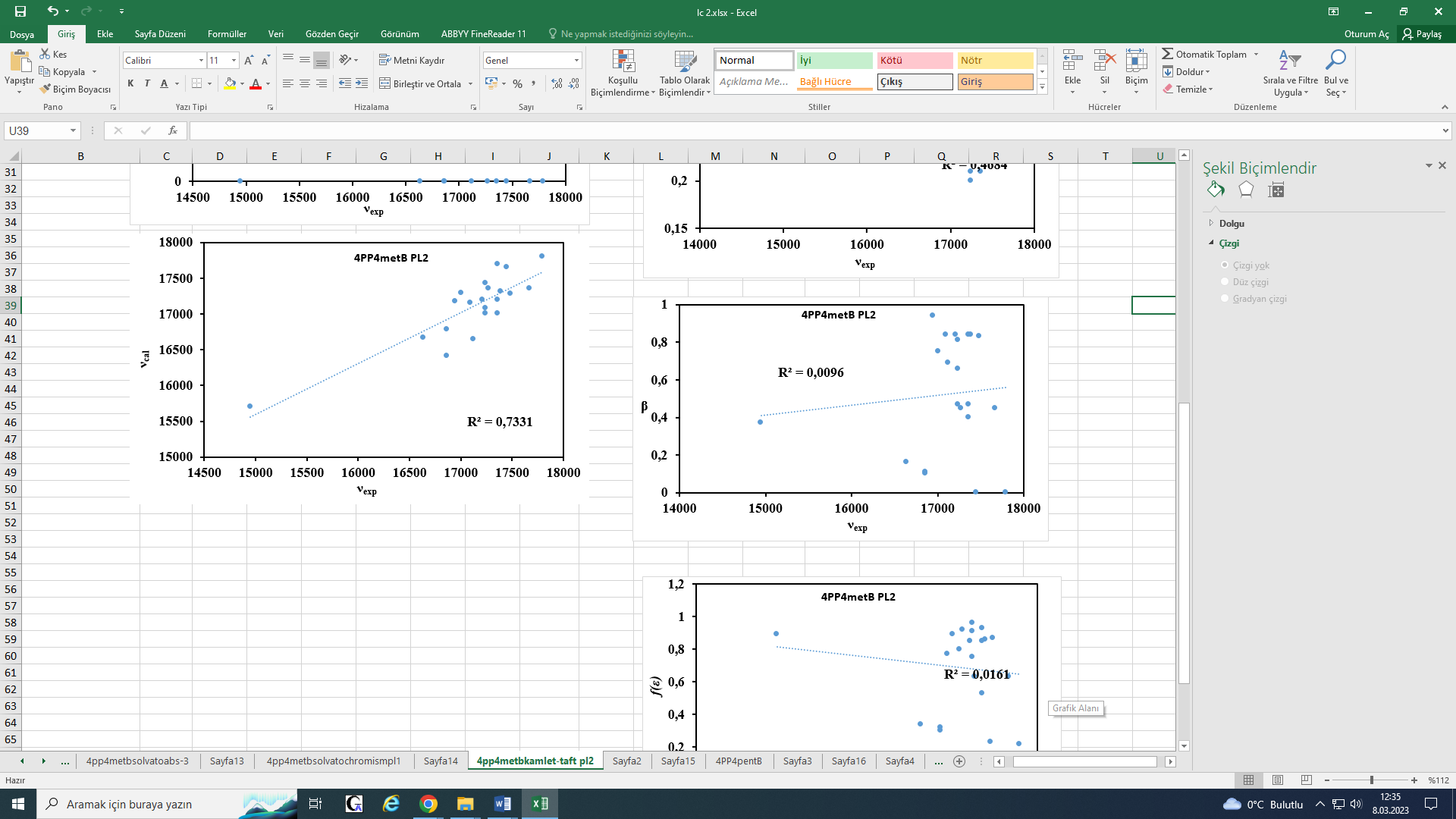


**Figure 6S.** The correlations of *ν_cal,_ β, α, f(n)* and *f(ε)* versus ν_exp_  of λ_PL2_ wavelength of 4PP4metB molecule.


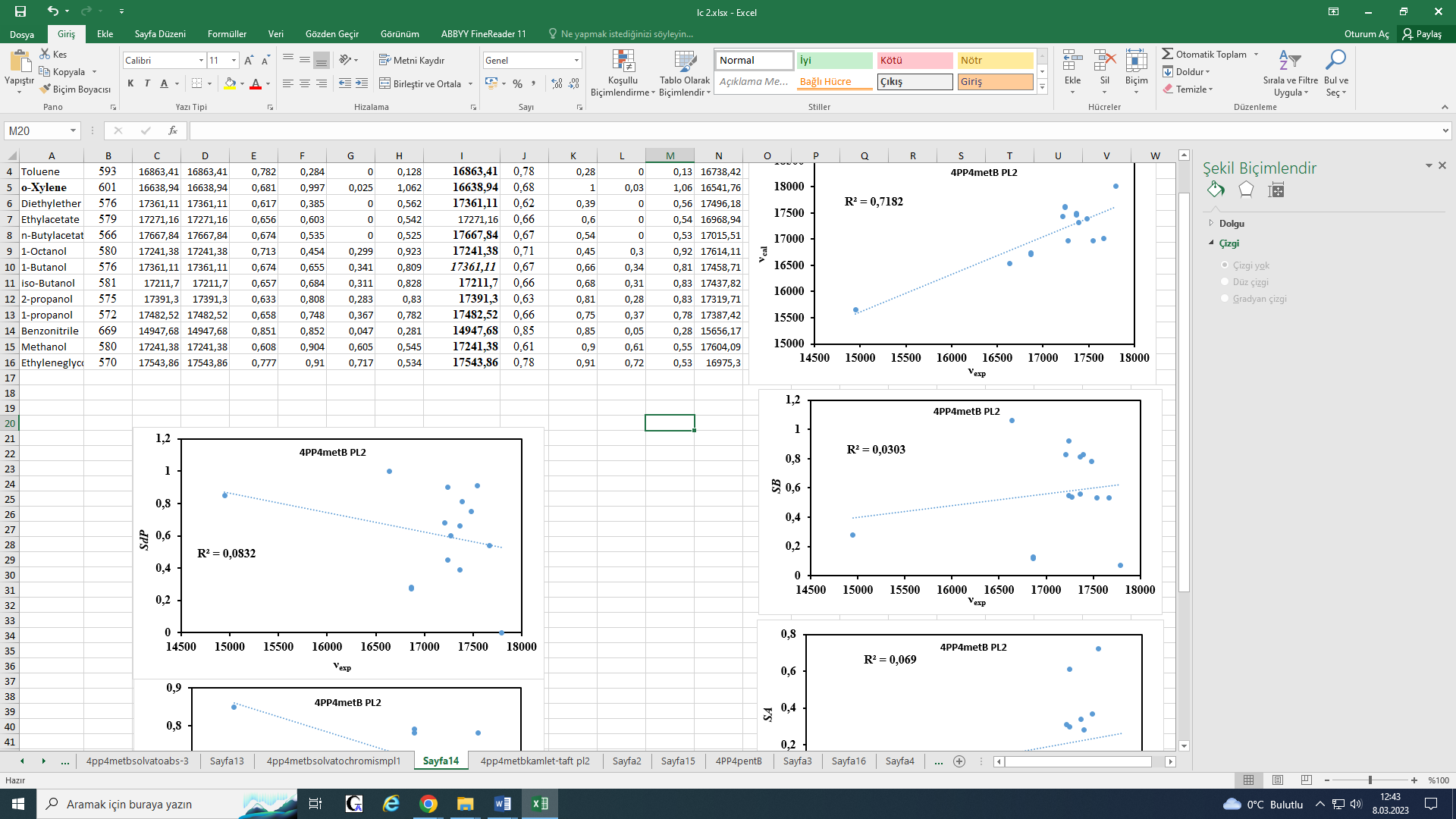

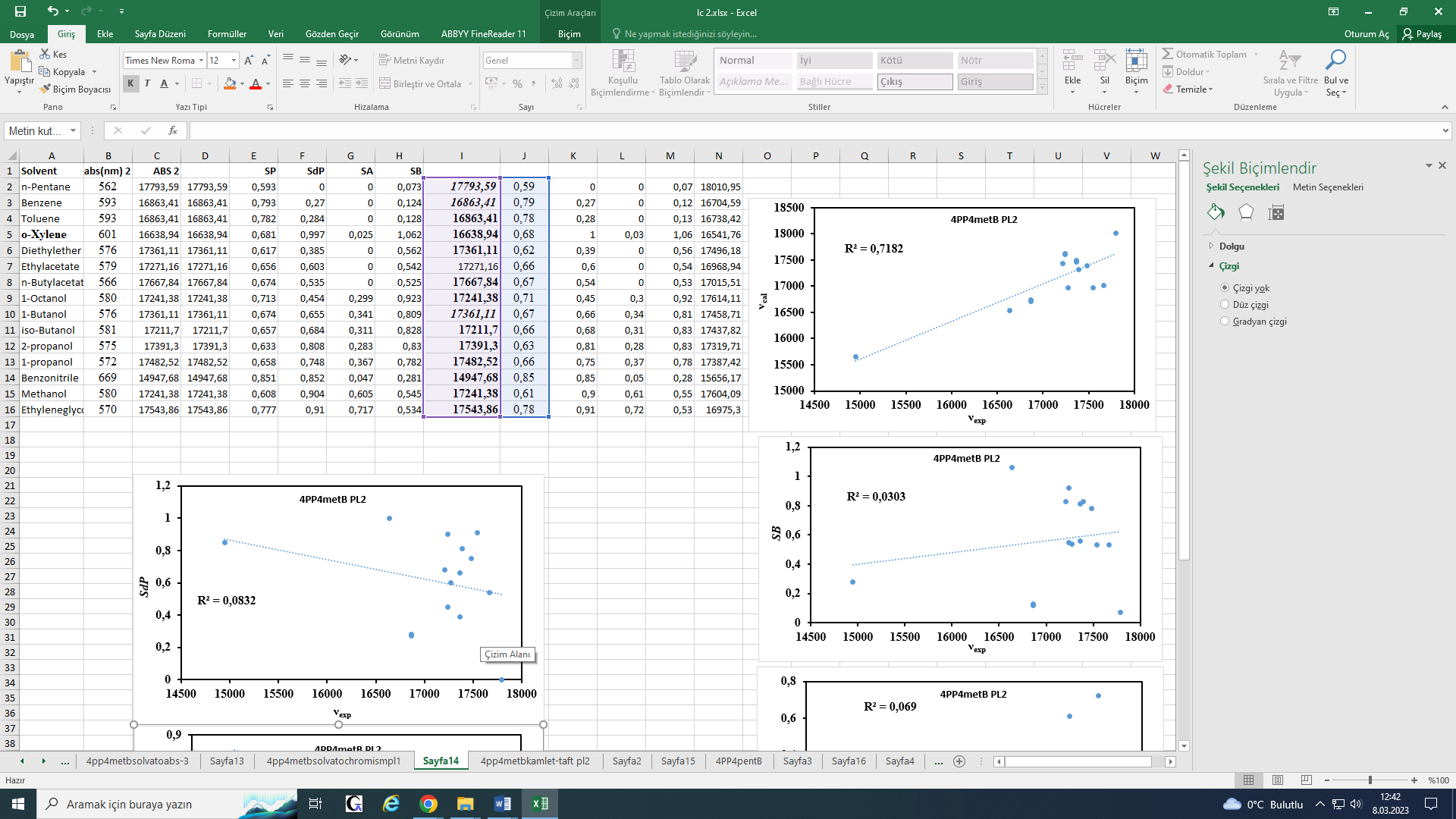

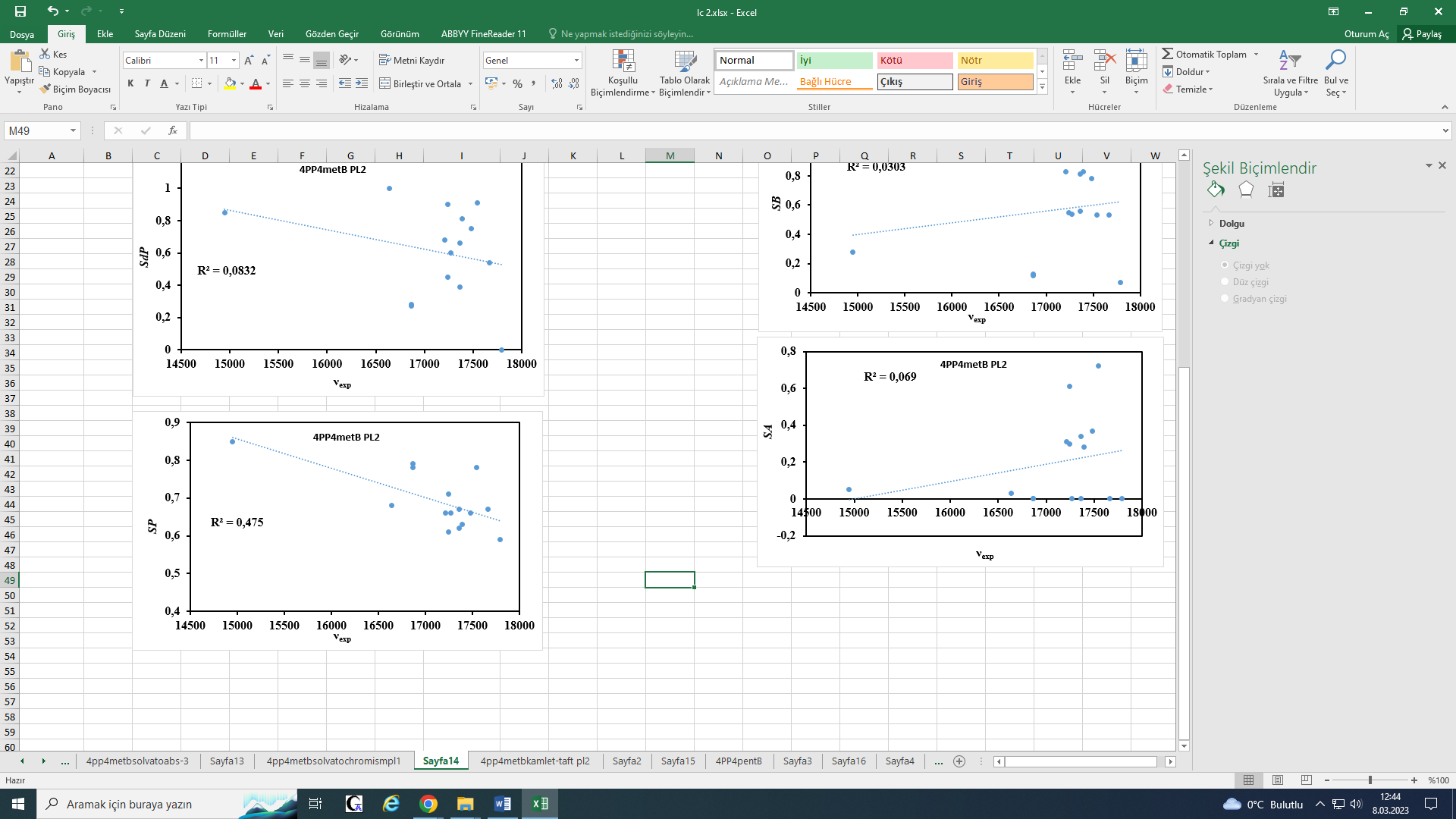

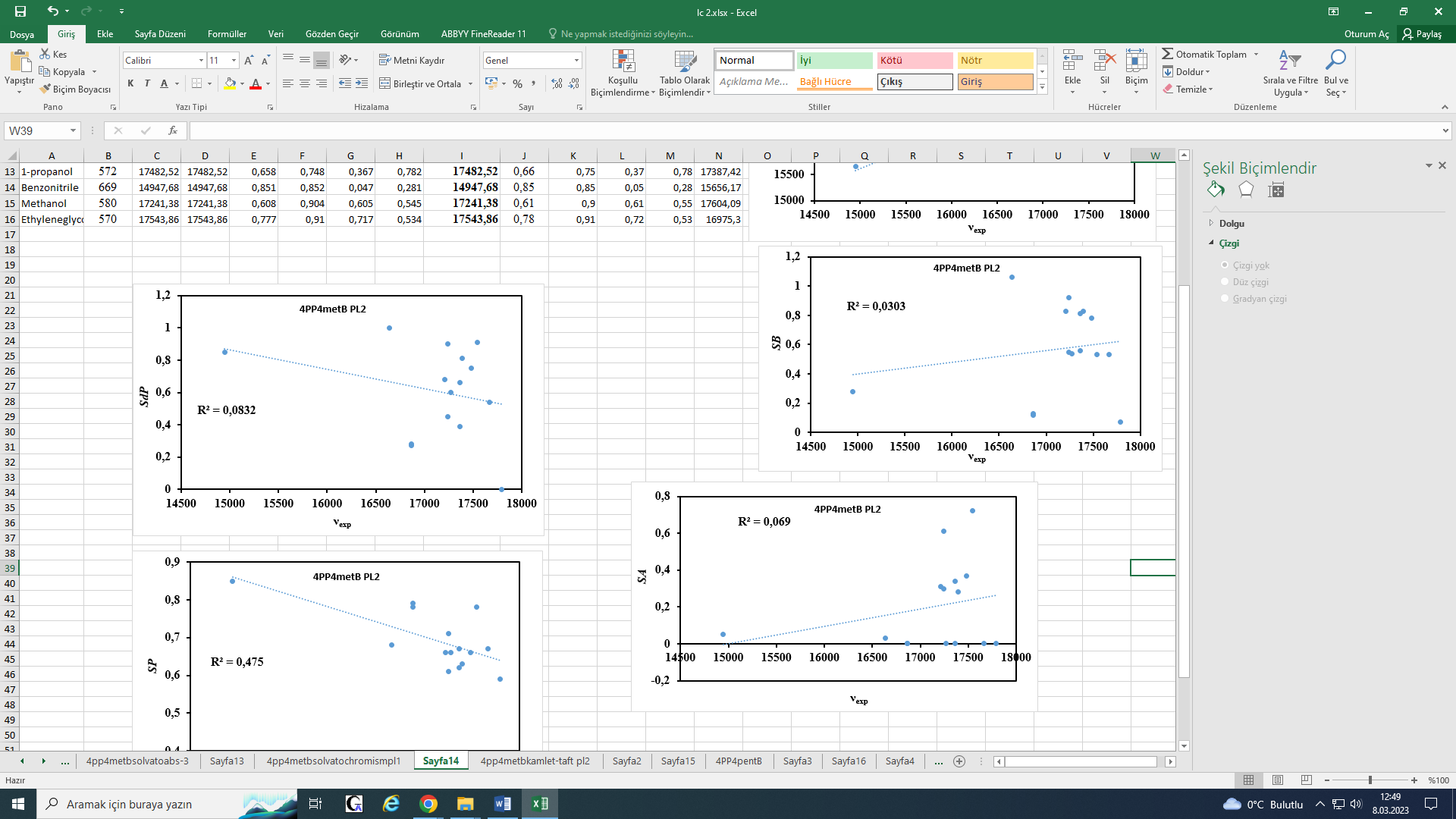

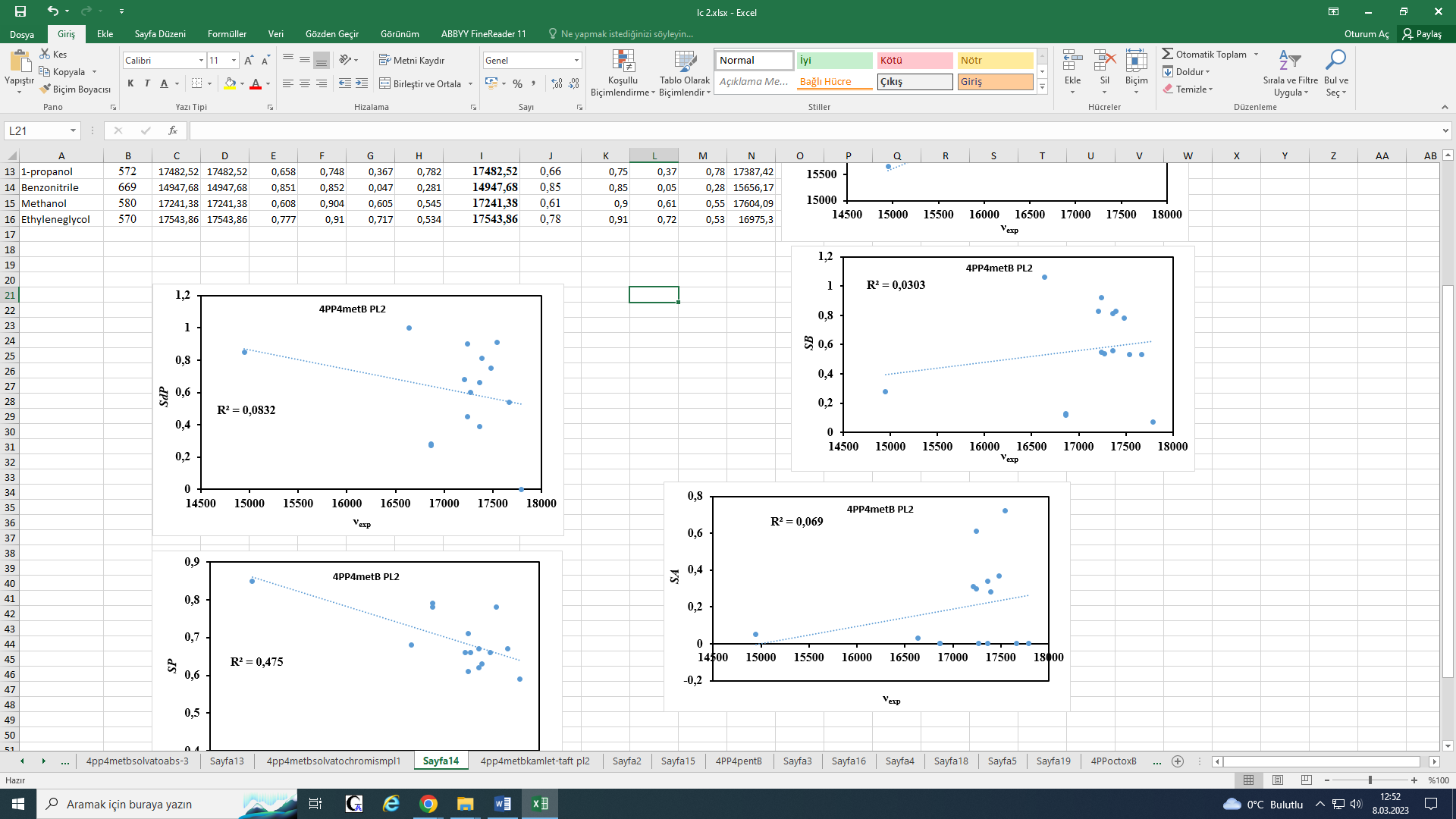


**Figure 7S.** The correlations of *ν_cal_, SP, SdP, SA* and *SB* versus ν_exp_  of λ_PL2_ wavelength of 4PP4metB molecule.


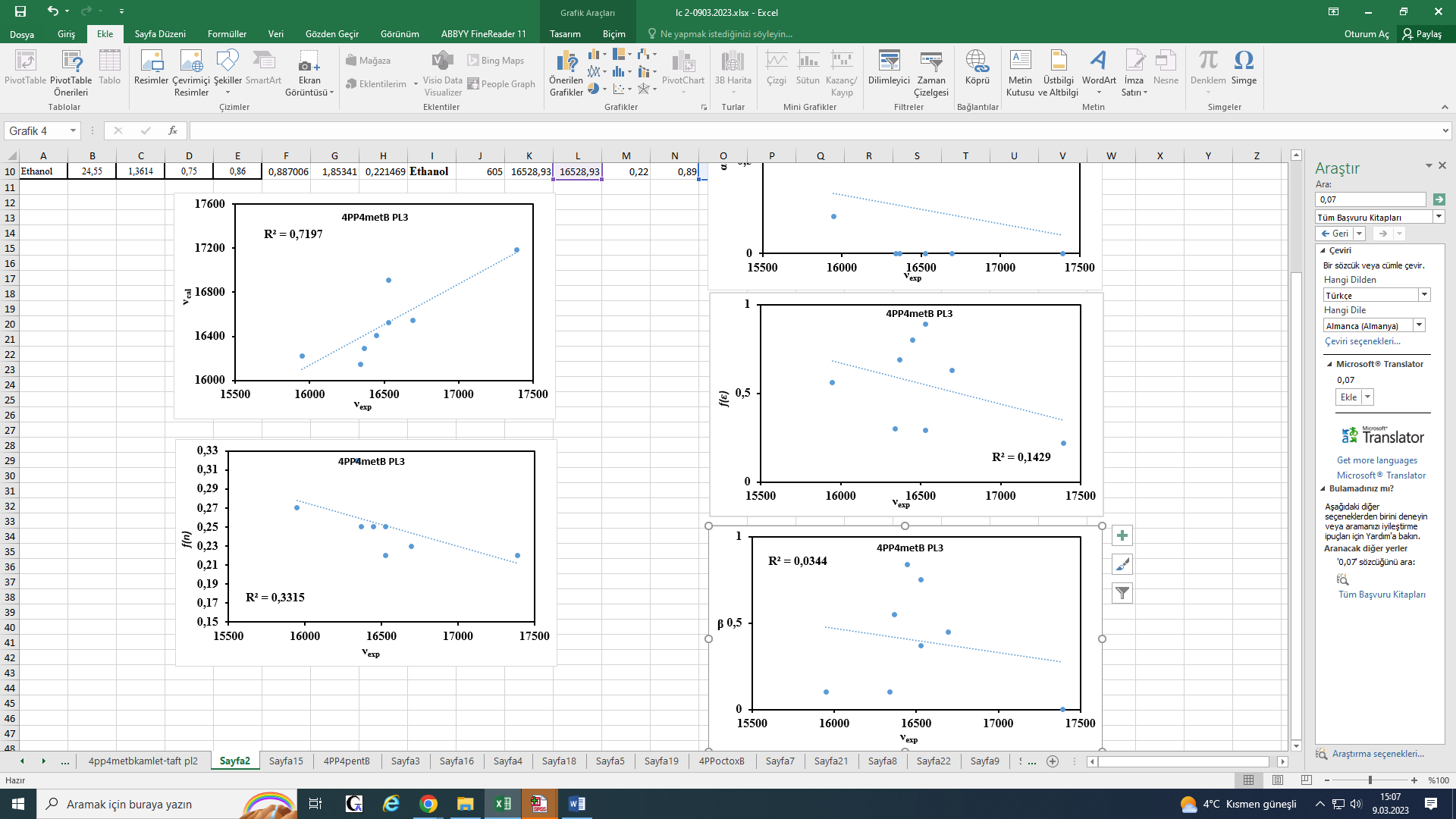

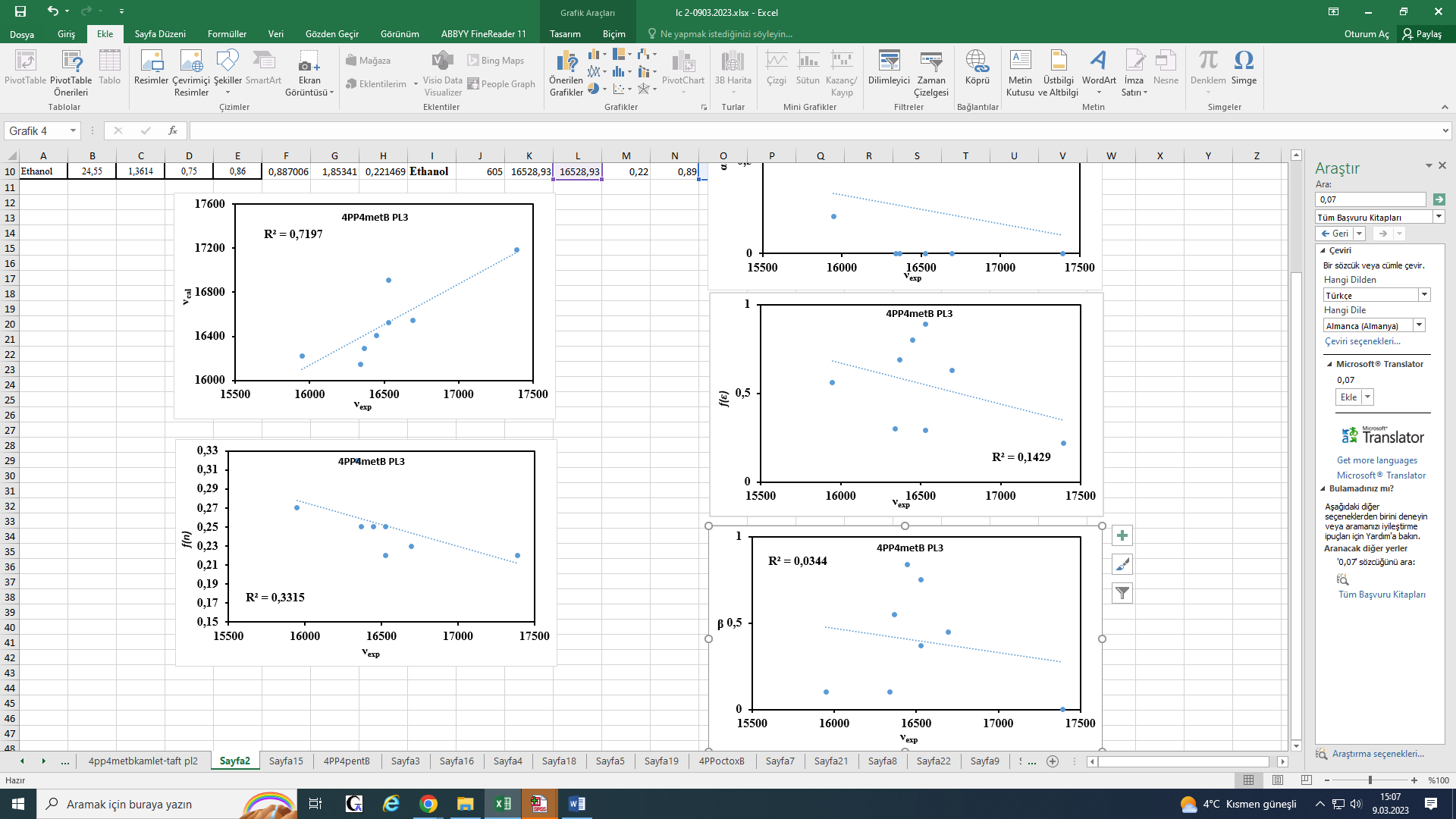

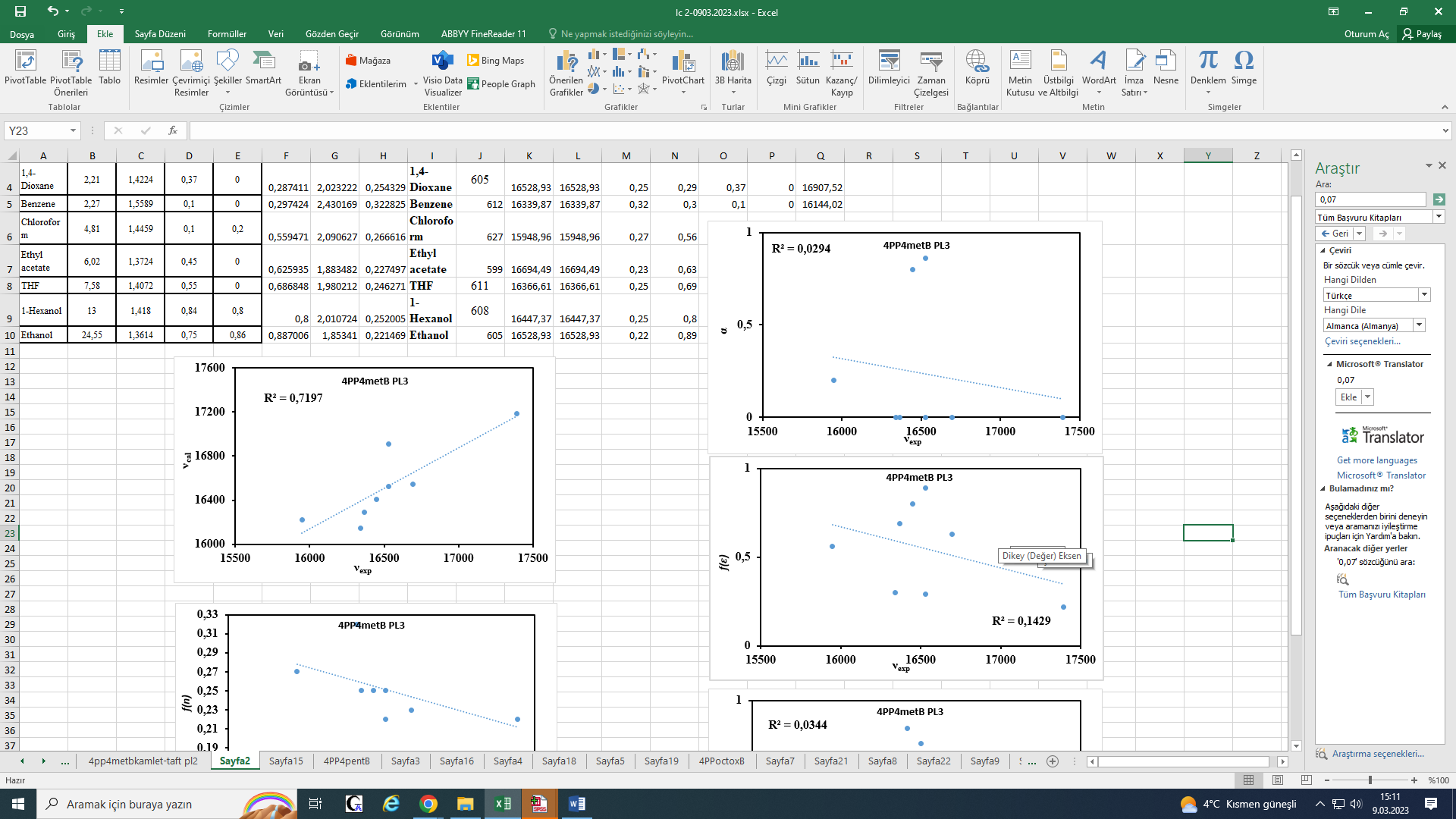

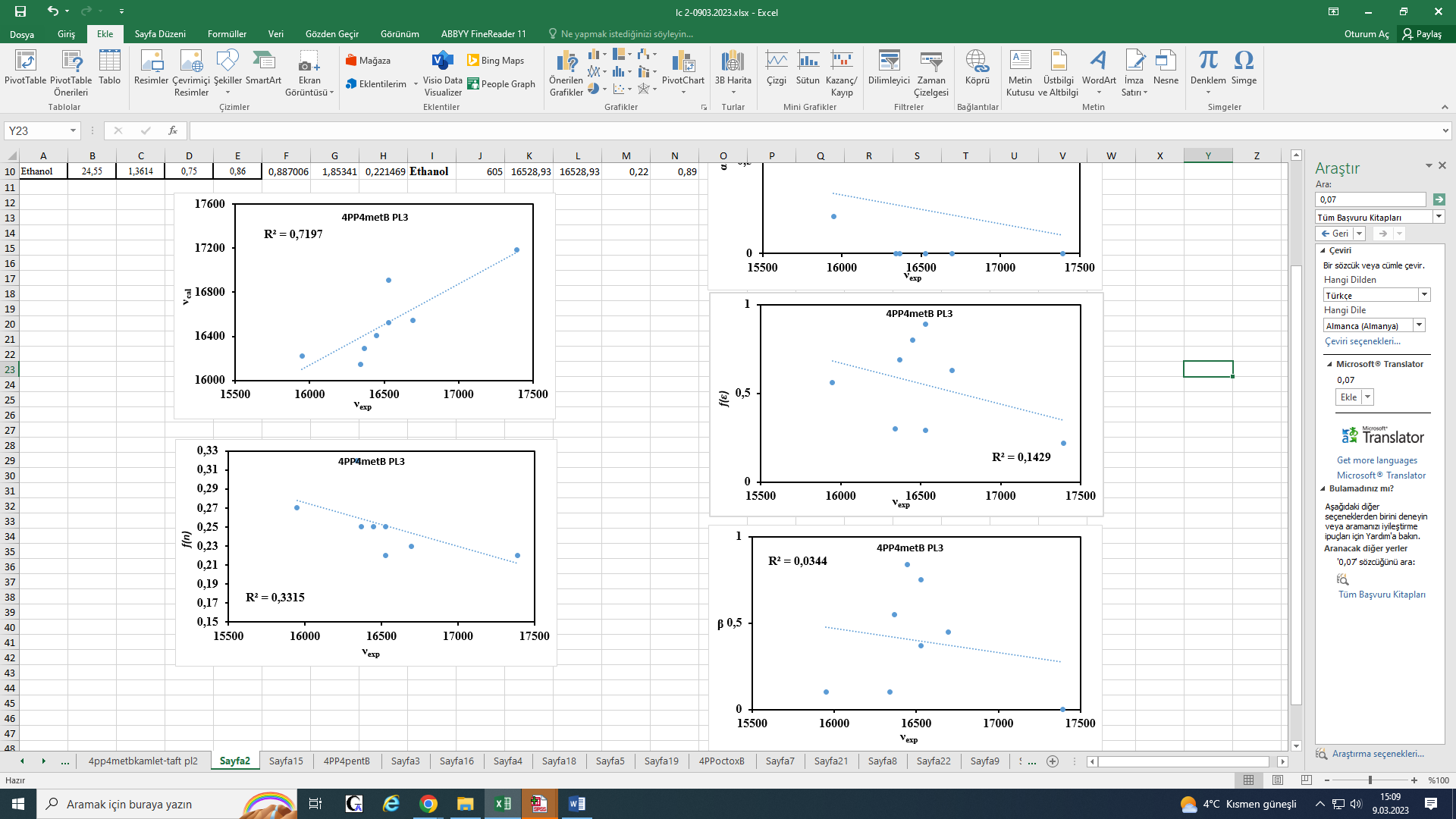

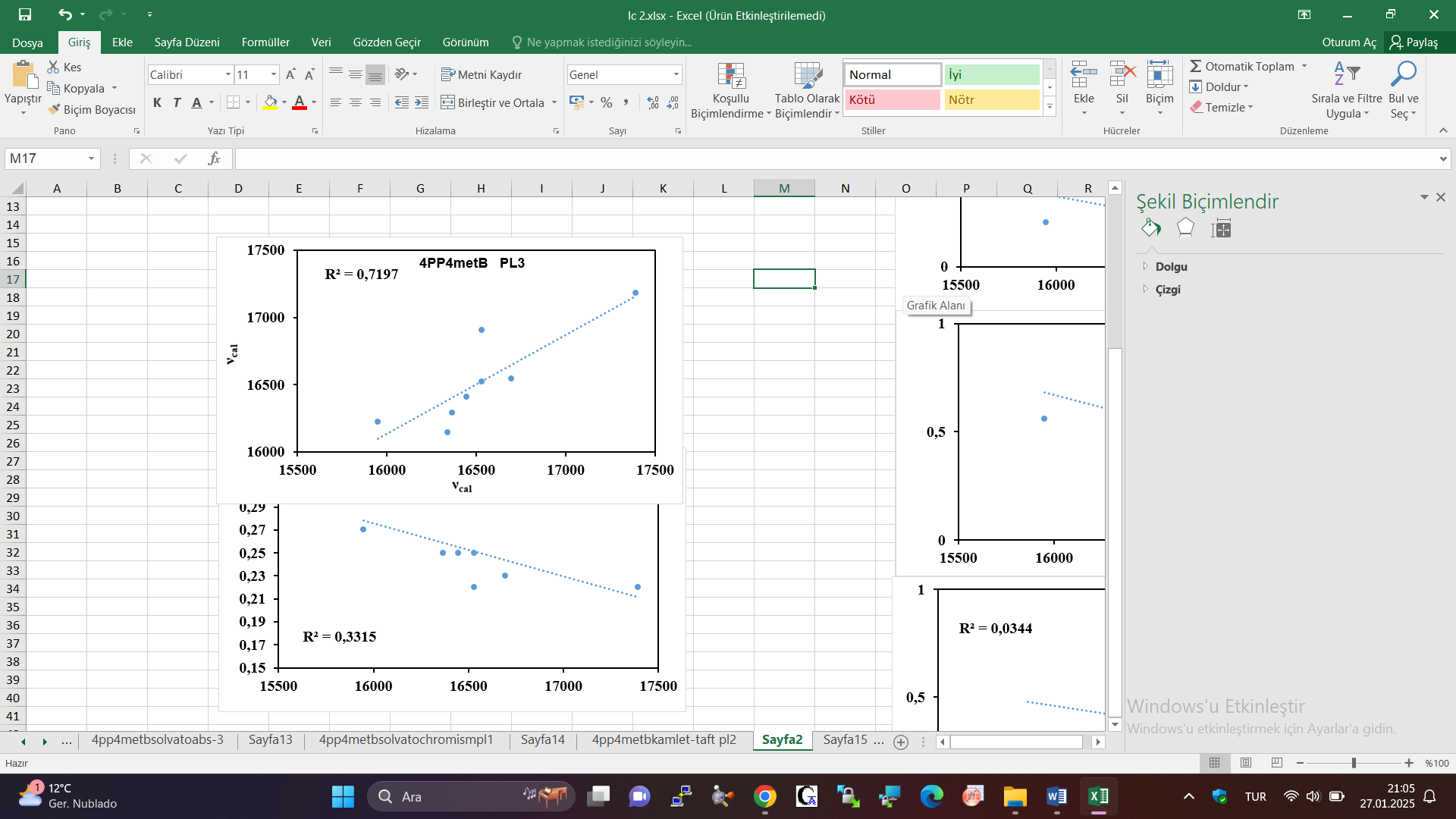


**Figure 8S.** The correlations of *ν_cal,_ β, α, f(n)* and *f(ε)* versus ν_exp_ of λ_PL3_ wavelength of 4PP4metB molecule.


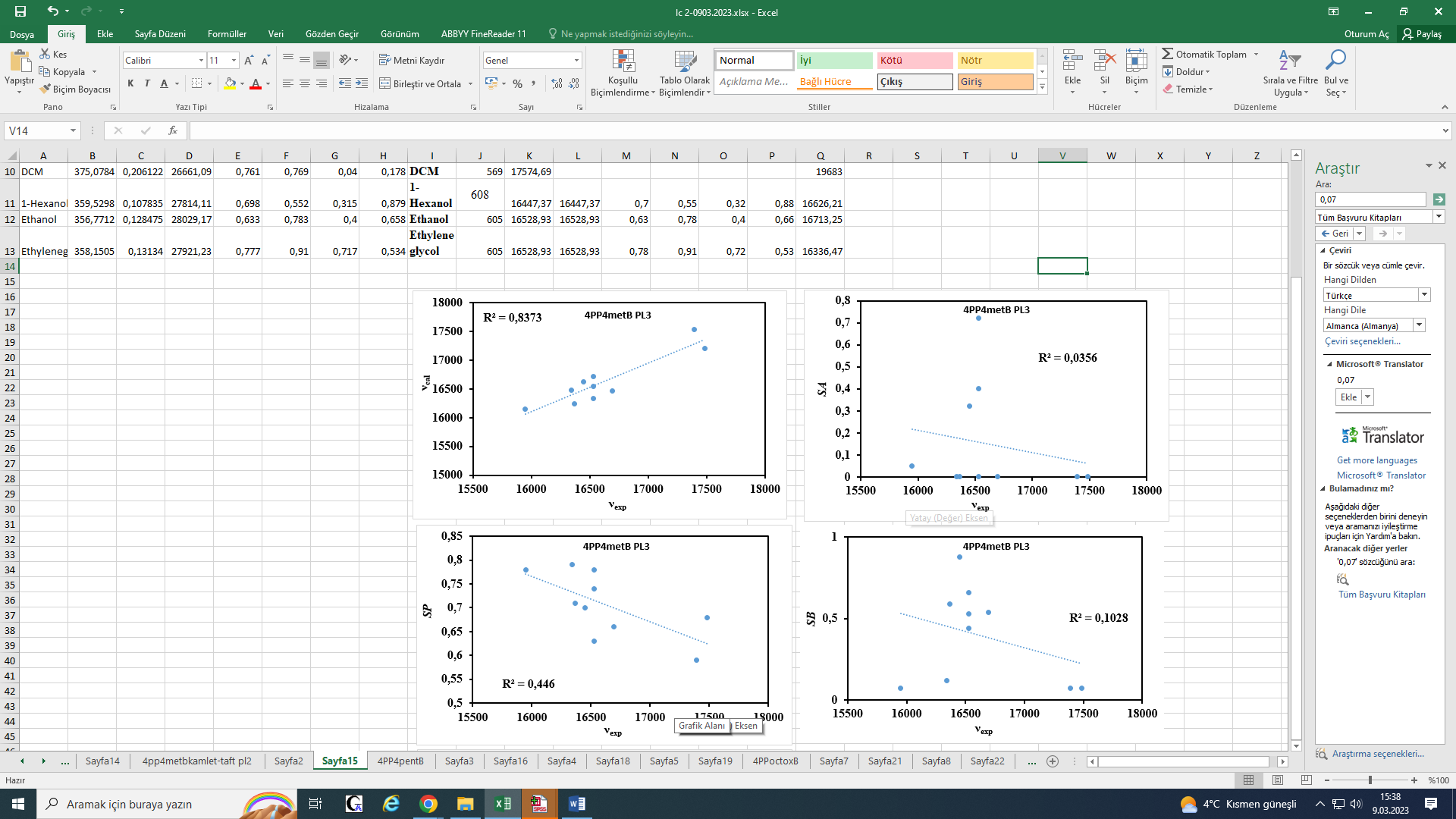

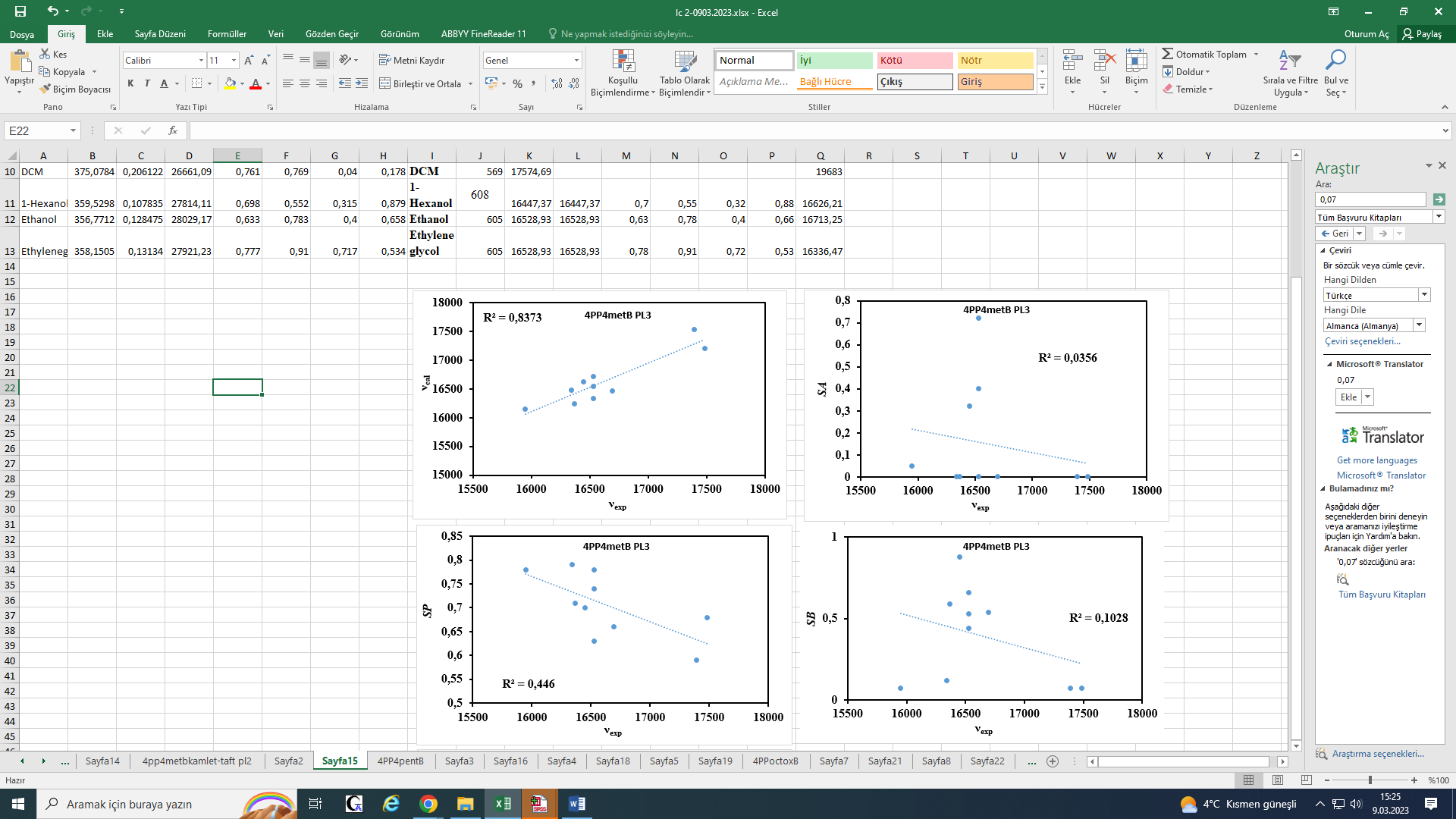

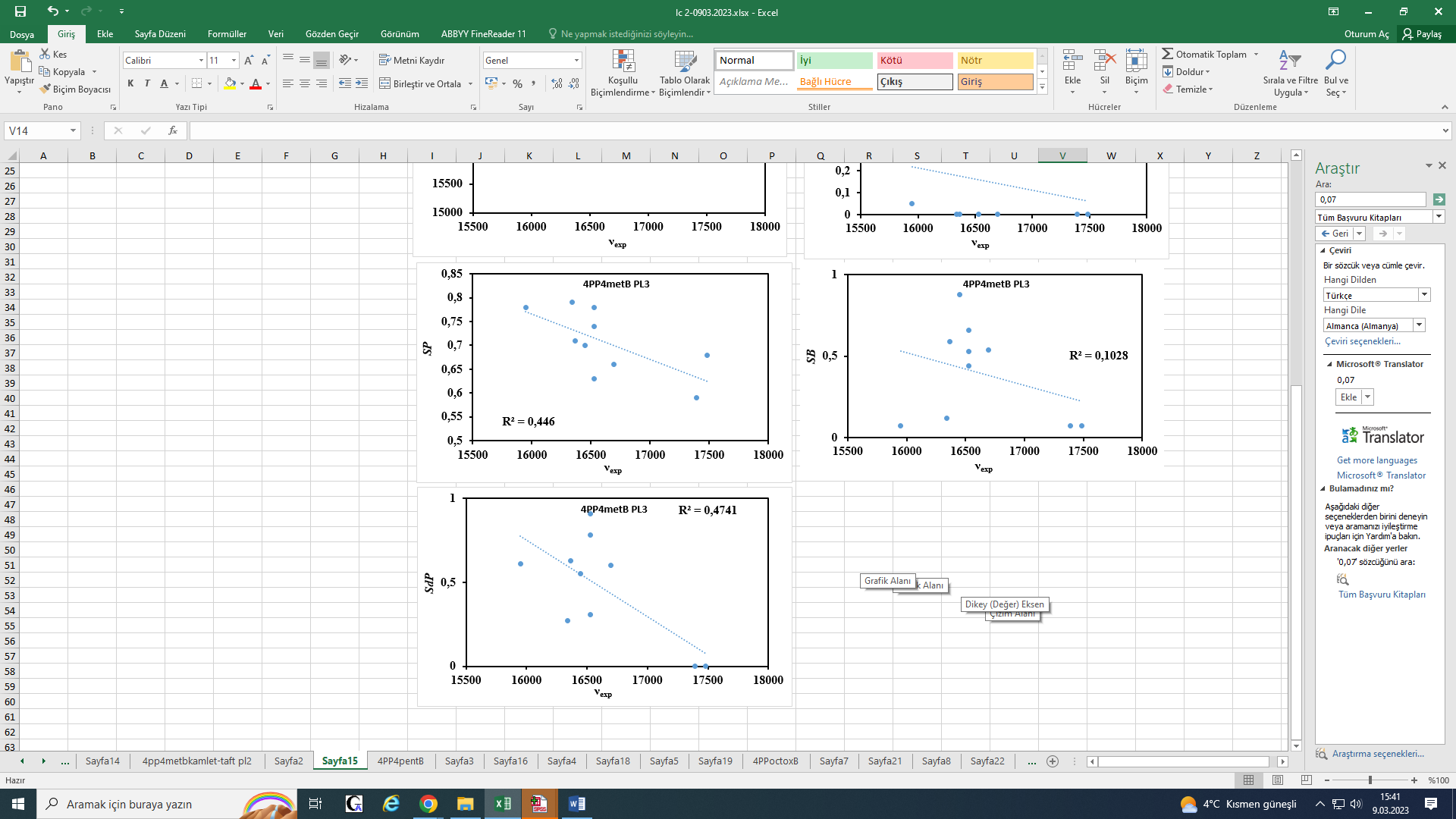

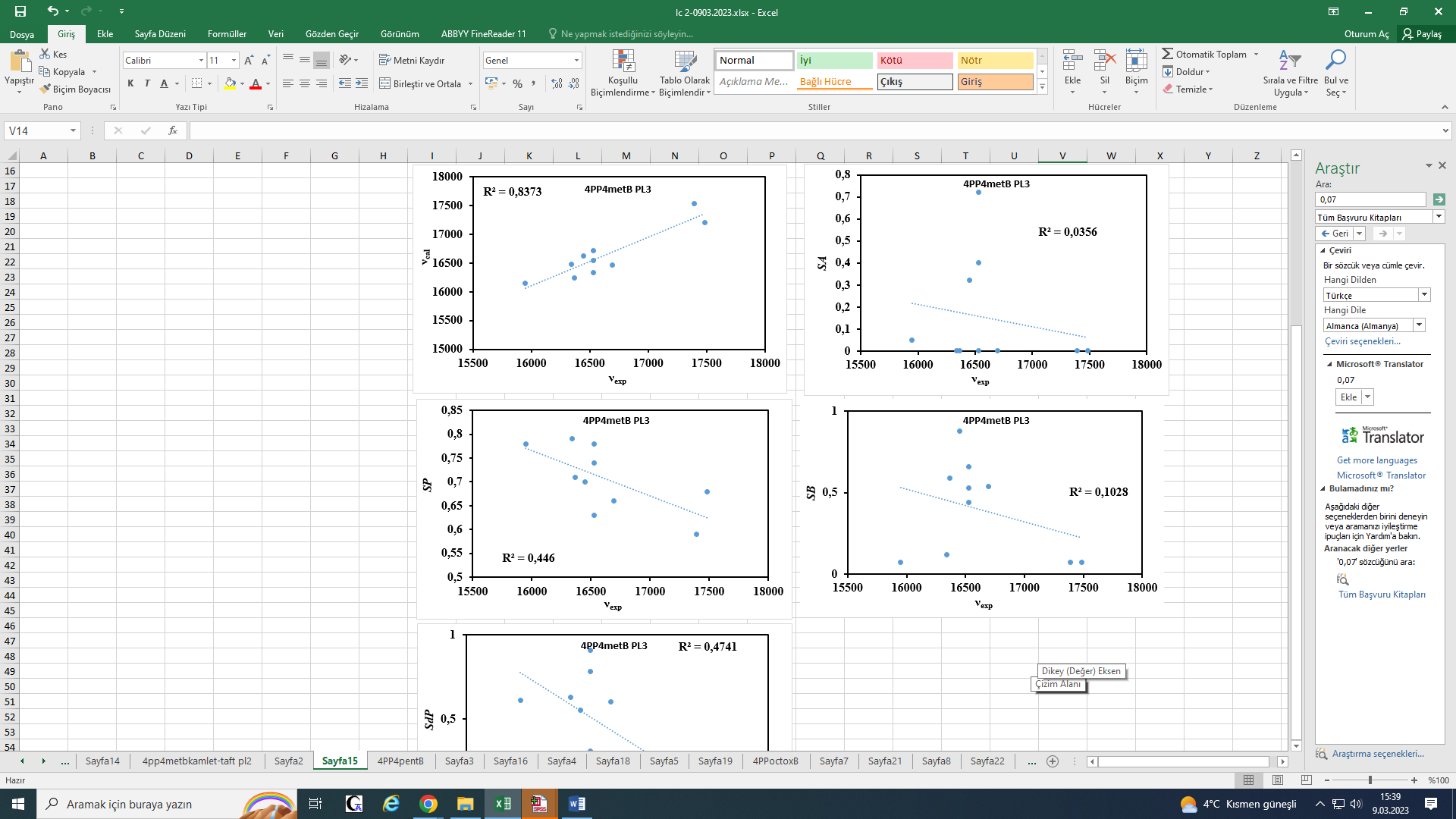

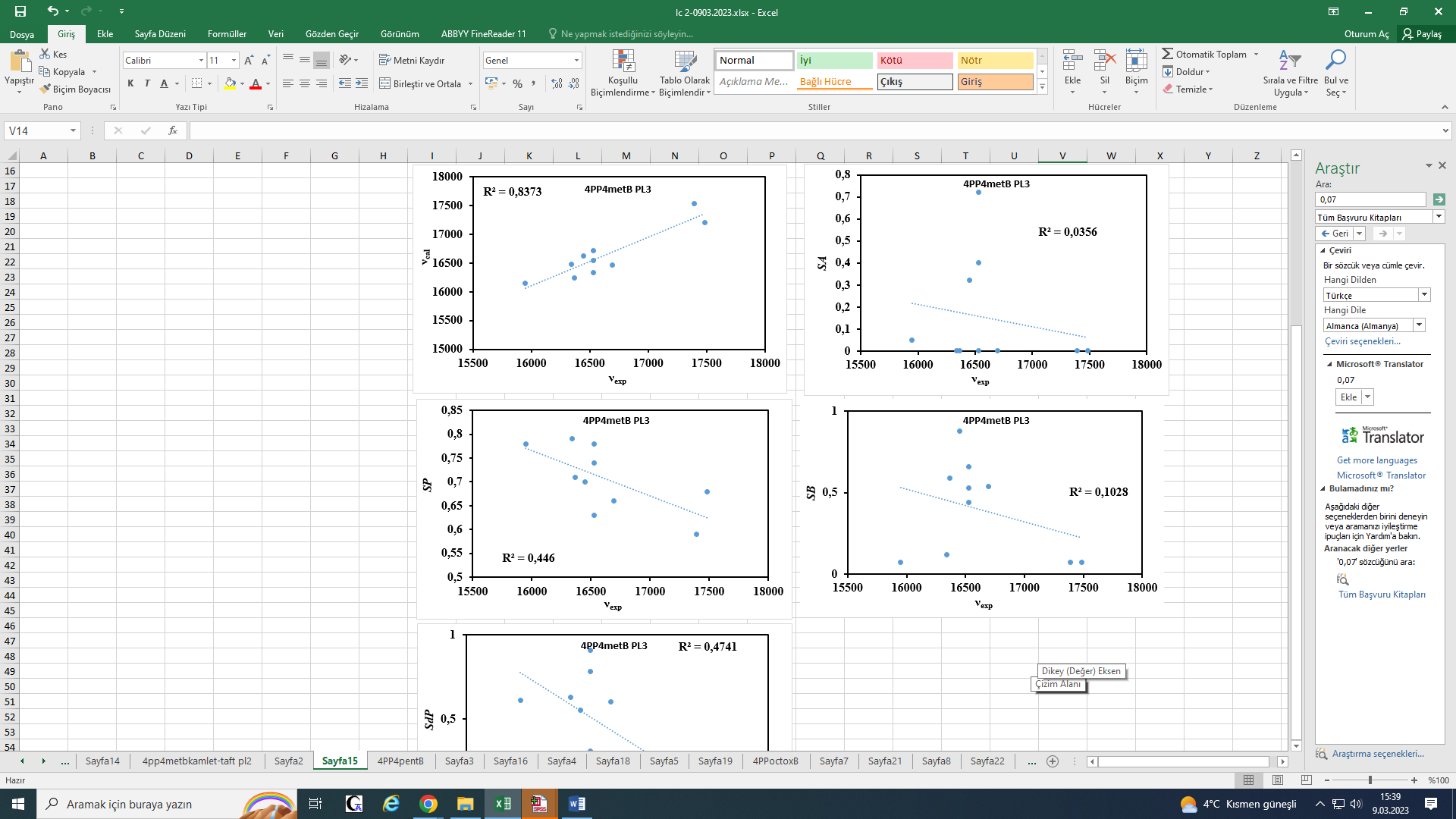


**Figure 9S.** The correlations of *ν_cal_, SP, SdP, SA* and *SB* versus ν_exp_ of λ_PL3_ wavelength of 4PP4metB molecule.


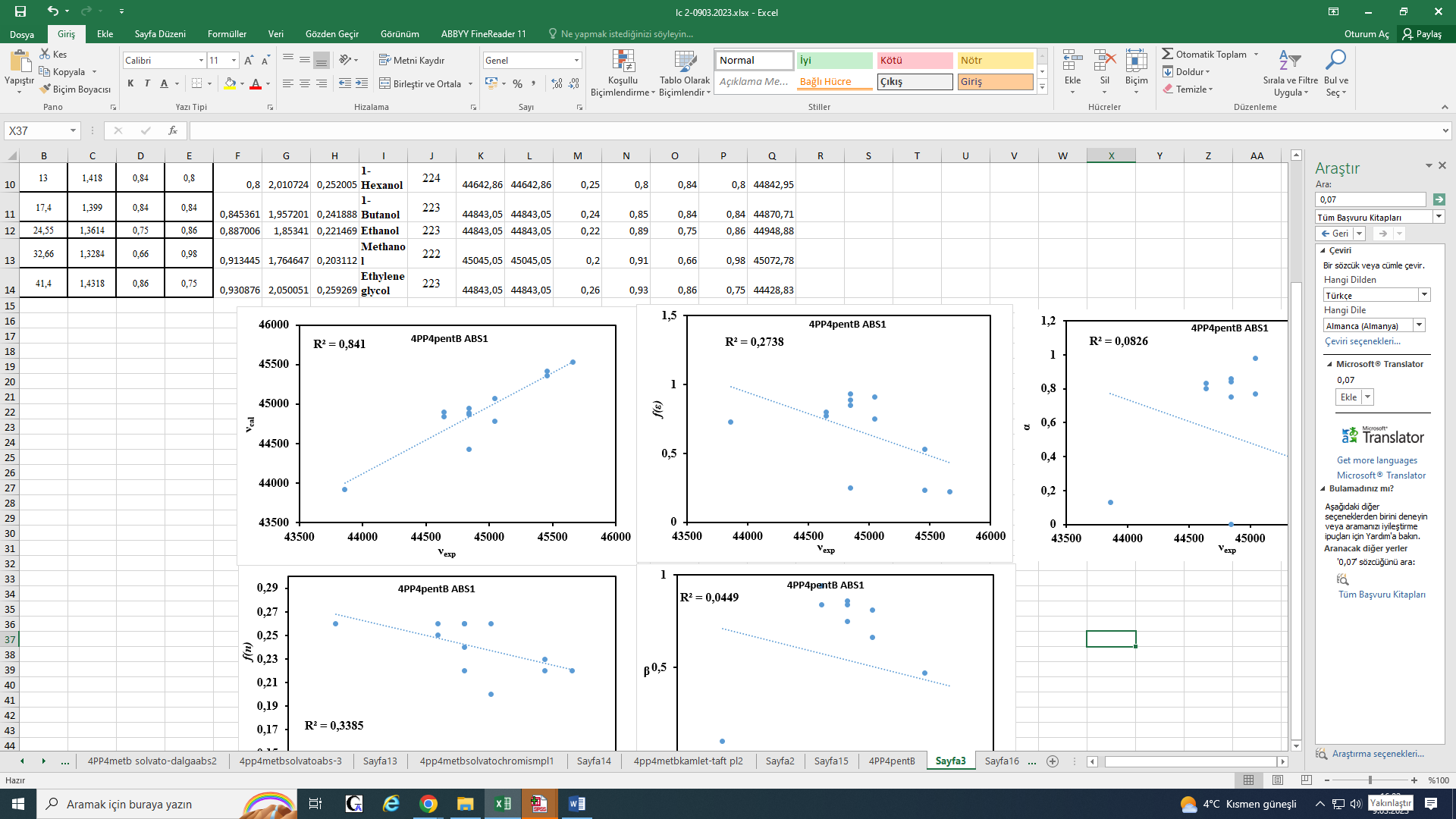

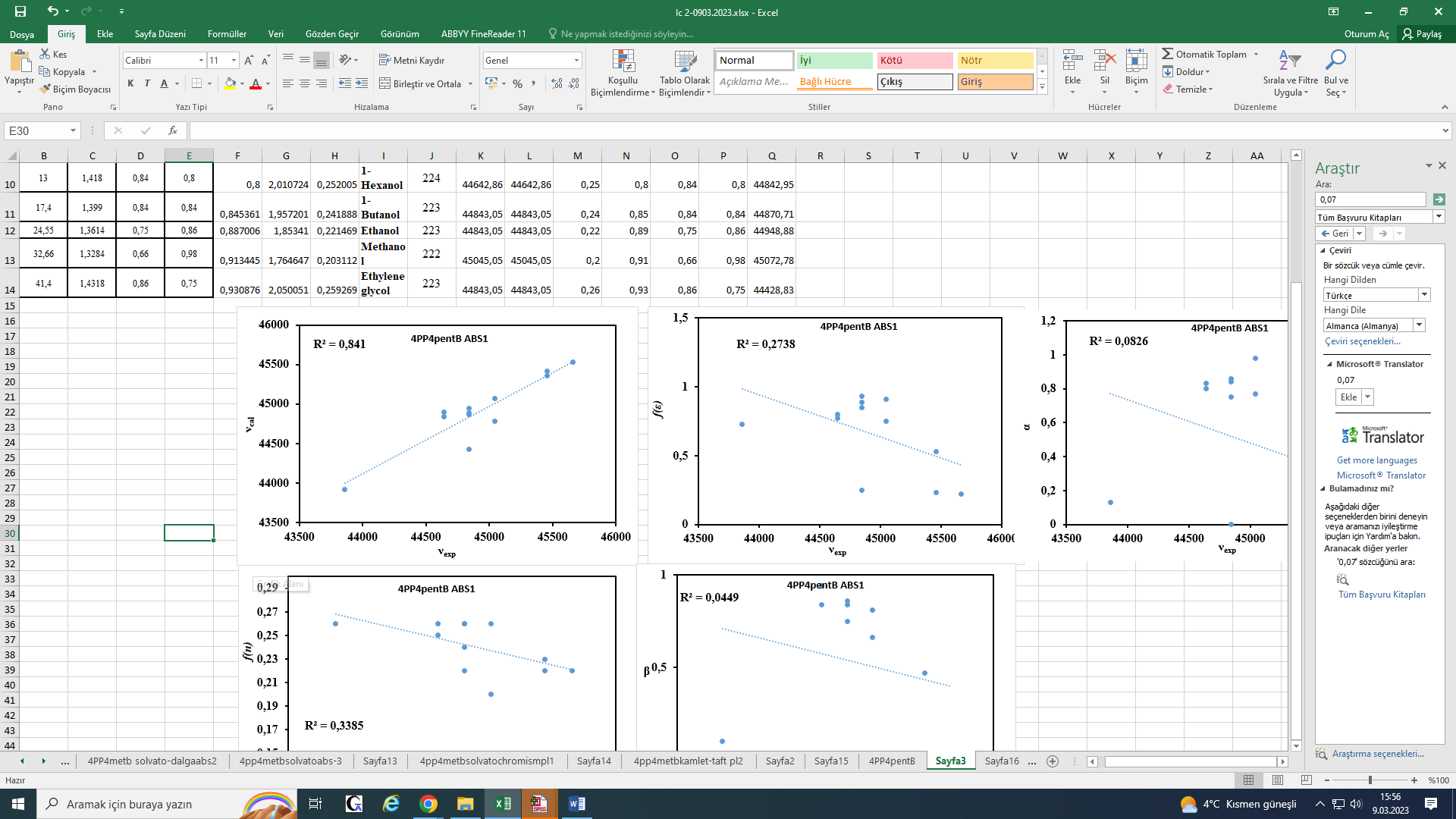

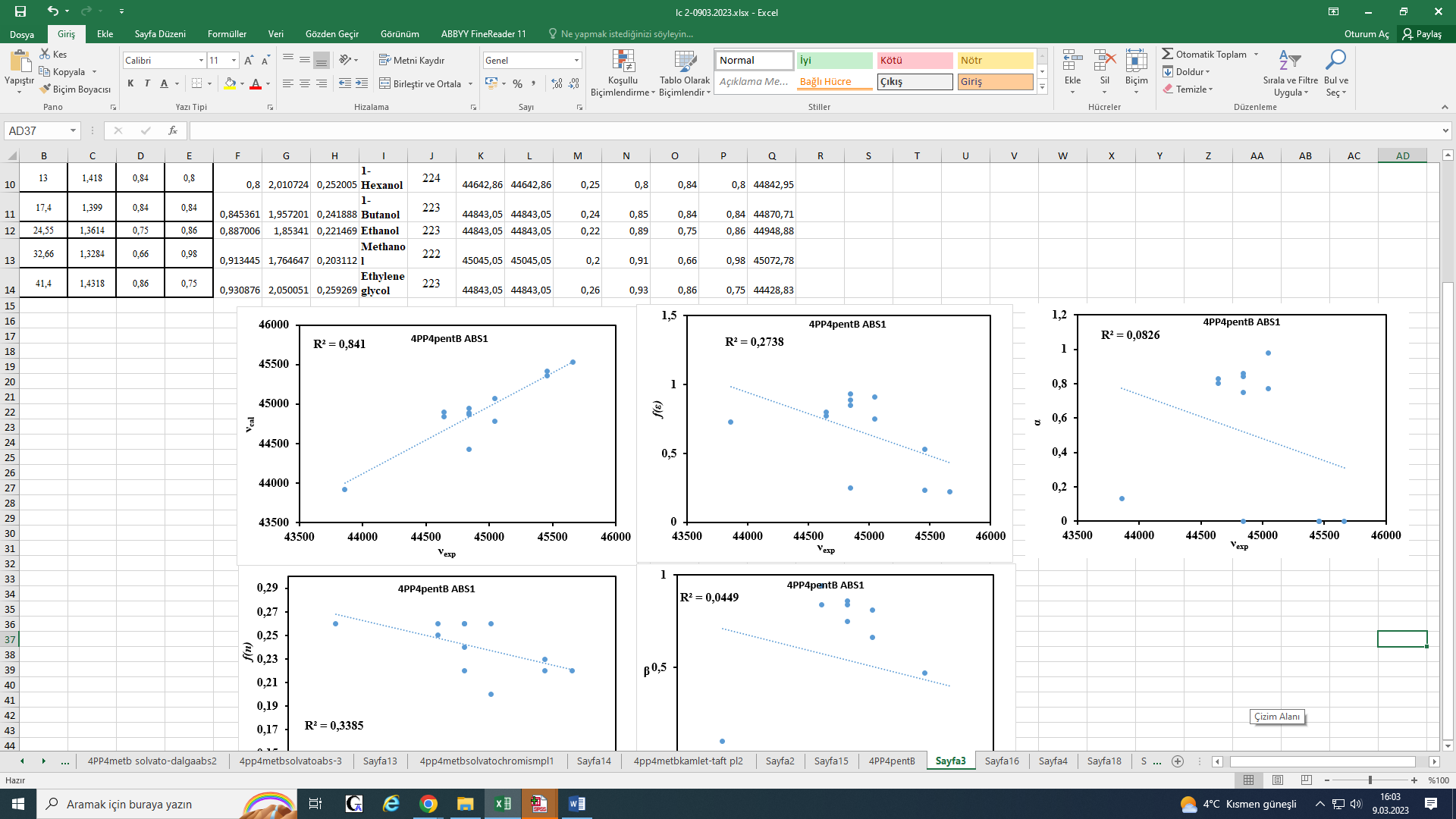

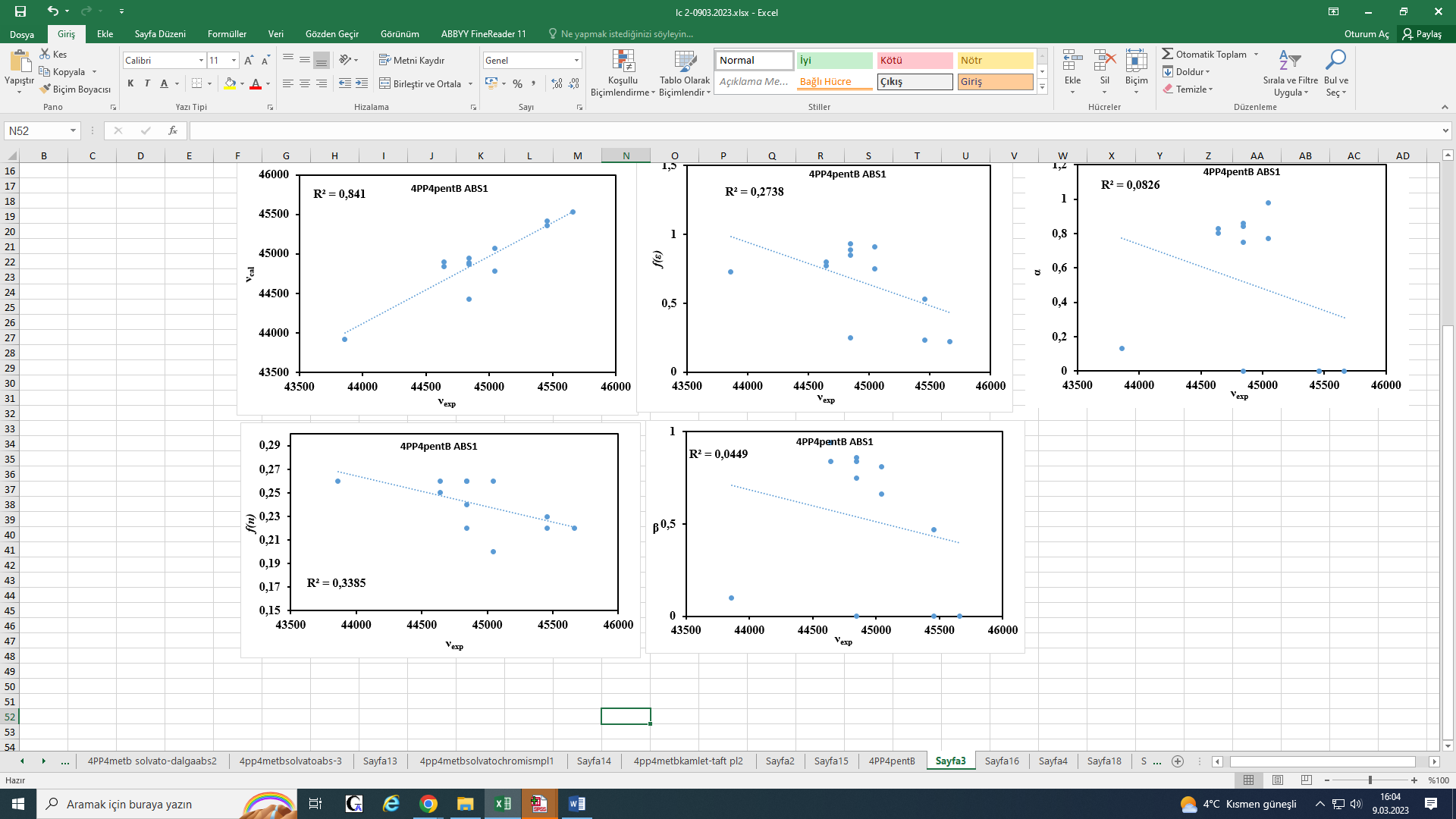

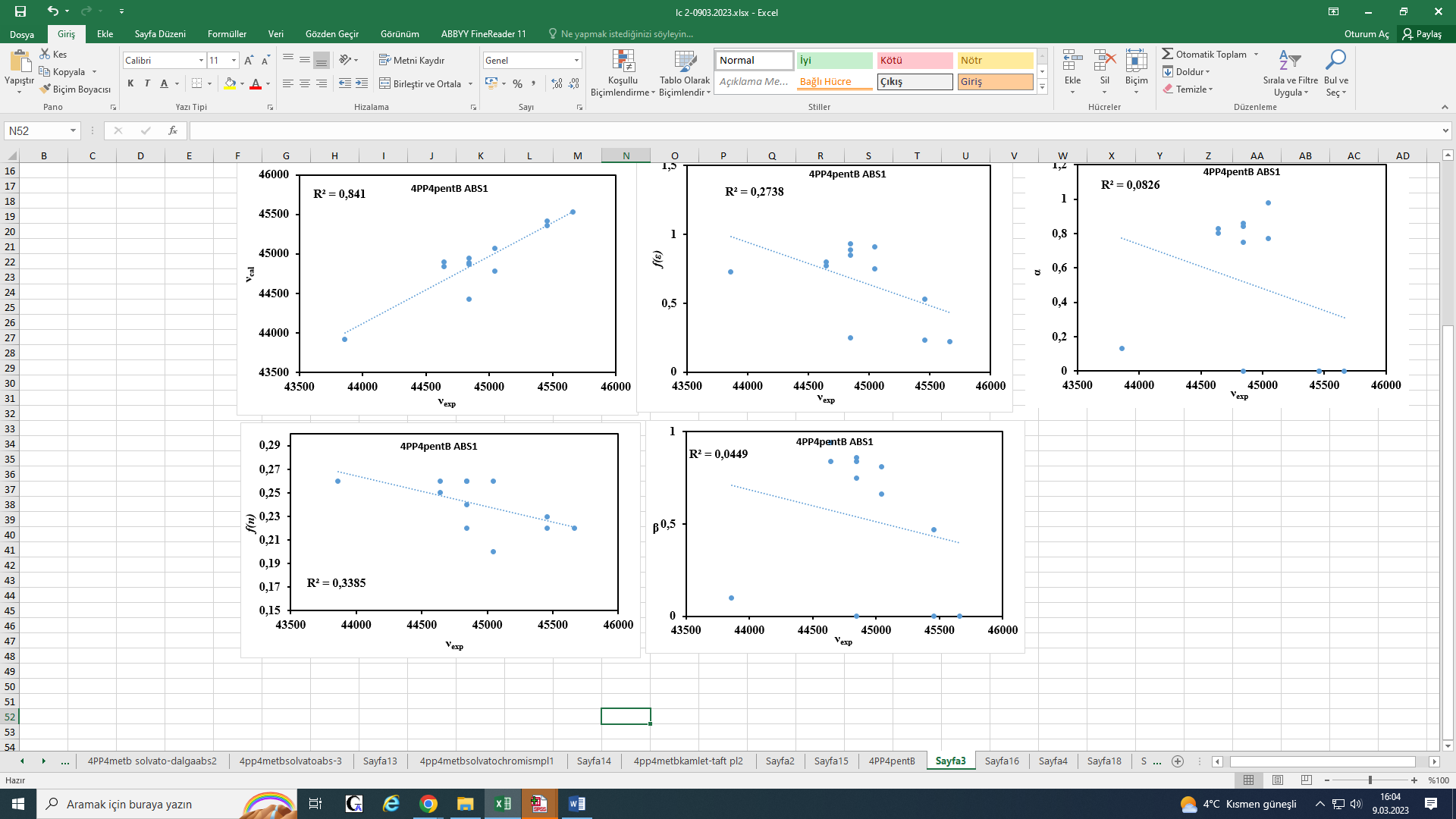


**Figure 10S.** The correlations of *ν_cal_, β,α, f(n)* and *f(ε)* versus ν_exp_  of λ_ABS1_ wavelength of 4PP4pentB molecule.


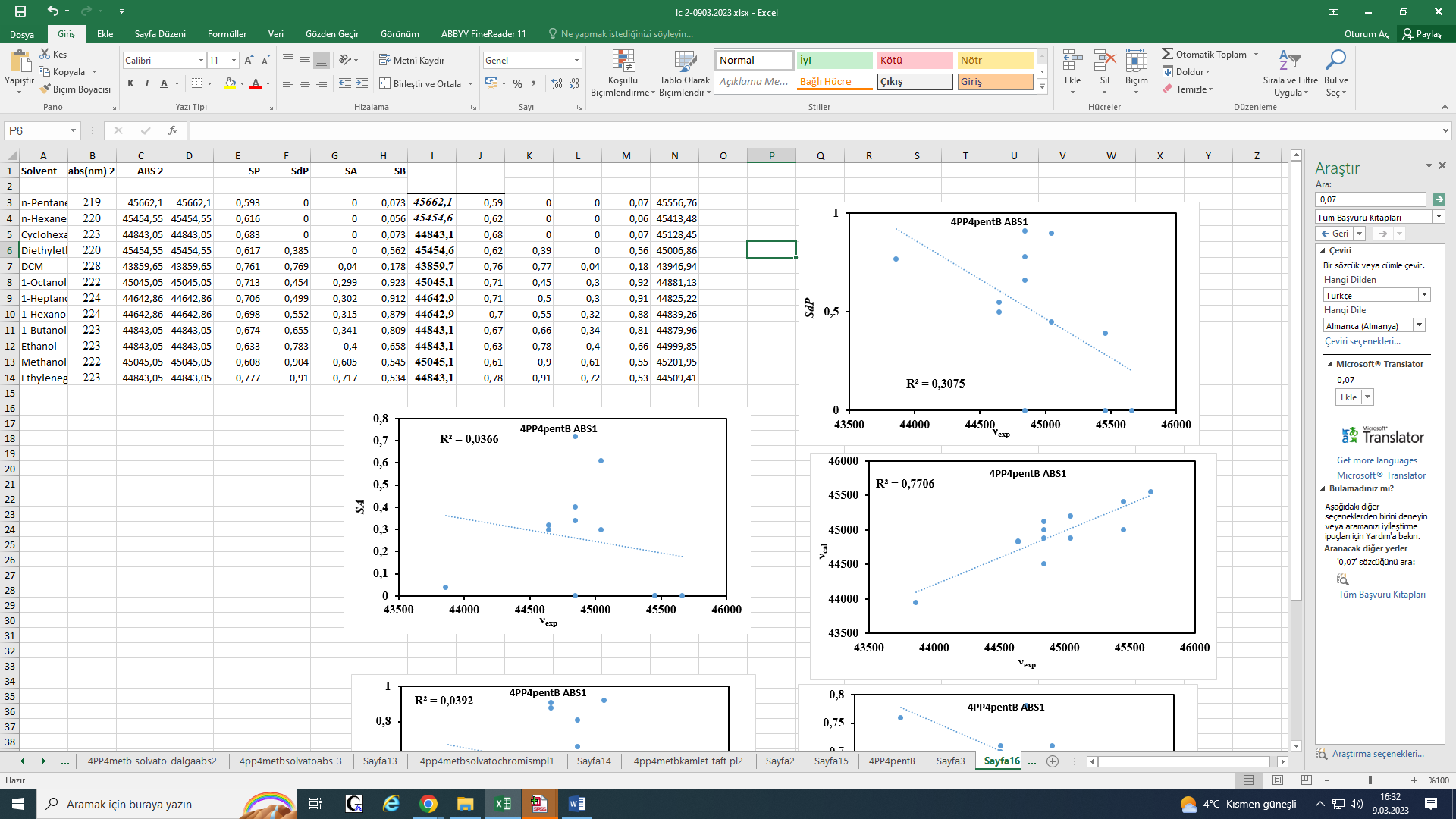

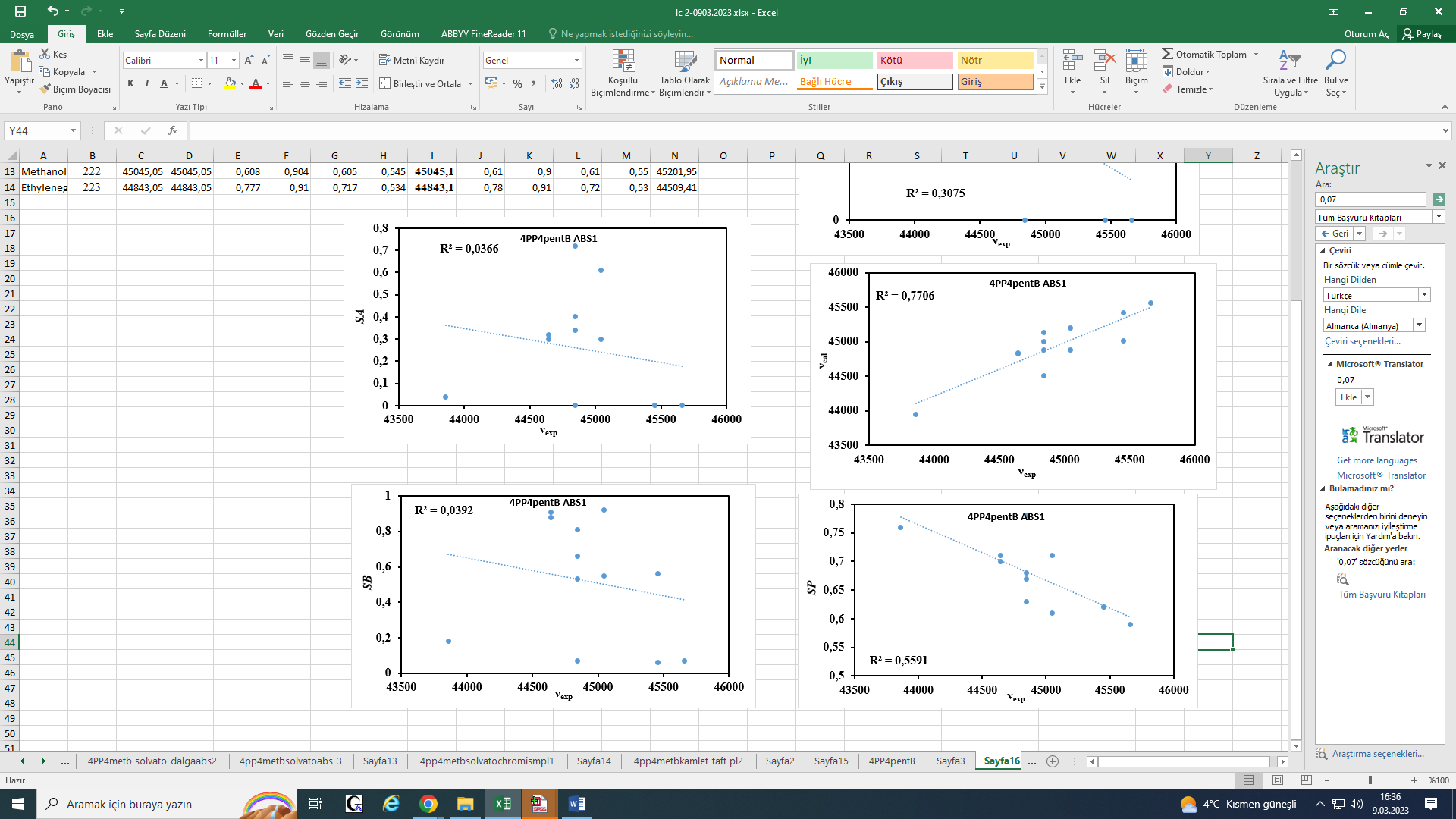

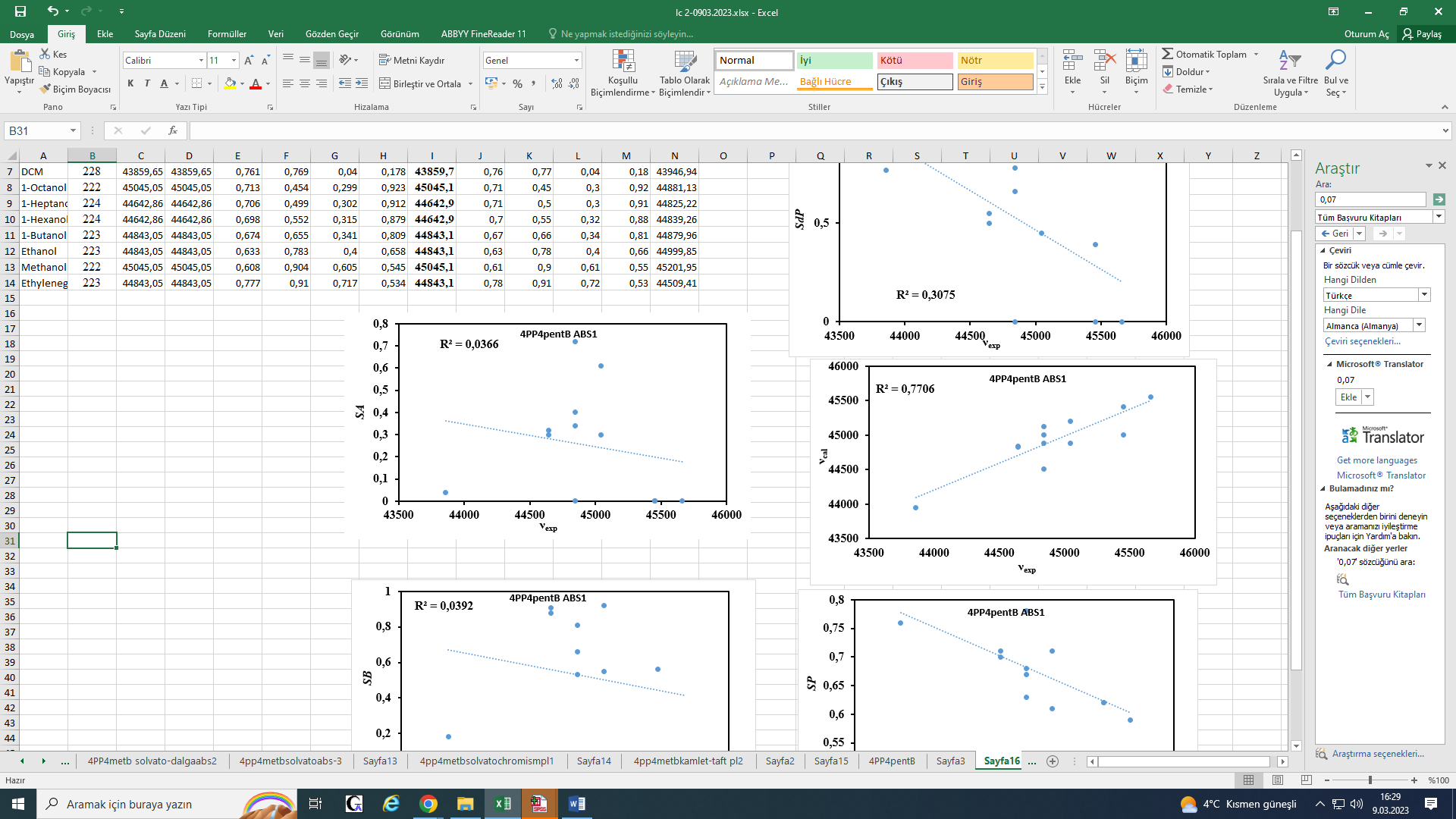

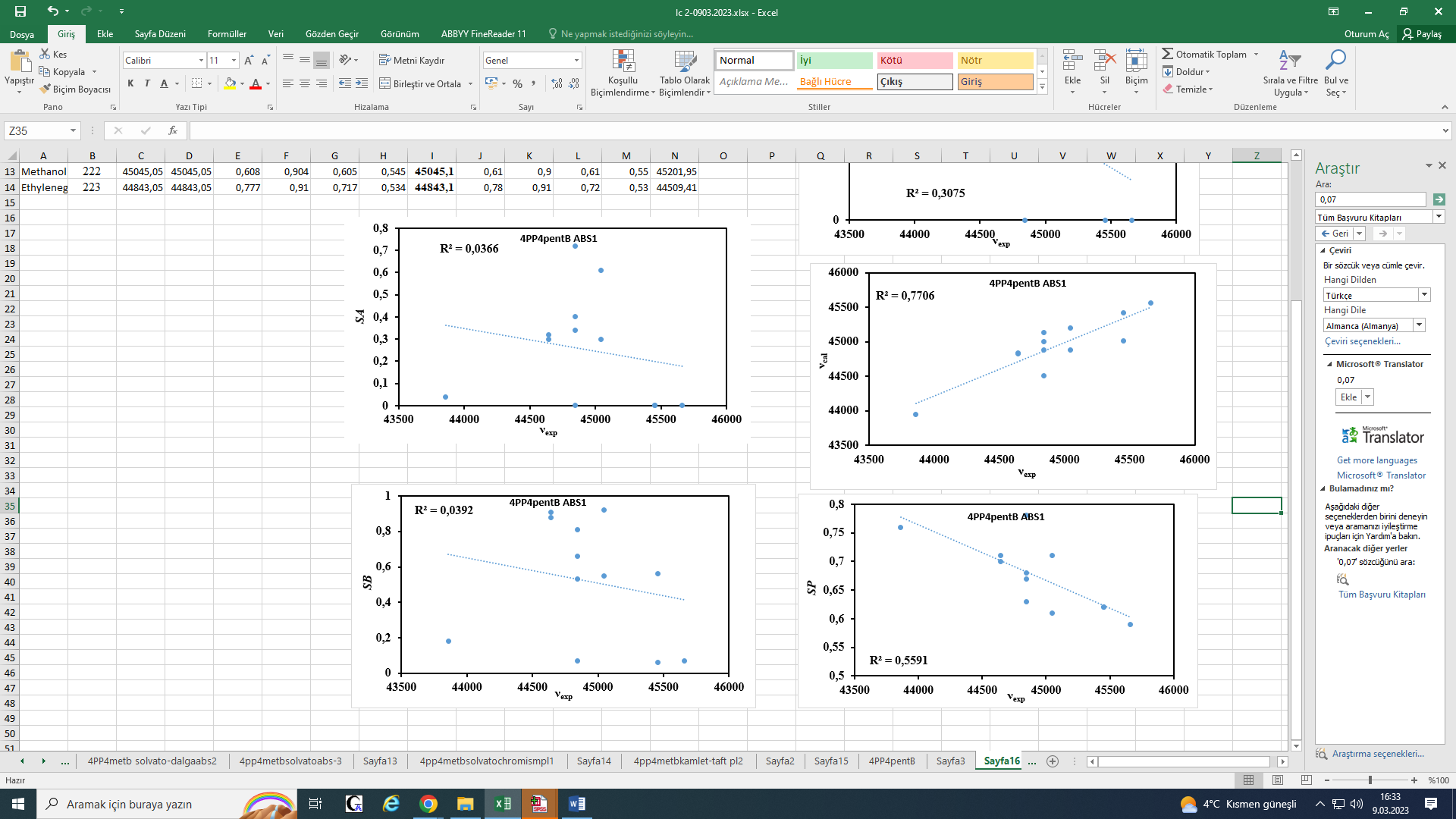

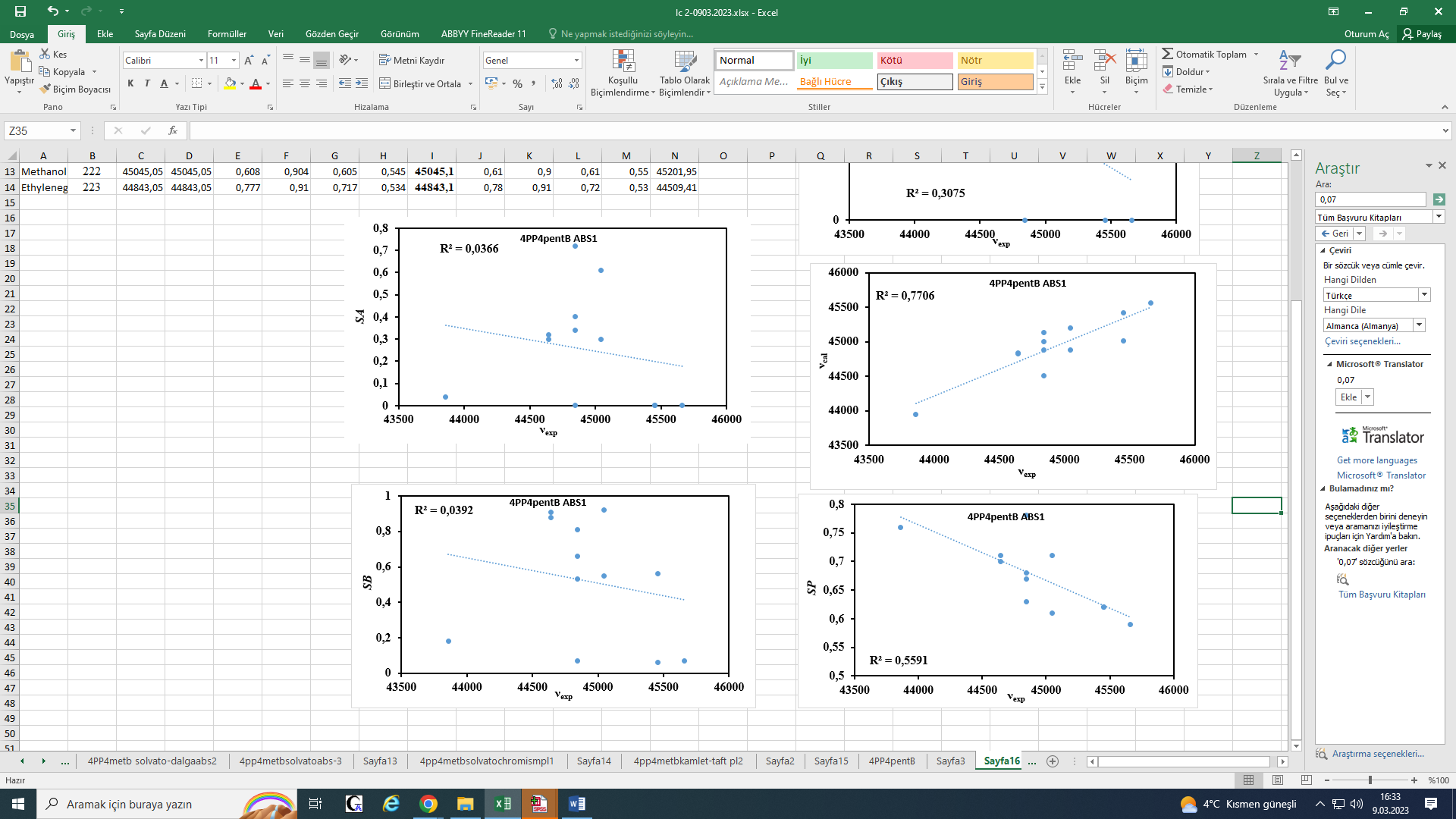


**Figure 11S.** The correlations of *ν_cal_, SP, SdP, SA* and *SB* versus ν_exp_ of λ_ABS1_ wavelength of 4PP4pentB molecule.


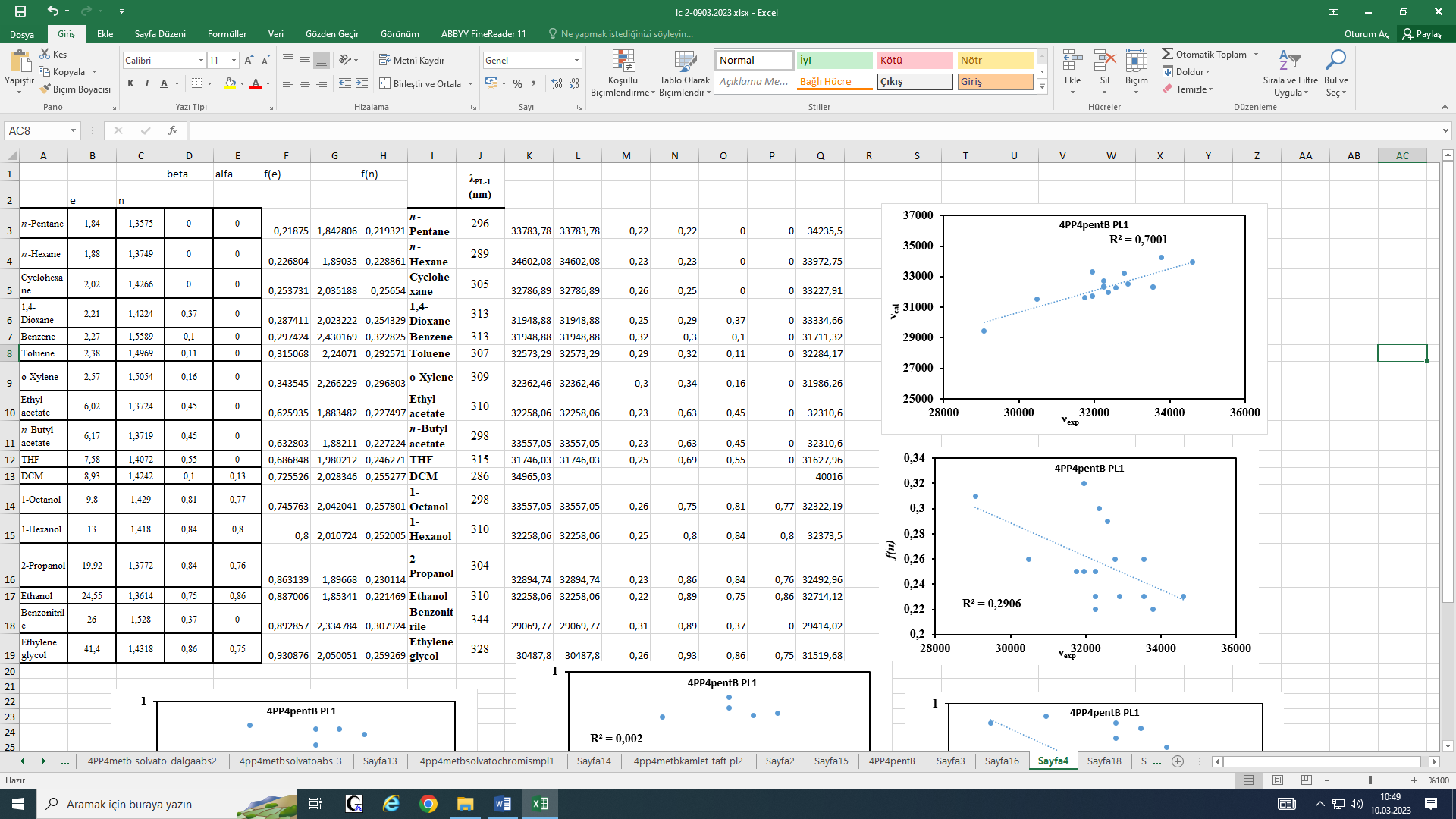

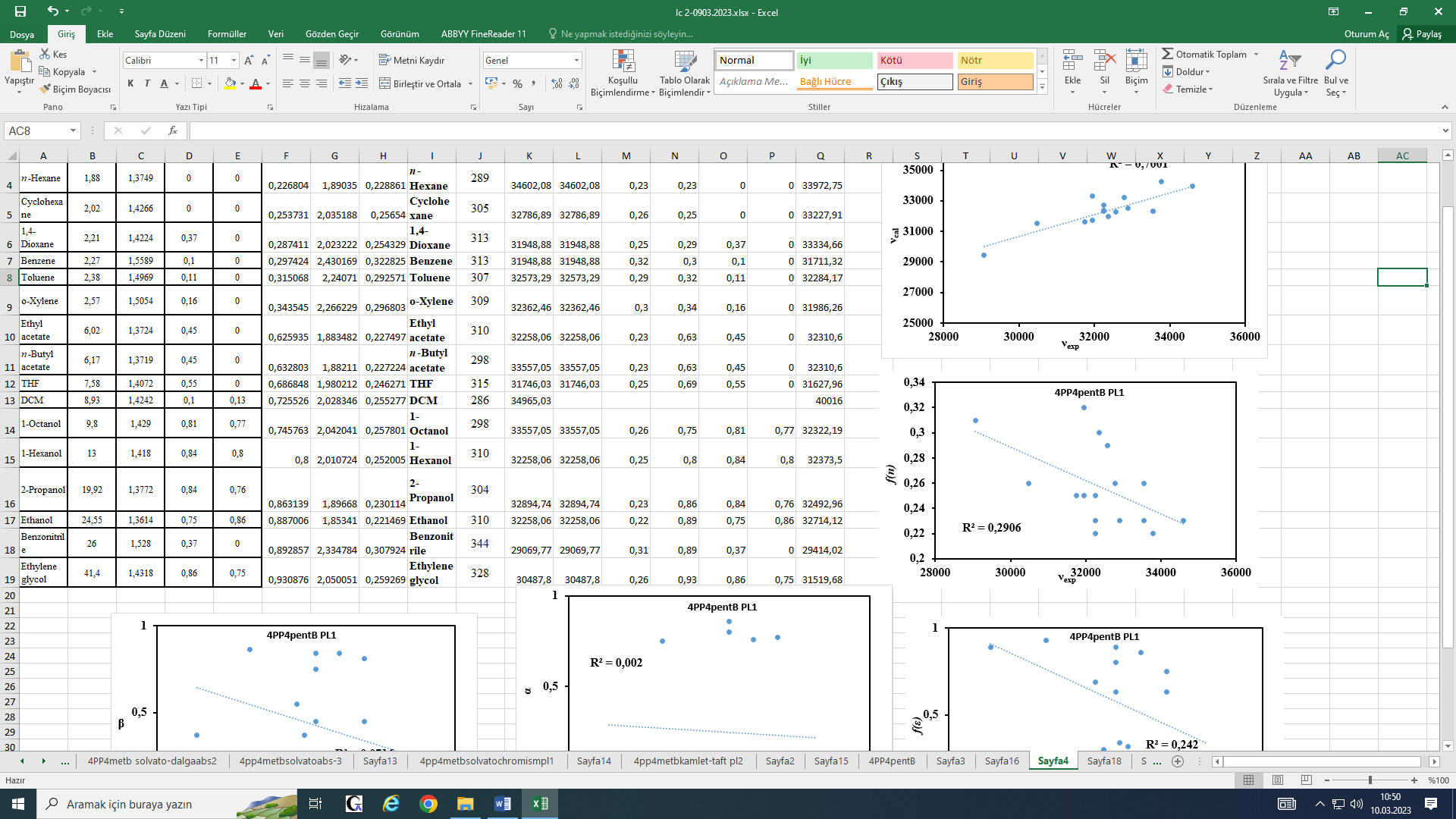

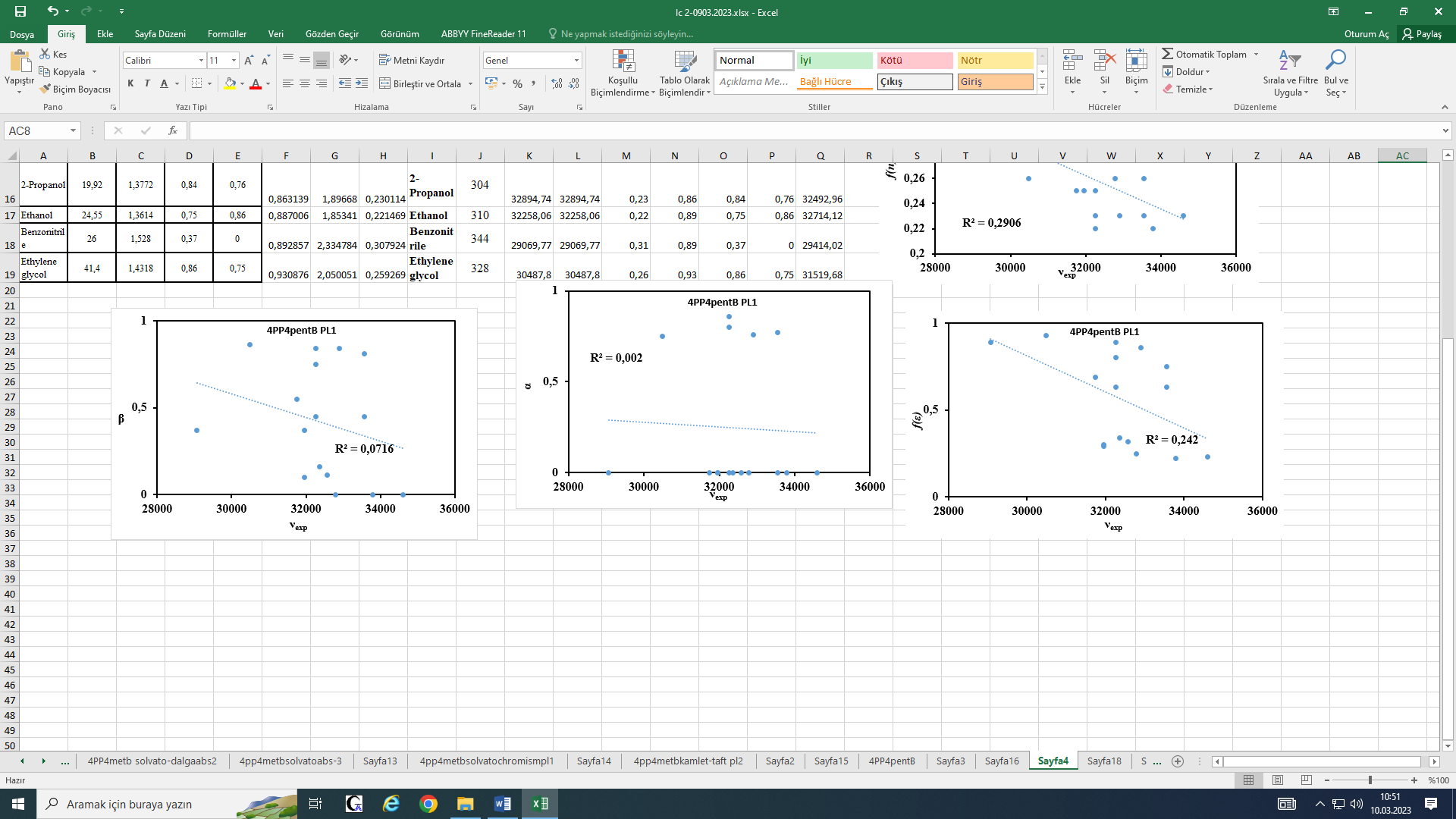

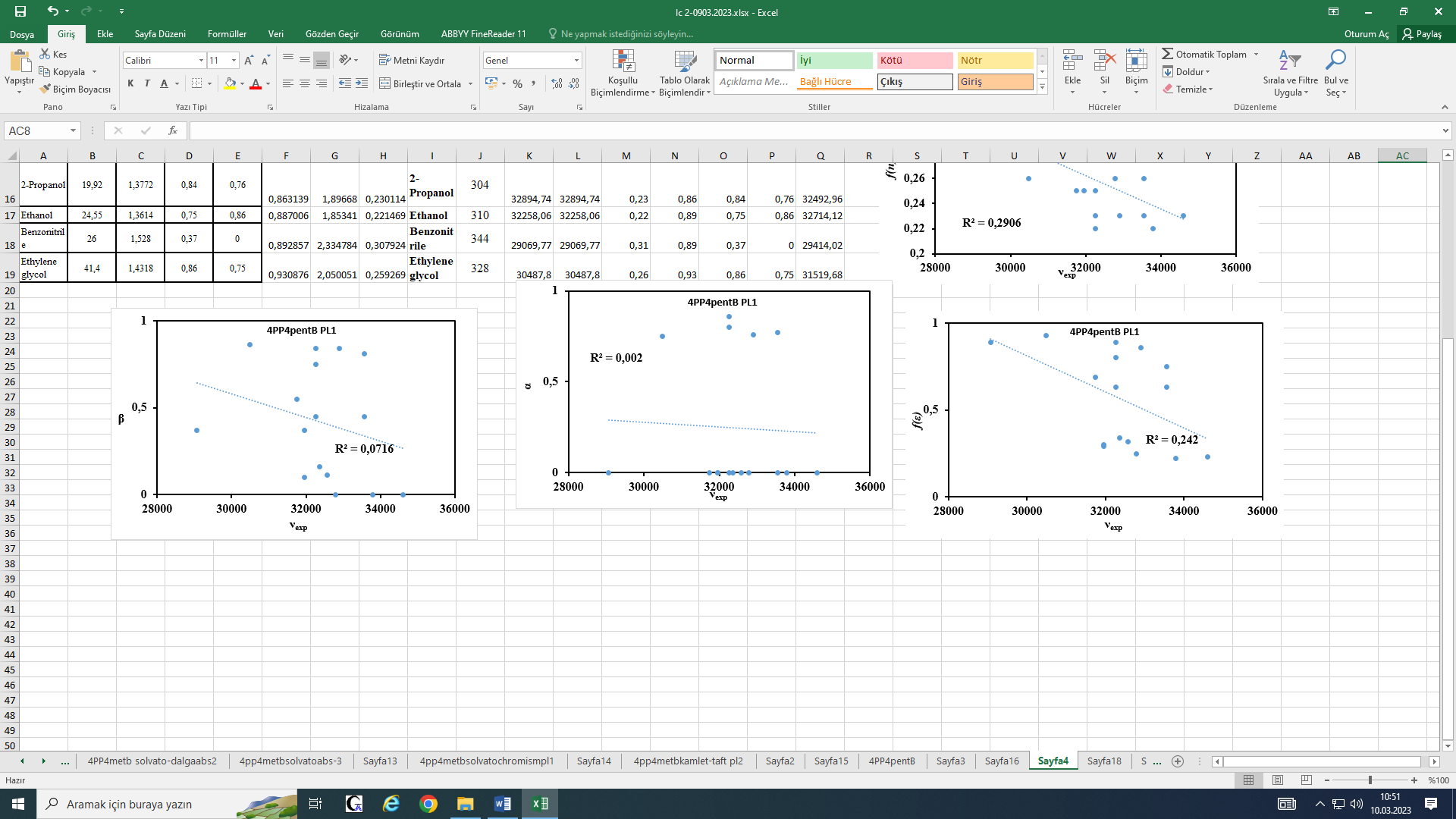

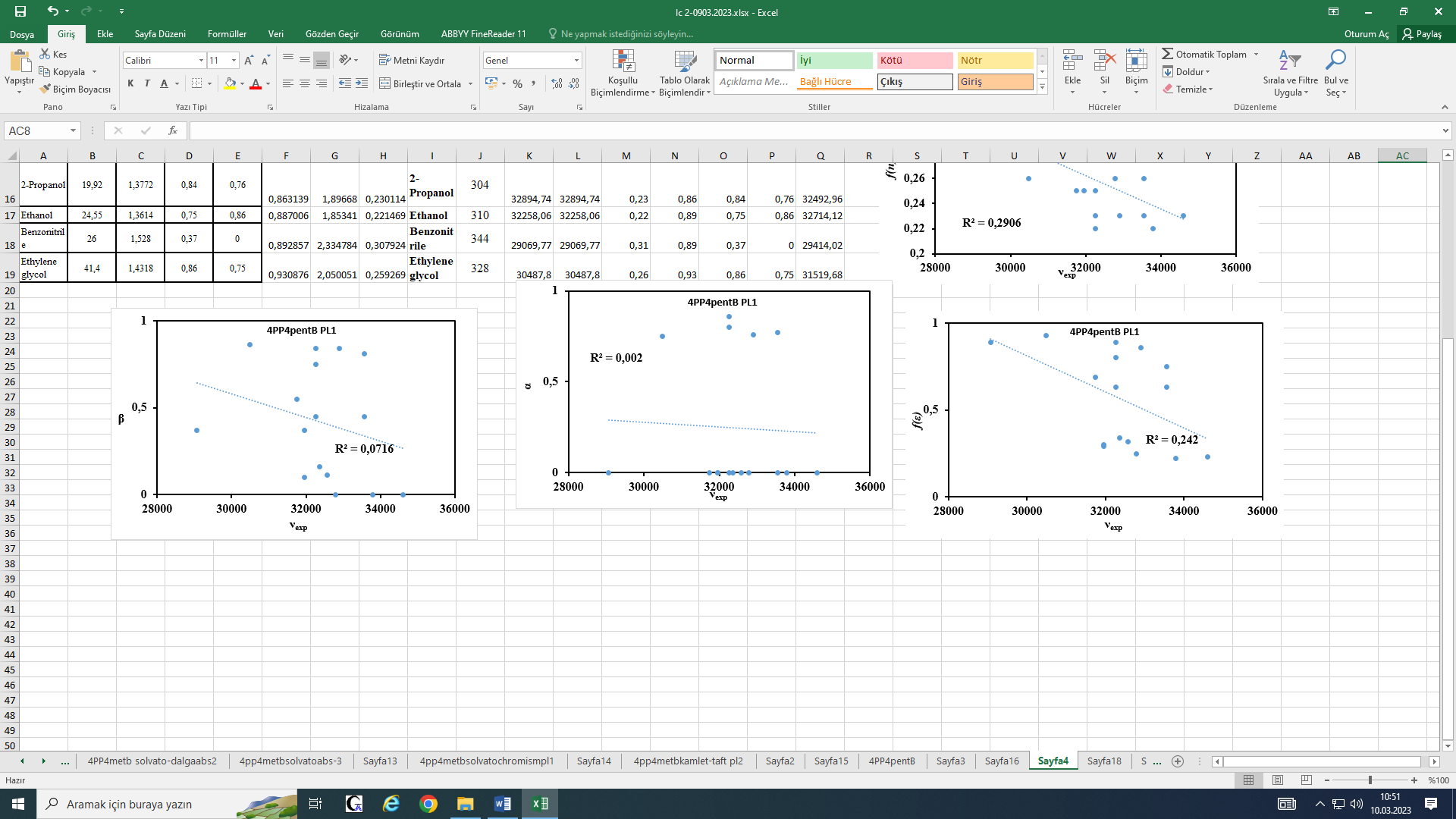


**Figure 12S.** The correlations of *ν_cal_, β, α, f(n)* and *f(ε)* versus ν_exp_ of λ_PL1_ wavelength of 4PP4pentB molecule.


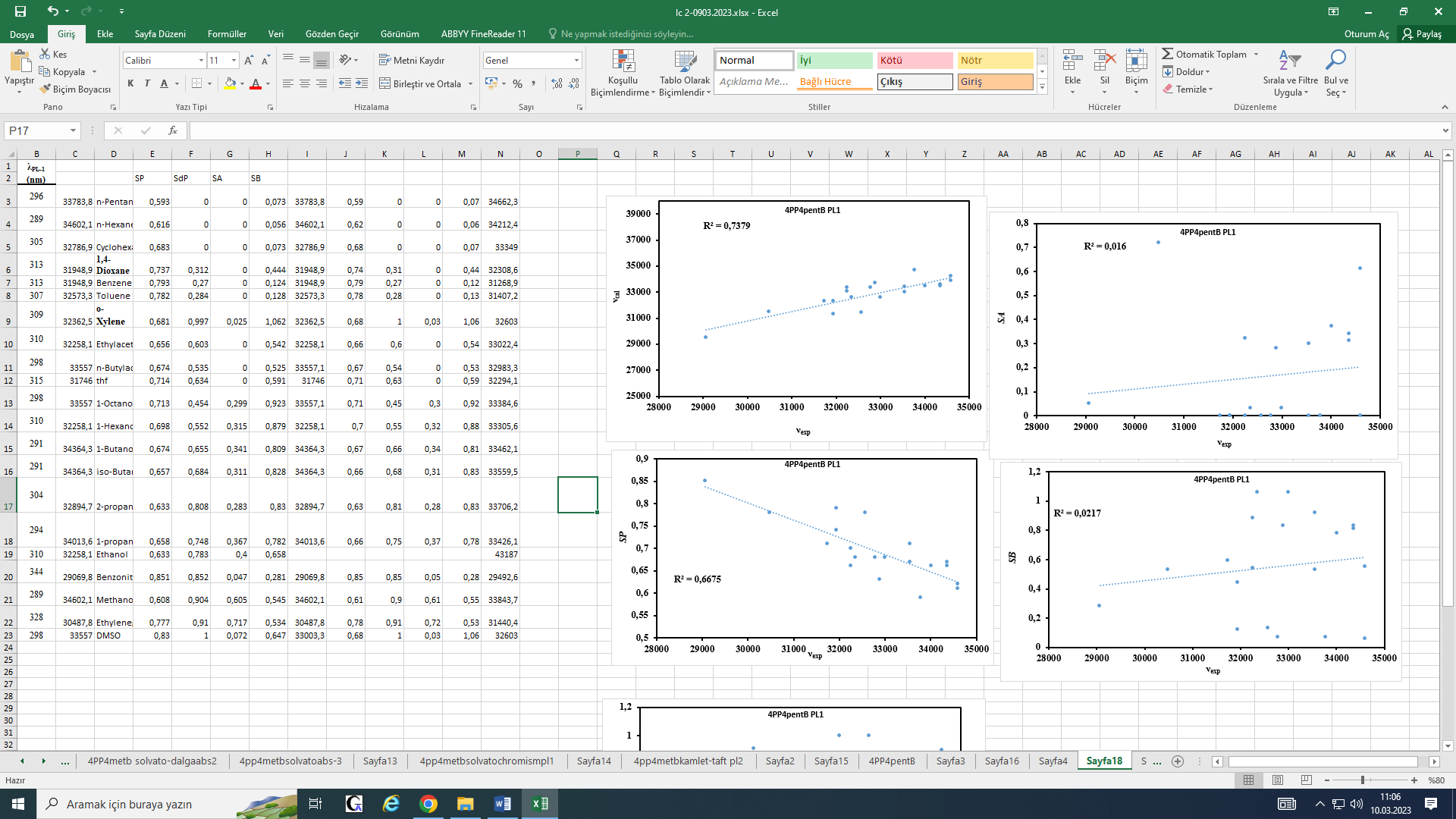

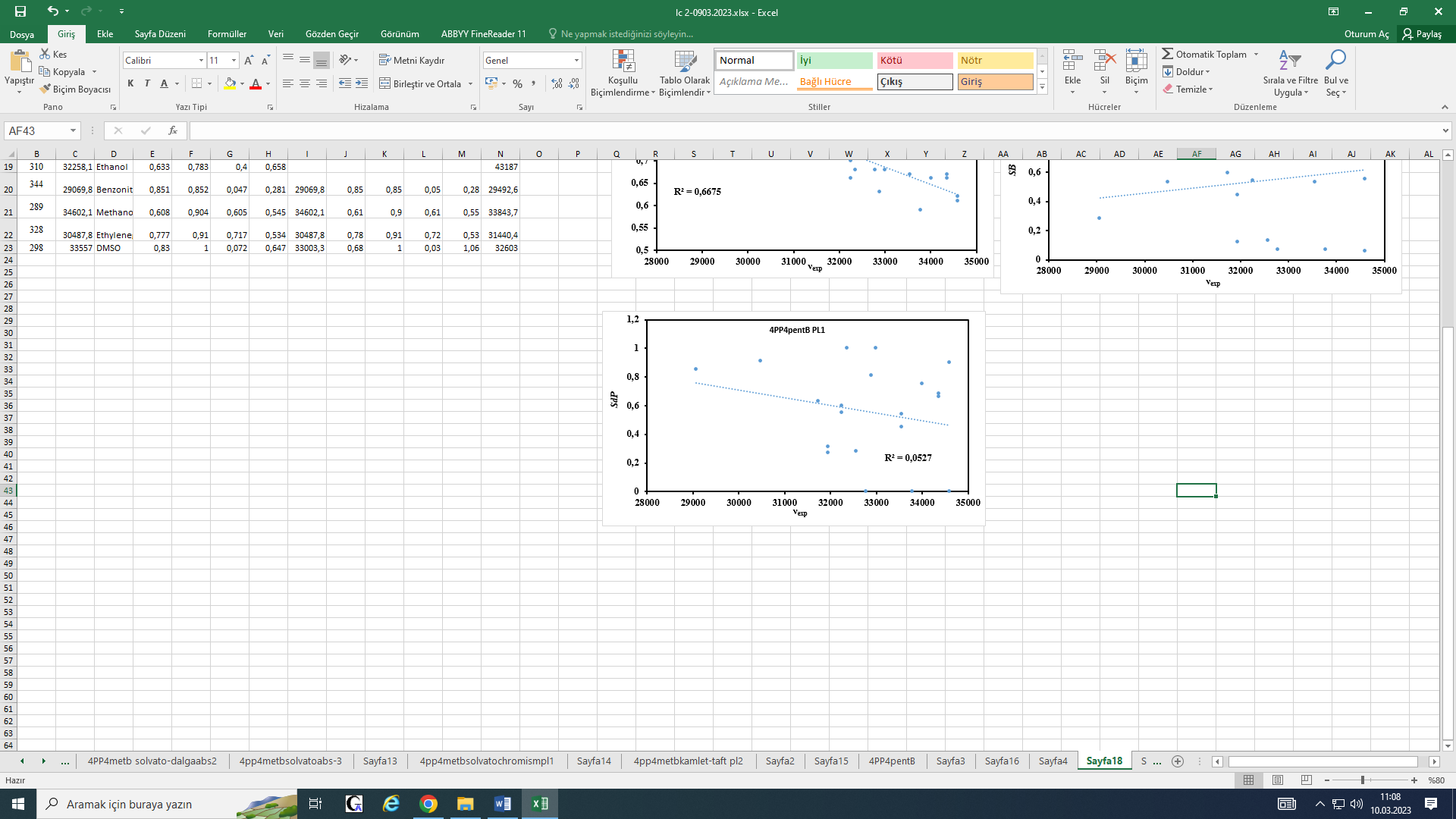

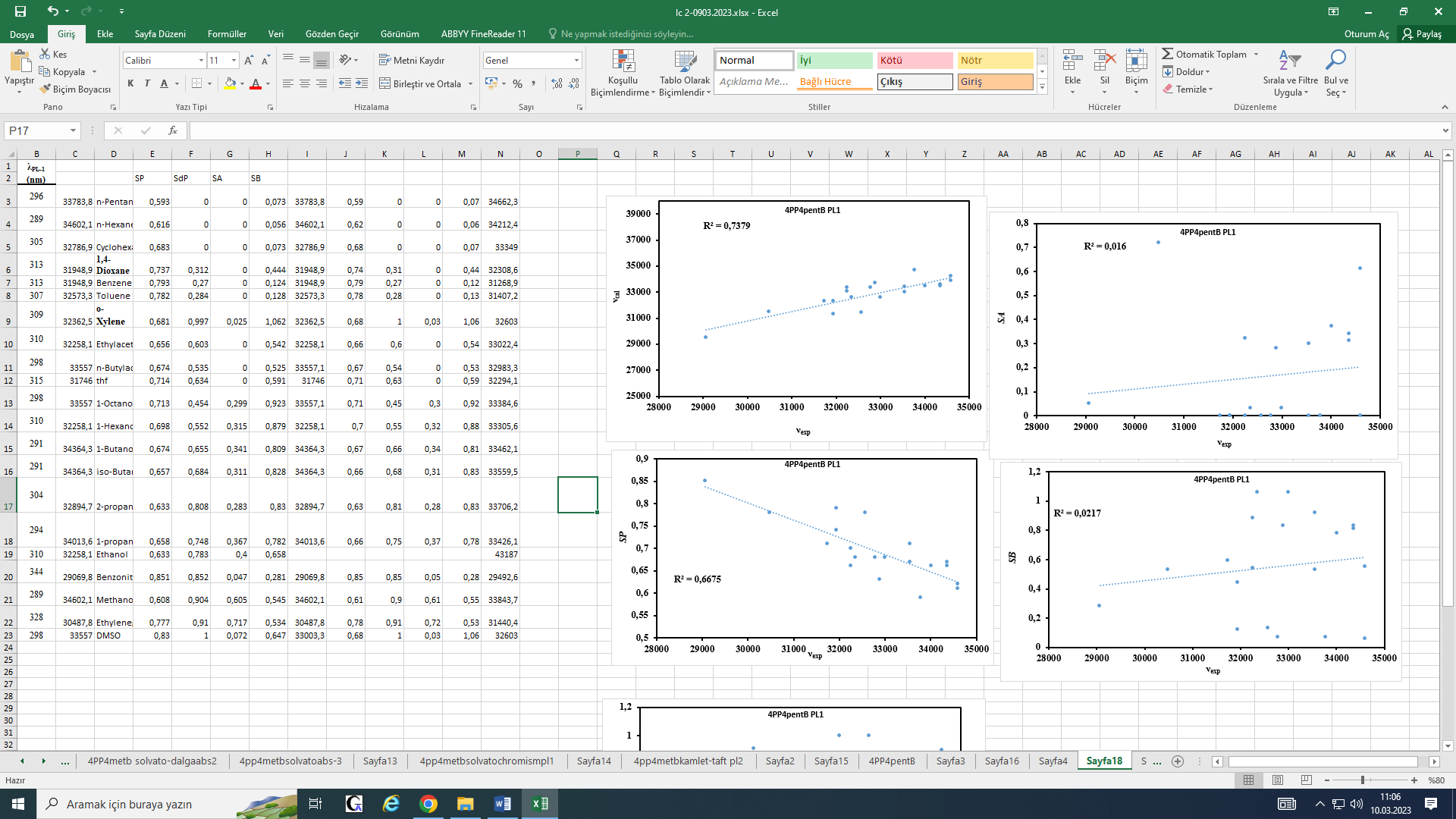

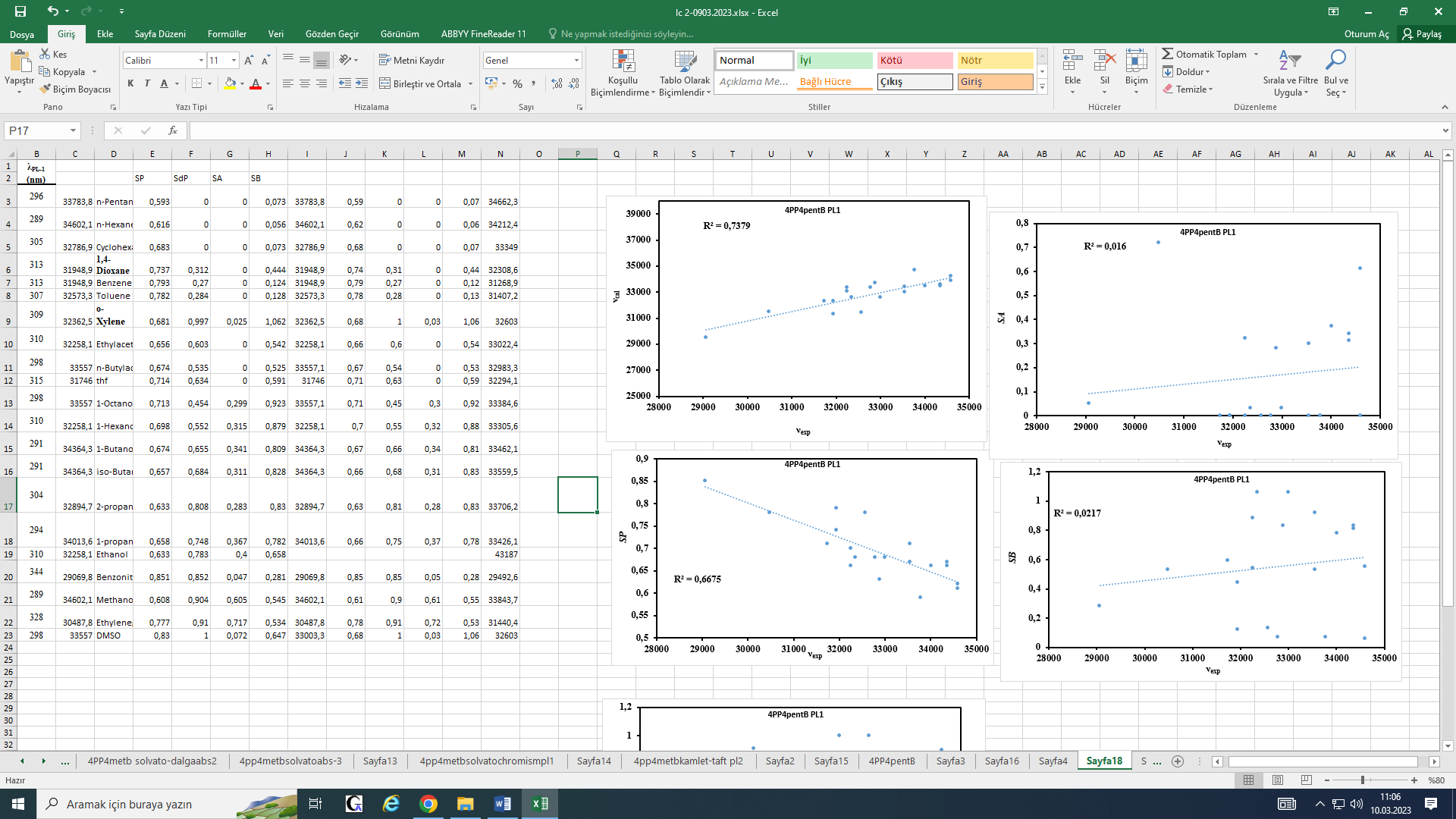

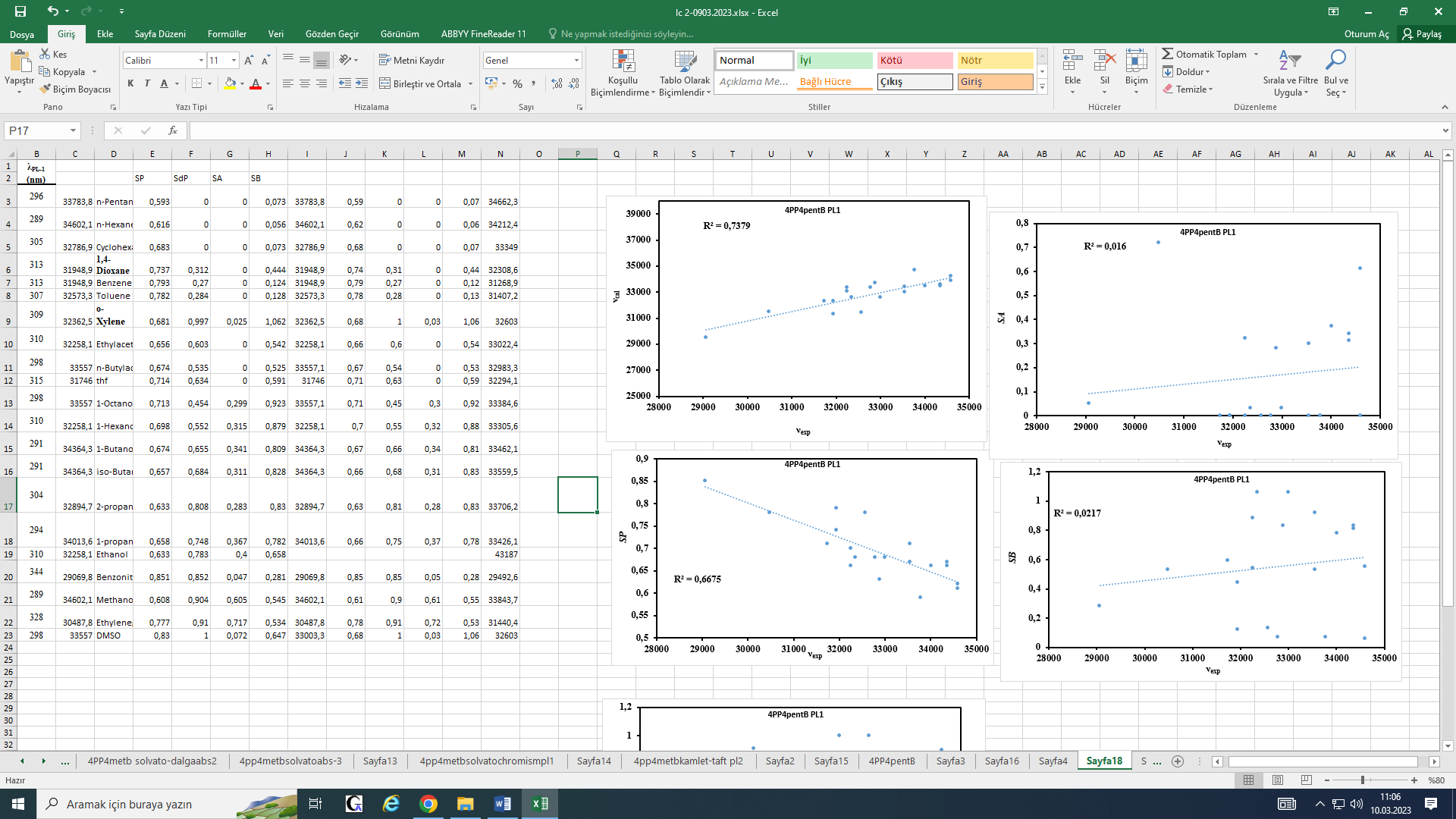


**Figure 13S.** The correlations of *ν_cal_, SP, SdP, SA and SB* versus ν_exp_ of λ_PL1_ wavelength of 4PP4pentB molecule.


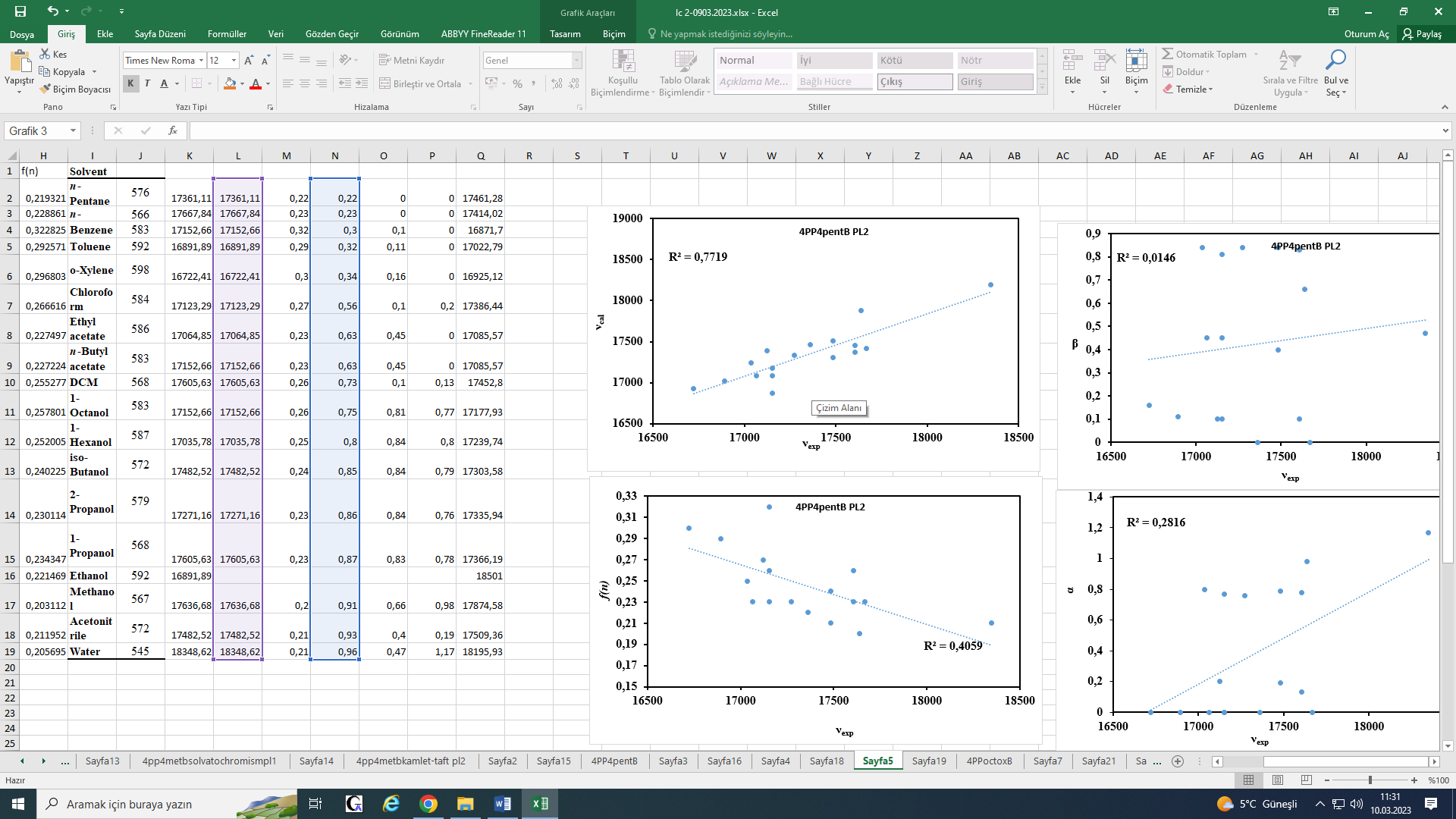

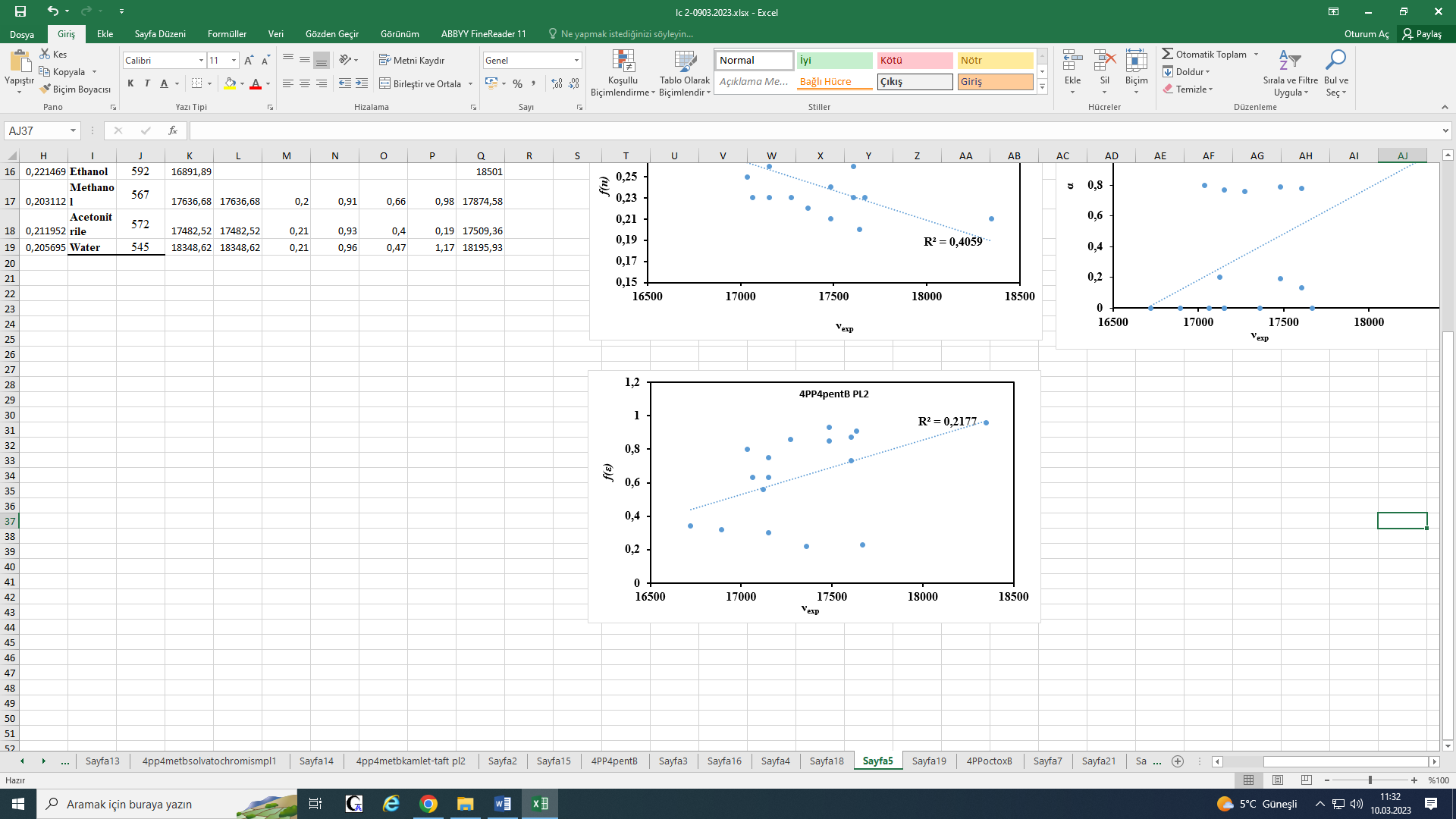

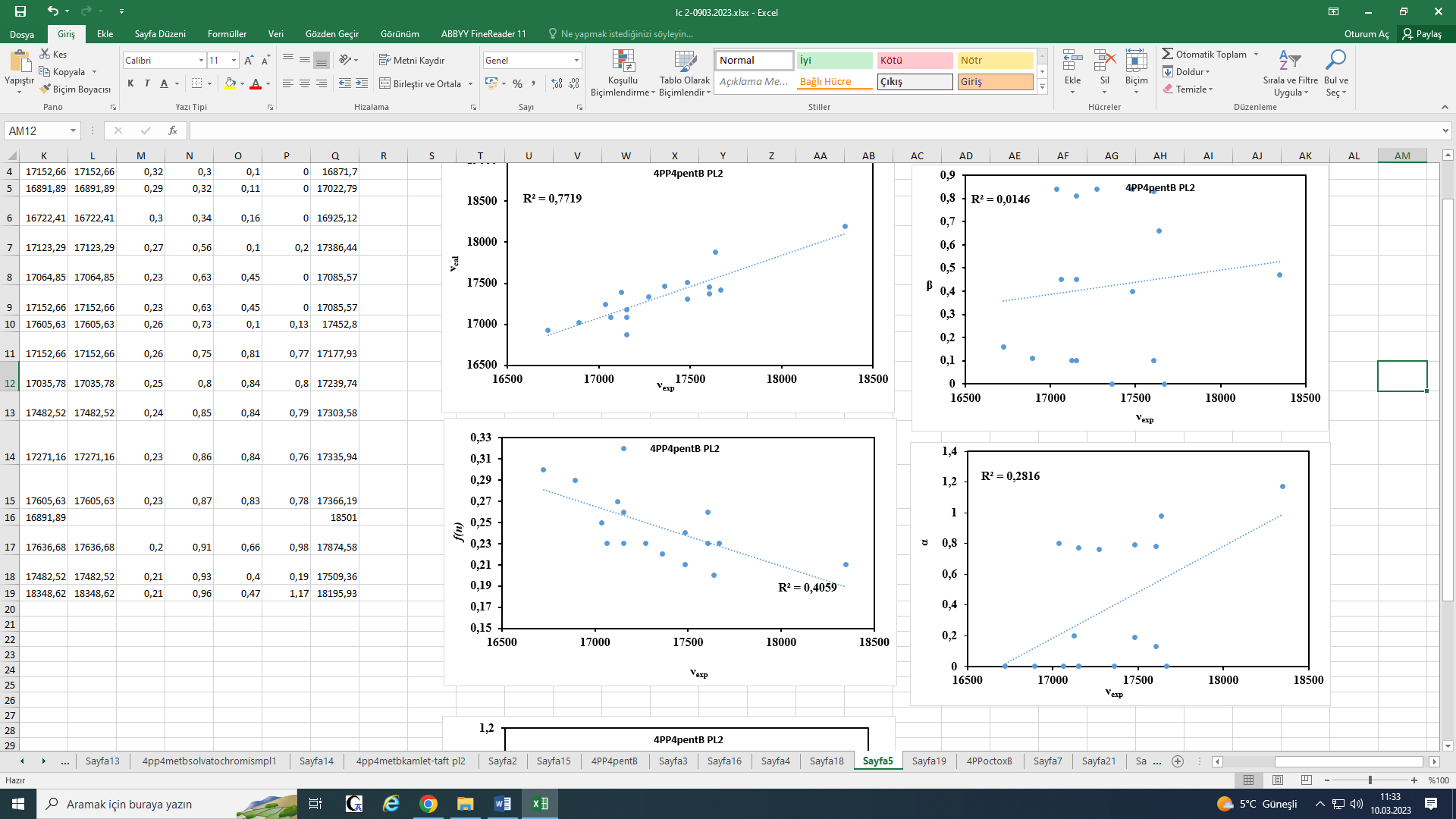

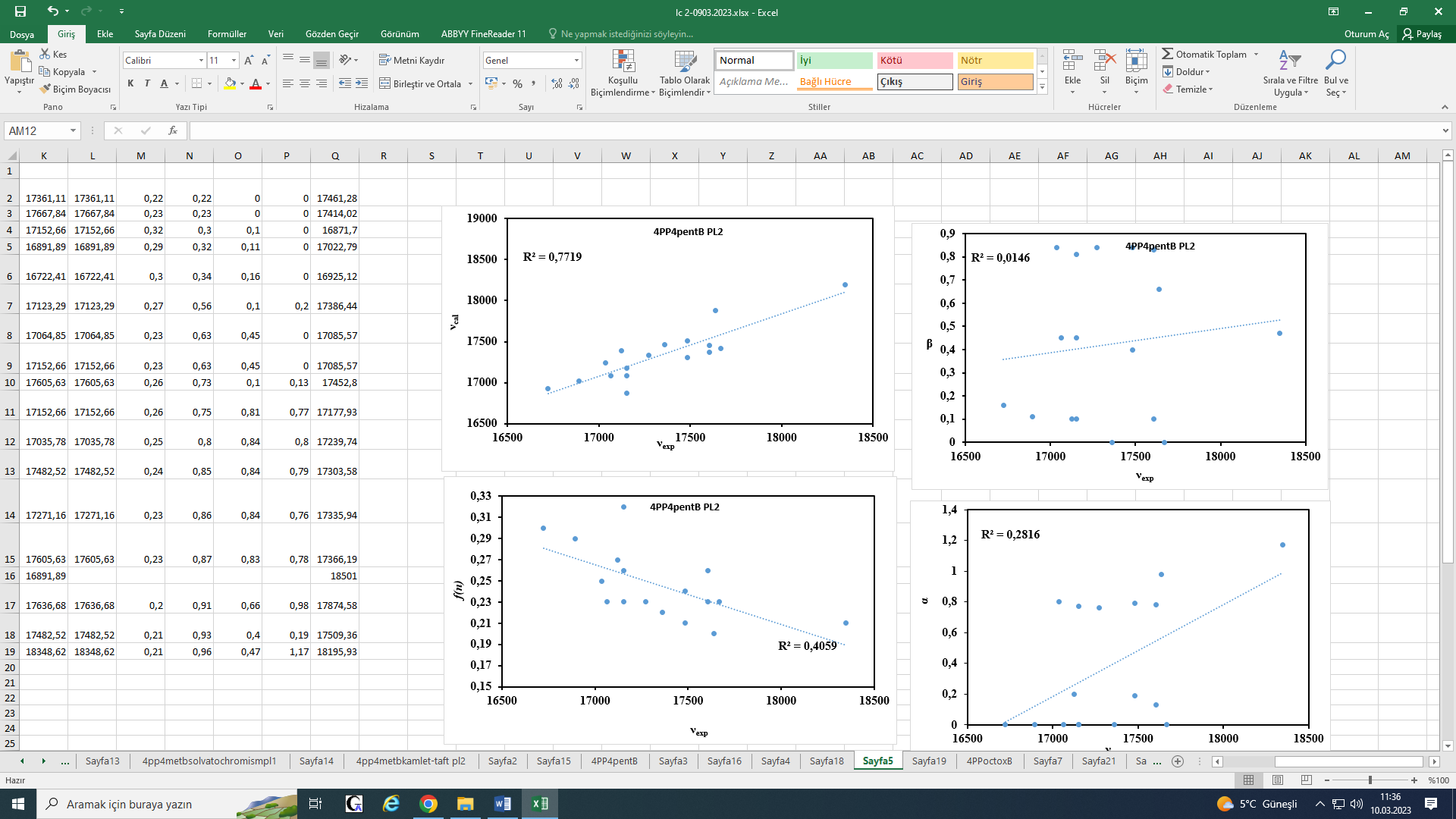

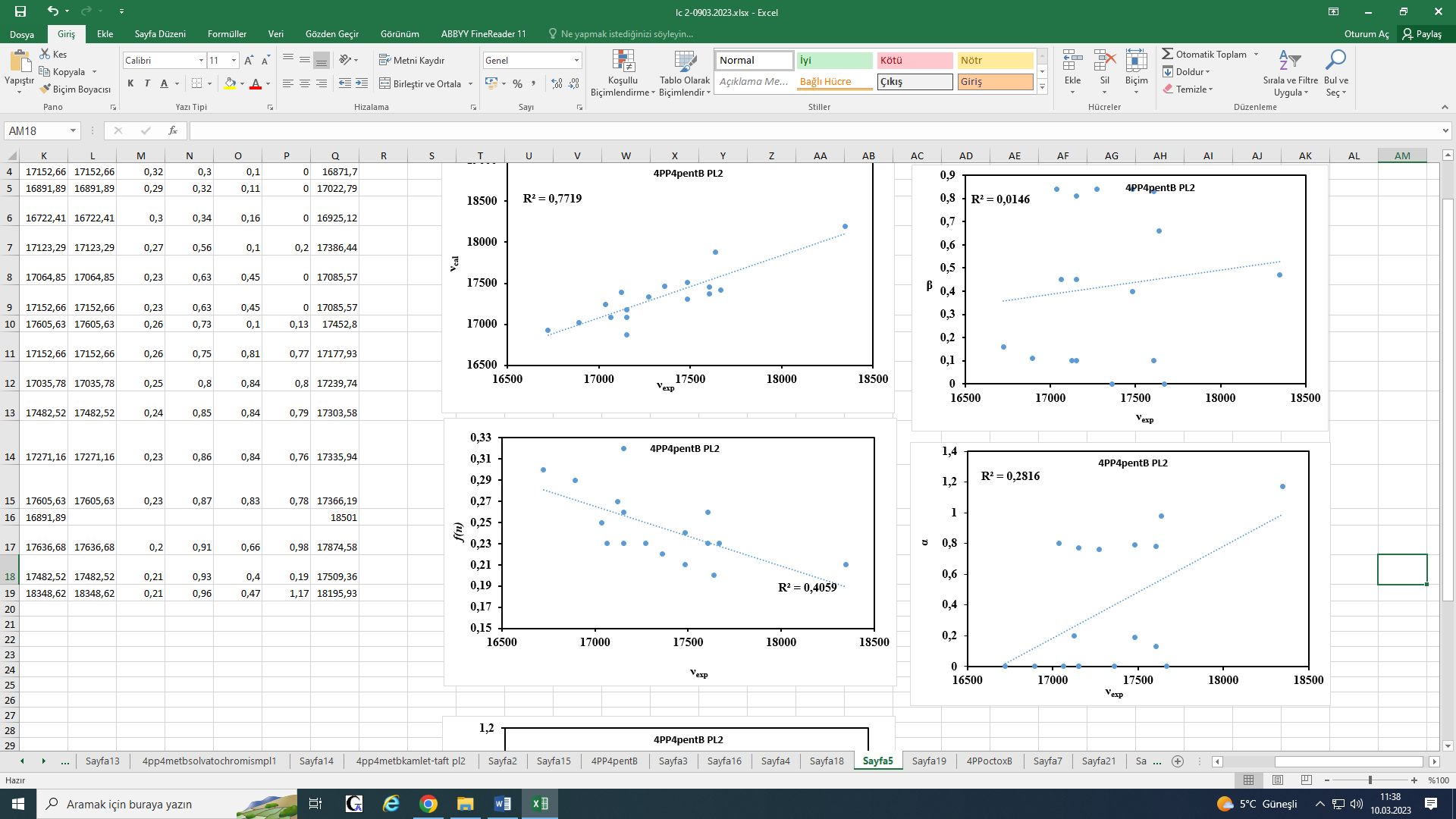


**Figure 14S.** The correlations of *ν_cal,_ β, α, f(n) and f(ε)* versus ν_exp_ of λ_PL2_ wavelength of 4PP4pentB molecule.


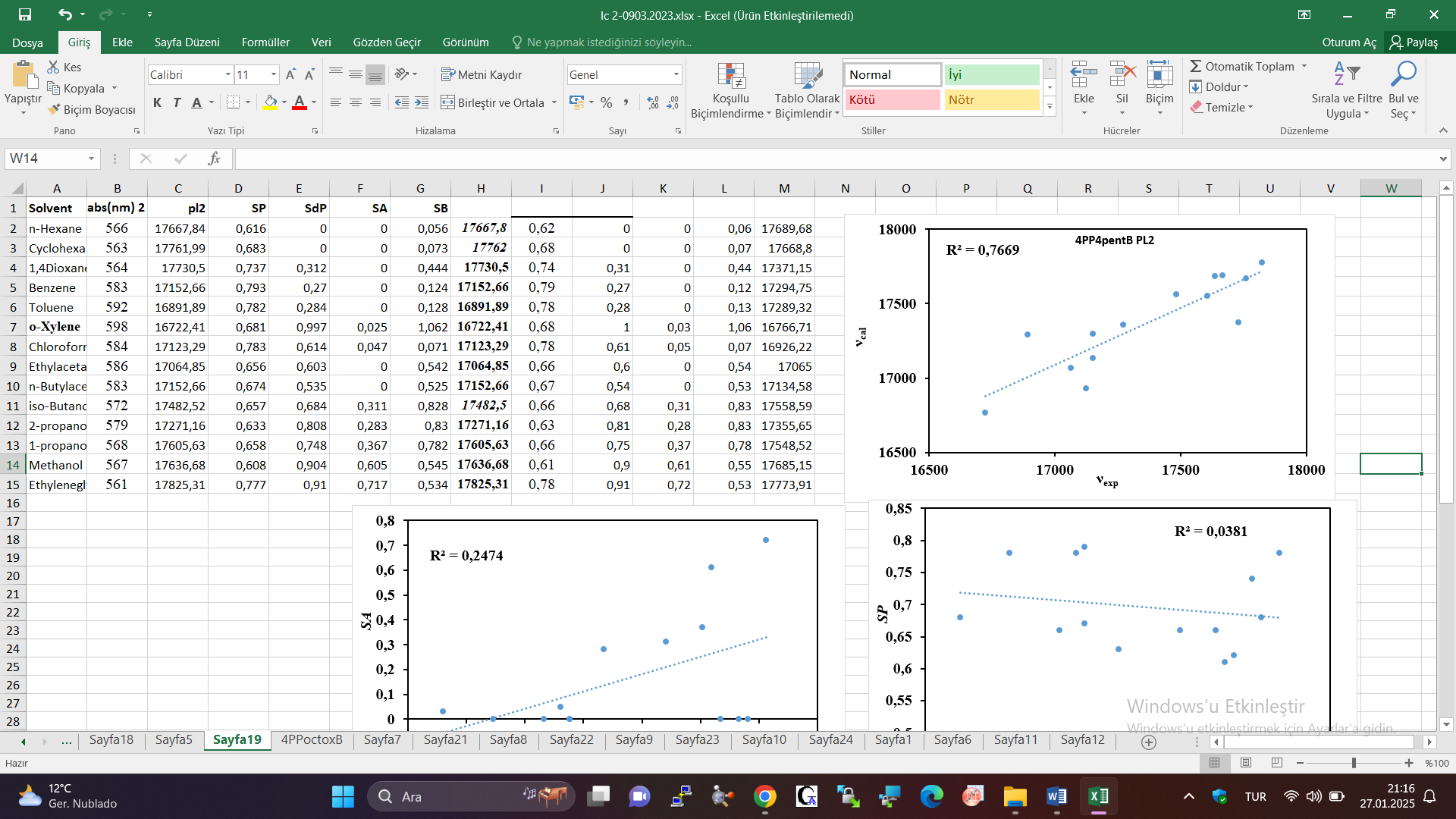


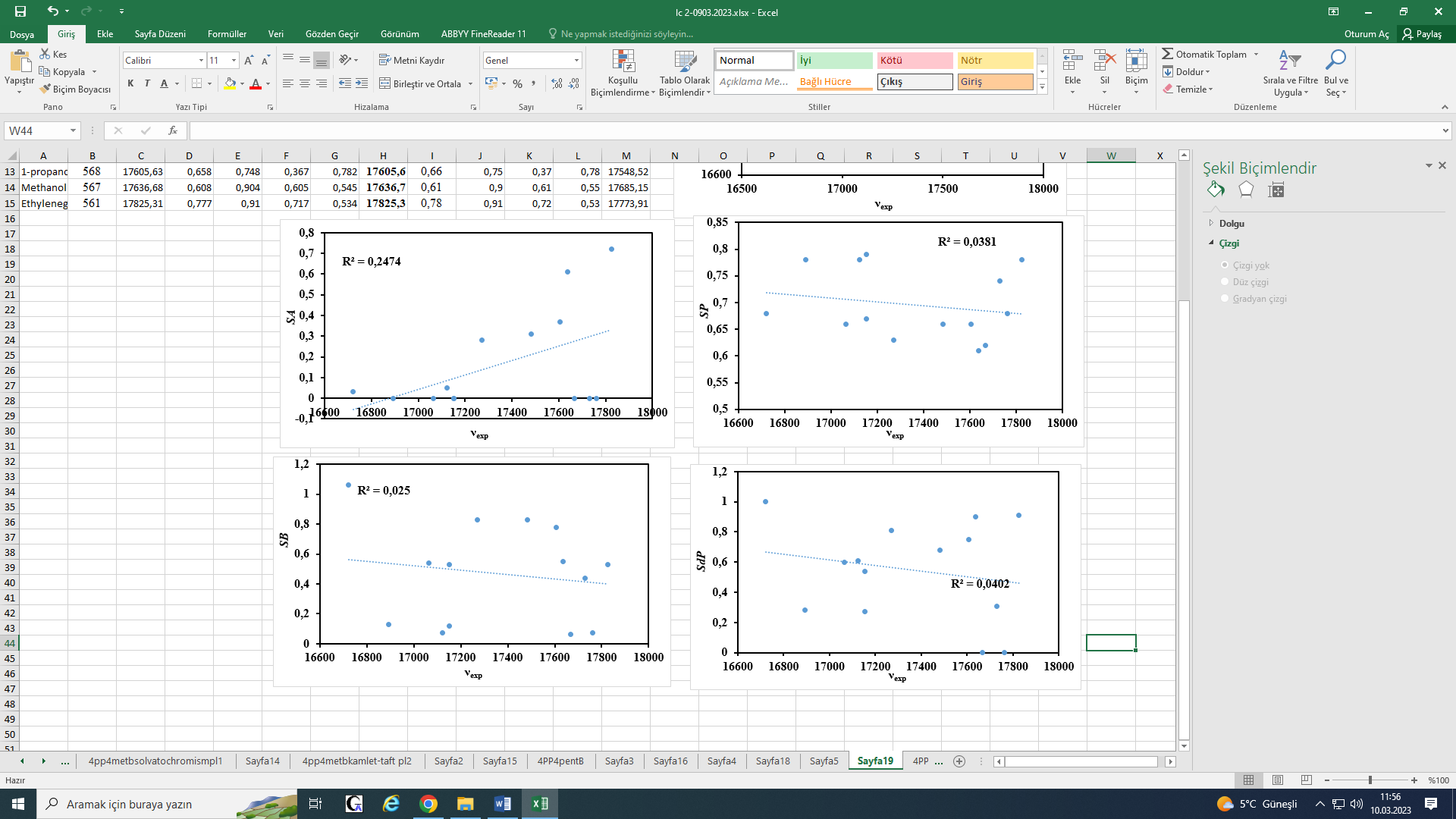

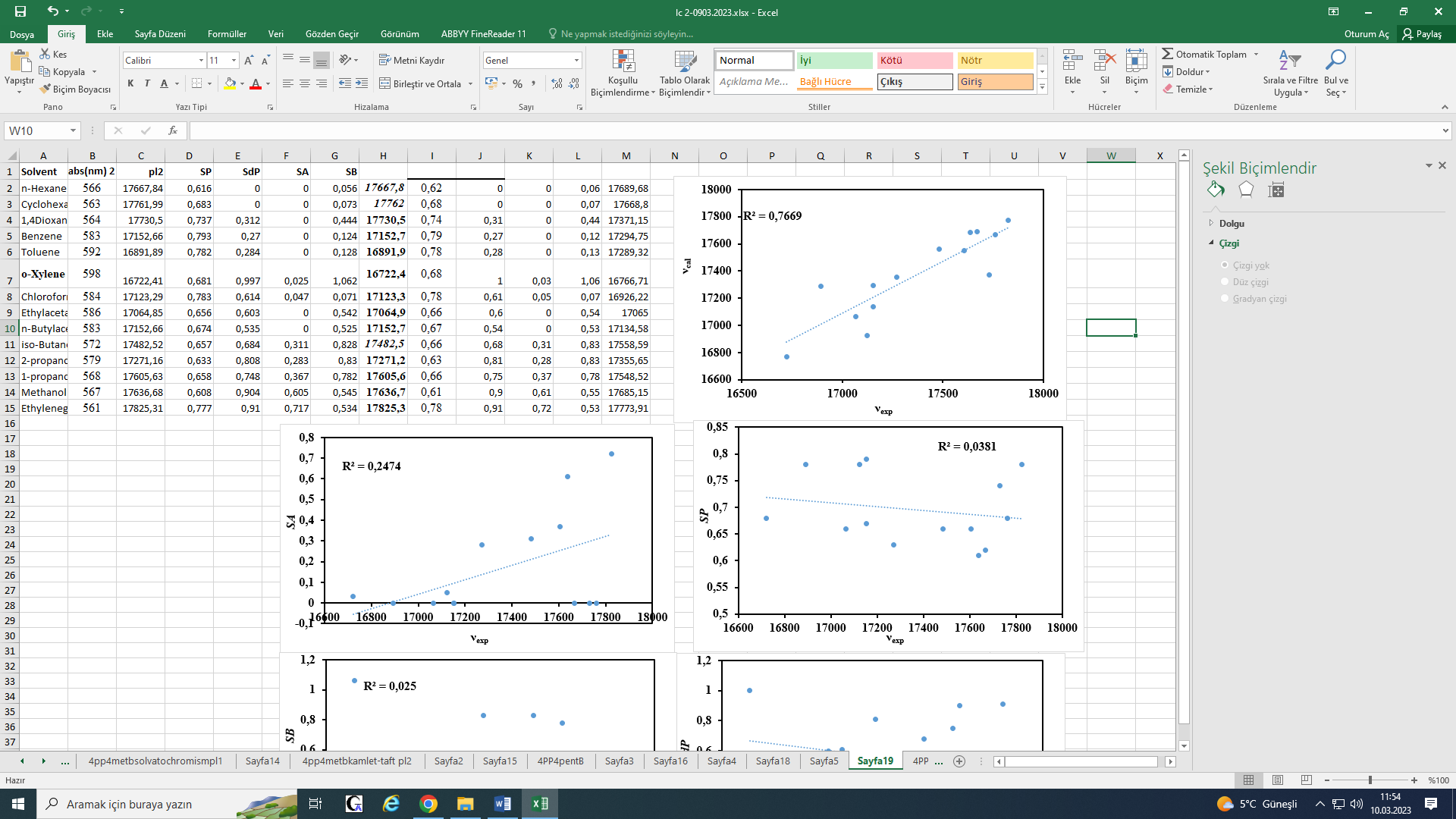

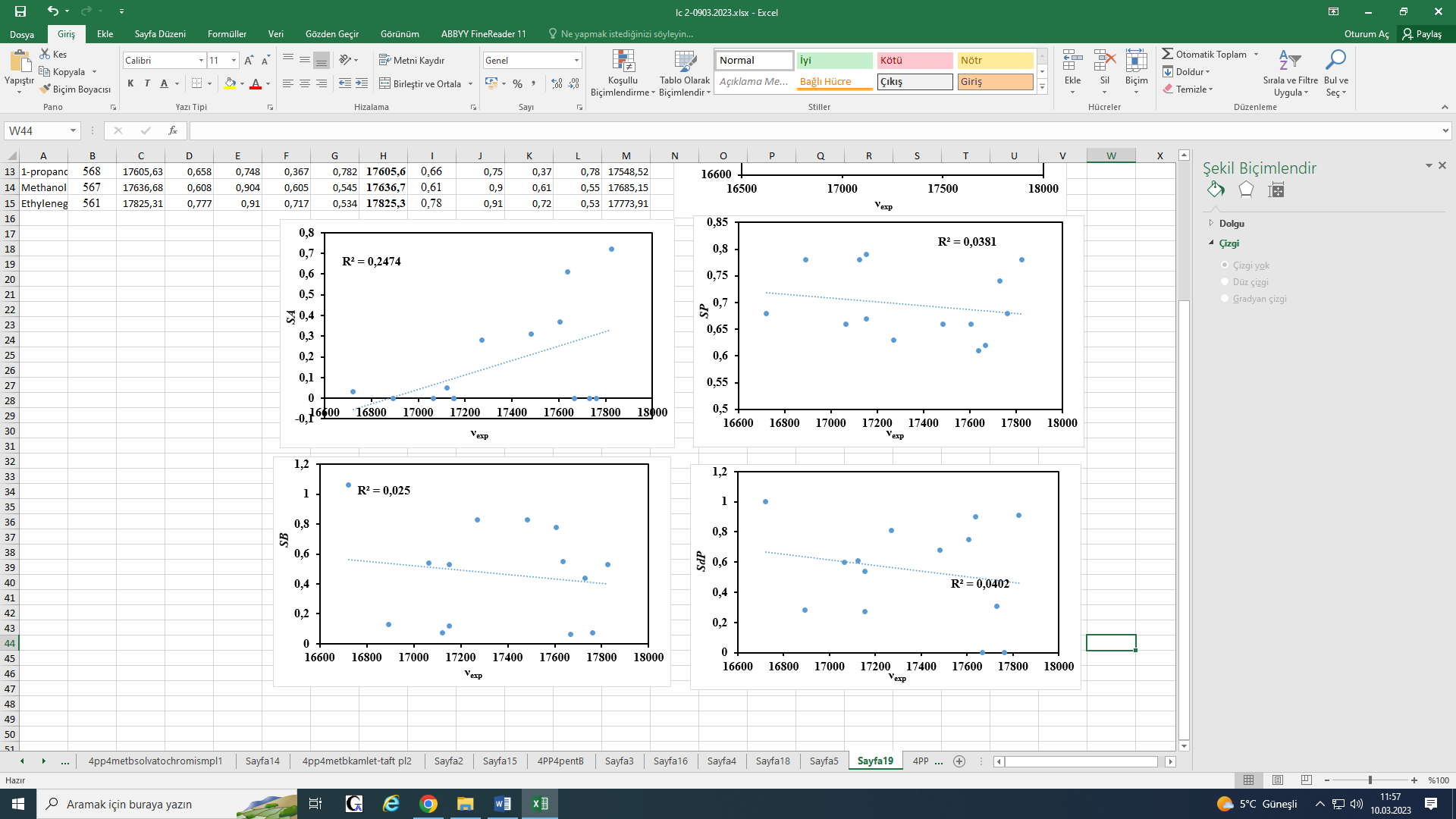

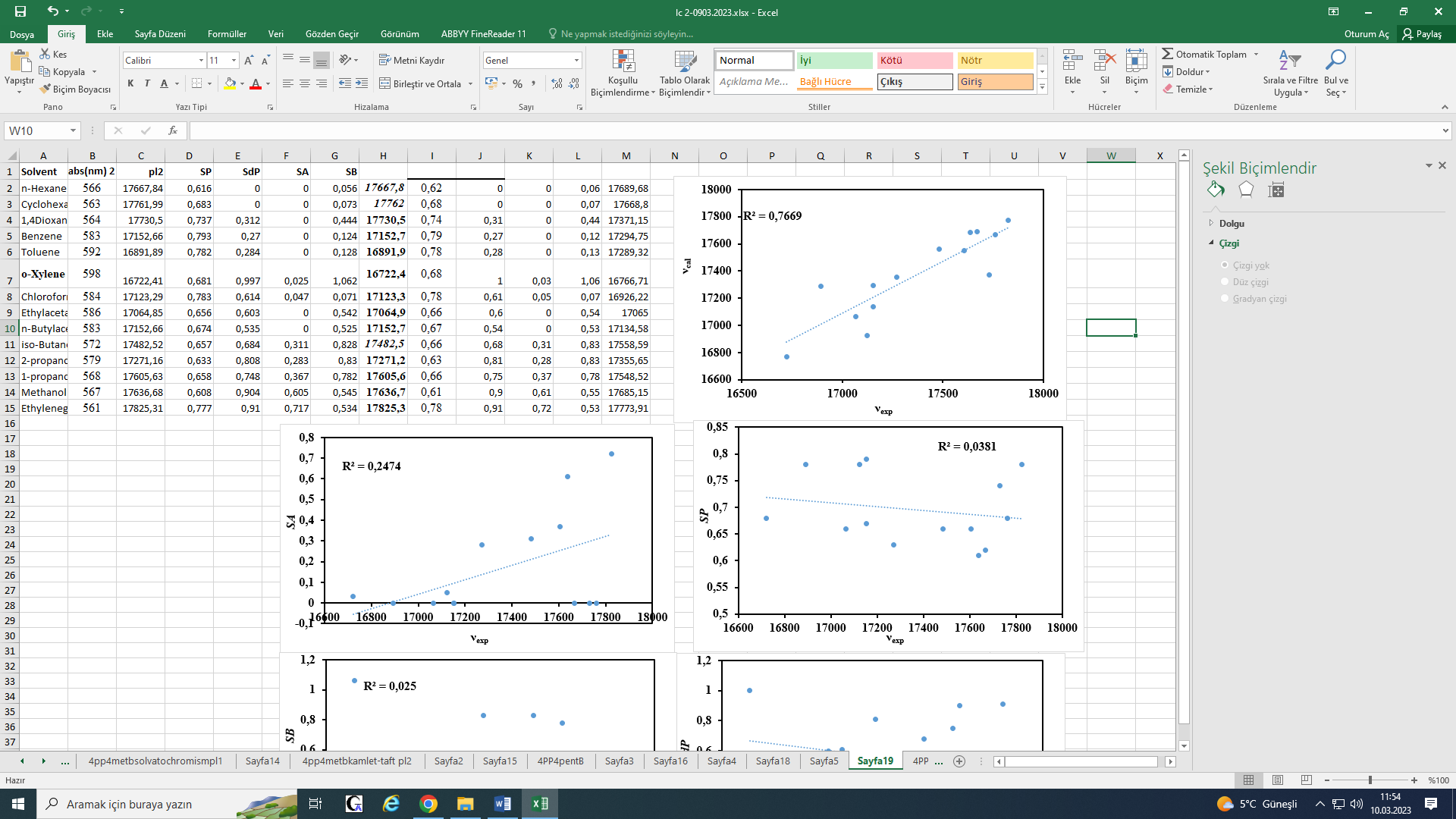


**4PP4pentB PL2**

**4PP4pentB PL2**

**4PP4pentB PL2**

**4PP4pentB PL2**


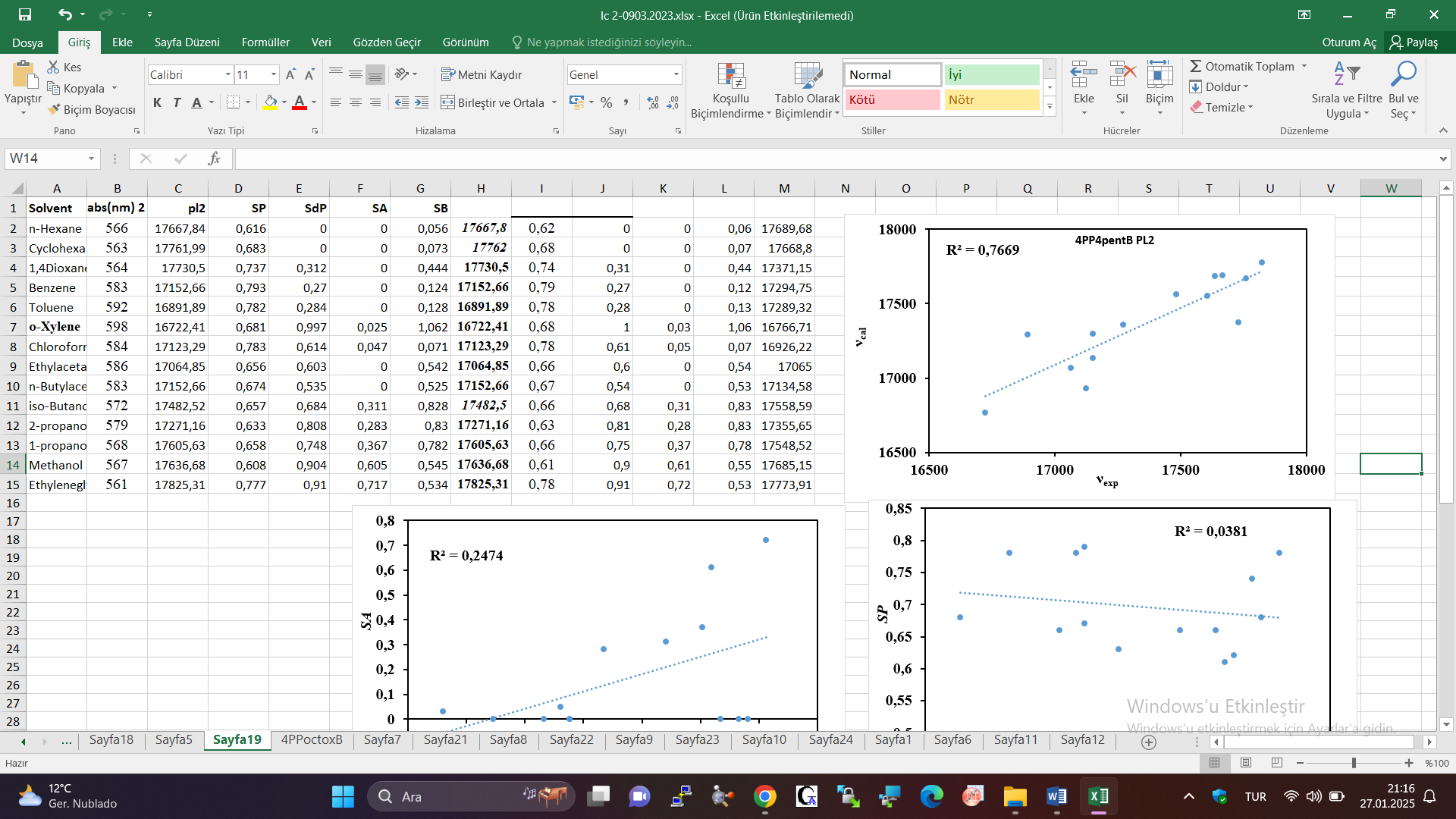


**Figure 15S.** The correlations of *ν_cal_, SP, SdP, SA* and *SB* versus ν_exp_ of λ_PL2_ wavelength of 4PP4pentB molecule.


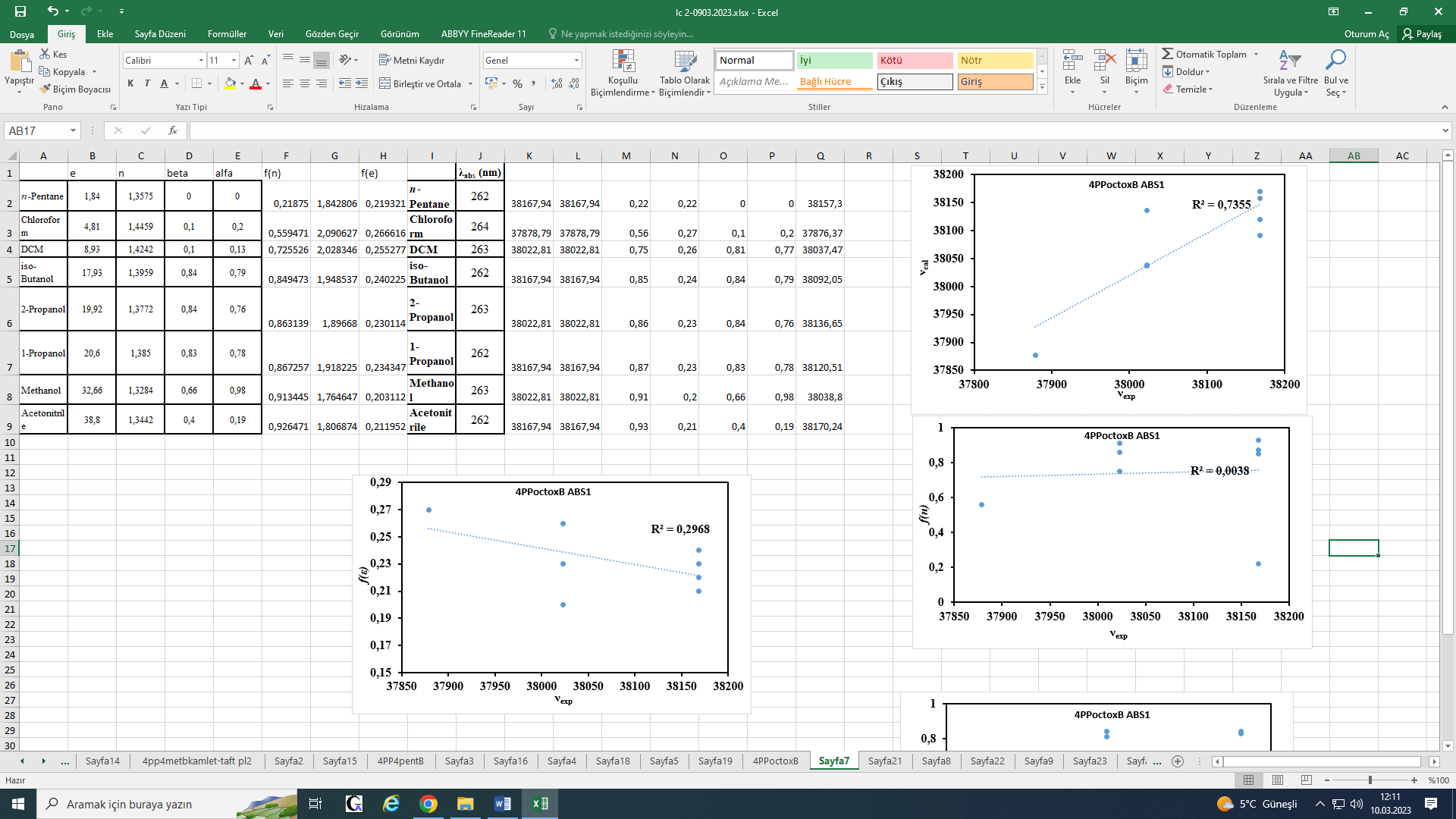

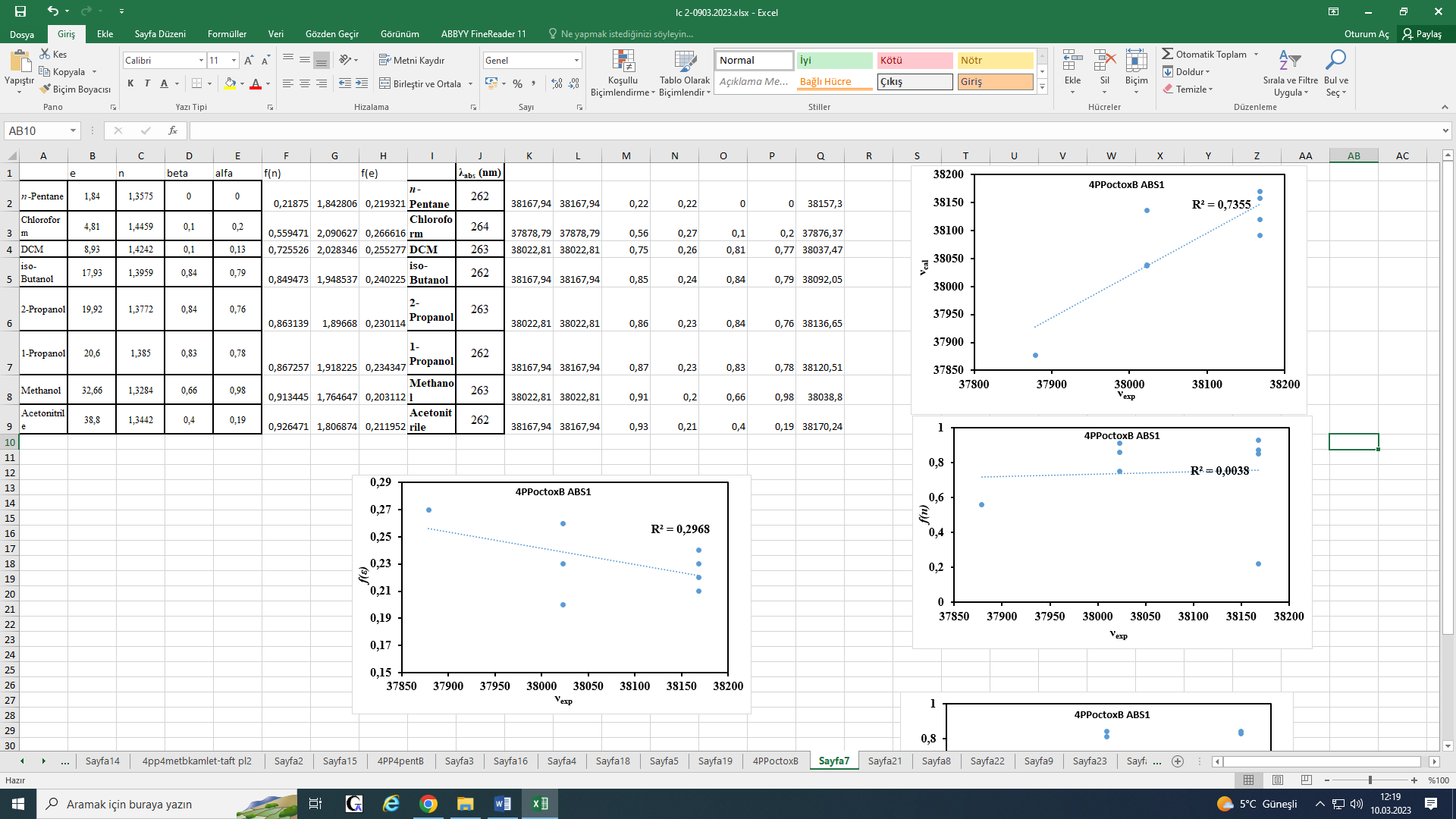

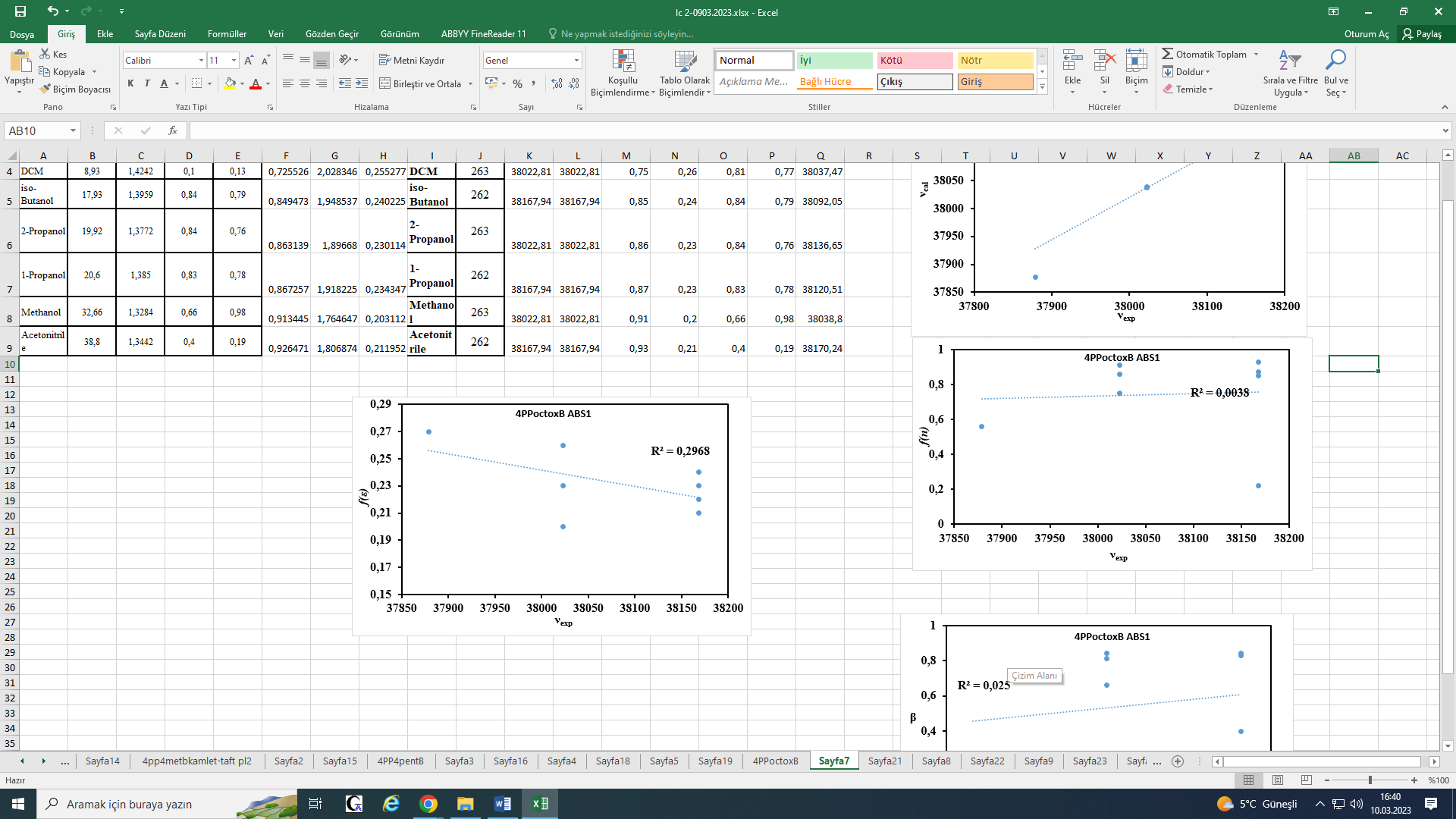

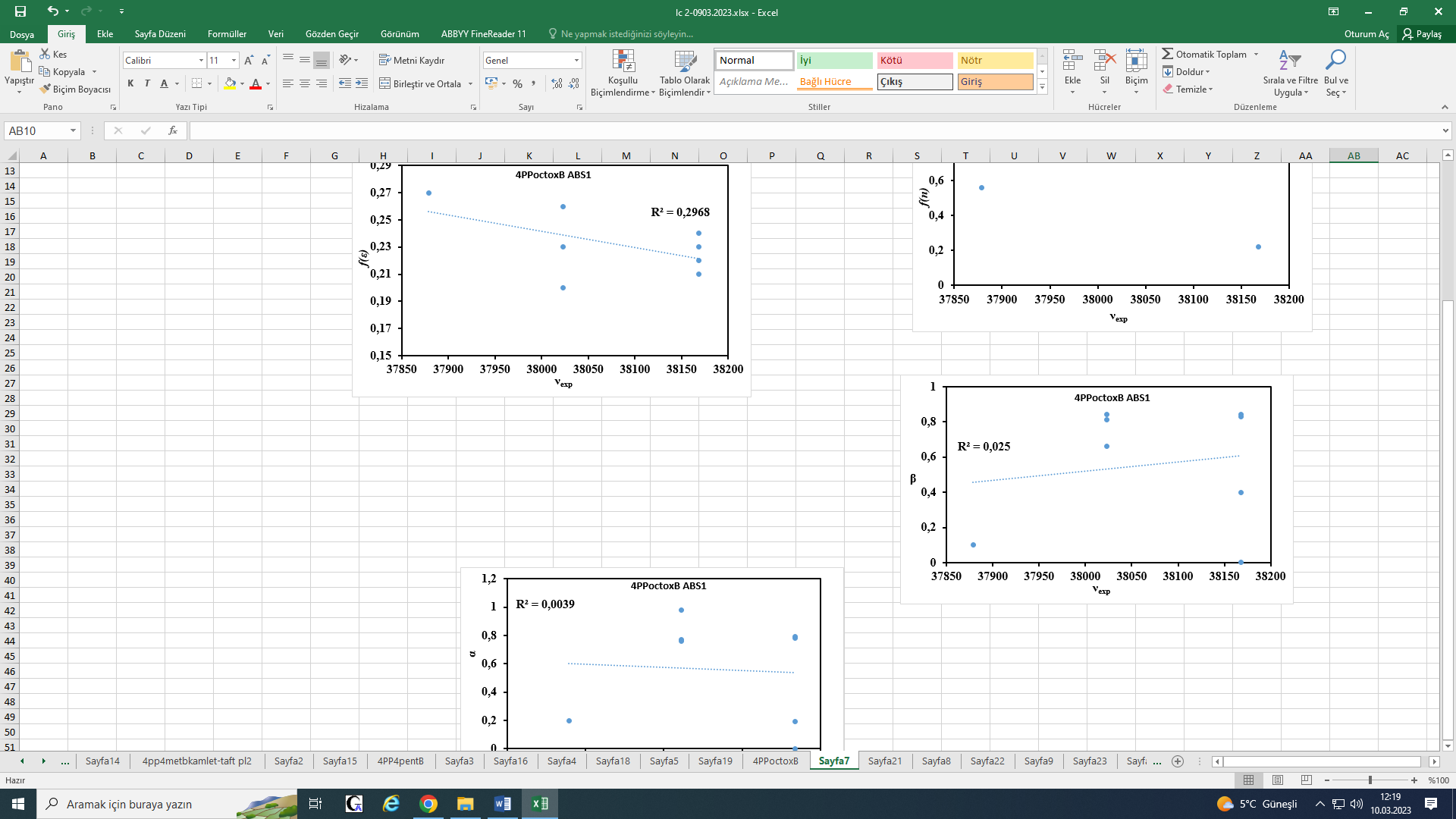

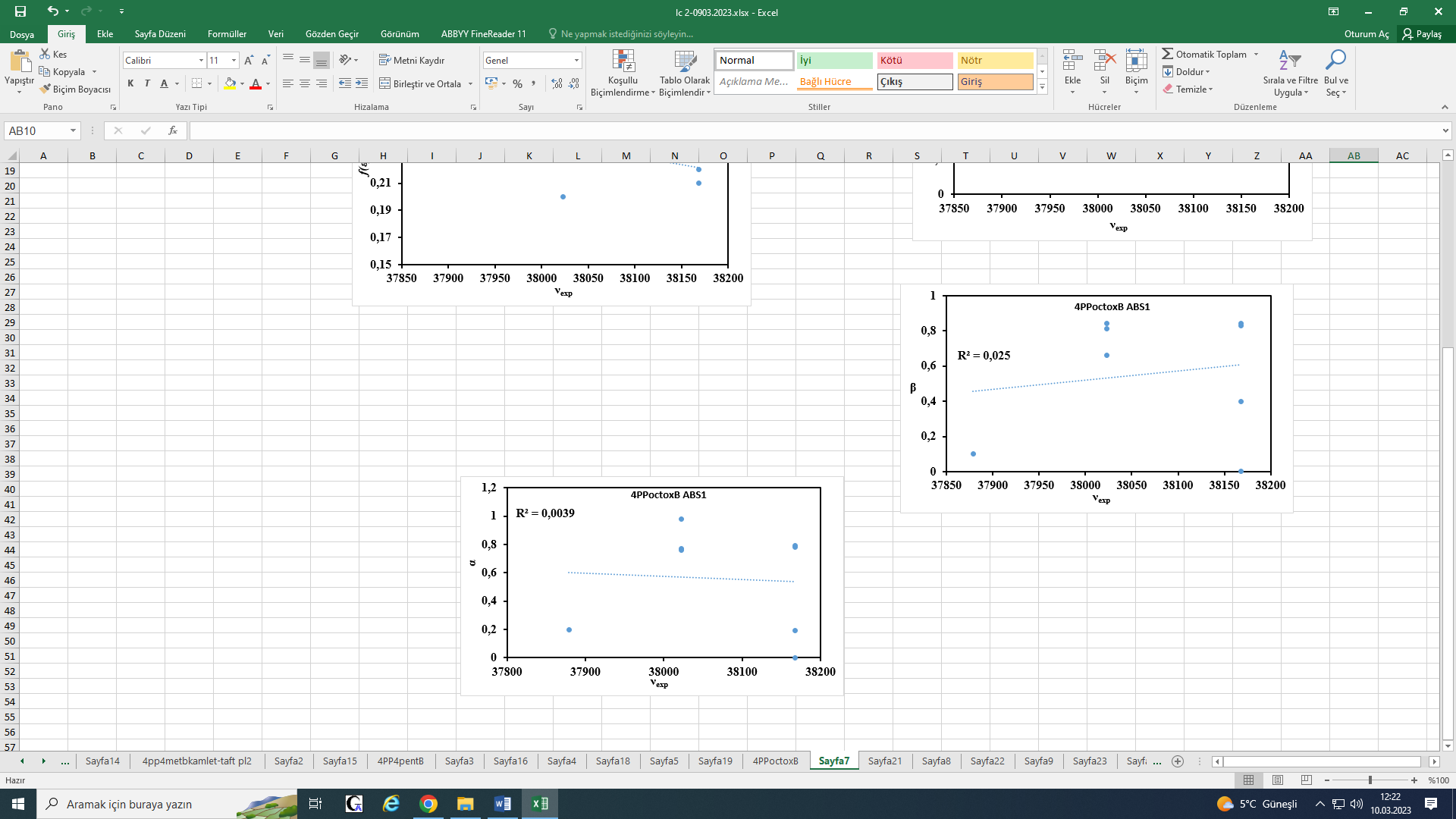


**Figure 16S.** The correlations of *ν_cal,_ β, α, f(n)* and *f(ε)* versus ν_exp_  of λ_ABS1_ wavelength of 4PPoctoxBB molecule.


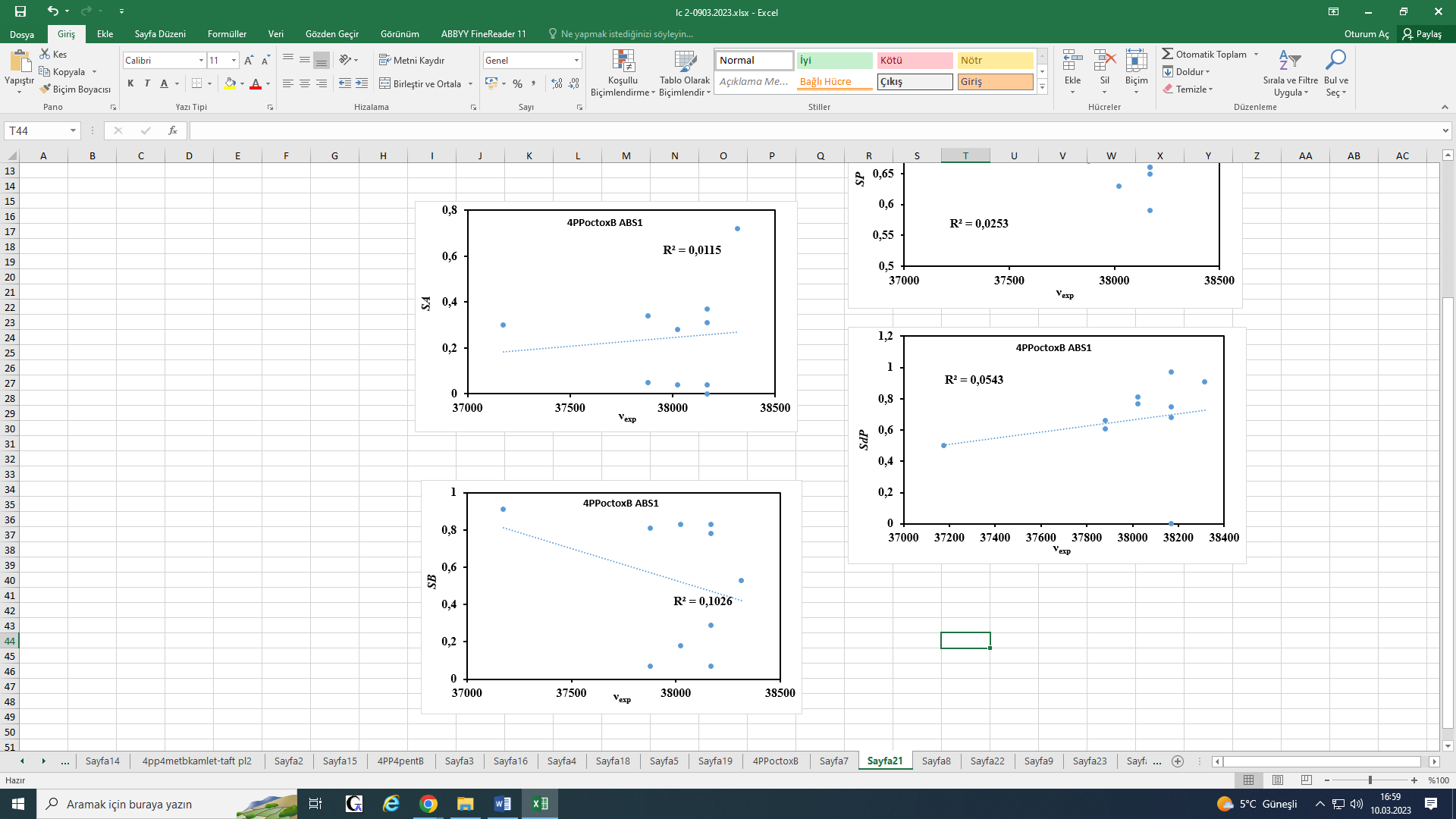

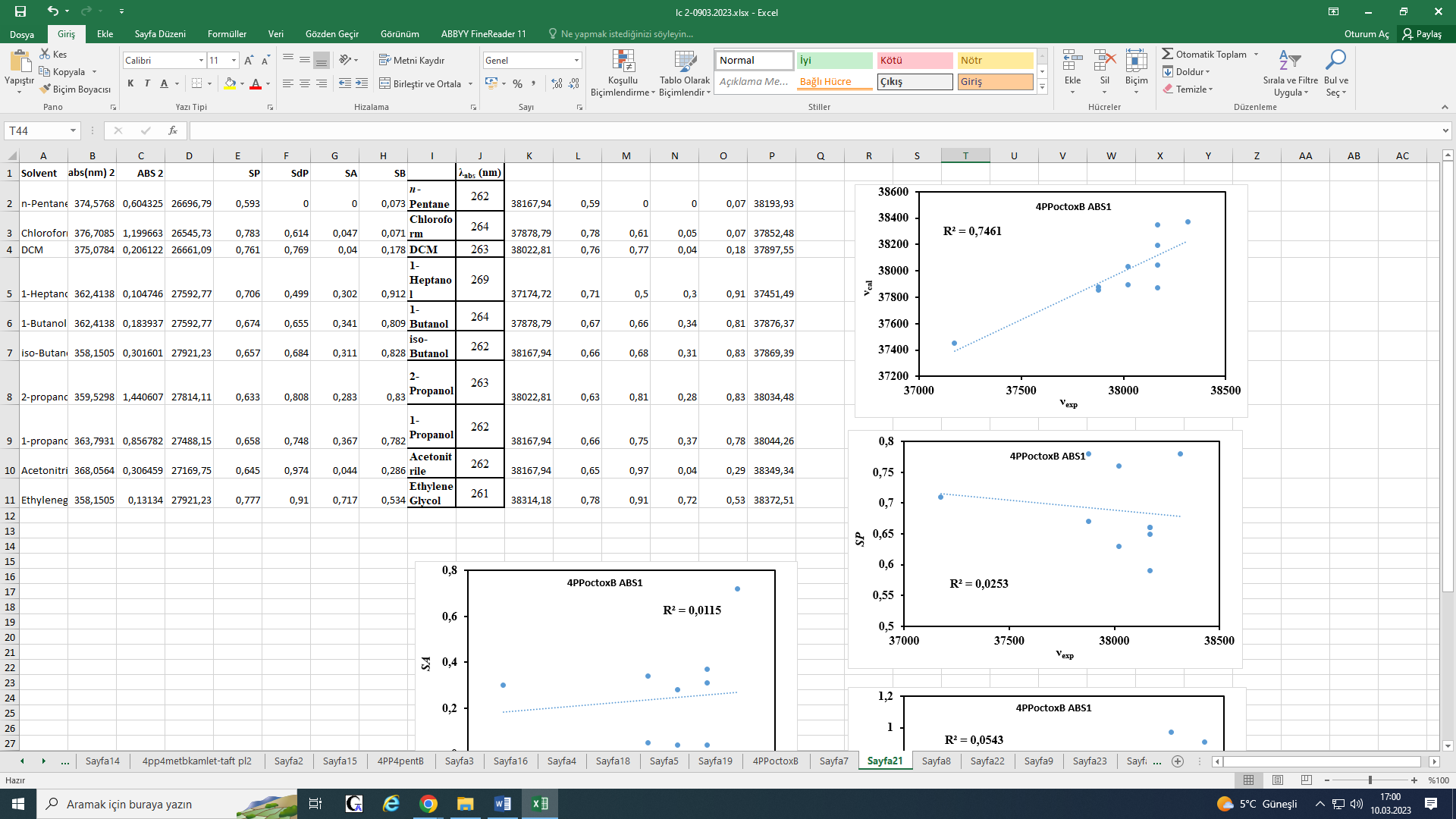

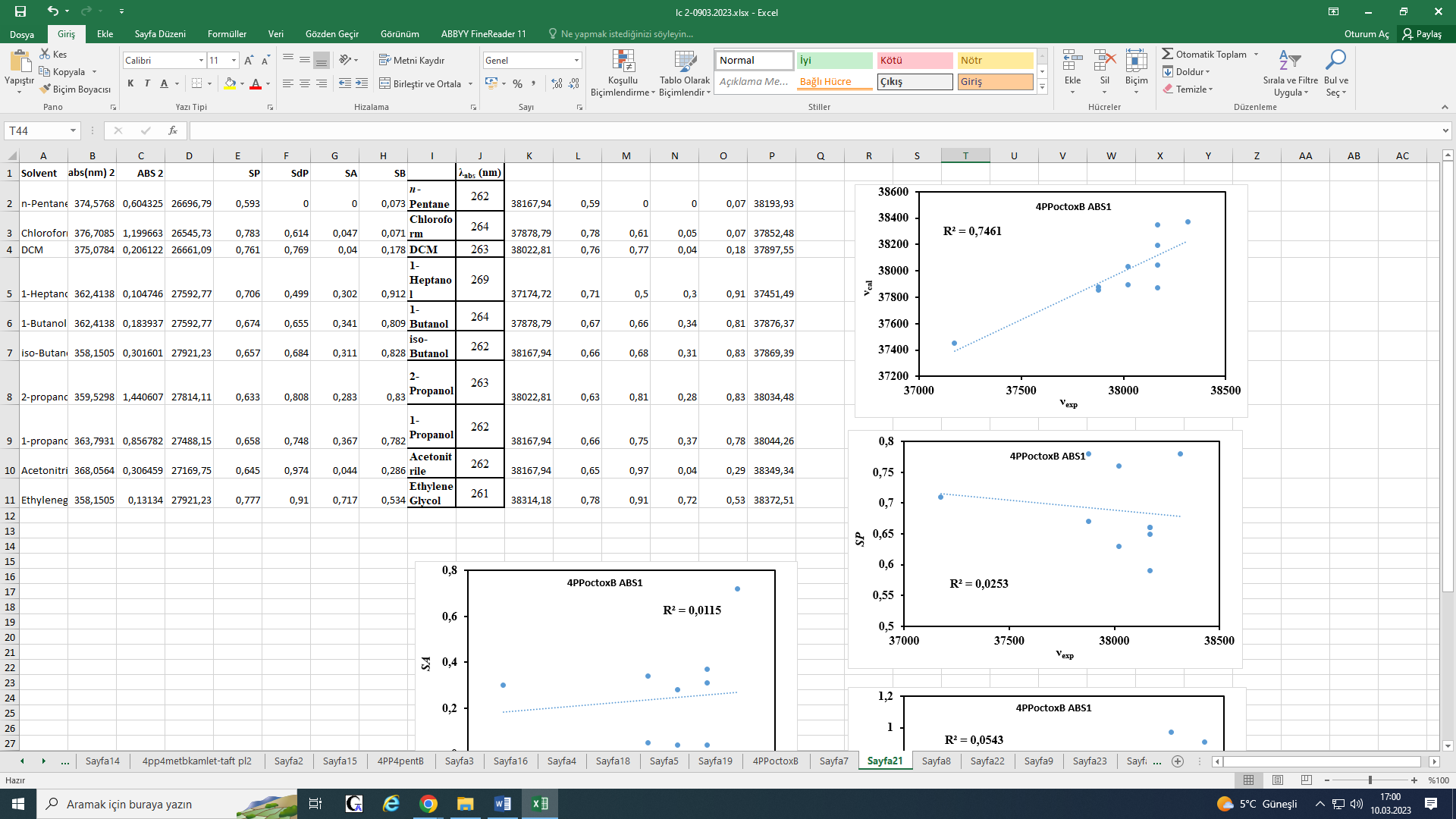

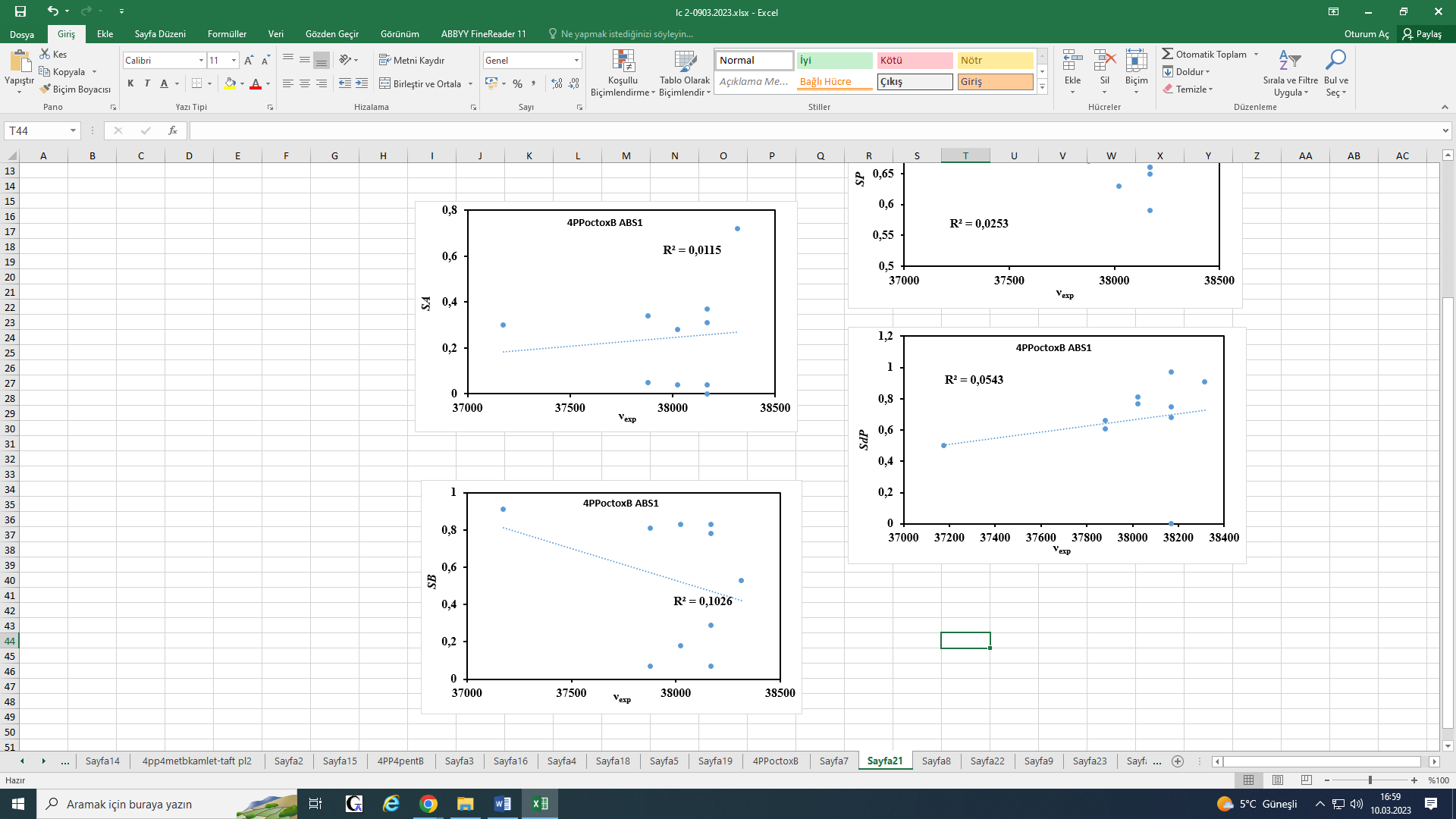

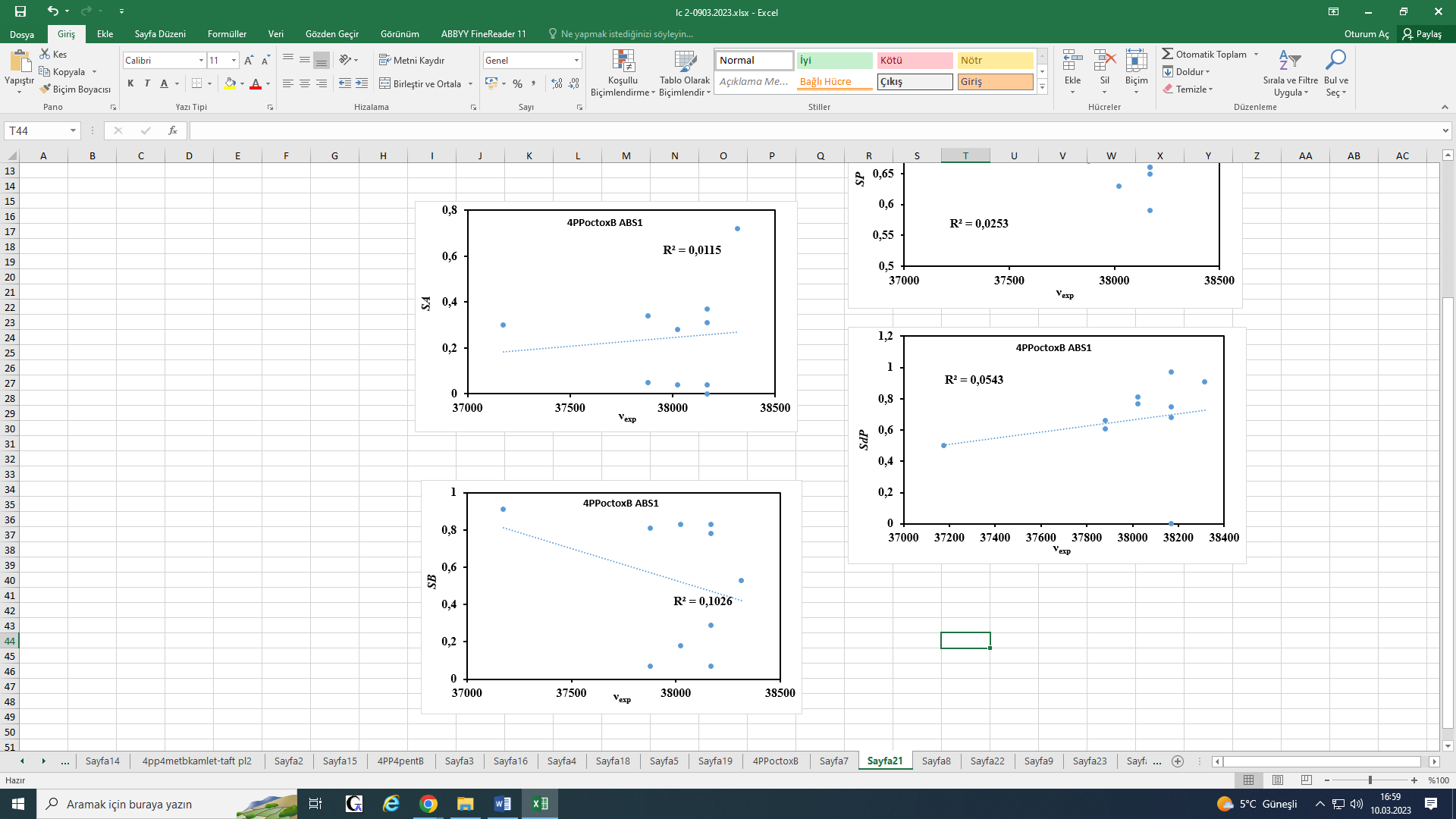


**Figure 17S.** The correlations of *ν_cal_, SP, SdP, SA* and *SB* versus ν_exp_  of λ_ABS1_ wavelength of 4PP4pentB molecule.


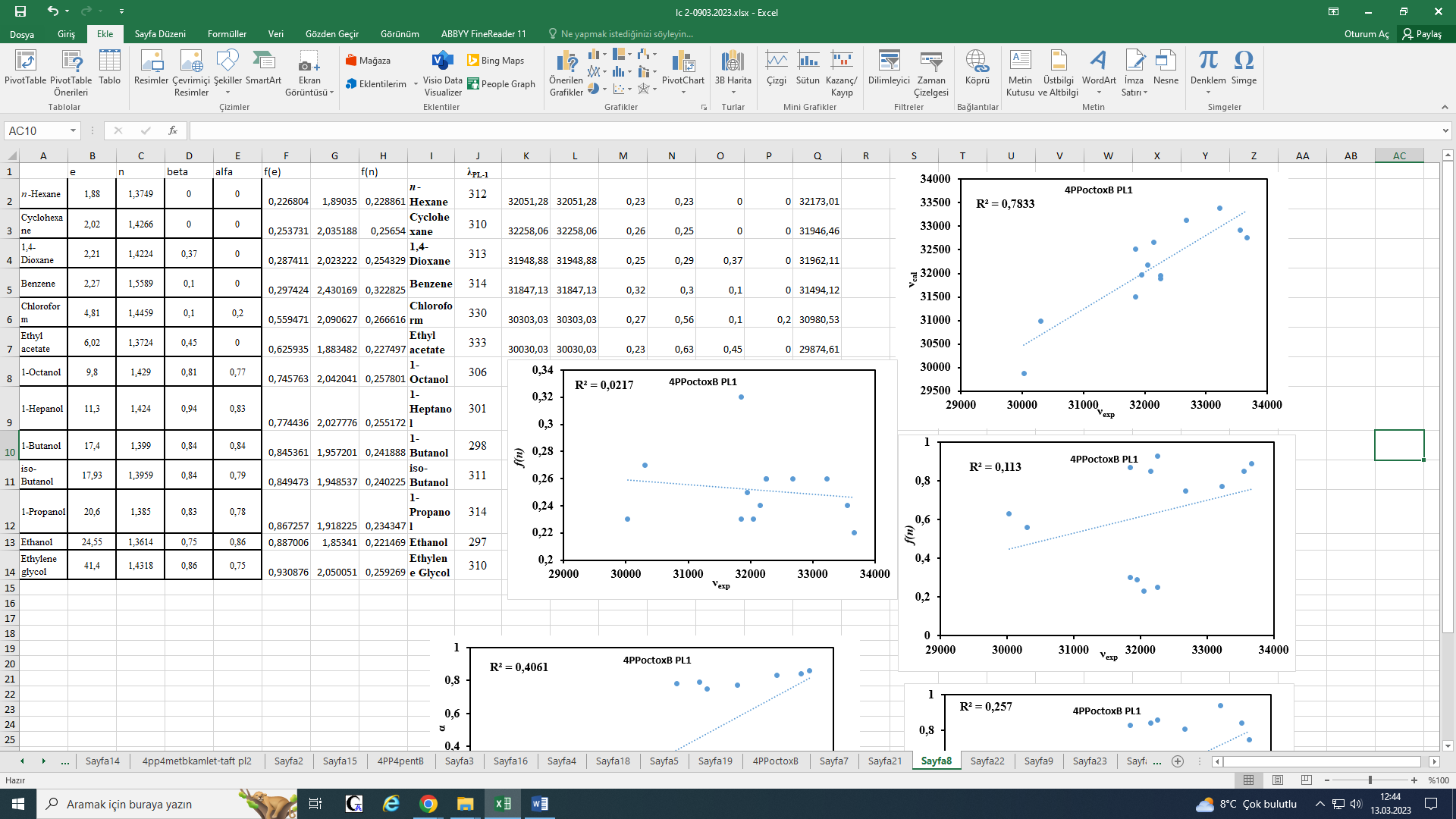

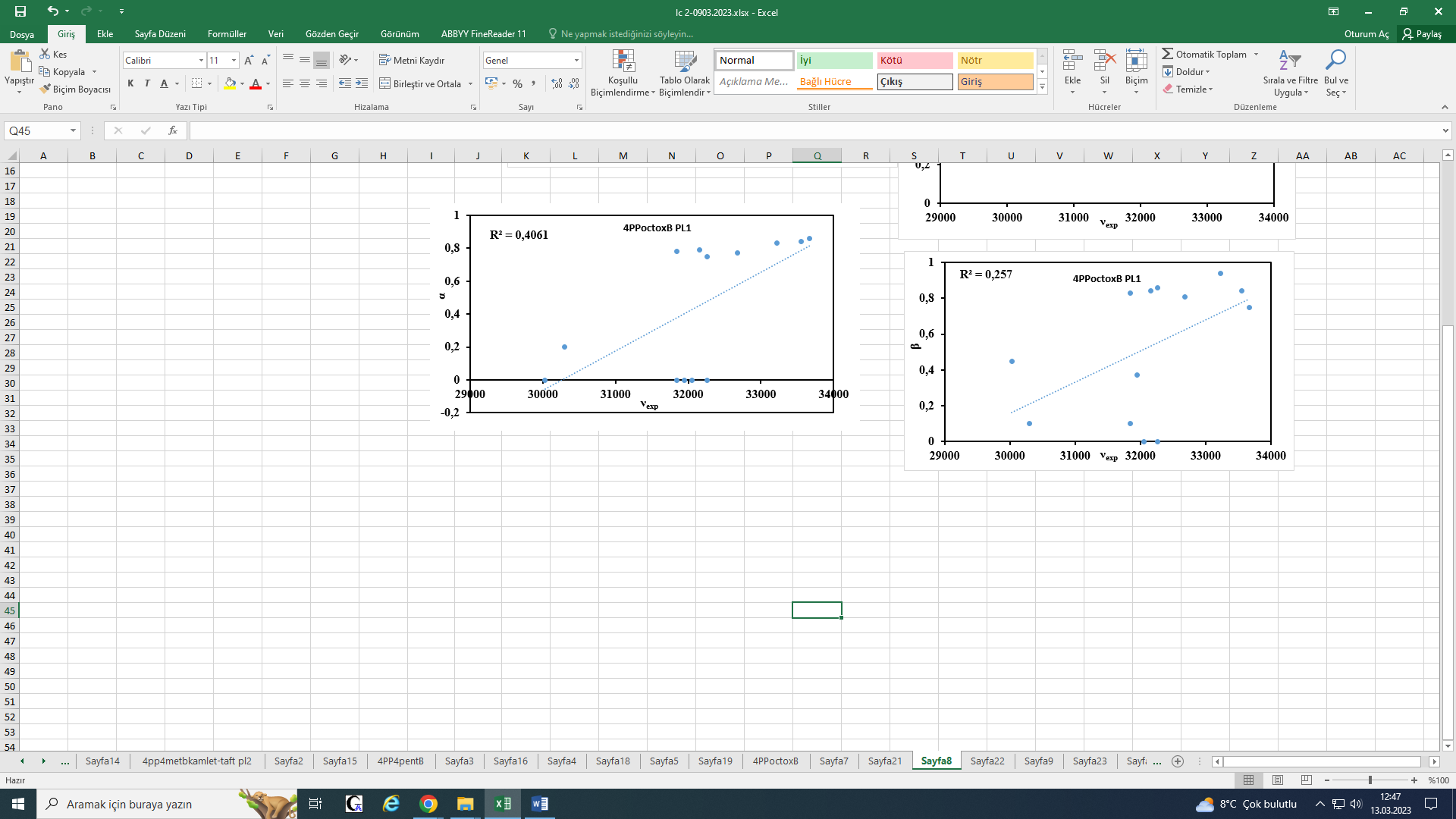

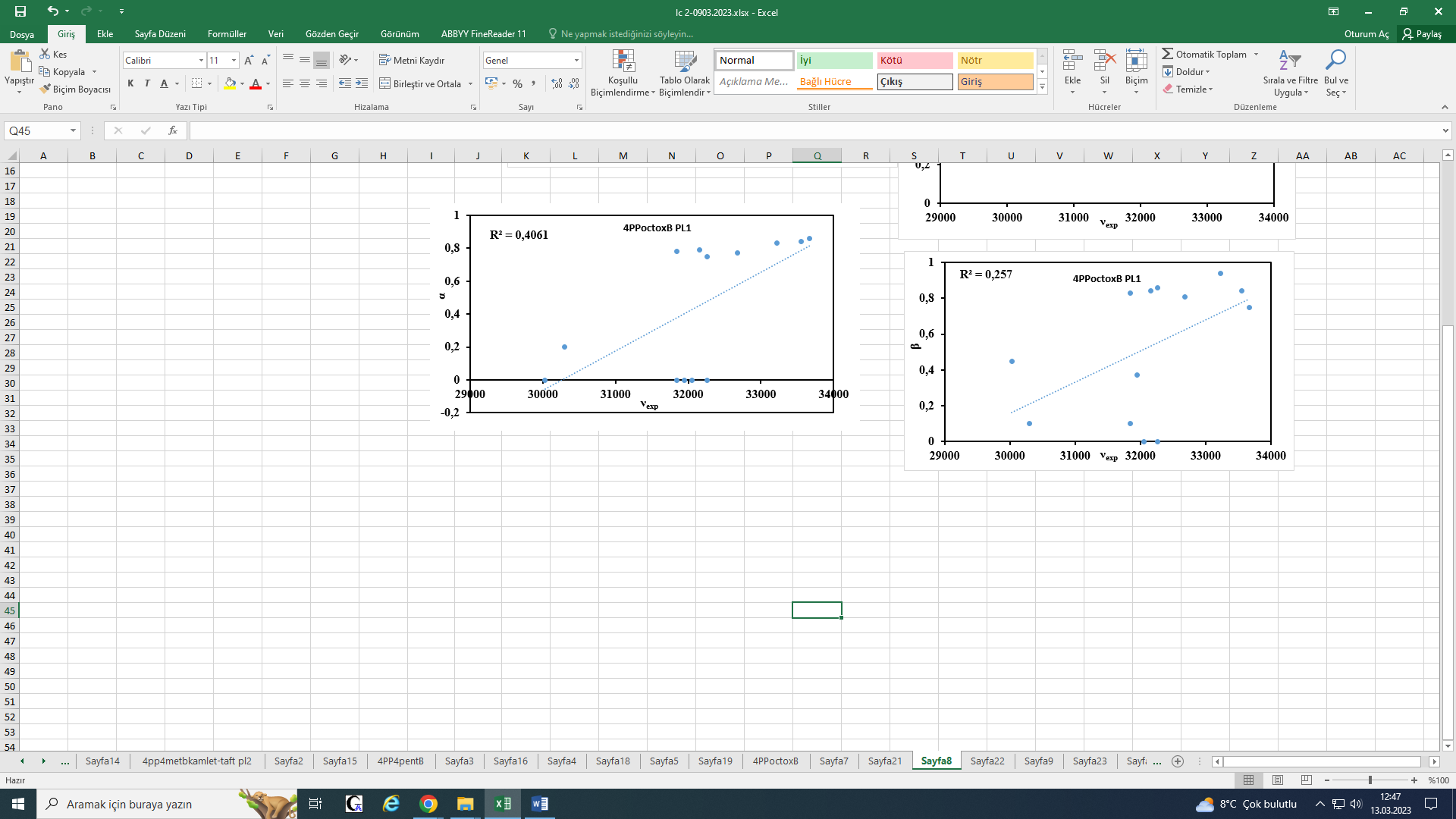

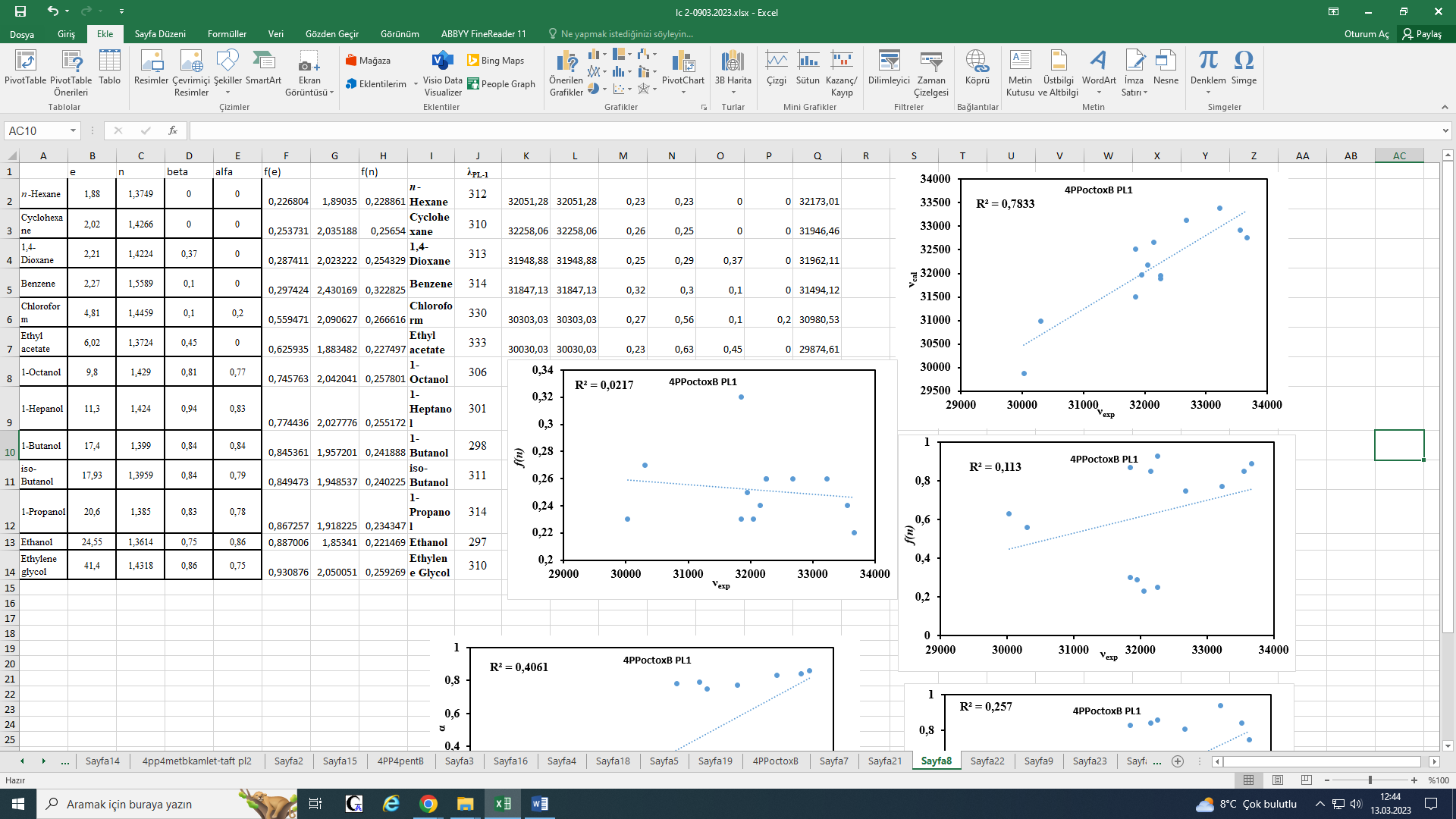

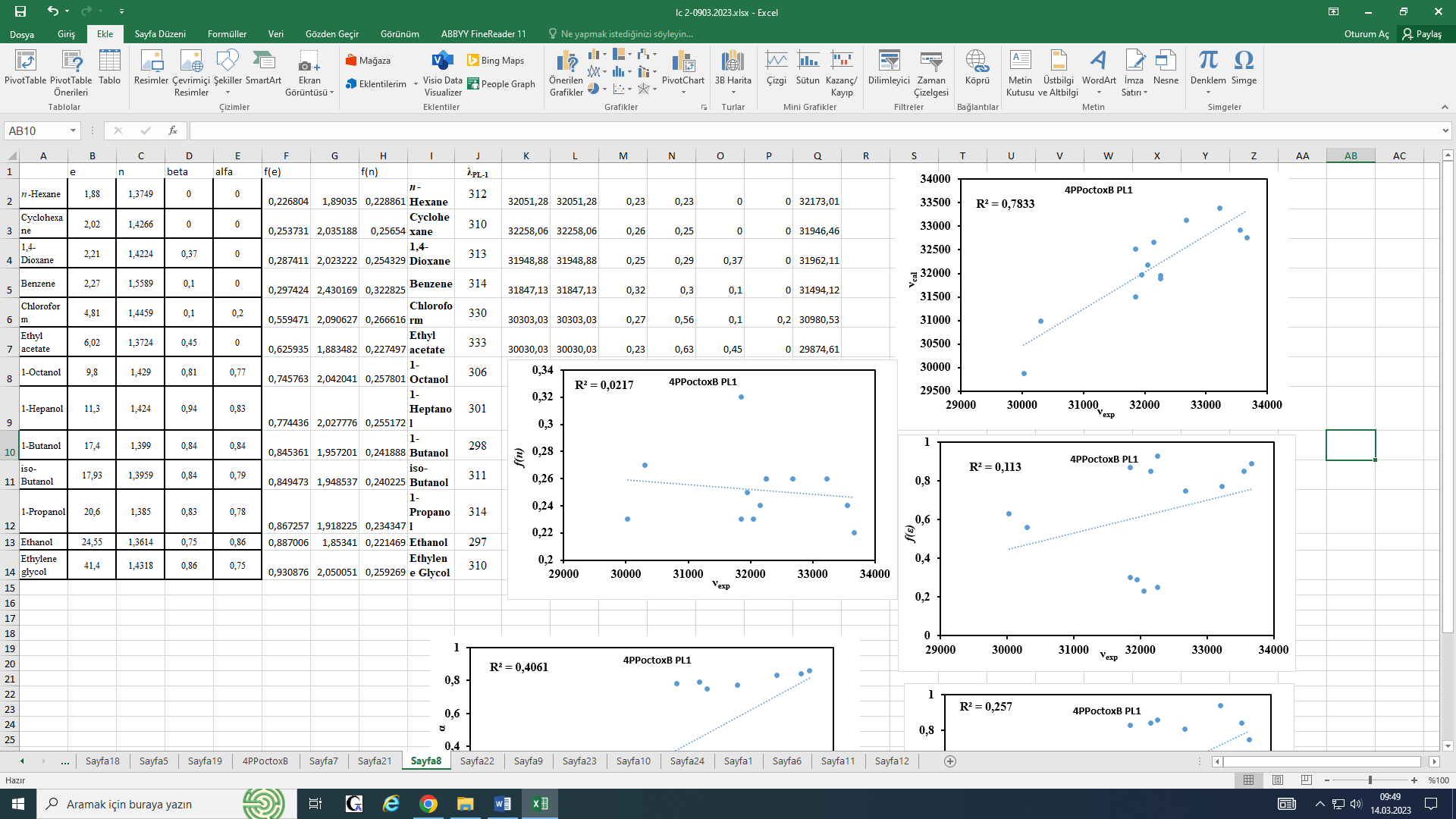


**Figure 18S.** The correlations of *ν_cal_, β, α, f(n)* and *f(ε)* versus ν_exp_ of λ_PL1_ wavelength of 4PPoctoxB molecule.


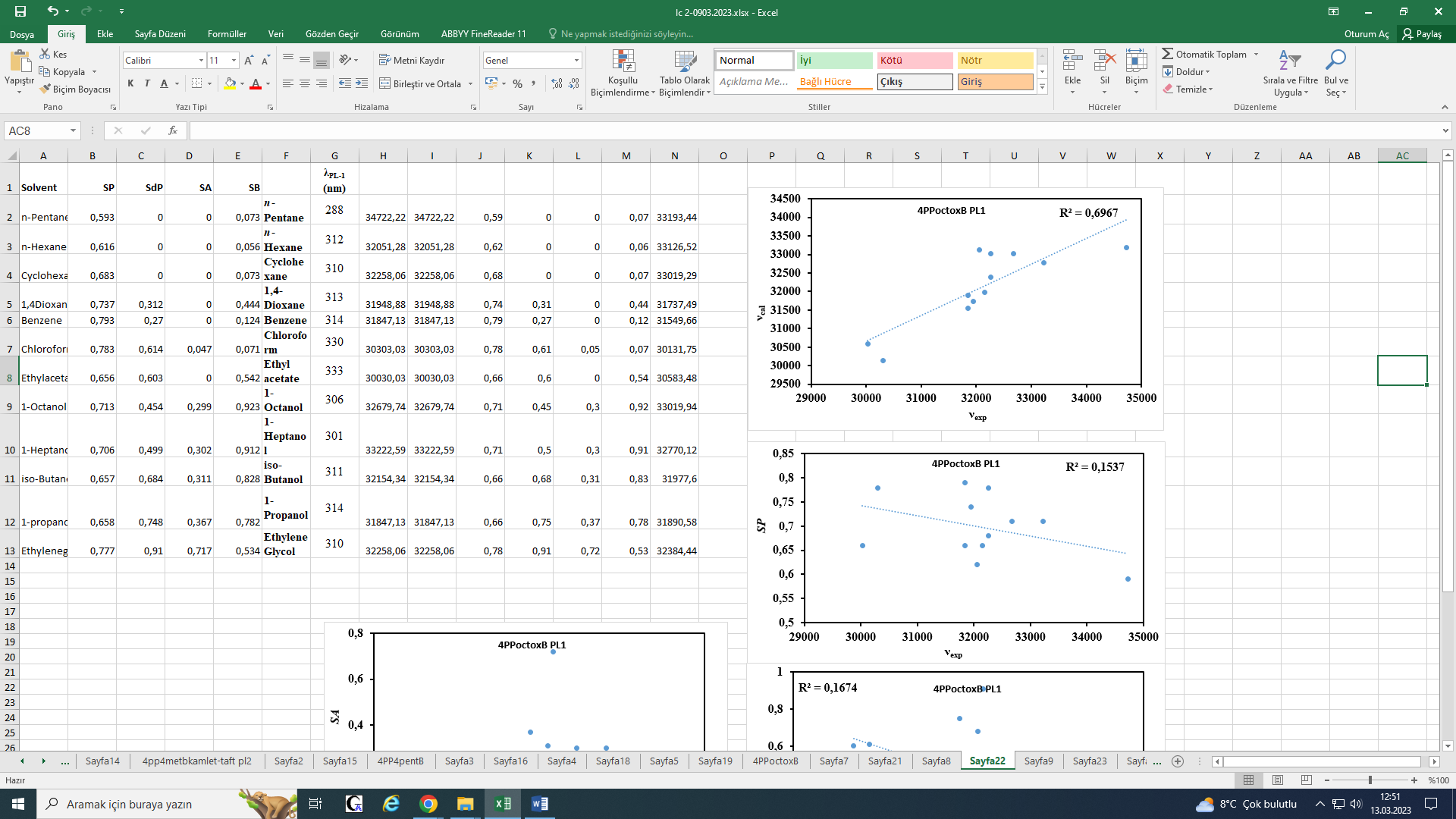

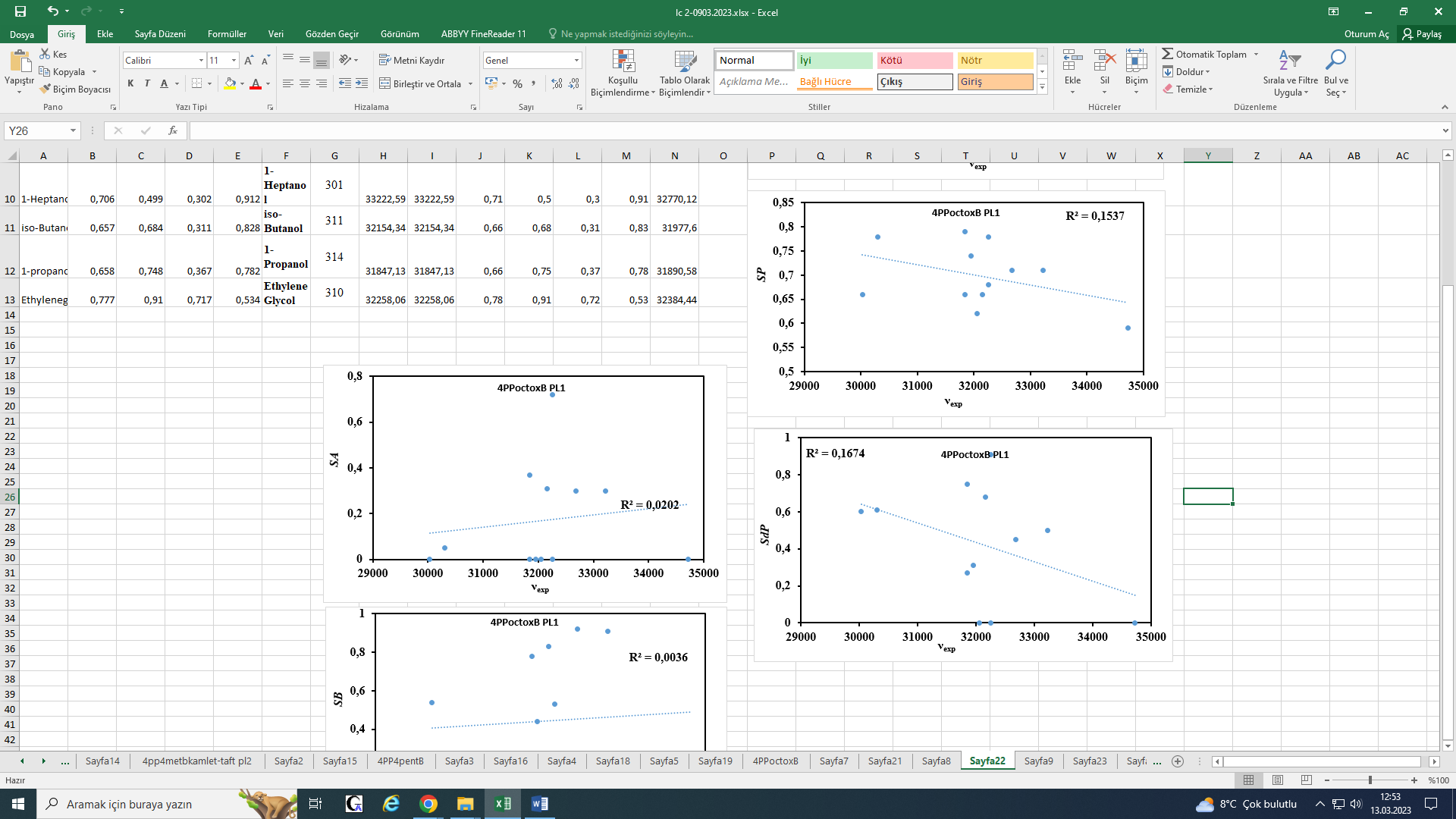

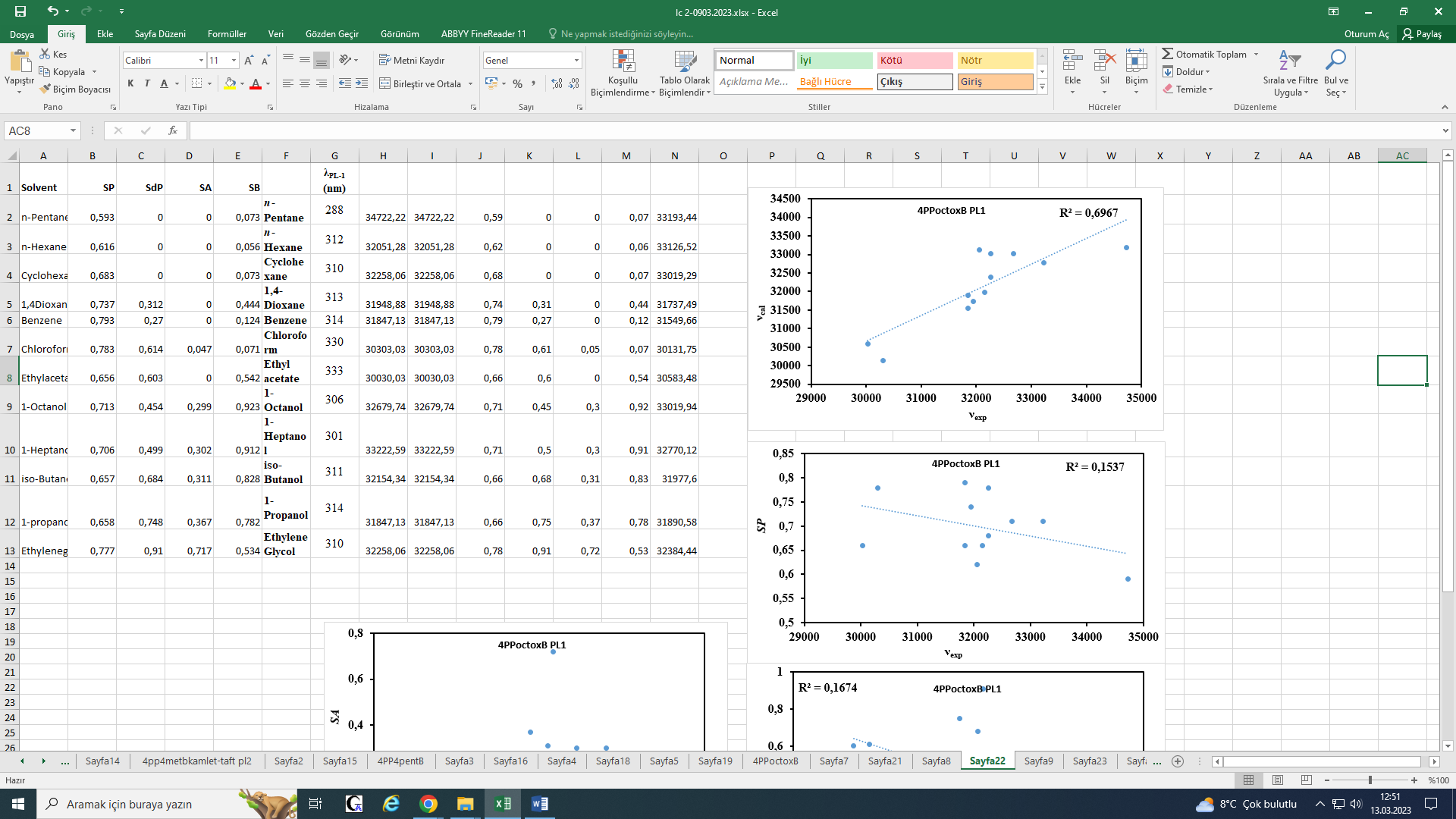

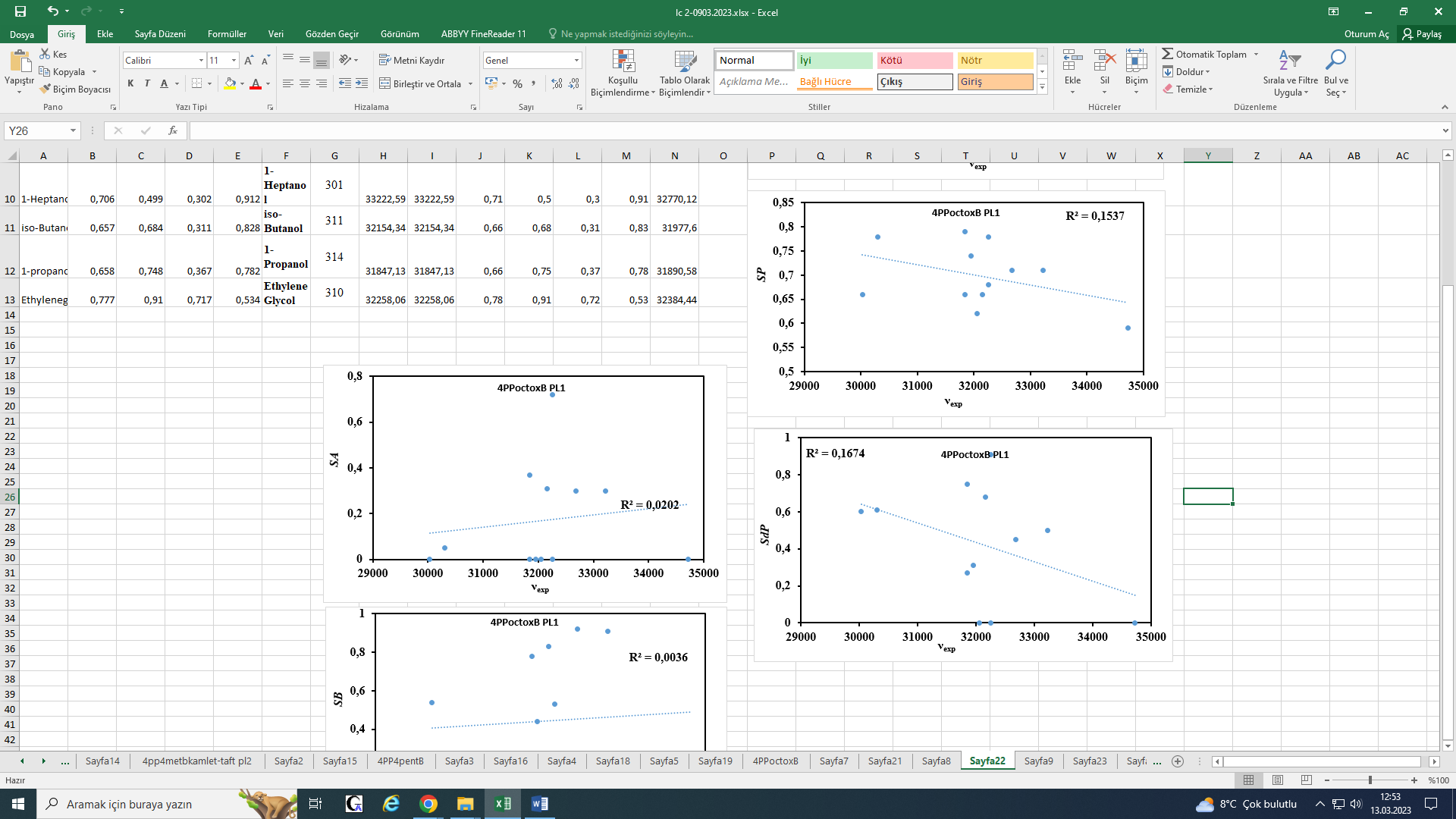

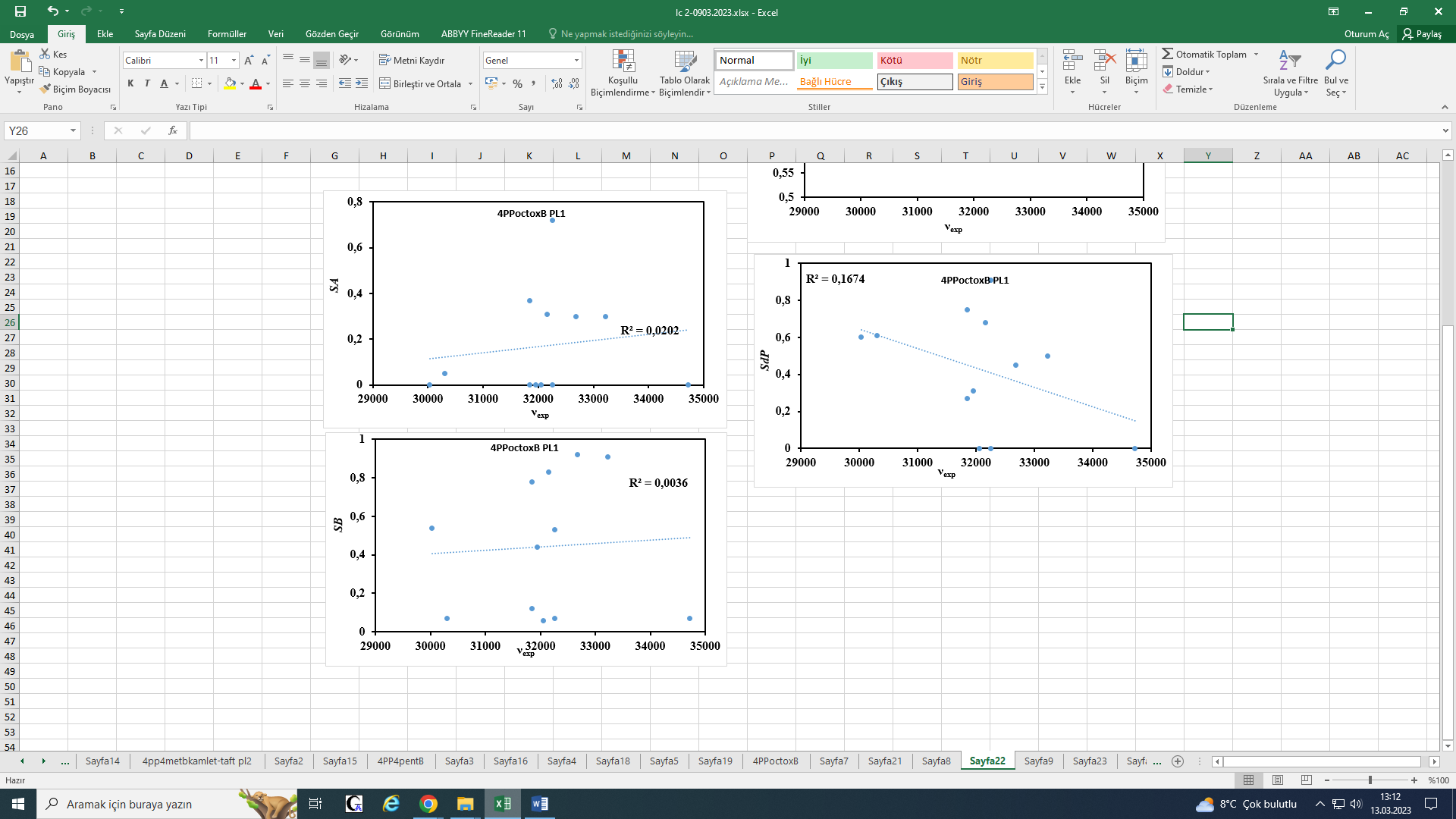


**Figure 19S.** The correlations of *ν_cal_, SP, SdP, SA* and *SB* versus ν_exp_ of λ_PL1_ wavelength of 4PPoctoxB molecule.


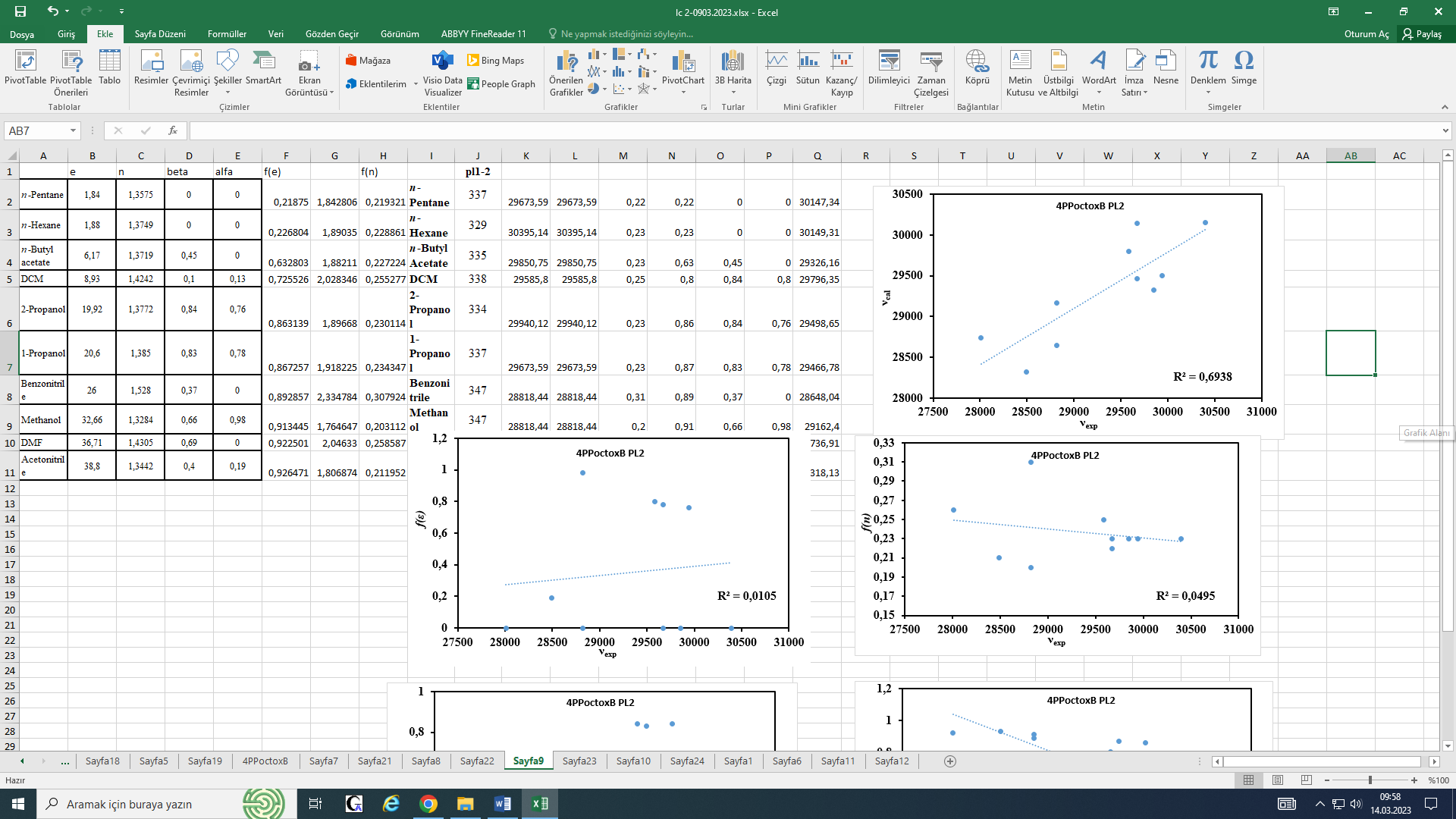

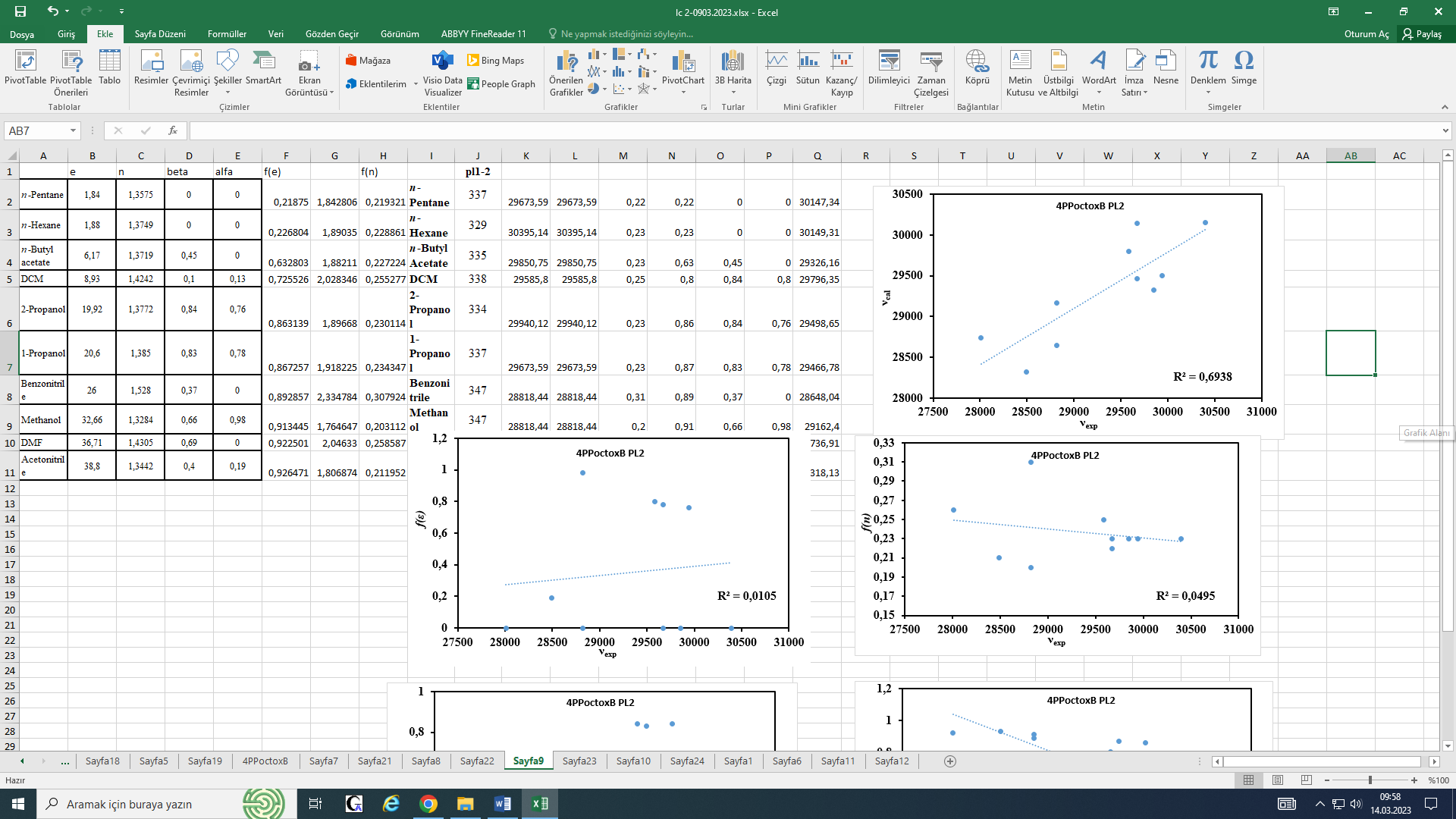

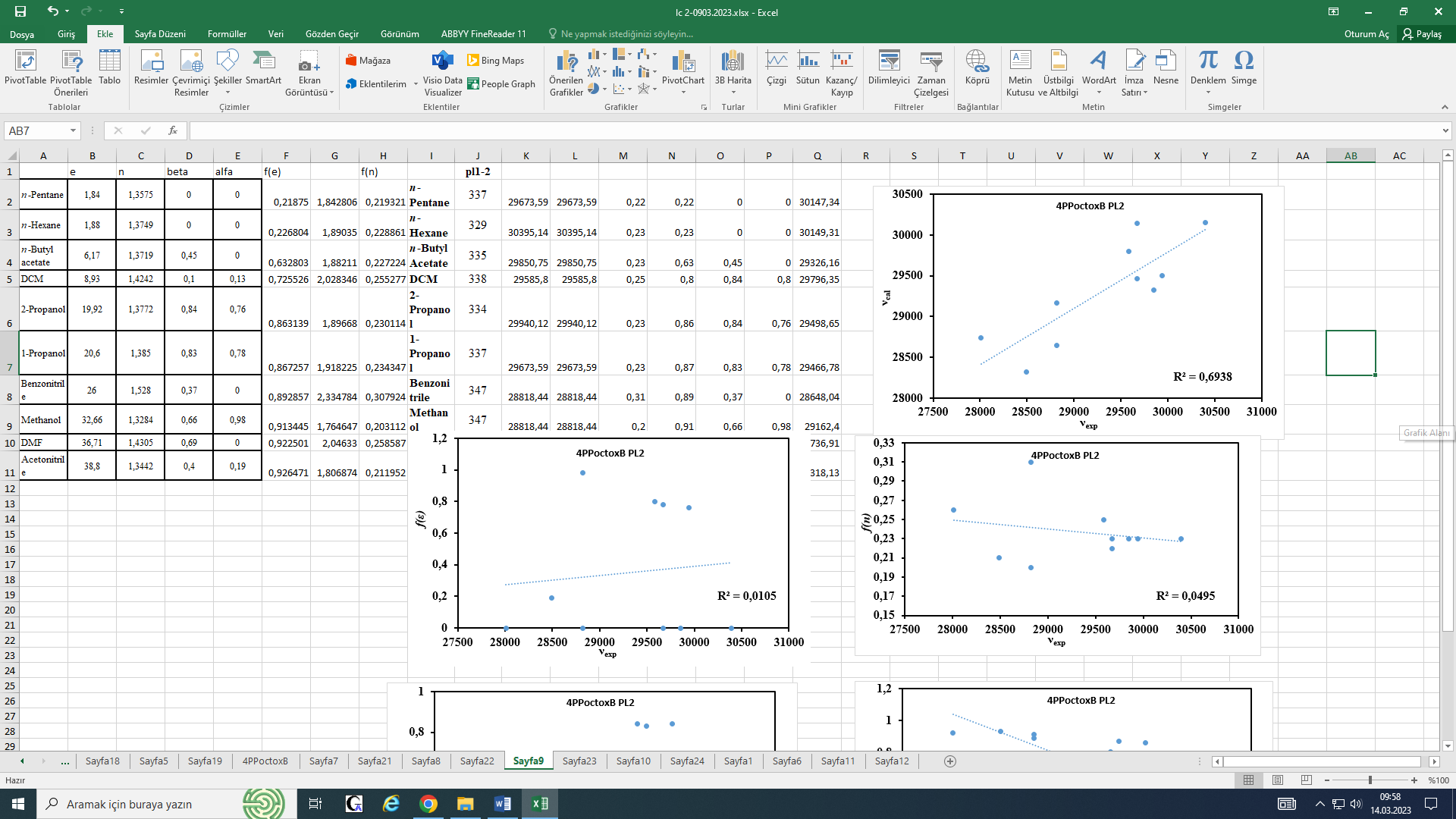

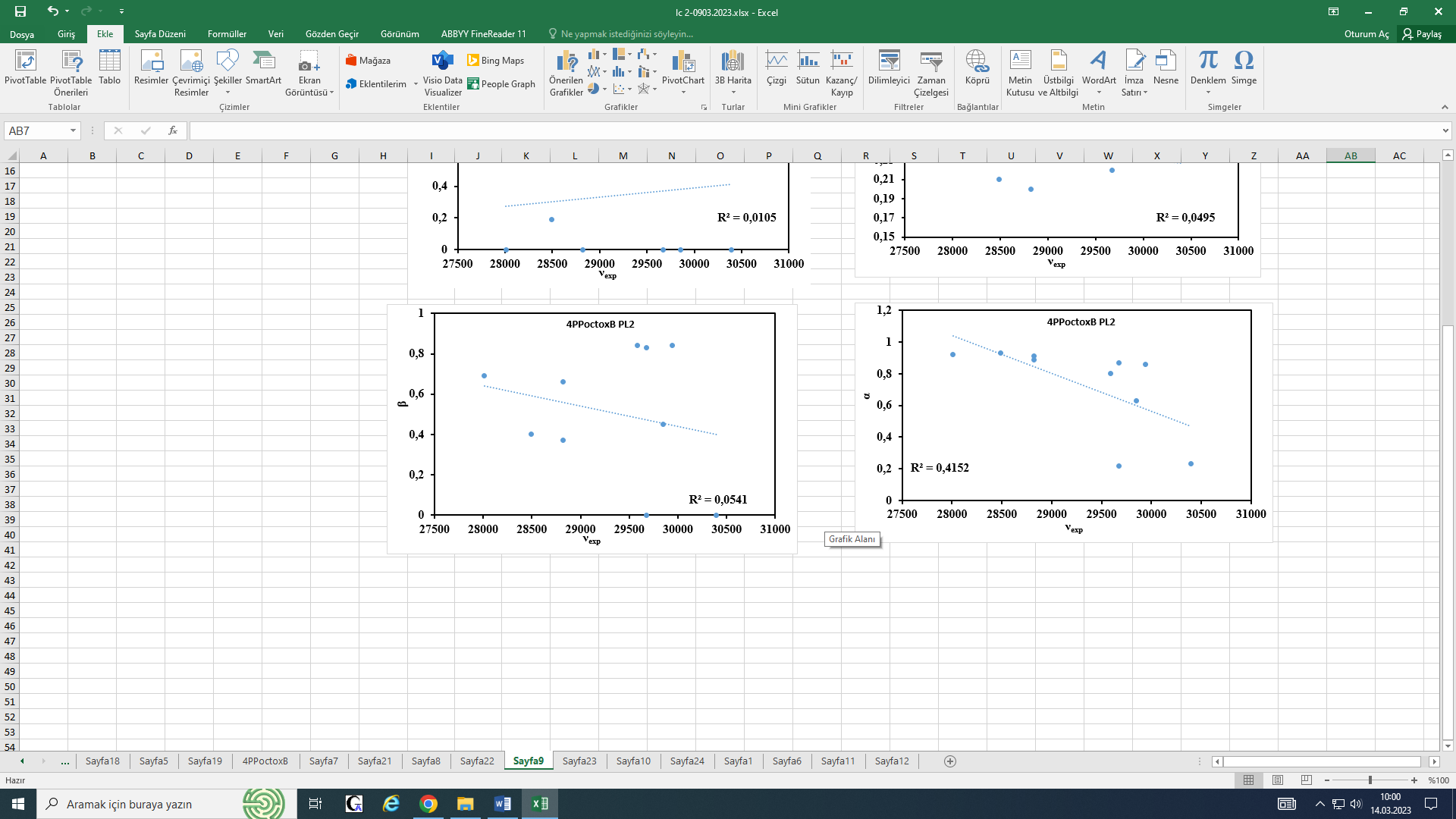

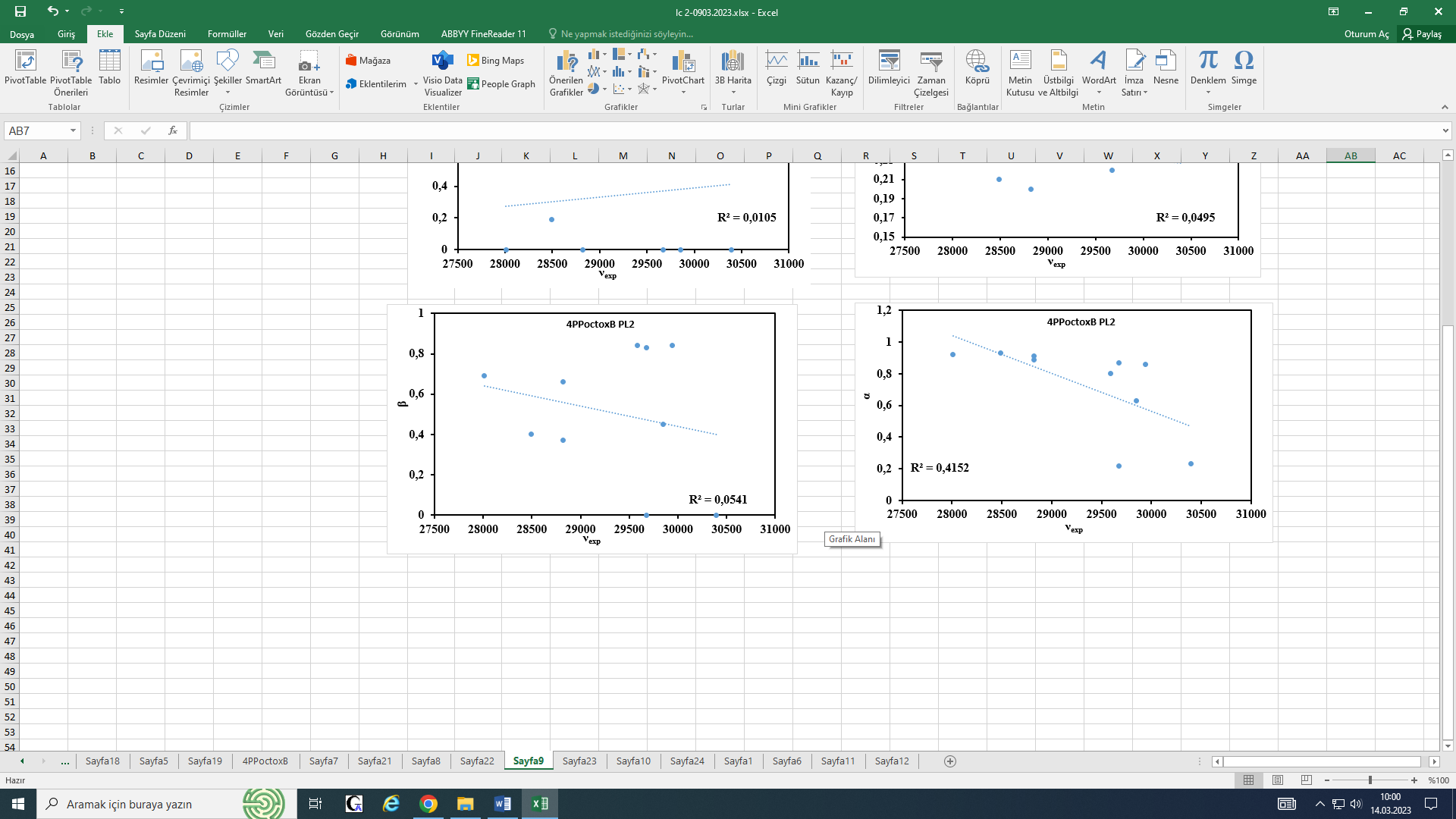


**Figure 20S.** The correlations of *ν_cal_, β, α, f(n)* and *f(ε)* versus ν_exp_ of λ_PL2_ wavelength of 4PPoctoxB molecule.


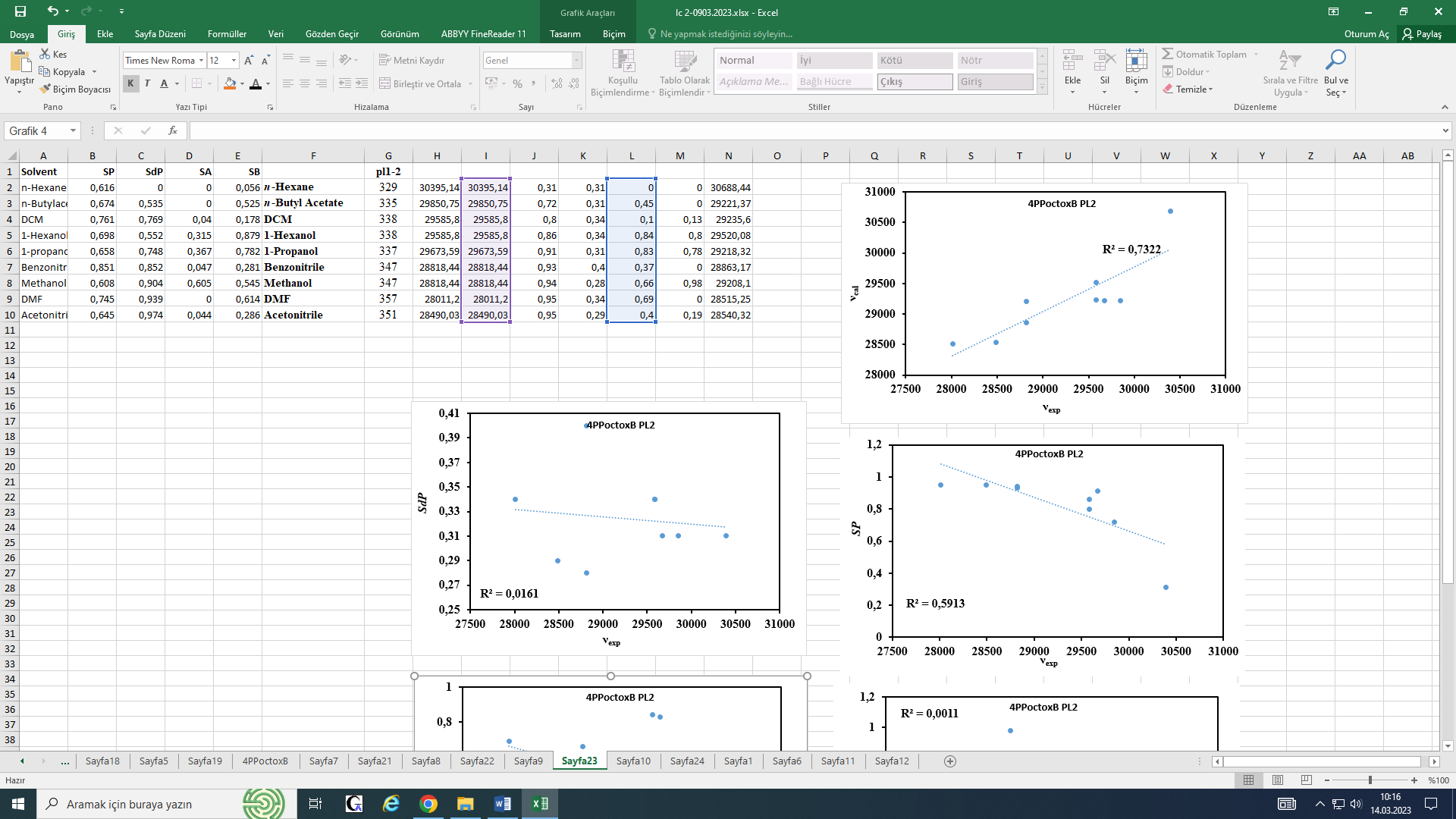

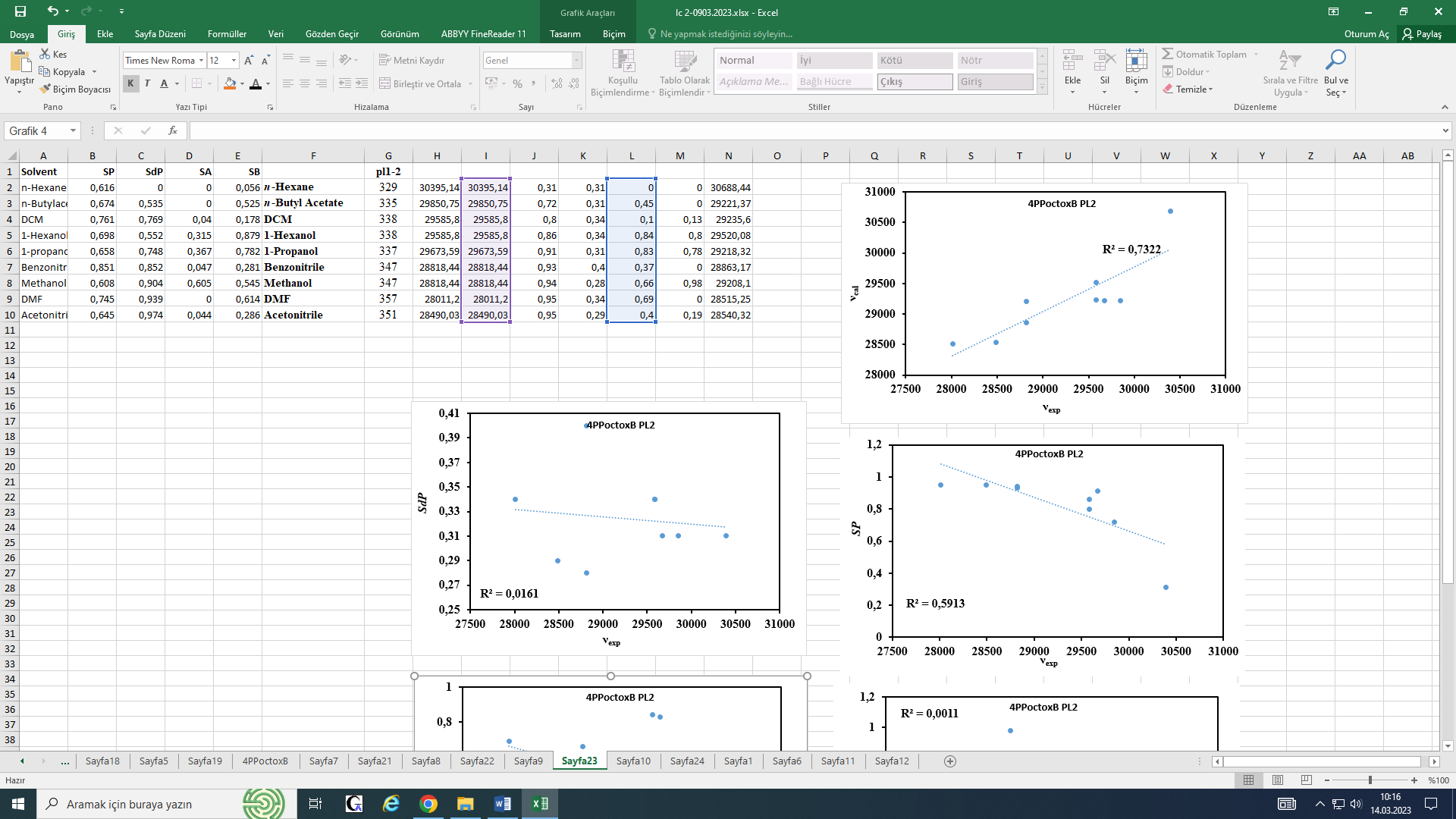

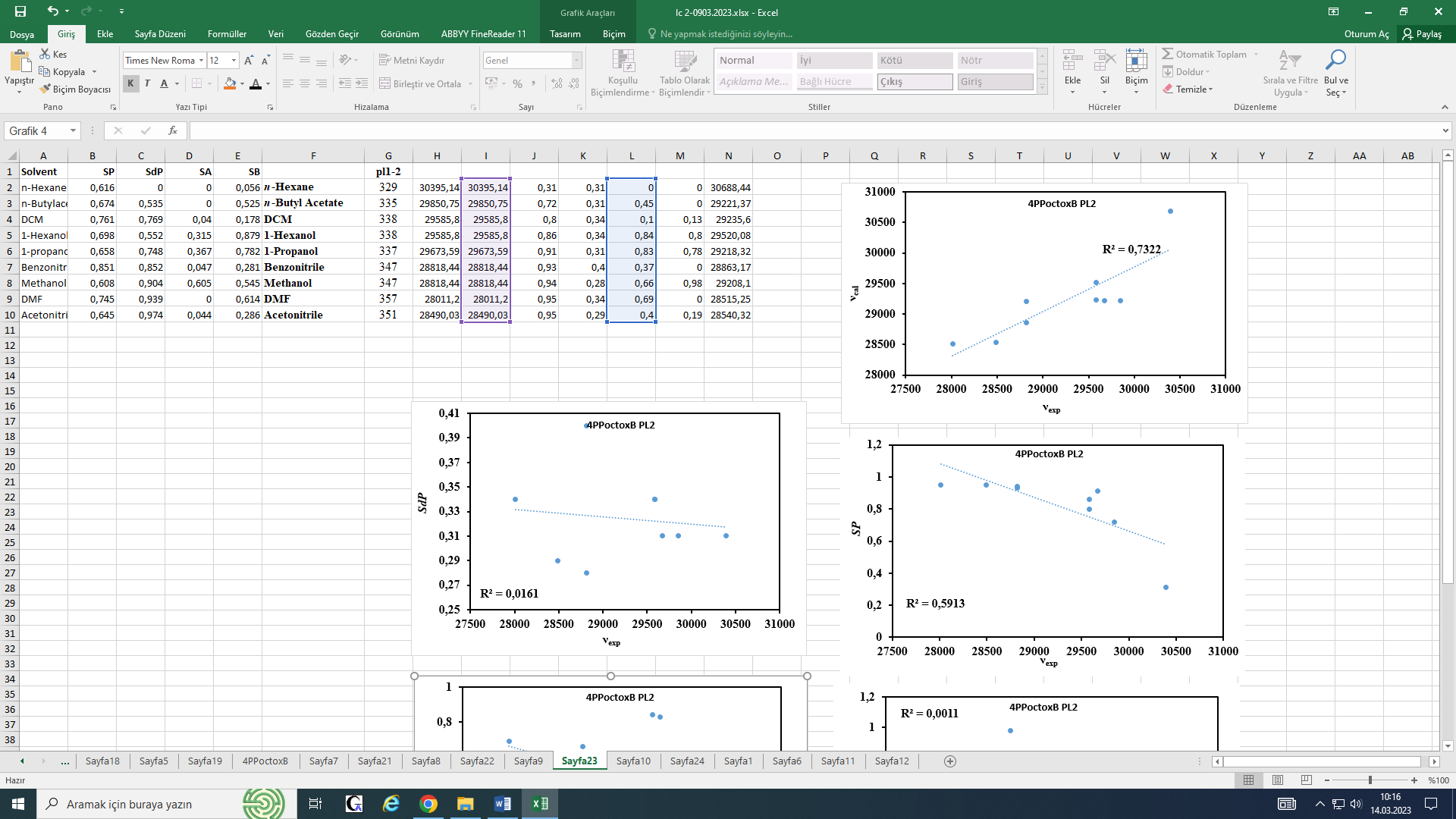


**Figure 21S.** The correlations of *ν_cal_, SP, SdP, SA* and *SB* versus ν_exp_ of λ_PL2_ wavelength of 4PPoctoxB molecule.

**Figure 22S.** The correlations of *ν_cal_, β, α, f(n)* and *f(ε)* versus ν_exp_ of λ_PL3_ wavelength of 4PPoctoxB molecule.

**Figure 23S.** The correlations of *ν_cal_, SP, SdP, SA* and *SB* versus ν_exp_  of λ_PL3_ wavelength of 4PPoctoxB molecule.

**Figure 24S.** The correlations of *ν_cal_, β, α, f(n)* and *f(ε)* versus ν_exp_  of λ_PL4_ wavelength of 4PPoctoxBB molecule.

**Figure 25S.** The correlations of *ν_cal_, SP, SdP, SA* and *SB* versus νexp of λ_PL4_ wavelength of 4PPoctoxB molecule.

**Figure 26S.** The correlations of *ν_cal_, β, α, f(n)* and *f(ε)* versus ν_exp_ of λ_PL5_ wavelength of 4PPoctoxB molecule.

**Figure 27S.** The correlations of *ν_cal,_ SP, SdP, SA* and *SB* versus ν_exp_ of λ_PL5_ wavelength of 4PPoctoxB molecule.

| **Çözücüler** | **^a^** | **^a^** | **^a^** | **^a^** | **^a^** | ^a^ | ***SP^a,b^*** | ***SdP^a,b^*** | ***SA^a,b^*** | ***SB^a,b^*** |
| --- | --- | --- | --- | --- | --- | --- | --- | --- | --- | --- |
| ***n*-Pentane** | **1.84** | **1.3575** | **0.00** | **0.00** | **0.218** | **0.296** | **0.593** | **0** | **0** | **0.073** |
| ***n*-Hexane** | **1.88** | **1.3749** | **0.00** | **0.00** | **0.226** | **0.308** | **0.616** | **0** | **0** | **0.056** |
| **Cyclohexane** | **2.02** | **1.4266** | **0.00** | **0.00** | **0.253** | **0.341** | **0.683** | **0** | **0** | **0.073** |
| **1.4-Dioxane** | **2.21** | **1.4224** | **0.37** | **0.00** | **0.287** | **0.338** | **0.737** | **0.312** | **0** | **0.444** |
| **Benzene** | **2.27** | **1.5589** | **0.10** | **0.00** | **0.297** | **0.416** | **0.793** | **0.27** | **0** | **0.124** |
| **Toluene** | **2.38** | **1.4969** | **0.11** | **0.00** | **0.315** | **0.382** | **0.782** | **0.284** | **0** | **0.128** |
| **o-Xylene** | **2.57** | **1.5054** | **0.16** | **0.00** | **0.343** | **0.387** | **0.681** | **0.997** | **0.025** | **1.062** |
| **Diethyl Ether** | **4.34** | **1.3497** | **0.47** | **0.00** | **0.526** | **0.291** | **0.617** | **0.385** | **0** | **0.562** |
| **Chloroform** | **4.81** | **1.4459** | **0.10** | **0.20** | **0.559** | **0.352** | **0.783** | **0.614** | **0.047** | **0.071** |
| **Ethyl acetate** | **6.02** | **1.3724** | **0.45** | **0.00** | **0.625** | **0.306** | **0.656** | **0.603** | **0** | **0.542** |
| ***n*-Butyl acetate** | **6.17** | **1.3719** | **0.45** | **0.00** | **0.632** | **0.306** | **0.674** | **0.535** | **0** | **0.525** |
| **THF** | **7.58** | **1.4072** | **0.55** | **0.00** | **0.686** | **0.328** | **0.714** | **0.634** | **0** | **0.591** |
| **DCM** | **8.93** | **1.4242** | **0.10** | **0.13** | **0.725** | **0.339** | **0.761** | **0.769** | **0.04** | **0.178** |
| **1-Octanol** | **9.80** | **1.4290** | **0.81** | **0.77** | **0.745** | **0.342** | **0.713** | **0.454** | **0.299** | **0.923** |
| **1-Heptanol** | **11.30** | **1.4240** | **0.94** | **0.83** | **0.774** | **0.339** | **0.706** | **0.499** | **0.302** | **0.912** |
| **1-Hexanol** | **13.00** | **1.4180** | **0.84** | **0.8** | **0.800** | **0.335** | **0.698** | **0.552** | **0.315** | **0.879** |
| **1-Butanol** | **17.40** | **1.3990** | **0.84** | **0.84** | **0.845** | **0.323** | **0.674** | **0.655** | **0.341** | **0.809** |
| **iso-Butanol** | **17.93** | **1.3959** | **0.84** | **0.79** | **0.849** | **0.321** | **0.657** | **0.684** | **0.311** | **0.828** |
| **2-Propanol** | **19.92** | **1.3772** | **0.84** | **0.76** | **0.863** | **0.309** | **0.633** | **0.808** | **0.283** | **0.83** |
| **1-Propanol** | **20.60** | **1.3850** | **0.83** | **0.78** | **0.867** | **0.314** | **0.658** | **0.748** | **0.367** | **0.782** |
| **Ethanol** | **24.55** | **1.3614** | **0.75** | **0.86** | **0.887** | **0.299** | **0.633** | **0.783** | **0.4** | **0.658** |
| **Benzonitrile** | **26.00** | **1.5280** | **0.37** | **0.00** | **0.892** | **0.400** | **0.851** | **0.852** | **0.047** | **0.281** |
| **Methanol** | **32.66** | **1.3284** | **0.66** | **0.98** | **0.913** | **0.276** | **0.608** | **0.904** | **0.605** | **0.545** |
| **DMF** | **36.71** | **1.4305** | **0.69** | **0.00** | **0.922** | **0.343** | **0.745** | **0.939** | **0** | **0.614** |
| **Acetonitrile** | **38.80** | **1.3442** | **0.40** | **0.19** | **0.926** | **0.287** | **0.645** | **0.974** | **0.044** | **0.286** |
| **Ethylene glycol** | **41.40** | **1.4318** | **0.86** | **0.75** | **0.930** | **0.344** | **0.777** | **0.91** | **0.717** | **0.534** |
| **DMSO** | **46.45** | **1.4793** | **0.76** | **0.00** | **0.938** | **0.372** | **0.83** | **1** | **0.072** | **0.647** |
| **Water** | **78.36** | **1.3330** | **0.47** | **1.17** | **0.962** | **0.279** | **0.681** | **0.997** | **0.025** | **1.062** |
| **^a^** **^Reichardt C, 2008. Solvents and Solvent Effects in Organic Chemistry, VCH, New York.^**  **^Reichardt C, 1994. Solvatochromic Dyes as Solvent Polarity Indicators, Chem. Rev., 94: 2319–2358.^**  **^Reichardt, C., Welton, T.: Solvents and Solvent Efects in Organic Chemistry. Wiley, New York (2011)^**  **^Kamlet, M. J. And Taft, R. W, 1982. Linear solvation energy relation ships. 20. Intravs.^**  **^İnter molecular hydrogen bond ing by some 2-nitroaniline and 2-nitro phenol derivatives, J. Org. Chem., 47(9): 1734-1738.^**  **^Kamlet, M. J. and Taft, R. W, 1976, The solvatochromic comparison method. 2. The. alpha.-scale of solvent hydrogen-bond donor (HBD) acidities, J. Am. Chem. Soc., 98(10): 377, 2886–2894.^**  **^Kamlet, M.J, Abboud J.L.M, Abraham, M.H. and Taft, R.W, 1983, Linear Solvation Energy Relationships, J Org Chem., 48: 2877-2888.^**  **^Kamlet, M.J, Abboud, J.L.M. and Taft, R.W., 1977, The solvatochromic comparison method. 6. The .pi.* scale of solvent polarities, J. Am. Chem. Soc., 99 (18): 6027–6038.^**  **^b^** **^Reichardt C, 2008. Solvents and Solvent Effects in Organic Chemistry, VCH, New York.^**  **^Catalán, J.: Toward a generalized treatment of the solvent effect based on four empirical scales: dipolarity (SdP, a new scale), polarizability (SP), acidity (SA), and basicity (SB) of the medium. J. Phys. Chem. B 113(17), 5951–5960 (2009)^** | | | | | | | | | | |

**Table 1S.** The parameters used in LSERs calculatio

1. Corresponding Author: Tel.: +90 434 2220020 (2313); fax: +90 434 2229143

   E-mail address: ygsidir@beu.edu.tr, yadigar.gulseven@gmail.com (YGS) [↑](#footnote-ref-1)
